# Thiol-Retaining N‑Terminal Cysteine Chemistry for Dual Modification and Bicyclic Peptide Construction

**DOI:** 10.1021/jacs.6c01648

**Published:** 2026-04-20

**Authors:** Junjie Liu, Shixiang Duan, Yang Huang, Ming-Yi Jian, Hsuan Suan Lee, Gaocan Dai, Chuanliu Wu, Yi-Lin Wu, Yu-Hsuan Tsai

**Affiliations:** † Institute of Molecular Physiology, 551667 Shenzhen Bay Laboratory, Shenzhen 518132, China; ‡ Guizhou Provincial Key Laboratory of Innovation and Manufacturing for Pharmaceuticals, School of Pharmacy, 66367 Zunyi Medical University, Zunyi 563003, China; § Department of Applied Chemistry, 34914 National Yang Ming Chiao Tung University, Hsinchu 30010, Taiwan; ∥ Department of Chemistry, College of Chemistry and Chemical Engineering, the MOE Key Laboratory of Spectrochemical Analysis and Instrumentation, State Key Laboratory of Physical Chemistry of Solid Surfaces, Xiamen University, Xiamen 361005, China; ⊥ Center for Emergent Functional Matter Science, National Yang Ming Chiao Tung University, Hsinchu 30010, Taiwan; 6 Tsinghua University, Beijing 100084, China; 7 Shenzhen Medical Academy of Research and Translation (SMART), Shenzhen 518107, China

## Abstract

N-terminal cysteine presents a uniquely reactive 1,2-aminothiol motif that enables site-specific modification of peptides and proteins composed solely of canonical amino acids. For both *in vitro* and *in vivo* applications, this operationally simple chemistry is an attractive alternative to bioorthogonal strategies that require noncanonical handles. However, most 1,2-aminothiol-selective reagents irreversibly consume both amine and thiol, yielding inert heterocycles and limiting downstream diversification. Here, we report a thiol-retaining N-terminal cysteine chemistry by repurposing 2-((alkyl**t**hio)­(**a**ryl/alkyl)**m**ethylene)**m**alononitriles (TAMMs) to favor a thiol-containing conjugate over the canonical cyclized product. Through rational design and mechanistic analysis of *ortho*-substituted TAMMs (*o*-TAMMs), we established steric hindrance as a key determinant of thiol-retaining adduct stability. The retained thiol provides an immediate handle for sequential dual modification of peptides and proteins. Extending this concept to scaffold design, an electrophile-equipped *o*-TAMM cross-linker converts CX_m_CX_n_C peptides into compact bicyclic architectures comprising a thioether ring and a disulfide ring. Phage display using this chemistry affords high-affinity bicyclic binders of KEAP1, and the disulfide can be transformed into a redox-stable thioacetal without a loss of affinity. Collectively, this work establishes a mechanistically grounded platform for thiol-retaining N-terminal cysteine ligation, enabling dual functionalization and access to structurally distinctive bicyclic peptides.

## Graphical abstract

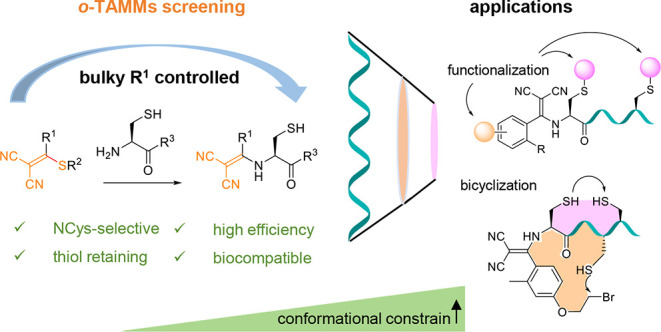

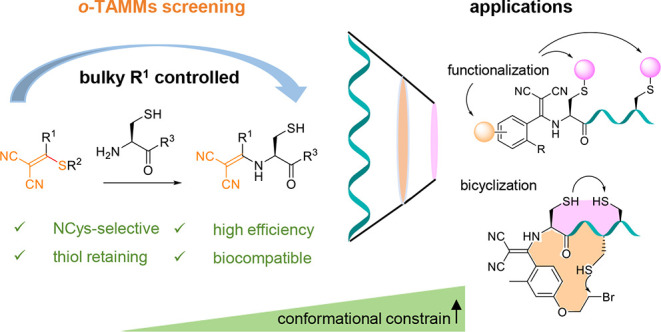

## Introduction

Precise chemical modification of peptides and proteins underpins applications ranging from mechanistic biology to diagnostics and therapeutics.^1–7^ Many established bioorthogonal reactions rely on noncanonical functionalities introduced via enzymatic tagging or engineered translation systems, which can be costly and suffer from reduced protein yields.^7–10^ In contrast, strategies that directly target canonical amino acids are operationally simple and readily deployed across diverse biological settings.^11^ In this context, N-terminal cysteine is a privileged handle.^12^ Its 1,2-aminothiol motif is distinctive in native proteomes because protein translation is initiated from methionine, enabling high chemoselectivity with minimal background interference.

Such a feature has inspired the development of reagents that preferentially engage N-terminal cysteine, such as 2-cyanobenzothiazole (CBT),^13^ 2-formylphenylboronic acid,^14^ 2-benzylacrylaldehyde,^15^ cyclopropenone,^16^ and NHS-activated acrylamide.^17^ Despite their structural diversity, these reagents share a common mechanistic outcome: tandem nucleophilic additions of the thiol and amine that irreversibly consume both functionalities to form chemically inert heterocycles. As a result, ligation at N-terminal cysteine typically precludes further derivatization, limiting modular diversification and access to higher-order architectures.^12^

Native chemical ligation represents a notable exception, as it can furnish thiol-containing products.^18^ However, the instability of thioester substrates, the reliance on thiol additives, slow kinetics (ca. 0.1 M^–1^ s^–1^), and frequently denaturing conditions restrict its direct use for modifying proteins on living cell surfaces.^19^ Consequently, a general and rapid 1,2-aminothiol ligation that preserves thiol functionality remains a long-standing unmet need.

We recently reported a mechanistic analysis of the reaction between 2-((alkyl**t**hio)­(**a**ryl)**m**ethylene)**m**alononitriles (TAMMs, **1**; Figure 1a) and 1,2-aminothiols (**2**),^20^ revealing a *kinetically accessible* thiol-containing enamine intermediate (**5**).^21^ Stabilizing this intermediate would represent a significant conceptual advance; it would retain the nucleophilic thiol at the N-terminus, enabling direct derivatization and compact cross-linking (Figure 1b). In particular, pairing the TAMM-derived linkage with the liberated thiol near the N-terminus offers a route to tight bicyclic topologies (thioether and disulfide rings) from CX_m_CX_n_C peptides (Figure 1c). Such compact architectures can reduce conformational entropy and increase scaffold rigidity, features that are advantageous for discovering high-affinity peptide binders.^22–24^

**Figure 1:**
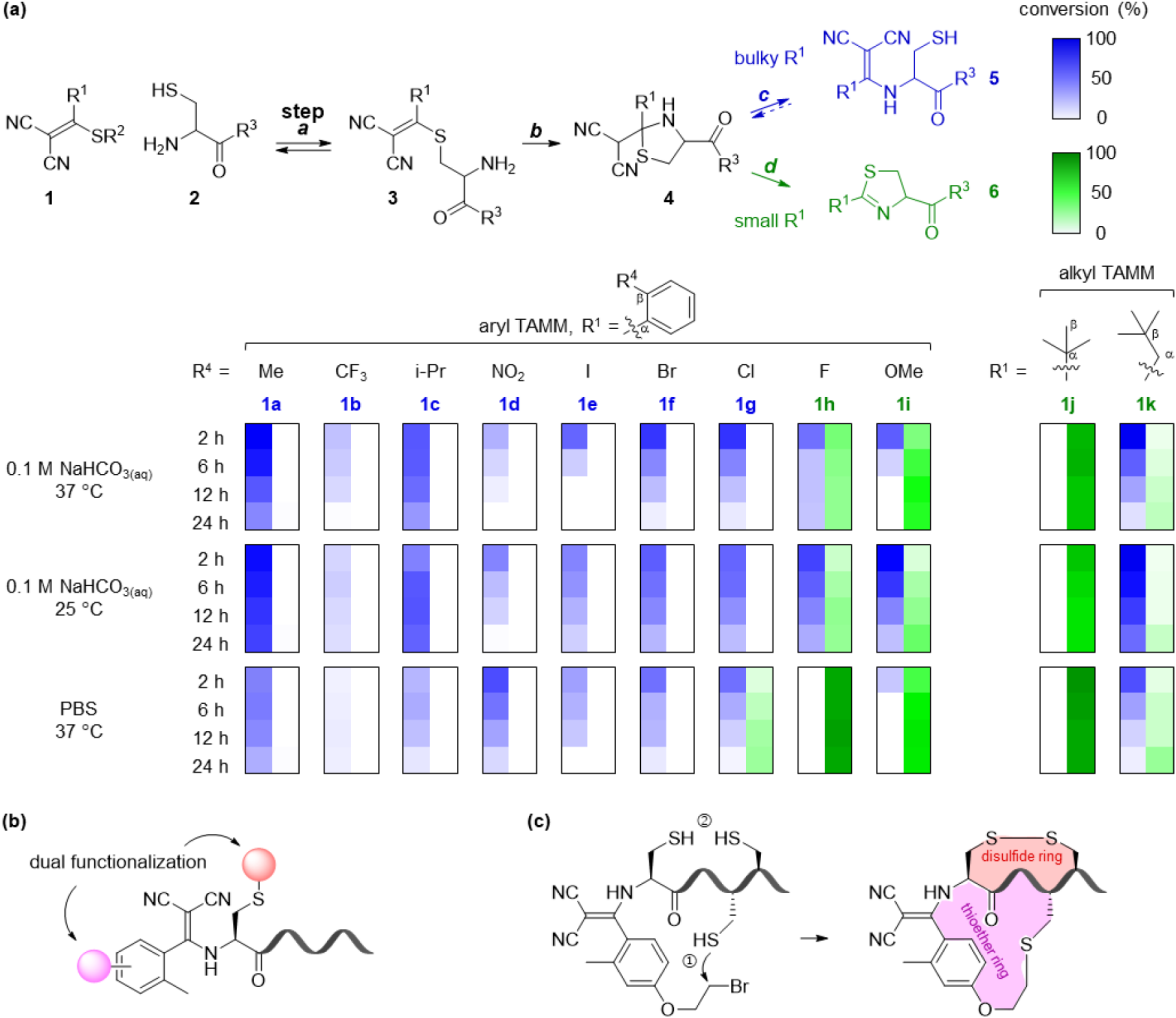
Thiol-retaining N-terminal cysteine modification enabled by steric regulation (a) with applications in dual functionalization (b) and construction of compact bicyclic topologies (c). Conversions were quantified by HPLC for reactions of 50 μM peptide **2x** and 200 μM **1** in the presence of 500 μM TCEP and 500 μM Ac-Cys-OMe under the indicated conditions.

A key limitation, however, is that enamine **5** is typically short-lived, rapidly converting into the thermodynamically favored dihydrothiazole **6**, which irreversibly consumes the thiol and prevents practical exploitation of this intermediate. We reasoned that this outcome is not inevitable but rather reflects conformational and kinetic preferences embedded in the TAMM scaffold. Here, we show that steric hindrance can be leveraged as a reagent-level design principle to reshape the reaction landscape. By introducing steric congestion at the β-position of TAMMs, we suppress thiol-consuming cyclization pathways and stabilize thiol-retaining enamine adducts, enabling their isolation and downstream functionalization.

## Results

### Reactions of 1,2-Aminothiol with Sterically Hindered TAMM to Afford Thiol-Bearing Products

Mechanistically, TAMM **1** first undergoes thiol exchange with 1,2-aminothiol **2** to form **3** (step *a*), which rapidly cyclizes to thiazolidine **4** (step *b*). Conversion of **4** to **5** (step *c*) is kinetically favored but reversible, while the formation of thermodynamically more stable **6** (step *d*) is slower yet effectively irreversible. Consequently, **5** ultimately becomes **6**. Consistent with step *c* being faster than step *d*, accelerating step *a* increases the extent of **5** accumulation, although only transiently.^21^ Inspired by prior work showing that steric hindrance adjacent to a 1,2-aminothiol can affect the lifetime of a CBT condensation intermediate,^25^ we set out to evaluate whether increasing congestion on the TAMM scaffold could stabilize the thiol-retaining adduct **5** (Figure S1).

Consistent with this idea, the reaction of *ortho*-methylphenyl TAMM **1a** and peptide **2x** (H-CGGGKGW–OH) in 0.1 M NaHCO_3(aq)_ produced thiol-containing enamine **5ax** as the sole detectable product (Figure 1a). Screening across other commonly used buffers and pH values revealed 0.1 M NaHCO_3(aq)_ as the optimal condition for clean thiol-retaining enamine formation (Figure S2). For comparison, we also evaluated the reaction in PBS (pH 7.4) at 37 °C, which served as the standard condition in our previous kinetic and mechanistic studies of TAMM condensation.^21^ Ac-Cys-OMe was included in all reaction conditions to control thiol-exchange equilibria and make the protocol broadly applicable to substrates containing internal cysteine residues. In such substrates, TAMM can reversibly exchange with side-chain thiols to form internal-cysteine TAMM adducts.^20,21^ Maintaining Ac-Cys-OMe in excess suppresses the accumulation of these adducts by shifting the equilibrium toward the Ac-Cys-OMe-associated TAMM species (e.g., **1a**), so that any internal-cysteine adducts that do form are driven back through thiol exchange with Ac-Cys-OMe (Le Châtelier’s principle). This helps confine stable modification to the N-terminal cysteine at reaction completion. Although peptide **2x** contains no internal cysteine, Ac-Cys-OMe was retained to define a general set of conditions readily transferable to peptides and proteins that do.

We next assessed the reactivity of TAMM **1a** in the absence of an N-terminal cysteine. Specifically, **1a** was reacted with two model peptides, TQCDEW and SNHKRW, which contain different nucleophilic residues and, in the case of TQCDEW, also an internal cysteine residue. Under our standard conditions (50 μM peptide, 200 μM **1a**, 500 μM TCEP, 500 μM Ac-Cys-OMe, 0.1 M NaHCO_3(aq)_, 37 °C), no detectable reaction was observed with either peptide (Figure S3). These results are consistent with our previous studies^20,21^ and further confirm the high selectivity of TAMM chemistry for N-terminal cysteine.

The identity of **5ax** was supported by alkylation with iodoacetamide (Figure S4). Notably, **5ax** showed no detectable conversion to **6ax** in solution over 48 h (Figures S5 and S6). We then synthesized a series of *ortho*-substituted TAMMs (*o*-TAMMs) **1b**–**1i** and evaluated their reactions with **2x** (Figure 1 and S7–S14). Bulky substituents (**1c**-**1g**) consistently favored the formation of **5** (Figures S8–S12). Intriguingly, **1b** (R^4^ = CF_3_) showed only partial conversion (Figure S7). In PBS, **1b** rapidly consumed **2x** but formed unidentified products rather than **5** or **6**, whereas in NaHCO_3(aq)_ both **1b** and **2x** remained detectable after 24 h, with only trace amounts of **5bx** observed at all examined time points. These results suggest that the *ortho*-CF_3_ substituent diverts the reaction toward competing noncanonical pathways. On the other hand, *o*-TAMMs **1h** and **1i** with small substituents gave dihydrothiazole **6** as the exclusive product (Figures S13 and S14). We further examined two alkyl TAMMs, **1j** and **1k**, derived from pivalic acid and 3,3-dimethylbutanoic acid, respectively. TAMM **1j** yielded only **6jx** (Figure S15), whereas **1k** produced a mixture of **5kx** and **6kx** after overnight incubation (Figure S16). Although **5ax**–**5ag** formed selectively, their abundance gradually decreased upon prolonged incubation (Figure 1a). Stability studies of **1a**, **2x**, and **5ax** showed that both **1a** and **5ax** slowly decomposed under extended alkaline incubation, whereas they were more stable at physiological or acidic pH and were not measurably destabilized by the presence of excess glutathione (Figure S6). Thus, the reduced persistence of **5** under alkaline conditions reflects general, condition-dependent decomposition rather than preferential attack by an external thiol or simple conversion to **6**. Nevertheless, the reagent and product remained sufficiently stable for hours, exceeding the time required to reach maximum formation of **5ax**, which was attained at about 2 h in NaHCO_3(aq)_ at 37 °C.

Collectively, these data indicate that β substitution is a key determinant of product selectivity. In addition, the reaction medium also influences the product distribution: NaHCO_3(aq)_ favors the formation of the thiol-containing enamine **5**, whereas PBS favors the formation of dihydrothiazole **6**. This trend is particularly evident for compounds **1g**–**1i**. Overall, bulky β-substituents likely suppress the reverse conversion of **5** to **4** and/or slow the subsequent formation of **6**, enabling the isolation of **5** as a stable thiol-retaining product.

### Mechanistic Investigation of *o*-TAMM Properties

Bulky *o*-TAMMs also display more complex ^1^H NMR behavior, consistent with restricted conformational dynamics. At 25 °C in CDCl_3_, *o*-TAMMs **1a**–**1g** display a doublet-like resonance for the methyl ester protons, whereas these protons appear as a singlet in **1h**–**1k** (see Supporting Information). In DMSO-*d*_6_, several resonances of **1a** appear as two sets of peaks (ca. 55:45) at 25 °C but coalesce at 75 °C (Figures 2a and S17a). Similar behavior was observed for *ortho*-iodo-substituted **1e** (Figure S17b), as well as *ortho*-nitro-substituted **1d** (Figure S18a) and **1l** (R^2^ = CH_2_CF_3_, Figure S18b). By contrast, *o*-TAMMs bearing smaller substituents, such as **1h** (R^4^ = F) and **1i** (R^4^ = OMe), show peak splitting only at low temperature (Figures 2b, S19 and S20). These data indicate that bulky *ortho* substituents restrict internal rotation at room temperature, producing conformer populations that are slow on the NMR time scale.

**Figure 2:**
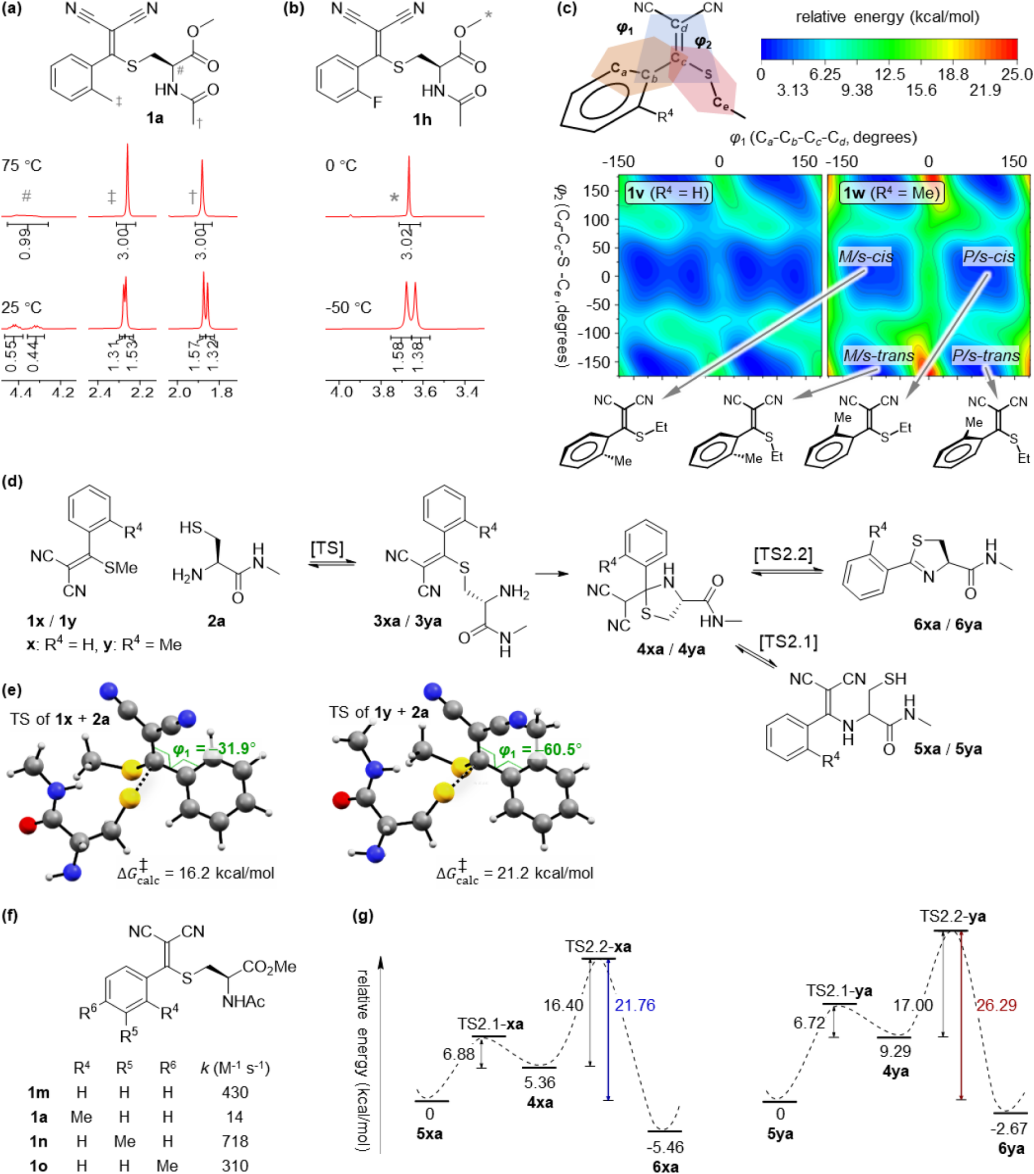
Mechanistic Investigation of *o*-TAMM. (a, b) Variable-temperature ^1^H NMR spectra, showcasing different rotational barriers of **1a** and **1h**. See Figures S17 and S19 for the full spectra. (c) Calculated energy profiles of **1v** (R^2^ = Et, R^4^ = H) and **1w** (R^2^ = Et, R^4^ = Me), confirming that a bulky ortho substituent increases the conformational interconversion barrier. (d) Model thiolate-exchange reactions of **1x** (R^2^ = Me, R^4^ = H) or **1y** (R^2^ = R^4^ = Me) with **2a** were used for DFT calculations of transition states and reaction barriers. (e) *Ortho*-methyl substitution increases the activation barrier for thiolate exchange, consistent with reduced aryl–dicyanovinyl conjugation in the transition state (TS). (f) Experimentally measured consumption rate constants of representative TAMMs in a Tris buffer (pH 8.5) at 37 °C. (g) Relative energies of **4**–**6** and the corresponding transition states rationalize the stabilization of *ortho*-methyl substituted **5** at room temperature by increasing the effective barrier for conversion from **5** to **4**, consequently suppressing the formation of **6**. The relative energies of TS2.1 were estimated from the interconversion of the corresponding deprotonated anionic intermediates (Figure S24).

This behavior parallels that of enamine **5**, where conjugation between the nitrogen lone pair and the dicyanovinyl acceptor increases the C–N bond order and slows rotation.^21^ To further examine how the *o*-TAMM-derived enamine linkage differs from a conventional amide bond, we synthesized representative enamine analogues derived from unsubstituted TAMM and *ortho*-methyl-substituted TAMM, together with the corresponding amide-linked analogues (Figure S21). Because the enamine from unsubstituted TAMM is short-lived, its thiol group was capped with iodoacetamide to prevent conversion to dihydrothiazole **6**;^21^ for consistency, the thiol groups in the other enamine analogues were capped in the same manner. Variable-temperature ^1^H NMR shows that both enamine analogues exist as two conformers (ca. 65:35) at room temperature that coalesce upon heating, whereas the corresponding amide-linked analogues show no resolvable conformational isomers even at −50 °C (Figure S21). These results demonstrate that the (dicyanomethylene)­enamine linkage imposes a conformational constraint and rigidity substantially greater than those of an amide linkage. Notably, the signals of the *o*-Me enamine analogue do not coalesce until 75 °C, whereas those of the unsubstituted enamine analogue already coalesce at 50 °C, consistent with a higher rotational barrier and greater stability for enamine **5** derived from *o*-TAMM.

Computational analysis^26^ further supports a steric origin for these effects. Conformational scans of model compounds **1v** (R^2^ = Et, R^4^ = H) and **1w** (R^2^ = Et, R^4^ = Me) reveal low-energy clusters corresponding to P/M atropisomers and *s-cis*/*s-trans* forms (Figure 2c). Steric hindrance increases the barrier for *P*/*s-cis* ⇌ *M*/*s-cis* interconversion from 3.9 kcal/mol for **1v** to 9.7 kcal/mol for **1w**, consistent with the distinct conformer populations observed by ^1^H NMR. Steric congestion also impacts reactivity. DFT calculations^27,28^ on thiolate exchange between cysteine methyl amide (Cys-NHMe, **2a**) and TAMM **1x** (R^2^ = Me, R^4^ = H) or **1y** (R^2^ = R^4^ = Me) indicate a higher activation free energy (21.2 vs 16.2 kcal/mol) for the sterically congested system (Figure 2d). The increase correlates with a larger aryl–dicyanovinyl dihedral angle (60.5° vs 31.9°; Figure 2e), which weakens π-conjugative stabilization in the transition state (TS). Experimentally, **2x** reacts more slowly with *o*-TAMM **1a** than unsubstituted, *meta-,* or *para*-substituted TAMMs (i.e., *m*-TAMM **1n** and *p*-TAMM **1o**; Figure 2f and S22). Finally, steric congestion increases the effective barrier for malononitrile elimination. While the intrinsic elimination barrier (TS2.2) is similar for R^4^ = H (16.4 kcal/mol) and R^4^ = Me (17.0 kcal/mol; Figure 2g and S23), *ortho*-methyl substitution substantially increases the energy gap between **5** and **4** (Figure S24). Consequently, the effective barrier for elimination increases from 21.8 to 26.3 kcal/mol (see Supporting Information for discussion), shifting the reaction outcome toward **5**. Consistent with a higher barrier, heating **5ax** to 90 °C could yield some **6ax** (Figure S25). Together, these experimental and computational results explain how steric hindrance stabilizes enamine **5**, suppresses dihydrothiazole formation at room temperature, and generates a more conformationally constrained linkage than that of a conventional amide.

### *o*-TAMM for Peptide and Protein Functionalization

We next evaluated *o*-TAMMs for the site-specific modification of peptides and proteins. *O* *rtho*-methyl TAMMs **1a** and **1p** were reacted with peptides **2x** (H–CGGGKGW–OH) and **2y** (H–CPVRYGWDMRC–OH), as well as zHER2 and SUMO proteins engineered with an N-terminal cysteine (Figure 3a). In each case, a high conversion to the thiol-retaining enamine products was observed. TAMM **1p** further incorporates a *para*-homopropargyl group, enabling modular installation of diverse functionalities using commercially available azides through Cu-catalyzed azide–alkyne cycloaddition (CuAAC). As a proof of concept, peptide conjugate **5px** (from **1p** and **2x**) underwent sequential derivatization: maleimide capping of the liberated thiol afforded **7px**, followed by CuAAC with 2-azidoethanol to yield dual-functionalized **8px** (Figures 3b and S26). The same strategy was validated on peptide **2y**, which contains two cysteines, in which both thiols were efficiently modified by maleimide (Figures 3b and S27). Together, these results establish *o*-TAMMs as a general platform for thiol-retaining N-terminal cysteine ligation and dual modification.

**Figure 3:**
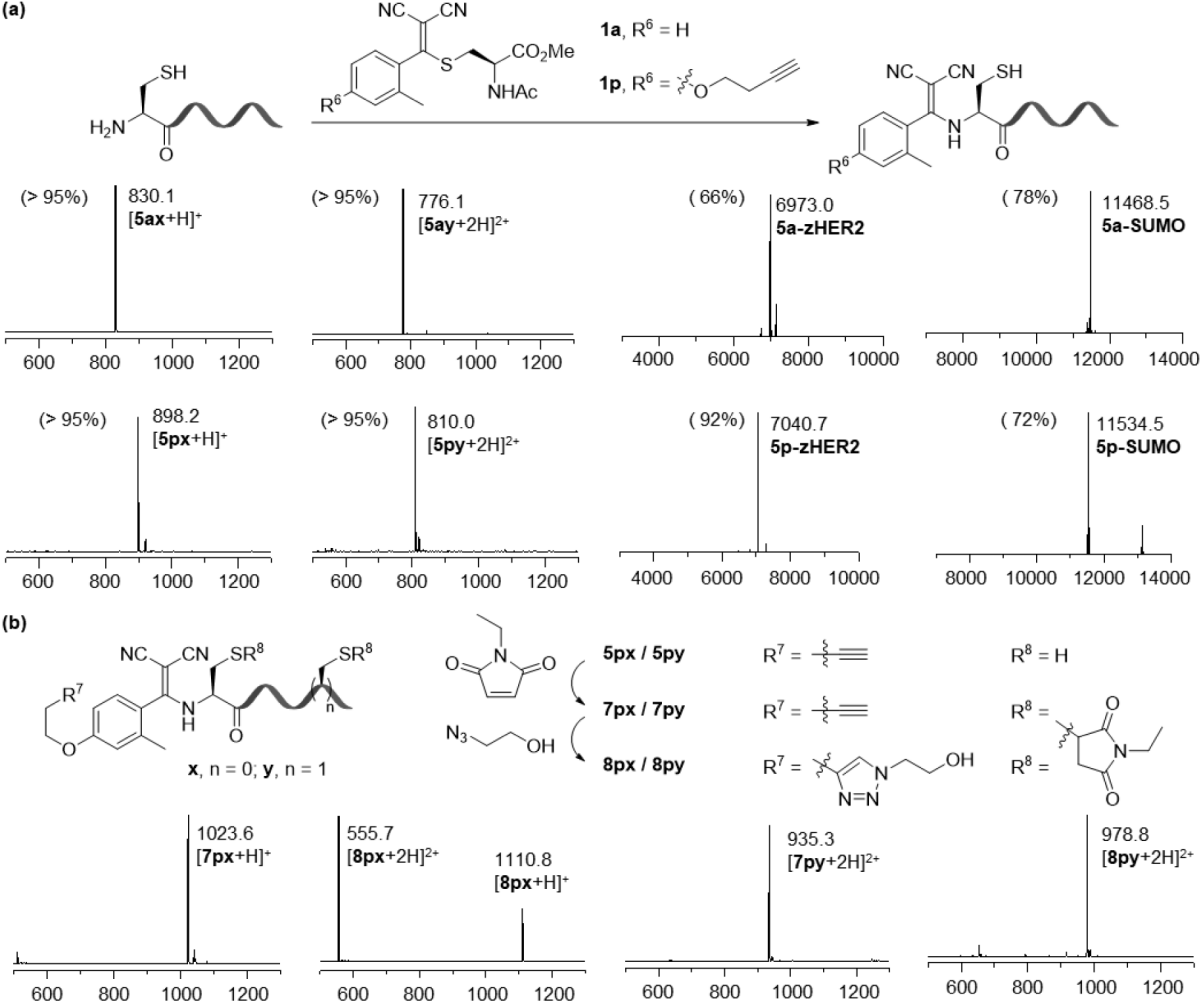
Site-specific functionalization of peptides and proteins via thiol-retaining N-terminal cysteine chemistry. (a) Formation of thiol-retaining enamine conjugates (**5a** or **5p** derivatives) by reaction of 200 μM TAMM **1a** or alkyne-bearing TAMM **1p** with 50 μM N-terminal cysteine peptides **2x** and **2y**, and proteins zHER2 and SUMO, in 0.1 M NaHCO_3(aq)_ at 37 °C in the presence of 500 μM TCEP and 500 μM Ac-Cys-OMe for 2 h. (b) One-pot sequential diversification of **5px** or **5py** by thiol conjugation with ethyl maleimide, followed by CuAAC with 2-azidoethanol in the presence of BTAA, CuSO_4_, and ascorbic acid, to afford dual-functionalized products. All reagents were added directly to the reaction mixtures containing **5px** or **5py** prepared as described in panel (a). The final concentration of 2-azidoethanol was 250 μM, whereas all other reagents were used at 1 mM. Percentages denote relative abundances determined by ESI-MS and are used to estimate conversions.

### Phage Display Screening of Bicyclic Peptide Binders Based on *o*-TAMM Modification

The thiol retained in enamine **5** can also engage an internal cysteine to form a disulfide bond, yielding side-chain-cyclized peptides. In parallel, TAMM reagents can be equipped with thiol-reactive handles to enable thioether-based cyclization.^20,29,30^ Combining these concepts, we designed *o*-TAMM **1q**, featuring an *ortho*-methyl group to stabilize enamine **5** and a *para*-substituted alkyl bromide as a thiol-reactive electrophile. We envisaged that the reaction of **1q** and CX_m_CX_n_C sequences would yield bicyclic scaffolds containing both a disulfide ring and a thioether ring (Figure 4a). A key requirement is chemoselectivity: the alkyl bromide must react with an internal cysteine rather than the N-terminal thiol generated upon enamine formation.

**Figure 4:**
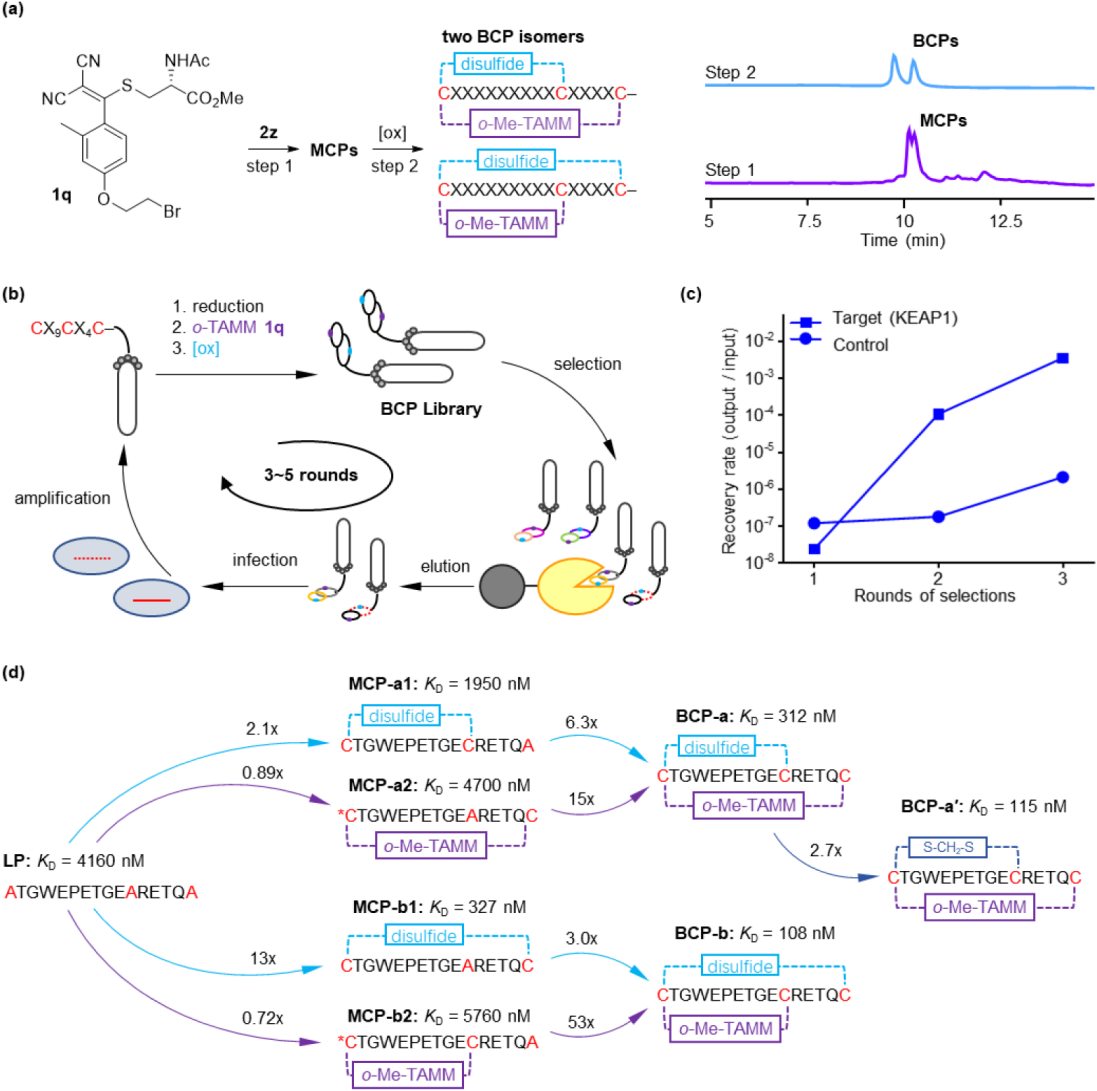
TAMM-mediated peptide cyclization and phage display discovery of bicyclic peptide binders. (a) Construction of bicyclic peptides from *o*-TAMM cross-linker **1q** and peptide **2z** (H–CGGRGGRGGSCGGRGCGW–NH_2_). Initial thioether cyclization affords two monocyclic peptide (MCP) regioisomers, which are resolved by chromatography; subsequent oxidation yields the corresponding bicyclic peptide (BCP) isomers. (b) Scheme for phage display of a CX_9_CX_4_C sequence at the N-terminus of g3p proteins for screening cyclic peptide binders against KEAP1. (c) Recovery efficiency (output/input phage ratio) of phages after iterative selection against target protein KEAP1 (squares) and negative control (circles). Concurrent increases in recovery amount and efficiency reflect the enrichment of specific binders. (d) KEAP1 binding affinities of A3–4 bicyclic isoforms and differently configured control peptides measured by SPR. *C in **MCP-a2**/**b2** denotes the cysteine side chain modified with methyl iodide to form an S-methyl thioether. Representative sensorgrams are shown in Figure S34.

We therefore tested **1q** with peptides containing an N-terminal cysteine and 0, 1, or 2 internal cysteines (i.e., **2x**, **2y**, or **2z**). With **2x**, **1q** produced the expected enamine **5qx** (Figure S28). With **2y**, we observed the formation of a cyclized adduct consistent with intramolecular substitution and HBr loss to form a thioether linkage (Figure S29). These results indicate that the alkyl bromide in **1q** preferentially undergoes substitution with an internal cysteine while sparing the N-terminal thiol. The reaction of **1q** with **2z** afforded two peaks of identical mass (Figure S30), assigned to alternative monocyclic peptide (**MCP**) products formed by thioether cyclization through either Cys10 or Cys15 (Figure 4a). Subsequent oxidation afforded two well-resolved bicyclic peptide (**BCP**) isomers by HPLC.

With the reactivity and selectivity established, we applied **1q** to construct a phage-displayed bicyclic peptide library and selected binders to Kelch-like ECH-associated protein 1 (KEAP1, Figure 4b). KEAP1 is a cytoplasmic adaptor protein and the central regulator of the NRF2-ARE pathway, which protects cells from oxidative stress.^31–33^ Under basal conditions, KEAP1 promotes the ubiquitination and degradation of the transcription factor NRF2; upon oxidative or electrophilic stress, this process is attenuated, allowing NRF2 to accumulate and activate antioxidant-response genes. Dysregulation of the KEAP1-NRF2 axis has been implicated in neurodegenerative diseases, cancer, and inflammation, motivating efforts to develop noncovalent inhibitors that disrupt the KEAP1-NRF2 protein–protein interaction. Moreover, KEAP1 offers a well-defined peptide-binding pocket and a structurally characterized interaction interface, making it an ideal benchmark target for evaluating constrained peptide architectures and selection chemistries.^34–38^

The phages showed progressive enrichment over three rounds, with enrichment values of ∼0.2-, ∼590-, and ∼1600-fold, respectively (Figures 4c, S31 and S32). Next-generation sequencing identified conserved, recurring sequences among the top hits (Figure S33). We selected sequence A3–4 (CTGWEPETGECRETQC), the most abundant after rounds 1 and 2, for further characterization. Two bicyclic isomers, **BCP-a** and **BCP-b**, were synthesized and showed dissociation constants (*K*_D_) of 312 nM and 108 nM for KEAP1 by surface plasmon resonance (SPR) (Figures S34). In contrast, the linear analogue (**LP**), in which all cysteines were mutated to alanines, displayed negligible affinity (*K*_D_ = 4160 nM, Figure S34). To provide more relevant baselines, we also prepared monocyclic controls containing either only the *o*-TAMM-derived thioether ring (**MCP-a1** and **MCP-b1**) or only the disulfide ring (**MCP-a2** and **MCP-b2**). The disulfide-only monocyclic peptides **MCP-a2** and **MCP-b2** showed weak binding (*K*_D_ = 4700 and 5760 nM, respectively), comparable to that of **LP**. The thioether-only monocycle **MCP-a1** also bound weakly (*K*_D_ = 1950 nM), whereas **MCP-b1** retained appreciable affinity (*K*_D_ = 327 nM), although it remained weaker than the corresponding bicyclic construct **BCP-b**. These data indicate that the contribution of each ring is sequence- and isomer-dependent, but overall, the bicyclic architecture provides the strongest binding and most consistent affinity enhancement.

To further assess whether bicyclization occurred on the phage surface, we amplified monoclonal phages harboring the A3–4 sequence and compared the KEAP1-binding enrichment of phages displaying the unmodified linear sequence with that of phages subjected to *o*-TAMM-mediated bicyclization. Because the bicyclic A3–4 peptide binds KEAP1 more strongly than the corresponding linear peptide, successful on-phage bicyclization would be expected to increase enrichment. Consistent with this expectation, the modified phage showed higher KEAP1-binding enrichment than the unmodified linear control (Figure S35), providing functional evidence that bicyclization can occur on the phage surface. Although this experiment does not allow quantitative determination of bicyclization efficiency, it supports the formation of the higher-affinity bicyclic species under phage-display conditions.

We next examined the stability of the disulfide ring in the bicyclic and disulfide-containing monocyclic scaffolds. In the presence of excess reduced glutathione (GSH), the disulfide bond in the corresponding monocyclic peptides was more susceptible to reduction than that in the bicyclic peptides (Figure S36), indicating that the *o*-TAMM-derived thioether ring enhances disulfide stability within the compact bicyclic architecture. Moreover, the disulfide in **BCP-a** could be converted to a redox-stable thioacetal (**BCP-a′**) through reaction with methylene iodide without a loss of KEAP1 affinity. Together, these results show that *o*-TAMM-mediated bicyclization can generate compact peptide ligands with enhanced affinity, improved disulfide stability, and tunable redox properties.

## Discussion

The *o*-TAMM platform offers distinct features that motivate its use relative to other 1,2-aminothiol chemistries, which can, in principle, furnish thiol-containing products. These alternatives include motif-directed CBT condensation,^25^ diphenylcyclopropenone (DPCP) ligation,^39^ and NCL.^18^ However, the practical utility of each approach for modifying surface-displayed proteins (e.g., on phages) or proteins in complex biological settings is limited by different inherent constraints.

CBT-based thiol retention can be achieved by slowing the conversion of a thiol-containing intermediate to the dihydrothiazole product. For example, an N-terminal Cys-Ile-Ser motif was reported to extend the lifetime of this intermediate and enable sequential dual modification through timed addition of a thiol-reactive reagent.^25^ However, the thiol-containing species remains transient and ultimately converts to the inert dihydrothiazole. Product distributions, therefore, depend strongly on the reaction timing. Early addition favors thiol trapping but can suppress CBT ligation, whereas late addition yields predominantly the dihydrothiazole. This operational sensitivity parallels our observations with *p*-TAMMs, where thiol-containing enamines can be captured but require close kinetic control.^20^ Overall, strategies that rely on trapping transient intermediates can be powerful but less practical for robust, user-independent workflows.

DPCP ligation affords a thermodynamically stable thiol-containing product.^39^ Nevertheless, its broader application is limited by selectivity, practicality, and potential bioactivity. DPCP can react with 1,2-aminoalcohols,^39^ and although this occurs more slowly, off-target modification may become relevant in complex proteomes or for N-terminal Ser/Thr sequences. In addition, DPCP has limited aqueous solubility and typically requires substantial organic cosolvent,^39^ which reduces compatibility with native protein conditions and phage display. Synthesis and diversification can also be challenging, particularly for preparing asymmetric or monofunctionalized derivatives needed for modular bioconjugation. Finally, DPCP is a known allergen in clinical use, and potential bioactivity should be considered when applying DPCP-derived conjugates in biological settings.^40^

NCL remains a powerful method for constructing native amide bonds and has transformed protein chemical synthesis.^18^ Although NCL can preserve thiol functionality in the product, it typically requires thioester substrates that are laborious to prepare. In addition, NCL often relies on thiol additives, which can be cytotoxic in cellular contexts. The reaction can also proceed with modest kinetics and may require partial denaturing conditions. These factors limit its direct use for surface-displayed proteins and workflows that require simple aqueous handling and rapid conversion.

In contrast, the *o*-TAMM ligation combines operational simplicity with sterically programmable product selectivity. Sterically hindered *o*-TAMMs stabilize the thiol-retaining enamine adduct as an isolable product under mild aqueous conditions. This enables downstream transformations without trapping transient intermediates. The product of the *o*-TAMM ligation is also structurally distinct. In our system, this feature gives rise to three advantageous properties of the resulting thioether-disulfide bicyclic scaffold: permanent structural preorganization, enhanced disulfide stability, and redox tunability. Specifically, *o*-TAMM ligation forms a (dicyanomethylene)­enamine linkage with greater conformational constraint and rigidity than a conventional amide linkage (Figure S21). The resulting thioether ring serves as a permanent, protease- and reduction-resistant constraint, which preorganizes the peptide into a compact bicyclic topology by shaping the spatial relationship and steric environment of the remaining two thiols. This preorganization appears to be functionally important, as it enhances disulfide stability (Figure S36) and likely facilitates formation of the second disulfide ring during bicyclization. One plausible explanation is that, after disulfide reduction, the thioether-constrained scaffold keeps the two liberated thiols in closer proximity, thereby facilitating disulfide reformation and making the overall structure appear more resistant to reduction. By the same reasoning, this conformational preorganization would also be expected to promote disulfide-ring closure during bicyclic peptide formation. At the same time, retention of the disulfide preserves a chemically editable, redox-responsive element that can be tuned without altering the overall topology. Consistent with this design, the disulfide can be converted at a late stage to a redox-stable thioacetal without disrupting the scaffold. More broadly, this asymmetric thioether-disulfide topology accesses a type of bicyclic framework that is not readily achieved with conventional symmetric cross-linking strategies.

Steric hindrance, however, does impose a kinetic penalty. *o*-TAMMs are less reactive than the corresponding *p*-TAMMs; for example, *o*-TAMM **1a** reacts about 20-fold more slowly than *p*-TAMM **1o** (Figure 2f). Thus, the reaction rate of **1a** is only moderate. Under comparable conditions, it is expected to be similar to conventional NCL^41,42^ and slower than either CBT^13^ or DPCP^39^ ligation (Table S2). Nevertheless, as shown in our previous studies,^21^ the use of a more electron-withdrawing thiol substituent, such as trifluoroethanethiol, could substantially accelerate the reaction (Figure S37), thereby mitigating the current kinetic limitation of *o*-TAMMs. Together, these findings show that *o*-TAMM chemistry offers a tunable balance between reactivity and product stability while also addressing the structural limitations of existing thiol-retaining 1,2-aminothiol ligations. This platform, therefore, enables robust dual modification and compact bicyclic peptide construction in settings relevant to phage and complex biological environments.

An additional opportunity lies in cellular engineering. We recently showed that cyclic peptide binders can improve the safety profile of chimeric antigen receptor (CAR) T cells relative to conventional antibody-derived binders.^43,44^ In this context, because TAMM condensation is compatible with *in vivo* labeling,^21^ this chemistry may offer a route to *o*-TAMM-mediated turn-on CAR systems, in which assembly of the recognition module is temporally controlled. Such an approach could be particularly attractive in nononcology settings, where tighter regulation of CAR activity may be required to balance efficacy and safety.

In conclusion, this work introduces a steric-control strategy that shifts the chemistry of TAMM and 1,2-aminothiol from thiol-consuming cyclization to thiol-retaining ligation. The key advance is a reagent-level design that stabilizes a normally transient thiol-containing intermediate. Computational analysis indicates that steric congestion suppresses the reversal of step *c*, making the thiol-retaining adduct persistent and usable for subsequent transformations. This platform offers at least two complementary capabilities. First, it enables dual modification of N-terminal cysteine peptides and proteins by combining the retained thiol with functionality carried by the TAMM reagent or an added orthogonal handle. Second, it enables compact bicyclic peptide construction through a chemoselective reaction of internal cysteines using an electrophile-equipped *o*-TAMM. By linking mechanistic understanding to reagent design and functional applications, this chemistry expands the synthetic scope of N-terminal cysteine modification and provides a practical route to structurally distinctive bicyclic peptide ligands.

## Supplementary Material



## Notes

Supplementary Figures S1–S37; Supplementary Tables S1–S2; experimental procedures for chemical syntheses of **1a**–**1r**, including ^1^H and ^13^C NMR spectra of the synthesized compounds; and computational analyses of reaction barriers; and experimental procedures for phage-display screening (PDF)
