# Supplementary material for: Thiol-Retaining N‑Terminal Cysteine Chemistry for Dual Modification and Bicyclic Peptide Construction
Source: J Am Chem Soc. 2026 Apr 20;148(17):18020–9. doi: 10.1021/jacs.6c01648 (PMC13154194; doi:10.1021/jacs.6c01648)
Supplement: Supplementary file 1 [file ja6c01648_si_001.pdf]

Supporting Information

Thiol-Retaining N-Terminal Cysteine Chemistry for Dual Modification and Bicyclic Peptide Construction

Junjie Liu,[1,*] Shixiang Duan,[1] Yang Huang,[2] Ming-Yi Jian,[3] Hsuan Suan Lee,[1] Gaocan Dai,[1] Chuanliu Wu,[4,*] Yi-Lin Wu,[3,5,*] Yu-Hsuan Tsai[1,*]

[1] Institute of Molecular Physiology, Shenzhen Bay Laboratory, Shenzhen 518132, China

[2] Guizhou Provincial Key Laboratory of Innovation and Manufacturing for Pharmaceuticals, School of Pharmacy, Zunyi Medical University, Zunyi 563003, China

[3] Department of Applied Chemistry, National Yang Ming Chiao Tung University, Hsinchu 30010, Taiwan

[4] Department of Chemistry, College of Chemistry and Chemical Engineering, The MOE Key Laboratory of Spectrochemical Analysis and Instrumentation, State Key Laboratory of Physical Chemistry of Solid Surfaces, Xiamen University, Xiamen 361005, China

[5] Center for Emergent Functional Matter Science, National Yang Ming Chiao Tung University, Hsinchu 30010, Taiwan

* Correspondence: liujunjie@szbl.ac.cn; chlwu@xmu.edu.cn; yilin.wu@nycu.edu.tw; tsai.y-h@outlook.com

Table of Contents

Chemical Synthesis 4
General procedure for TAMM synthesis 4
Synthesis of TAMM 1a 5
Synthesis of TAMM 1b 8
Synthesis of TAMM 1c 12
Synthesis of TAMM 1d 15
Synthesis of TAMM 1e 18
Synthesis of TAMM 1f 21
Synthesis of TAMM 1g 24
Synthesis of TAMM 1h 27
Synthesis of TAMM 1i 31
Synthesis of TAMM 1j 34
Synthesis of TAMM 1k 37
Synthesis of TAMM 1l 40
Synthesis of TAMM 1q 42
Synthesis of TAMM 1p 48
Synthesis of TAMM 1m 54
Synthesis of TAMM 1n 56
Synthesis of TAMM 1o 59
Synthesis of TAMM 1r 62
Synthesis of TAMM t1 64
Synthesis of TAMM t2 66
Synthesis of TAMM t3 68
One-pot sequential diversification of 5px 68
One-pot sequential diversification of 5py 68
Reaction of TAMM 1 with 2x 69
Reaction of TAMM 1a/1p with NCys protein 69
HPLC characterization of peptides 69
Mechanistic investigation of o-TAMM properties 70
Effective reaction barrier 70
The reaction system under consideration 70
Derivation of the solution under general conditions 70
(Re-)evaluating the solution under the rapid pre-equilibrium conditions 72
Effective reaction barrier under the rapid pre-equilibrium conditions 73
Conformation scan and Cartesian coordinates of optimized geometries 74
Phage Display Screening 85
Modification of phage surface proteins 85
Binder selection by phage display 85
Preparation of next-generation sequencing (NGS) samples 86
Surface plasmon resonance (SPR) assay 87
Supplementary Figures 88
Figure S1. Different reactivity of unsubstituted and ortho-substituted TAMMs 88
Figure S2. Effect of media compositions and pH values for reactions with 1a 89
Figure S3. Reaction of 1a with peptides SNHKRW, TQCDEW, and 2x 90
Figure S4. Reaction of 5ax with iodoacetamide 91
Figure S5. Reaction of 1a and 2x and stability of 5ax in the reaction system 92
Figure S6. Time-dependent stability of 1a, 2x, and 5ax under different pH conditions 94
Figure S7. Reaction of 1b and 2x 95
Figure S8. Reaction of 1c and 2x 96
Figure S9. Reaction of 1d and 2x 97
Figure S10. Reaction of 1e and 2x 98
Figure S11. Reaction of 1f and 2x 99
Figure S12. Reaction of 1g and 2x 100
Figure S13. Reaction of 1h and 2x 101
Figure S14. Reaction of 1i and 2x 102

Figure S15. Reaction of 1j and 2x 103
Figure S16. Reaction of 1k and 2x 104
Figure S17. ^{1}H NMR of 1a and 1e at different temperatures 105
Figure S18. ^{1}H NMR of 1d and 1l at different temperatures 106
Figure S19. ^{1}H NMR of 1h at different temperatures 107
Figure S20. ^{1}H NMR of 1i at different temperatures 108
Figure S21. ^{1}H NMR of t1, t2, t3, t4 at different temperatures 110
Figure S22. Rate constants of different TAMM molecules reacting with 2x........ 112
Figure S23. Lowest energy conformers of 4, 5, and TS2 by DFT calculation 113
Figure S24. Computed free energy profiles for deprotonated anionic 5 and 4 113
Figure S25. Transformation of 5ax to 6ax 114
Figure S26. HPLC chromatograms for the dual functionalization of 2x 114
Figure S27. HPLC chromatograms for the dual functionalization of 2y 115
Figure S28. Reaction between 2x and 1q 115
Figure S29. Reaction between 2y and 1q 116
Figure S30. Mass spectra of bicyclic peptide construction 116
Figure S31. Enrichment from KEAP1 selection 117
Figure S32. Recovered phages after iterative selection against KEAP1 117
Figure S33. HTS results of phages enriched after three rounds of KEAP1 selection 117
Figure S34. SPR results of different peptide variants binding to KEAP1 118
Figure S35. Validation of A3-4 bicyclization on phage surface 119
Figure S36. Ring opening of BCPs and MCPs 120
Figure S37. Reaction of 1r and 2x 121
Supplementary Tables 122
Table S1. Conversions of 1a – 1k reaction with 2x 122
Table S2. Comparison of rate constants 123
Reference 124

Chemical Synthesis

General procedure for TAMM synthesis

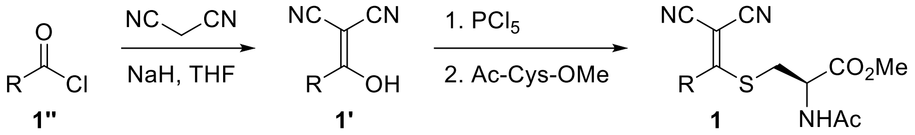

To a suspension of NaH (288 mg, 12 mmol, 6.0 eq) in anhydrous THF (10 mL) under a nitrogen atmosphere at 0 °C was added a solution of malononitrile (397 mg, 6 mmol, 3.0 eq) in anhydrous THF (10 mL) in a dropwise manner. After 1 hour at 0 °C, a solution of **1″** (2 mmol, 1.0 eq) in anhydrous THF (10 mL) was added to the mixture in a dropwise manner. After the addition, the reaction was allowed to warm up to room temperature. After 2 hours at room temperature, the solvent was removed under reduced pressure. The mixture was acidified to pH 1–2 using $HCl_{(aq)}$. The mixture was extracted with EtOAc (3 × 100 mL). The combined organic layers were washed with brine (3 × 50 mL), dried over Na_2SO_4, filtered and concentrated. Silica gel column chromatography was performed to isolate **1′**.

To a solution of **1′** (1 mmol, 1.0 eq) in anhydrous acetonitrile (20 mL) under a nitrogen atmosphere was added PCl_5 (624 mg, 3 mmol, 3 eq). After 6 hours at 65 °C, the solvent was removed under reduced pressure. The residue was dissolved in DCM (60 mL), washed with water (3 × 20 mL) and brine (20 mL), dried over Na_2SO_4, filtered and concentrated. The resulting yellow solid was then dissolved in acetonitrile (15 mL), followed by addition of Ac-Cys-OMe (213 mg, 1.2 mmol, 1.2 eq) and NaHCO3 (252 mg, 3 mmol, 3.0 eq). After stirring overnight at room temperature, the solvent was removed under reduced pressure. The mixture was purified by silica gel column chromatography to afford **1**.

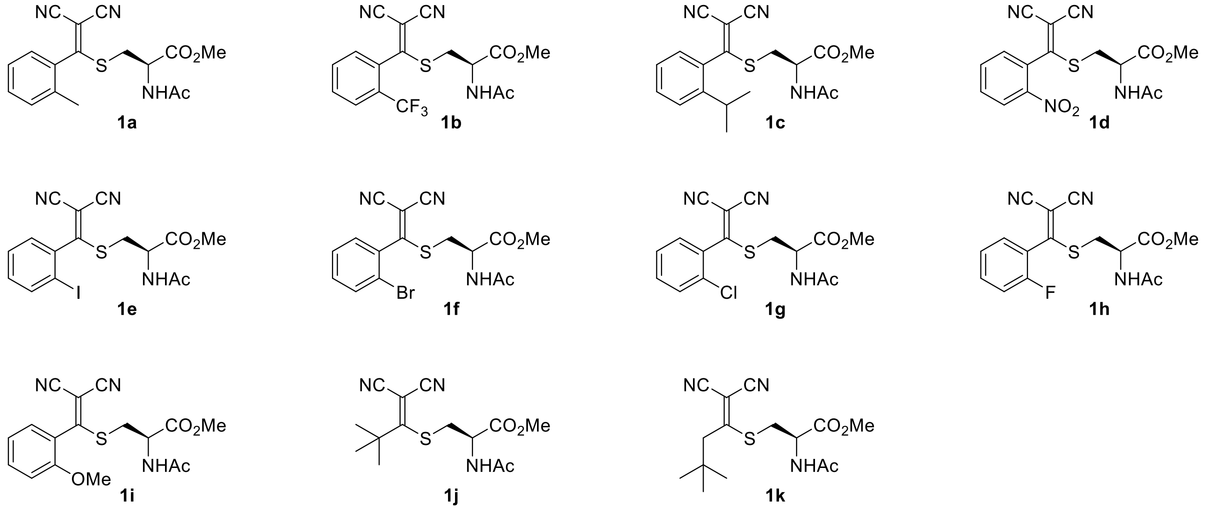

Synthesis of TAMM 1a

Compound **1a'** (360 mg, 1.96 mmol, 98% yield) was obtained as a light yellow solid from silica gel column chromatography (DCM:MeOH=10:1). ^{1}H NMR (400 MHz, Methanol-d_4) δ 7.482 (td, J = 7.40, 1.60 Hz, 1H), 7.398 – 7.327 (m, 3H), 2.392 (s, 3H). ^{13}C NMR (151 MHz, Methanol-d_4) δ 186.216, 135.572, 132.148, 131.447, 130.723, 127.642, 126.017, 113.633, 111.900, 63.555, 17.755. ESI-(-)-HRMS (M-H)$^-$ calculated for $C_{11}H_8N_2O$: 183.05639; found: 183.05632 (-3.1 ppm). R_f (DCM:MeOH=10:1) = 0.2.

Compound **1a** (133 mg, 0.38 mmol, 38% yield) was obtained as a light yellow solid from silica gel column chromatography (EA:PE=2:1). ^{1}H NMR (400 MHz, Chloroform-d) δ 7.426 – 7.388 (m, 1H), 7.328 (d, J = 7.60 Hz, 2H), 7.082 (dd, J = 22.40, 7.60 Hz, 1H), 6.621 – 6.518 (m, 1H), 4.584 (dtd, J = 11.00, 6.70, 5.60, 2.80 Hz, 1H), 3.682 (dd, J = 6.80, 3.20 Hz, 3H), 3.108 – 2.814 (m, 2H), 2.284 (s, 3H), 1.960 (s, 3H). ^{13}C NMR (151 MHz, Chloroform-d) δ 181.951, 181.771, 170.186, 169.524, 169.480, 134.975, 134.684, 131.714, 131.653, 131.585, 131.560, 131.455, 127.514, 127.379, 127.074, 126.963, 111.850, 111.587, 80.585, 80.496, 53.147, 53.102, 51.149, 51.078, 35.686, 35.660, 22.850, 22.833, 18.944, 18.909. ESI-(+)-HRMS (M+H)$^+$ calculated for $C_{17}H_{17}N_3O_3S$: 344.10634; found: 344.10592 (+0.4 ppm). R_f (EA:PE=3:1) = 0.55.

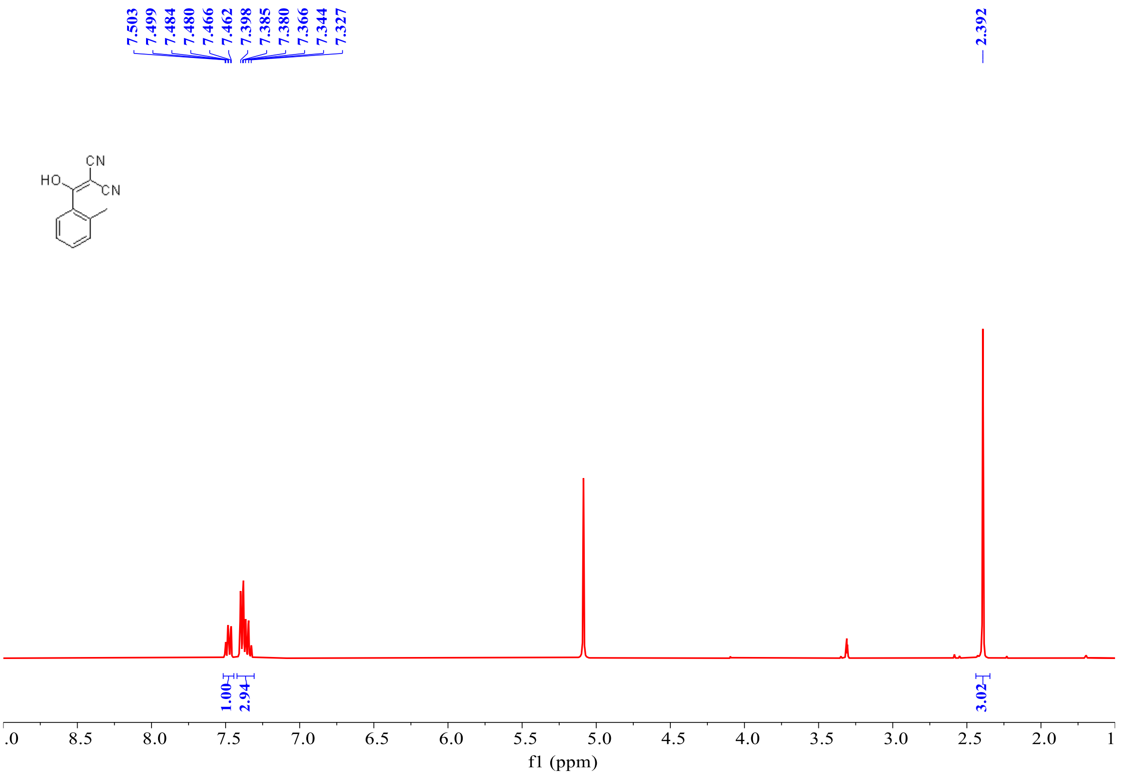

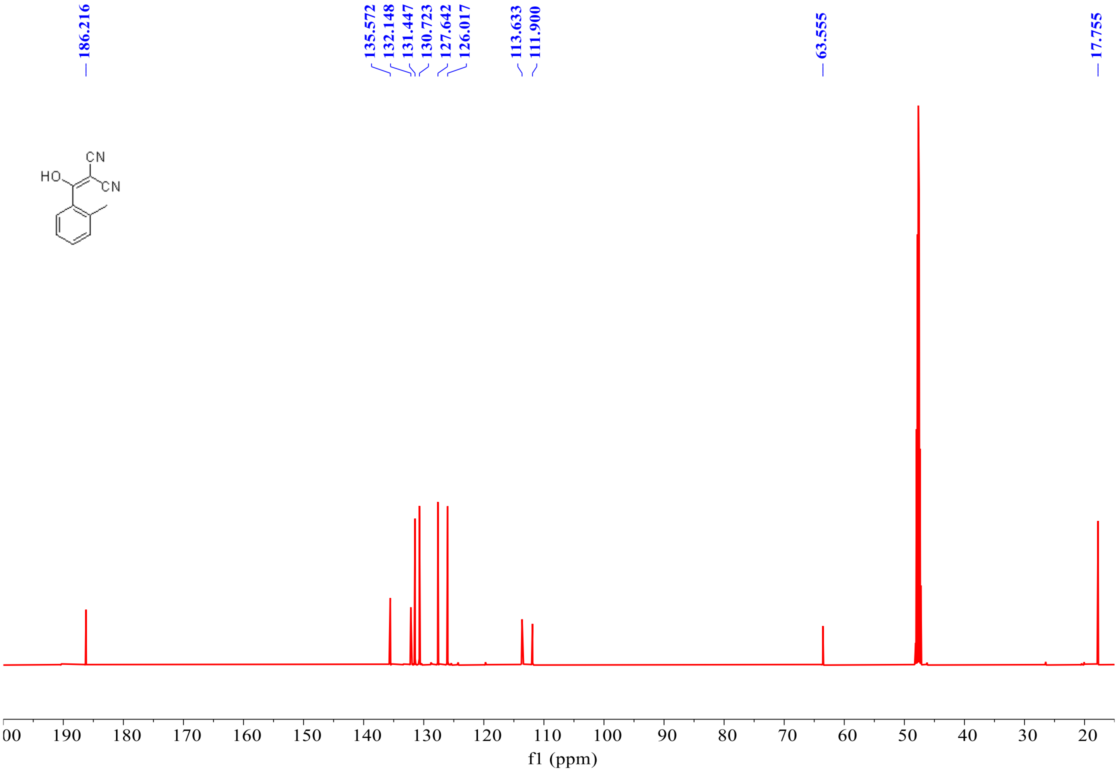
186.216
135.572
132.148
131.447
130.723
127.642
126.017
113.633
111.900
63.555
17.755
CN
HO
CN
f1 (ppm)

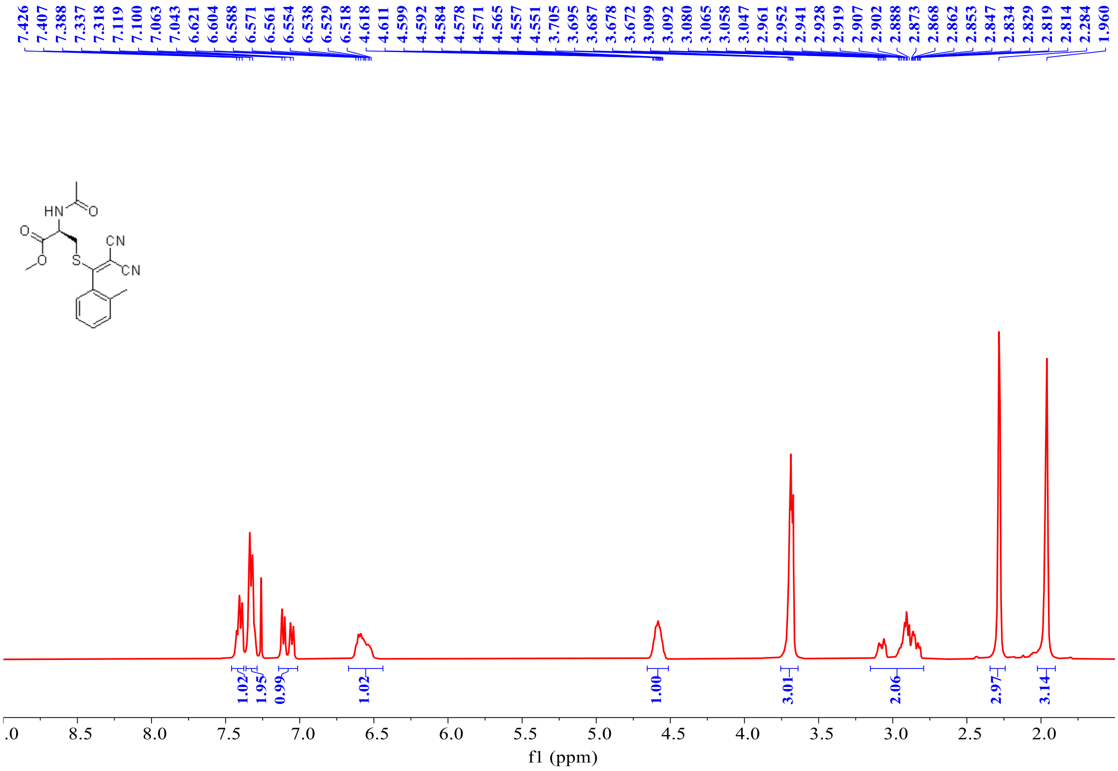
7.426
7.407
7.388
7.337
7.318
7.119
7.100
7.063
7.043
6.621
6.604
6.588
6.571
6.561
6.554
6.538
6.529
6.518
4.618
4.611
4.599
4.592
4.584
4.578
4.571
4.565
4.557
4.551
3.705
3.695
3.687
3.678
3.672
3.099
3.092
3.080
3.065
3.058
3.047
2.961
2.952
2.941
2.928
2.919
2.907
2.902
2.888
2.873
2.868
2.862
2.853
2.847
2.834
2.829
2.819
2.814
2.284
1.960
HN
O
O
O
S
CN
CN
1.02
1.95
0.99
1.02
1.00
3.01
2.06
2.97
3.14
f1 (ppm)

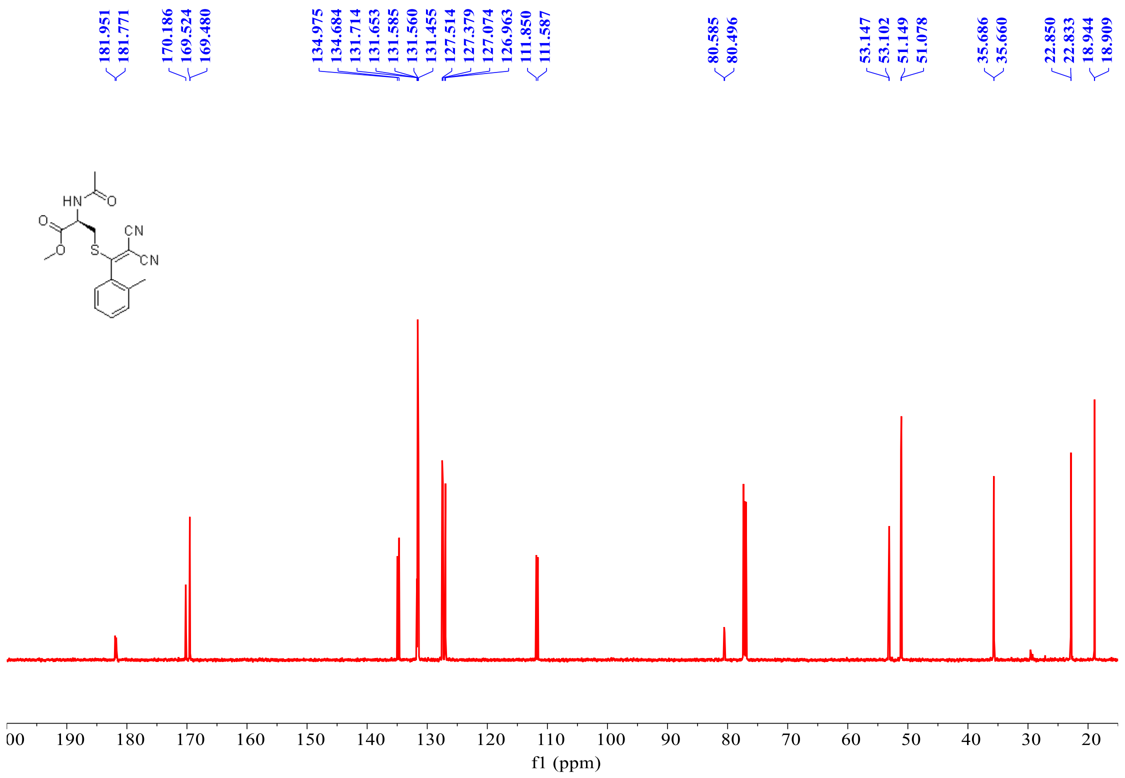
181.951
181.771
170.186
169.524
169.480
134.975
134.684
131.714
131.653
131.585
131.560
131.455
127.514
127.379
127.074
126.963
111.850
111.587
80.585
80.496
53.147
53.102
51.149
51.078
35.686
35.660
22.850
22.833
18.944
18.909
HN
O
O
O
S
CN
CN
00
190
180
170
160
150
140
130
120
110
100
90
80
70
60
50
40
30
20
f1 (ppm)

Synthesis of TAMM 1b

Compound **1b′** (471 mg, 1.98 mmol, 99% yield) was obtained as a light yellow solid from silica gel column chromatography (DCM:MeOH=10:1). ^{1}H NMR (400 MHz, Methanol-*d*$_4$) δ 7.846 – 7.817 (m, 1H), 7.779 – 7.687 (m, 2H), 7.575 (d, *J* = 7.20 Hz, 1H). ^{13}C NMR (151 MHz, Methanol-*d*$_4$) δ 183.897, 132.650, 131.520, 131.107, 129.260, 127.766, 127.555, 127.343, 126.750, 126.718, 126.686, 126.655, 126.118, 124.309, 122.499, 120.689, 113.566, 111.927, 63.762. ^{19}F NMR (376 MHz, Methanol-*d*$_4$) δ -60.637. ESI-(-)-HRMS (M-H)$^-$ calculated for $C_{11}H_5F_3N_2O$: 237.02812; found: 237.02815 (-0.1 ppm). R_f (DCM:MeOH=5:1) = 0.3.

Compound **1b** (92 mg, 0.23 mmol, 23% yield) was obtained as a light yellow solid from silica gel column chromatography (EA:PE=2:1). ^{1}H NMR (400 MHz, Chloroform-*d*) δ 7.870 (dd, *J* = 8.00, 2.80 Hz, 1H), 7.796 – 7.690 (m, 2H), 7.326 – 7.260 (m, 1H), 6.292 (dd, *J* = 10.40, 6.40 Hz, 1H), 4.717 – 4.639 (m, 1H), 3.775 (d, *J* = 20.80 Hz, 3H), 3.073 (td, *J* = 12.80, 4.80 Hz, 1H), 2.906 (ddd, *J* = 16.00, 11.20, 5.60 Hz, 1H), 2.028 (d, *J* = 20.00 Hz, 3H). ^{13}C NMR (151 MHz, Chloroform-*d*) δ 178.142, 178.004, 170.289, 170.066, 169.449, 169.418, 133.391, 133.265, 131.925, 131.891, 130.436, 130.352, 129.274, 129.202, 127.953, 127.921, 127.870, 127.838, 125.786, 123.970, 123.923, 122.154, 122.106, 120.339, 111.397, 111.195, 111.167, 82.460, 82.421, 53.520, 53.507, 51.047, 50.914, 36.325, 36.190, 23.157, 23.021. ^{19}F NMR (376 MHz, Chloroform-*d*) δ -59.366, -59.467. ESI-(+)-HRMS (M+H)$^+$ calculated for $C_{17}H_{14}F_3N_3O_3S$: 398.07807; found: 398.07710 (+2.4 ppm). R_f (EA:PE=3:1) = 0.5.

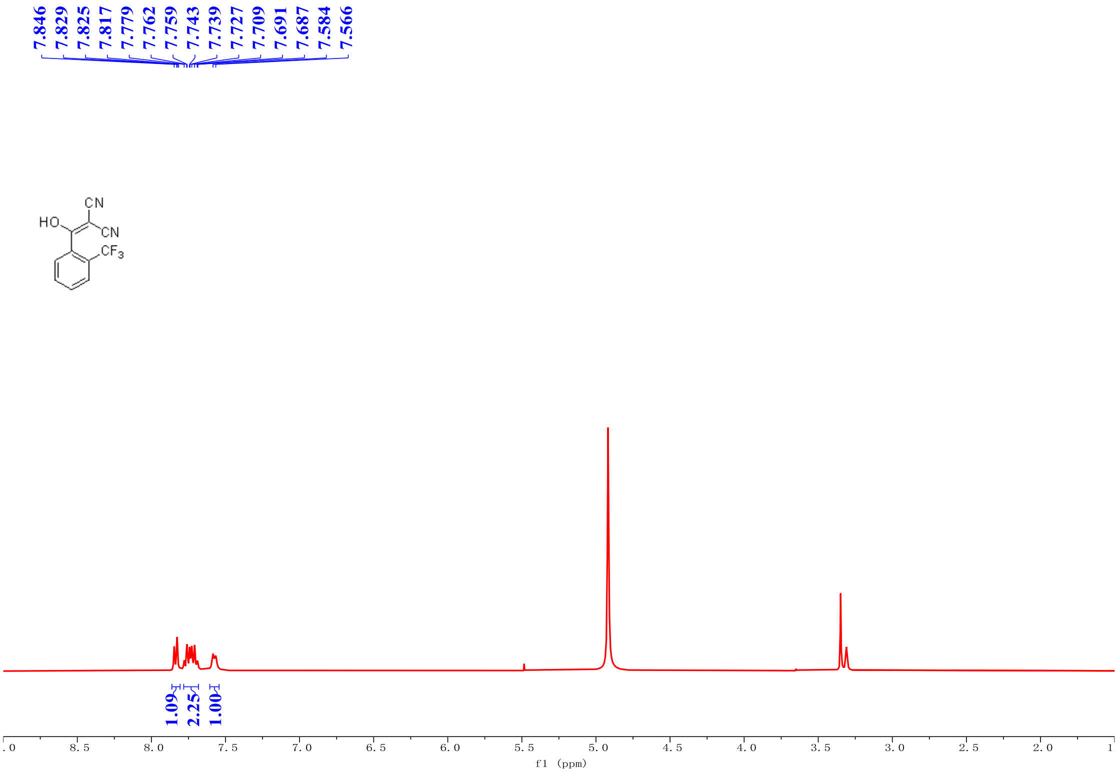

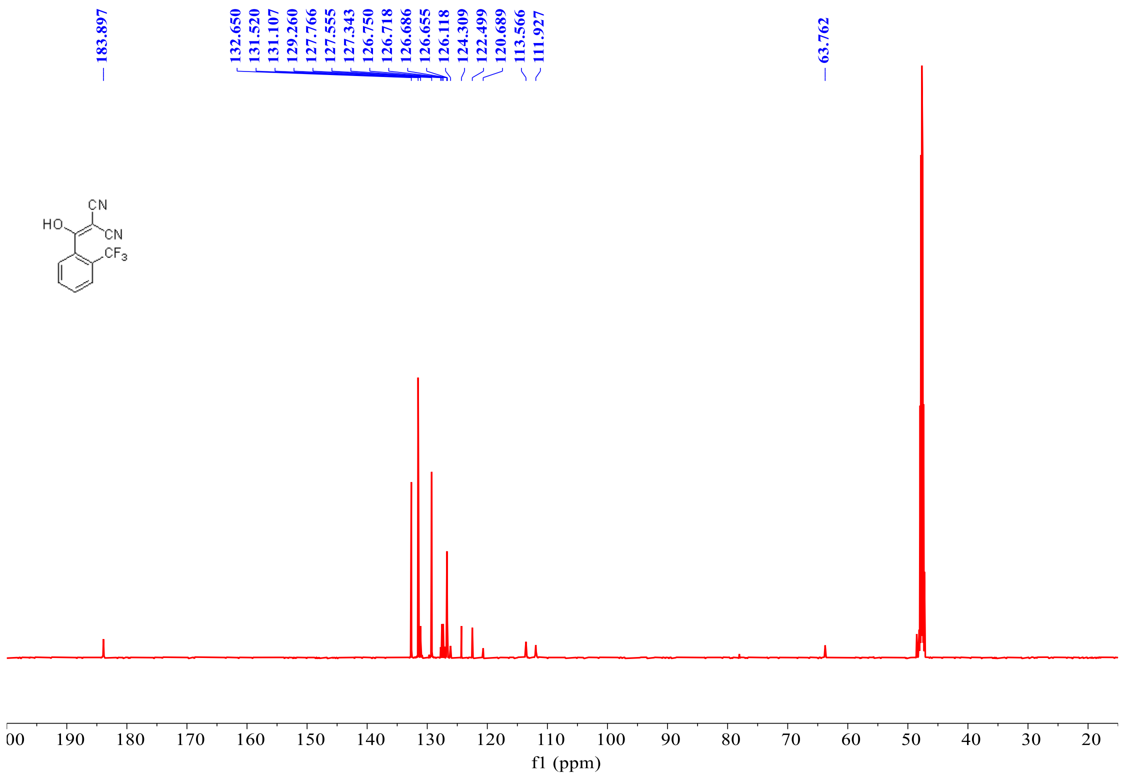
183.897
132.650
131.520
131.107
129.260
127.766
127.555
127.343
126.750
126.718
126.686
126.655
126.118
124.309
122.499
120.689
113.566
111.927
63.762
HO
CN
CN
CF_3
00 190 180 170 160 150 140 130 120 110 100 90 80 70 60 50 40 30 20
f1 (ppm)

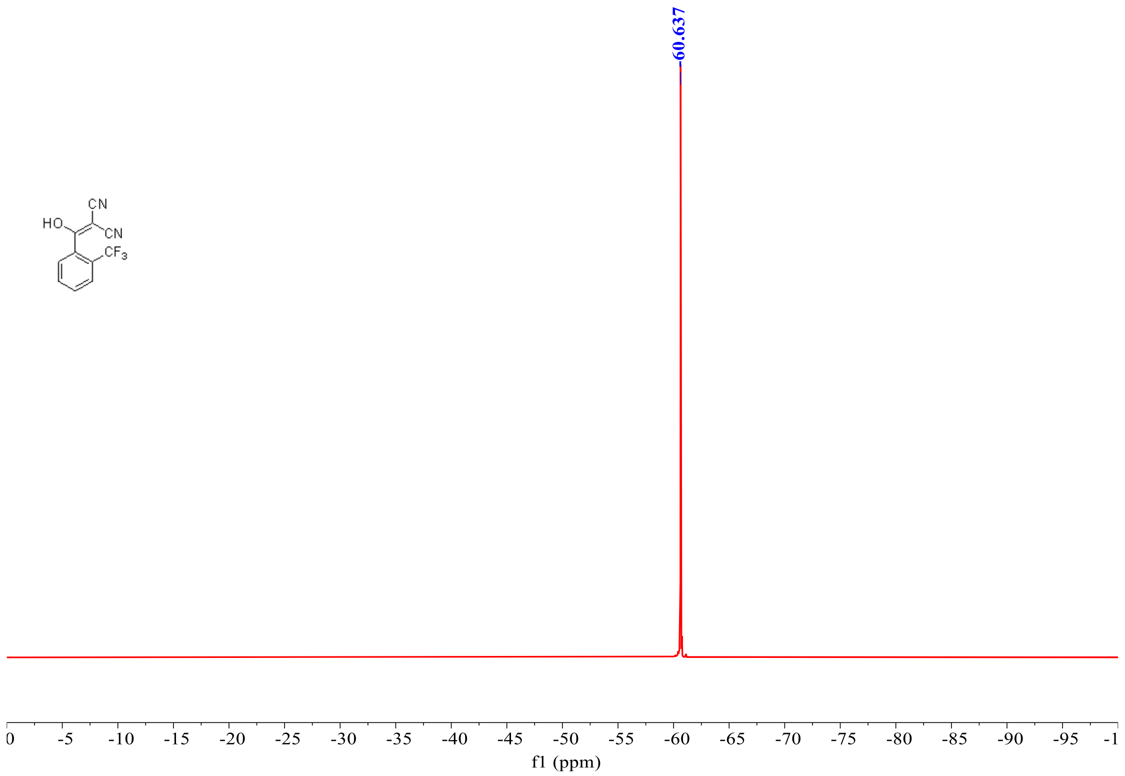
-60.637
HO
CN
CN
CF_3
0 -5 -10 -15 -20 -25 -30 -35 -40 -45 -50 -55 -60 -65 -70 -75 -80 -85 -90 -95 -1
f1 (ppm)

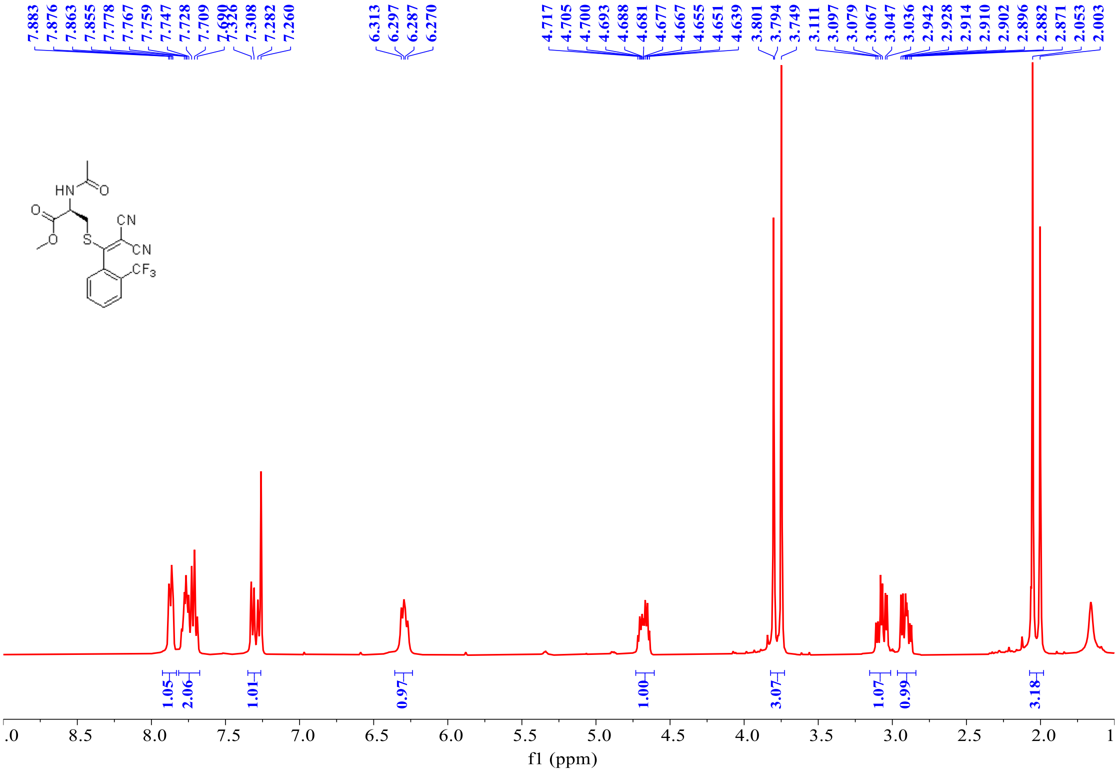
7.883 7.876 7.863 7.855 7.778 7.767 7.759 7.747 7.728 7.709 7.690 7.326 7.308 7.282 7.260
6.313 6.297 6.287 6.270
4.717 4.705 4.700 4.693 4.688 4.681 4.677 4.667 4.655 4.651 4.639
3.801 3.794 3.749
3.111 3.097 3.079 3.067 3.047 3.036 2.942 2.928 2.914 2.910 2.902 2.896 2.882 2.871 2.053 2.003
HN
O
CN
CN
CF3
S
1.05 2.06 1.01 0.97 1.00 3.07 1.07 0.99 3.18
8.5 8.0 7.5 7.0 6.5 6.0 5.5 5.0 4.5 4.0 3.5 3.0 2.5 2.0
f1 (ppm)

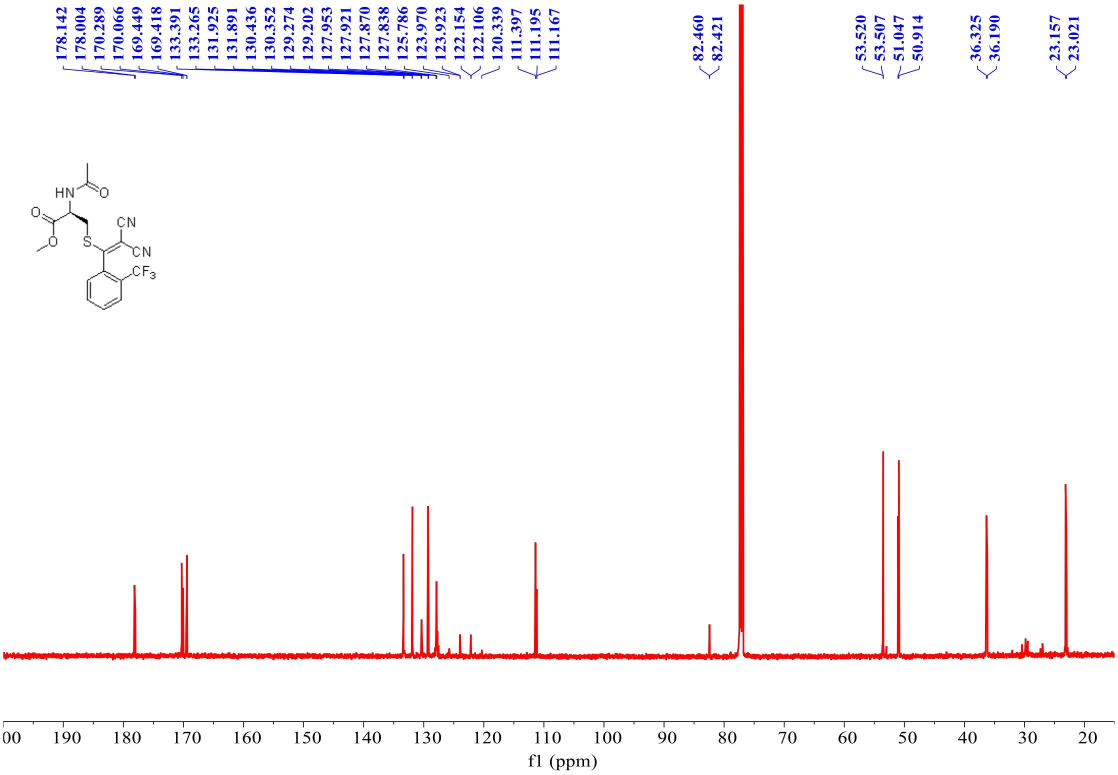
178.142 178.004 170.289 170.066 169.449 169.418 133.391 133.265 131.925 131.891 130.436 130.352 129.274 129.202 127.953 127.921 127.870 127.838 125.786 123.970 123.923 122.154 122.106 120.339 111.397 111.195 111.167
82.460 82.421
53.520 53.507 51.047 50.914
36.325 36.190
23.157 23.021
HN
O
CN
CN
CF3
S
190 180 170 160 150 140 130 120 110 100 90 80 70 60 50 40 30 20
f1 (ppm)

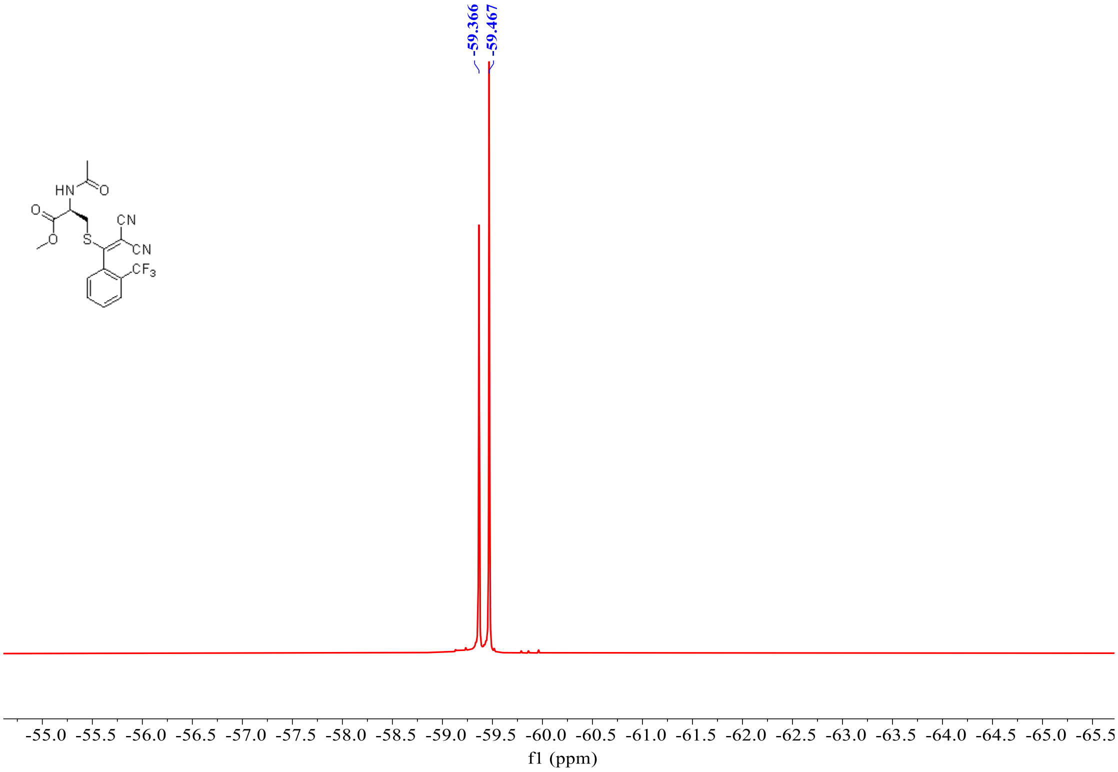

-59.366
-59.467
HN
O
O
O
S
CN
CN
CF3
-55.0 -55.5 -56.0 -56.5 -57.0 -57.5 -58.0 -58.5 -59.0 -59.5 -60.0 -60.5 -61.0 -61.5 -62.0 -62.5 -63.0 -63.5 -64.0 -64.5 -65.0 -65.5
f1 (ppm)

Synthesis of TAMM 1c

Compound **1c′** (201 mg, 1.9 mmol, 95% yield) was obtained as a light yellow solid from silica gel column chromatography (DCM:MeOH=10:1). ^{1}H NMR (400 MHz, Methanol-d_4) δ 7.577 – 7.541 (m, 2H), 7.392 – 7.300 (m, 2H), 3.009 (p, J = 6.83 Hz, 1H), 1.328 (d, J = 6.80 Hz, 6H). ^{13}C NMR (101 MHz, Methanol-d_4) δ 186.724, 146.377, 131.596, 131.404, 131.372, 127.204, 126.144, 126.116, 113.654, 111.751, 63.924, 30.785, 22.919. ESI-(-)-HRMS (M-H)$^-$ calculated for $C_{13}H_{12}N_2O$: 211.08769; found: 211.08787 (+0.8 ppm). R_f (DCM:MeOH=5:1) = 0.4.

Compound **1c** (85 mg, 0.23 mmol, 23% yield) was obtained as a light yellow solid from silica gel column chromatography (EA:PE=2:1). ^{1}H NMR (400 MHz, Chloroform-d) δ 7.518 – 7.450 (m, 2H), 7.353 – 7.301 (m, 1H), 7.016 (ddd, J = 22.40, 8.00, 1.60 Hz, 1H), 6.461 (dd, J = 10.00, 7.60 Hz, 1H), 4.709 – 4.632 (m, 1H), 3.724 (d, J = 16.40 Hz, 3H), 2.944 – 2.929 (m, 1H), 3.128 – 2.803 (m, 2H), 1.990 (d, J = 9.20 Hz, 3H), 1.270 – 1.226 (m, 6H). ^{13}C NMR (101 MHz, Chloroform-d) δ 182.303, 182.088, 170.134, 170.072, 169.528, 169.466, 145.811, 145.634, 132.008, 131.983, 130.688, 130.440, 127.285, 127.155, 127.127, 127.025, 126.988, 111.959, 111.618, 80.969, 80.921, 53.292, 53.221, 50.953, 50.867, 36.165, 36.077, 30.732, 30.679, 24.778, 24.717, 23.421, 23.345, 22.970, 22.919. ESI-(+)-HRMS (M+H)$^+$ calculated for $C_{19}H_{21}N_3O_3S$: 372.13764; found: 372.13724 (+1.1 ppm). R_f (EA:PE=3:1) = 0.45.

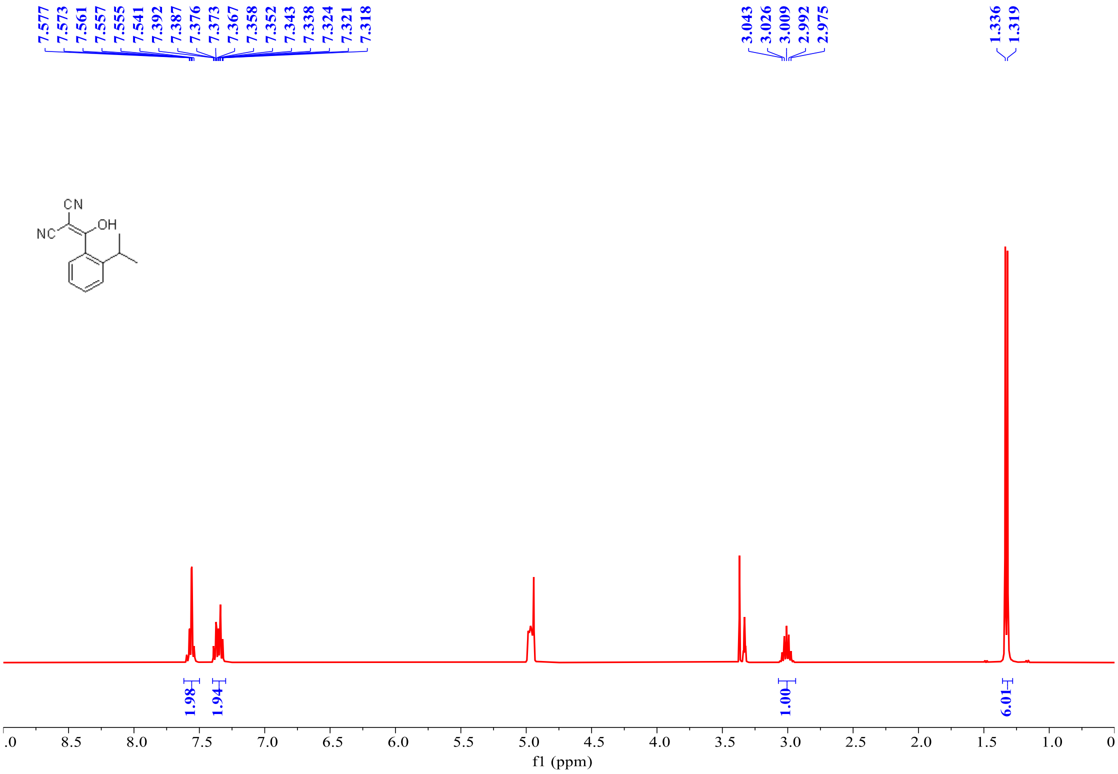

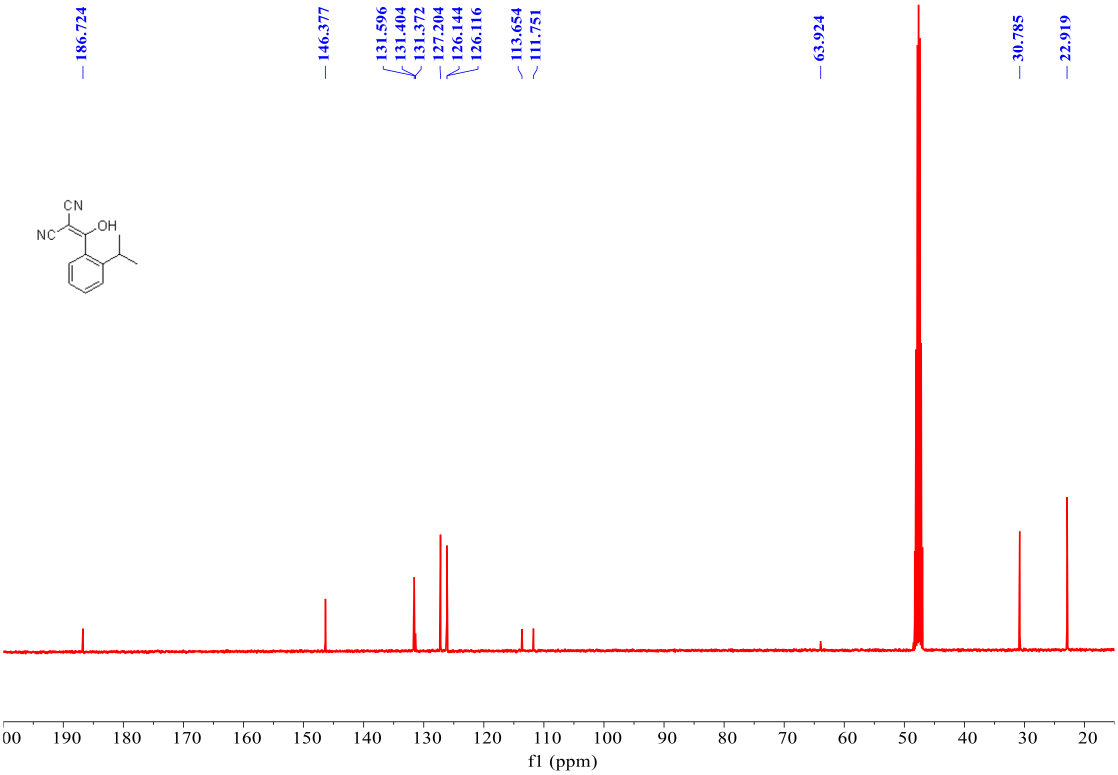
186.724
146.377
131.596
131.404
131.372
127.204
126.144
126.116
113.654
111.751
63.924
30.785
22.919
CN
NC
OH
00 190 180 170 160 150 140 130 120 110 100 90 80 70 60 50 40 30 20
f1 (ppm)

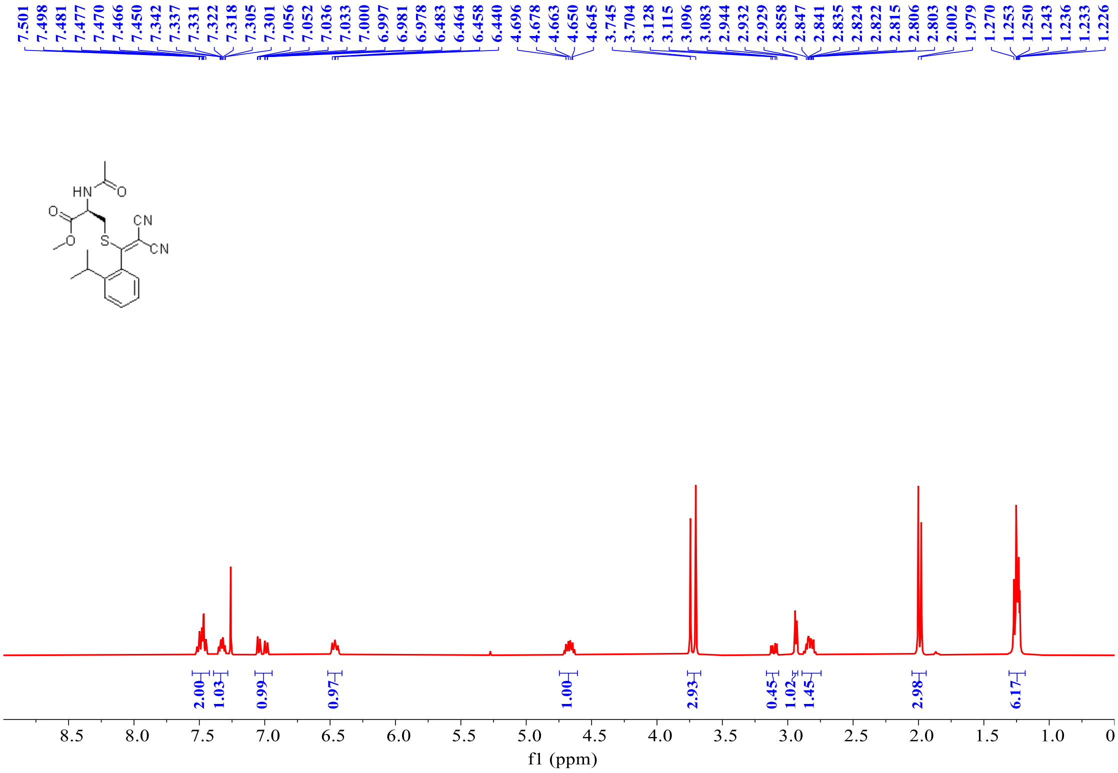
7.501
7.498
7.481
7.477
7.470
7.466
7.450
7.342
7.337
7.331
7.322
7.318
7.305
7.301
7.056
7.052
7.036
7.033
7.000
6.997
6.981
6.978
6.483
6.464
6.458
6.440
4.696
4.678
4.663
4.650
4.645
3.745
3.704
3.128
3.115
3.096
3.083
2.944
2.932
2.929
2.858
2.847
2.841
2.835
2.824
2.822
2.815
2.806
2.803
2.002
1.979
1.270
1.253
1.250
1.243
1.236
1.233
1.226
HN
O
O
O
S
CN
CN
2.00
1.03
0.99
0.97
1.00
2.93
0.45
1.02
1.45
2.98
6.17
8.5 8.0 7.5 7.0 6.5 6.0 5.5 5.0 4.5 4.0 3.5 3.0 2.5 2.0 1.5 1.0 0
f1 (ppm)

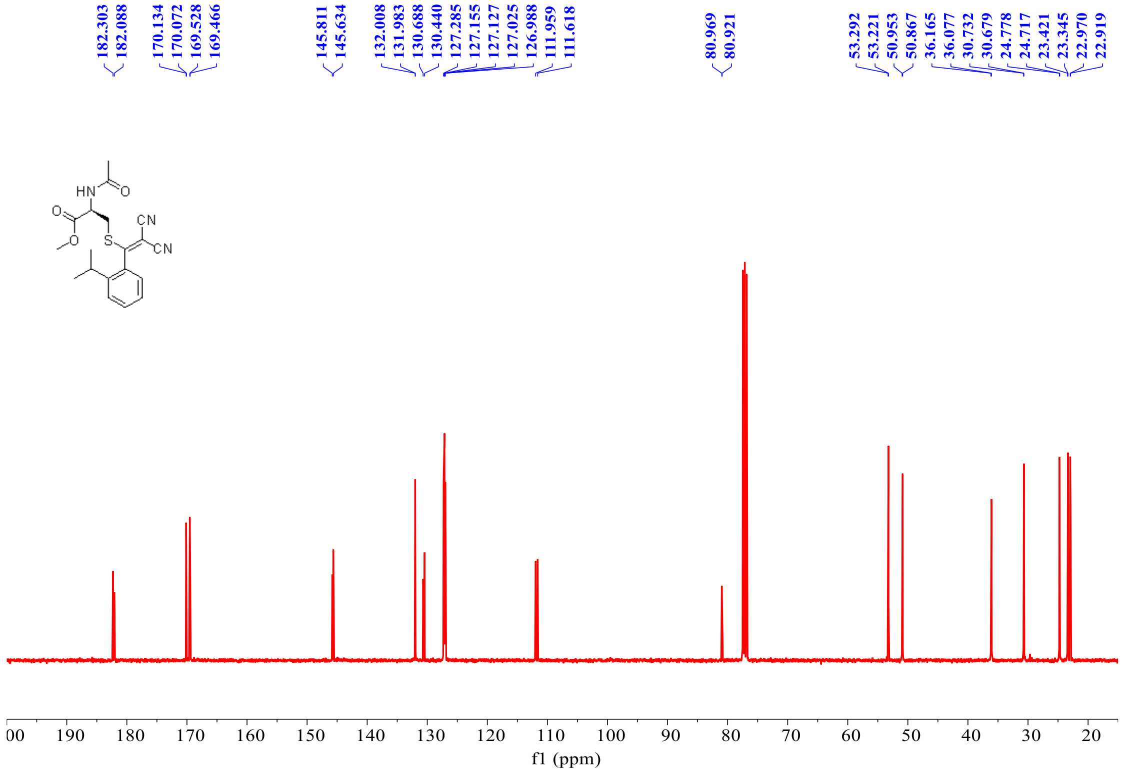
182.303
182.088
170.134
170.072
169.528
169.466
145.811
145.634
132.008
131.983
130.688
130.440
127.285
127.155
127.127
127.025
126.988
111.959
111.618
80.969
80.921
53.292
53.221
50.953
50.867
36.165
36.077
30.732
30.679
24.778
24.717
23.421
23.345
22.970
22.919
HN
O
O
O
S
CN
CN
00
190
180
170
160
150
140
130
120
110
100
90
80
70
60
50
40
30
20
f1 (ppm)

Synthesis of TAMM 1d

Compound **1d'** (408 mg, 1.9 mmol, 95% yield) was obtained as a light yellow solid from silica gel column chromatography (DCM:MeOH=10:1). ^{1}H NMR (400 MHz, Methanol-d_4) δ 8.310 (d, J = 8.40 Hz, 1H), 7.903 (t, J = 6.80 Hz, 1H), 7.821 (t, J = 7.80 Hz, 1H), 7.70 (d, J = 7.20 Hz, 1H). ^{13}C NMR (151 MHz, Methanol-d_4) δ 185.654, 147.393, 135.826, 133.575, 131.412, 130.515, 126.192, 115.863, 114.182, 62.195. ESI-(-)-HRMS (M-H)$^-$ calculated for $C_{10}H_5N_3O_3$: 214.02581; found: 214.02610 (-1.4 ppm). R_f (DCM:MeOH=10:1) = 0.2.

Compound **1d** (269 mg, 0.72 mmol, 72% yield) was obtained as a light yellow solid from silica gel column chromatography (EA:PE=2:1). ^{1}H NMR (400 MHz, Chloroform-d) δ 8.349 (dt, J = 8.40, 1.80 Hz, 1H), 7.901 (tt, J = 7.60, 1.40 Hz, 1H), 7.811 (td, J = 7.80, 1.60 Hz, 1H), 7.415 (ddd, J = 16.80, 7.60, 1.40 Hz, 1H), 6.464 (d, J = 6.80 Hz, 1H), 4.666 – 4.599 (m, 1H), 3.777 (d, J = 14.40 Hz, 3H), 3.209 – 2.994 (m, 2H), 2.020 (d, J = 10.80 Hz, 3H). ^{13}C NMR (151 MHz, Chloroform-d) δ 178.281, 178.192, 170.319, 169.339, 146.256, 135.188, 135.160, 132.953, 132.908, 129.964, 129.892, 127.942, 127.837, 126.154, 126.065, 111.361, 111.118, 111.084, 81.099, 81.080, 53.437, 53.414, 51.232, 51.101, 36.145, 36.088, 22.973, 22.901. ESI-(+)-HRMS (M+H)$^+$ calculated for $C_{16}H_{14}N_4O_5S$: 375.07577; found: 375.07505 (+1.9 ppm). R_f (EA:PE=3:1) = 0.4.

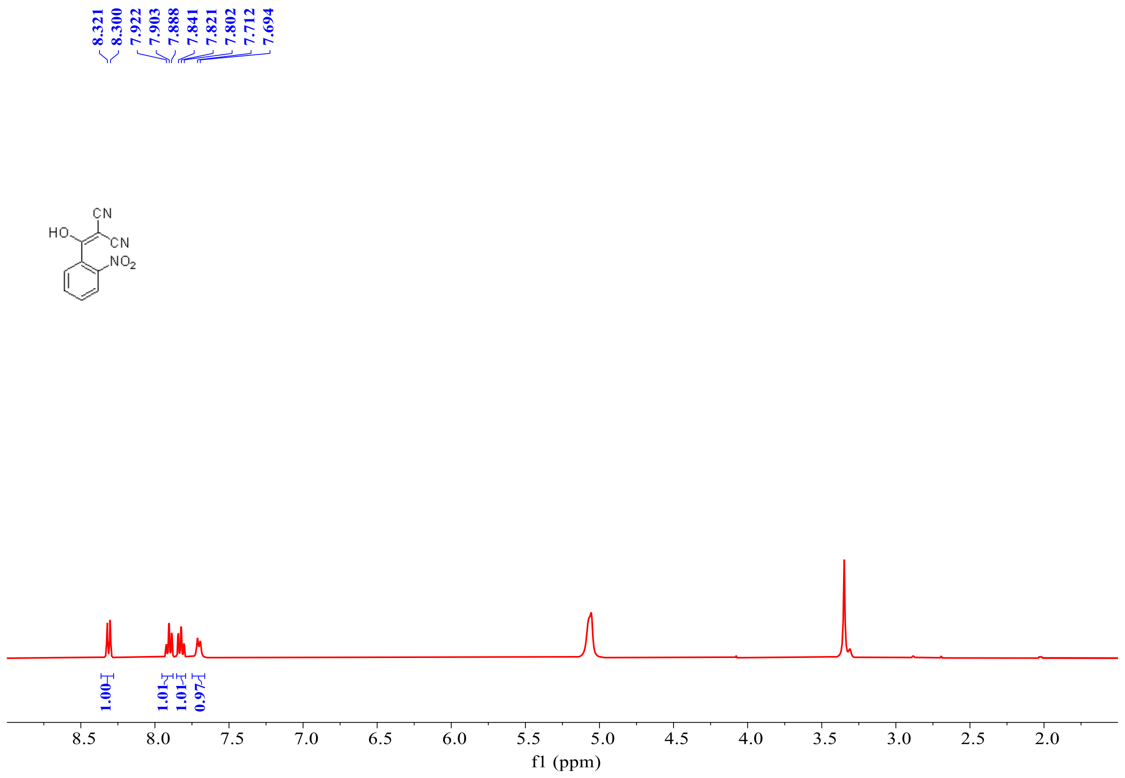

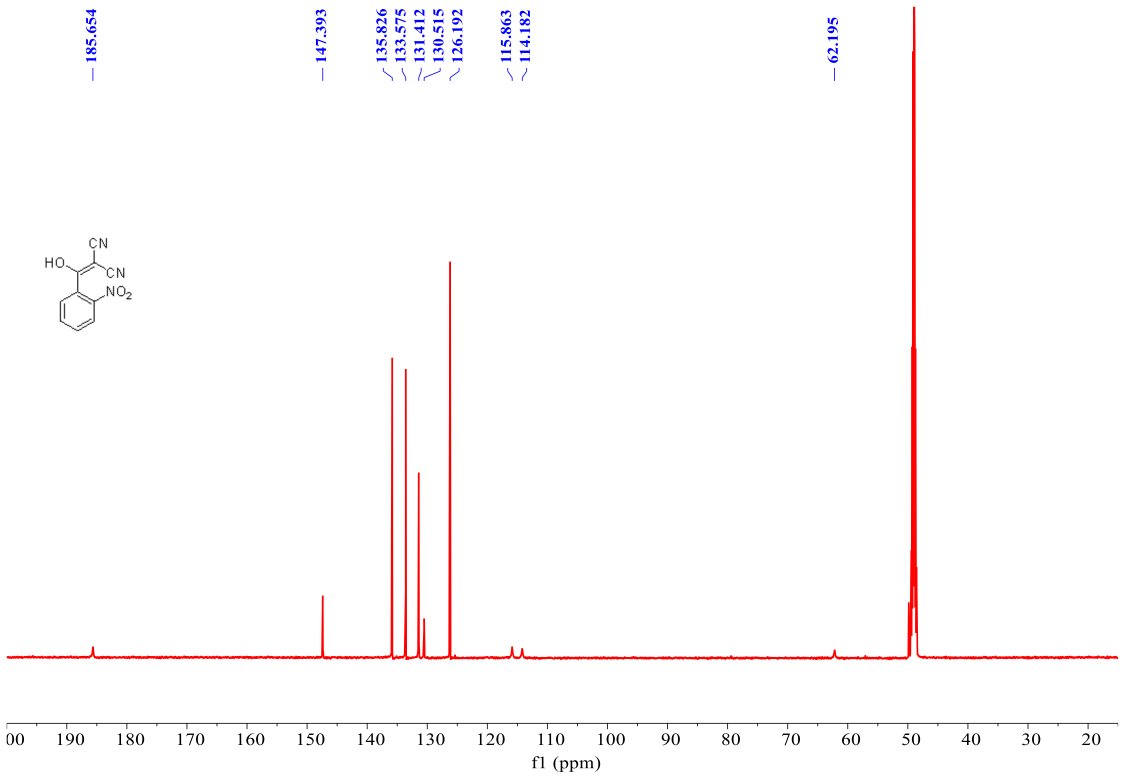
185.654
147.393
135.826
133.575
131.412
130.515
126.192
115.863
114.182
62.195
HO
CN
CN
NO2
f1 (ppm)

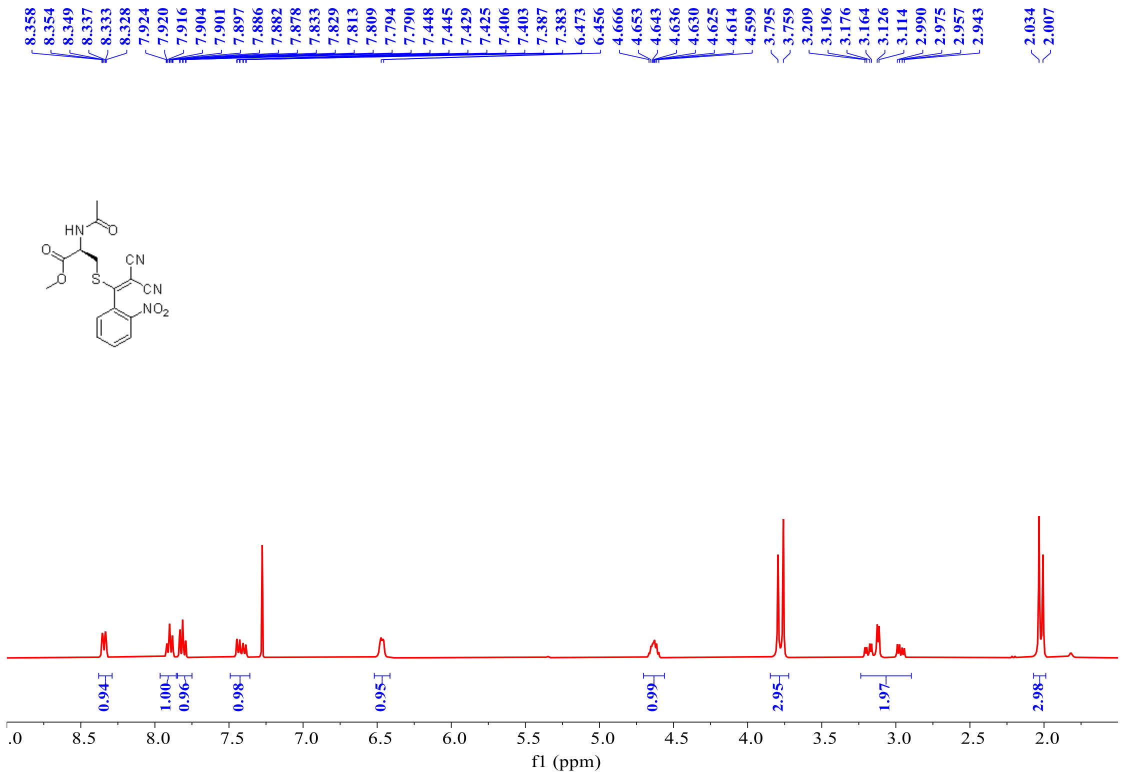
8.358
8.354
8.349
8.337
8.333
8.328
7.924
7.920
7.916
7.904
7.901
7.897
7.886
7.882
7.878
7.833
7.829
7.813
7.809
7.794
7.790
7.448
7.445
7.429
7.425
7.406
7.403
7.387
7.383
6.473
6.456
4.666
4.653
4.643
4.636
4.630
4.625
4.614
4.599
3.795
3.759
3.209
3.196
3.176
3.164
3.126
3.114
2.990
2.975
2.957
2.943
2.034
2.007
HN
O
O
O
S
CN
CN
NO2
0.94
1.00
0.96
0.98
0.95
0.99
2.95
1.97
2.98
f1 (ppm)

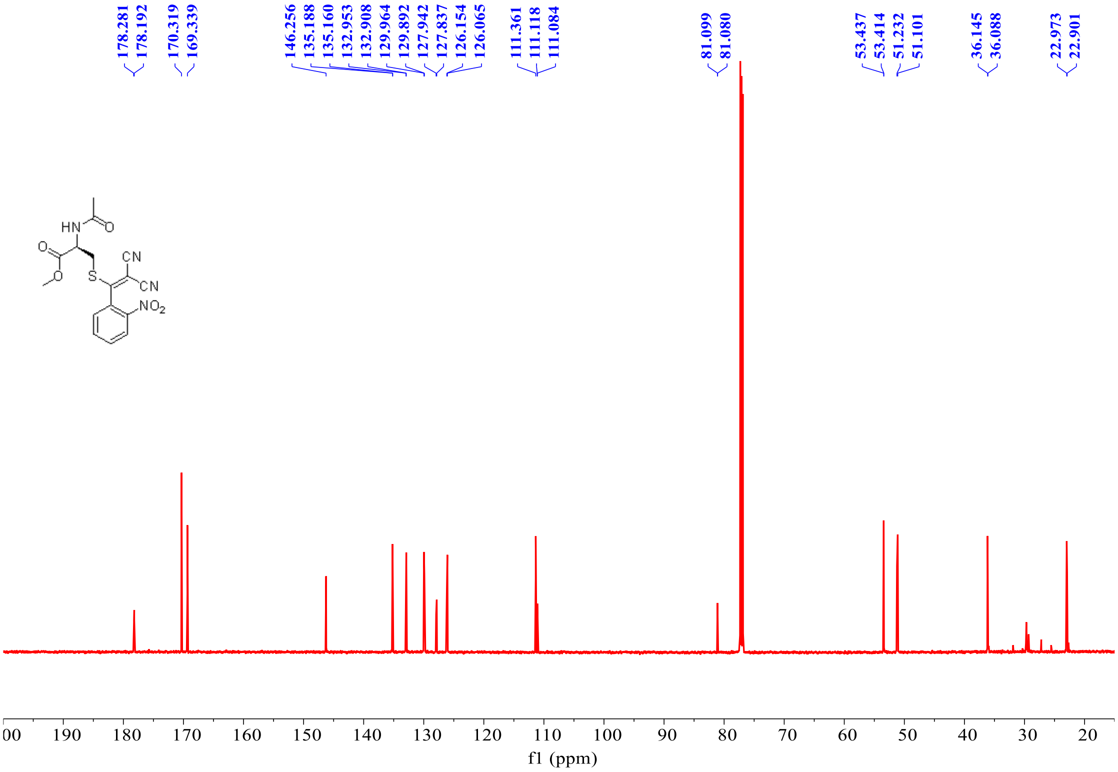
178.281
178.192
170.319
169.339
146.256
135.188
135.160
132.953
132.908
129.964
129.892
127.942
127.837
126.154
126.065
111.361
111.118
111.084
81.099
81.080
53.437
53.414
51.232
51.101
36.145
36.088
22.973
22.901
HN
O
O
O
S
CN
CN
NO2
00
190
180
170
160
150
140
130
120
110
100
90
80
70
60
50
40
30
20
f1 (ppm)

Synthesis of TAMM 1e

Compound **1e'** (550 mg, 1.86 mmol, 93% yield) was obtained as a light yellow solid from silica gel column chromatography (DCM:MeOH=10:1). ^{1}H NMR (400 MHz, Methanol-*d*$_4$) δ 7.856 (dd, *J* = 8.00, 1.20 Hz, 1H), 7.409 (td, *J* = 7.40, 1.20 Hz, 1H), 7.287 (dd, *J* = 7.20, 1.60 Hz, 1H), 7.098 (td, *J* = 7.80, 1.60 Hz, 1H). ^{13}C NMR (151 MHz, Methanol-*d*$_4$) δ 192.799, 145.717, 139.119, 130.071, 127.758, 127.267, 119.796, 118.362, 92.119, 51.863. ESI-(-)-HRMS (M-H)$^-$ calculated for $C_{10}H_5IN_2O$: 294.93738; found: 294.93748 (-0.3 ppm). R$_f$ (DCM:MeOH=10:1) = 0.2.

Compound **1e** (104 mg, 0.28 mmol, 28% yield) was obtained as a light yellow solid from silica gel column chromatography (EA:PE=2:1). ^{1}H NMR (400 MHz, Chloroform-*d*) δ 7.992 (dt, *J* = 7.60, 1.20 Hz, 1H), 7.556 (tdd, *J* = 7.60, 3.60, 1.20 Hz, 1H), 7.247 – 7.165 (m, 2H), 6.265 (dd, *J* = 10.00, 7.20 Hz, 1H), 4.731 – 4.652 (m, 1H), 3.780 (d, *J* = 4.40 Hz, 3H), 3.204 – 2.911 (m, 2H), 2.039 (d, *J* = 1.60 Hz, 3H). ^{13}C NMR (151 MHz, Chloroform-*d*) δ 182.038, 181.923, 170.105, 169.982, 169.483, 169.437, 162.611, 140.593, 140.495, 137.405, 137.315, 132.583, 132.561, 129.459, 129.350, 128.588, 128.394, 111.416, 111.325, 95.153, 94.995, 81.959, 81.840, 53.408, 53.351, 51.085, 50.873, 36.108, 35.951, 23.155, 23.071. ESI-(+)-HRMS (M+H)$^+$ calculated for $C_{16}H_{14}IN_3O_3S$: 455.98733; found: 455.98668 (+1.4 ppm). Rf (EA:PE=2:1) = 0.3.

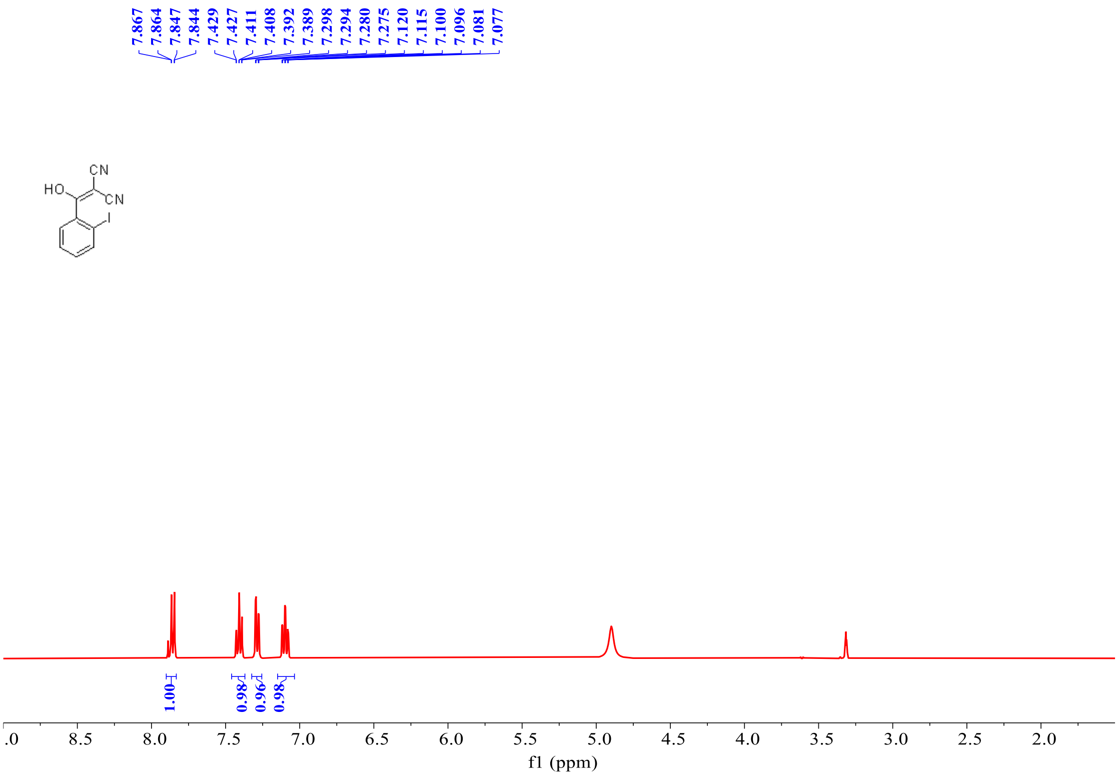

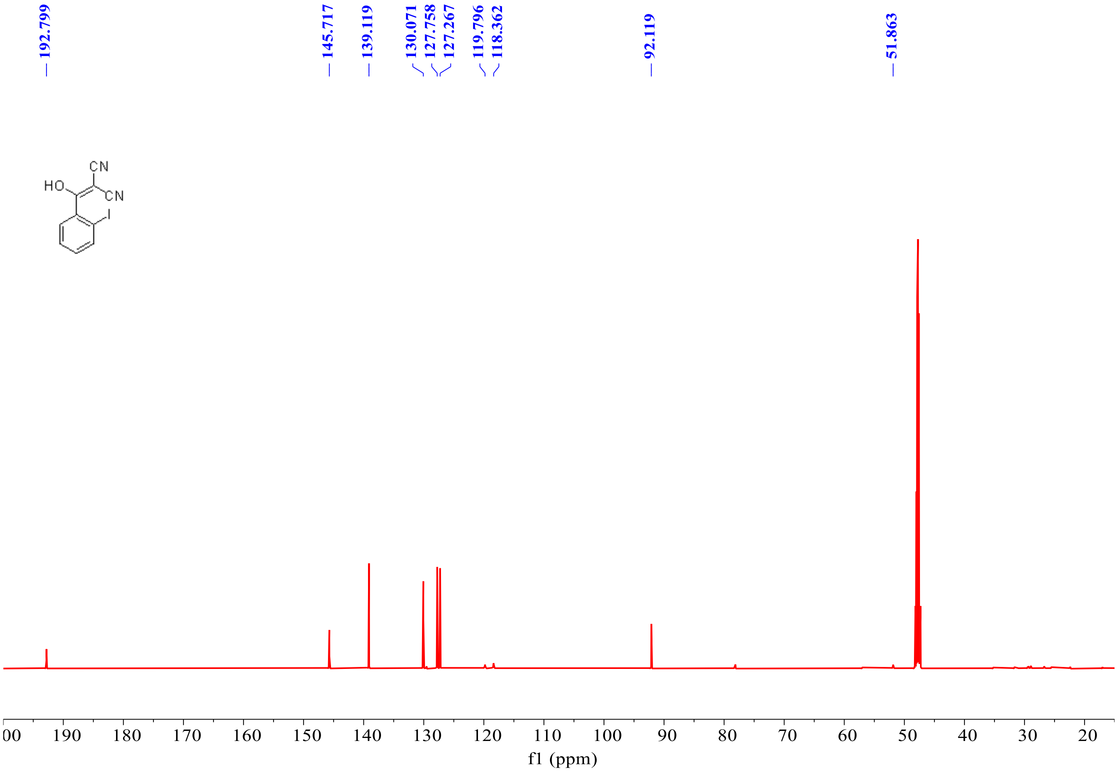
192.799
145.717
139.119
130.071
127.758
127.267
119.796
118.362
92.119
51.863
CN
HO
CN
I
00 190 180 170 160 150 140 130 120 110 100 90 80 70 60 50 40 30 20
f1 (ppm)

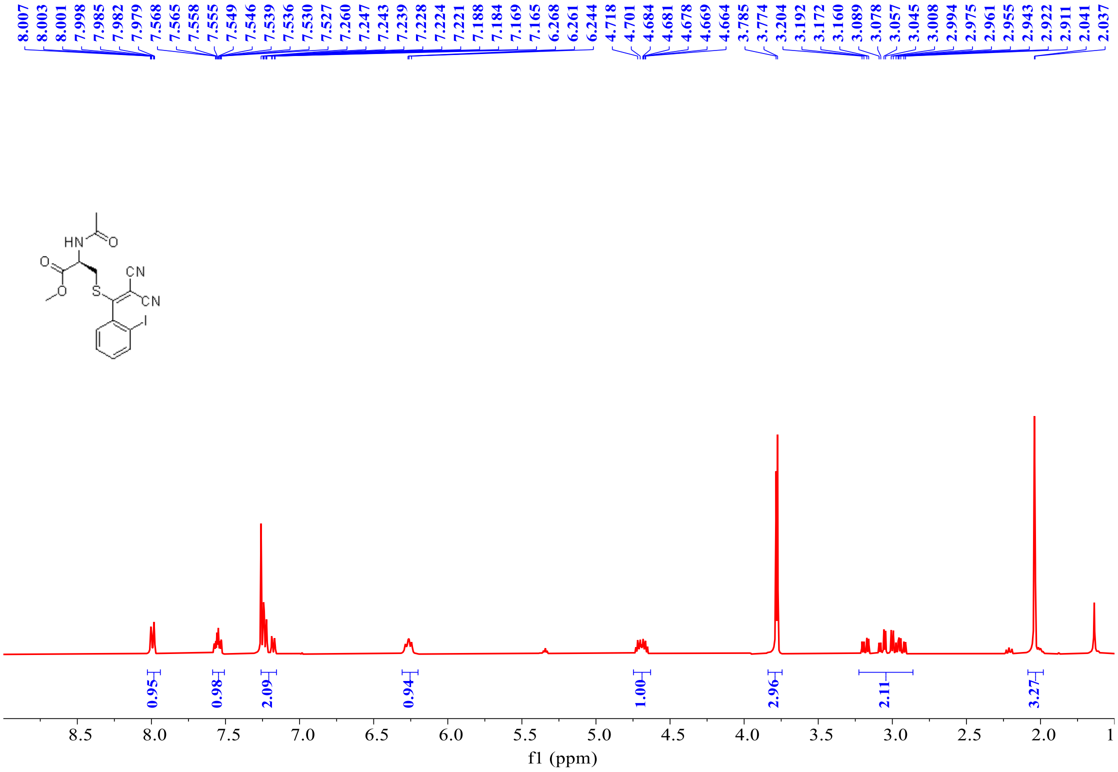
8.007 8.003 8.001 7.998 7.985 7.982 7.979 7.568 7.565 7.558 7.555 7.549 7.546 7.539 7.536 7.530 7.527 7.260 7.247 7.243 7.239 7.228 7.224 7.221 7.188 7.184 7.169 7.165 6.268 6.261 6.244 4.718 4.701 4.684 4.681 4.678 4.669 4.664 3.785 3.774 3.204 3.192 3.172 3.160 3.089 3.078 3.057 3.045 3.008 2.994 2.975 2.961 2.955 2.943 2.922 2.911 2.041 2.037
HN
O
O
O
S
CN
CN
I
0.95
0.98
2.09
0.94
1.00
2.96
2.11
3.27
8.5 8.0 7.5 7.0 6.5 6.0 5.5 5.0 4.5 4.0 3.5 3.0 2.5 2.0 1
f1 (ppm)

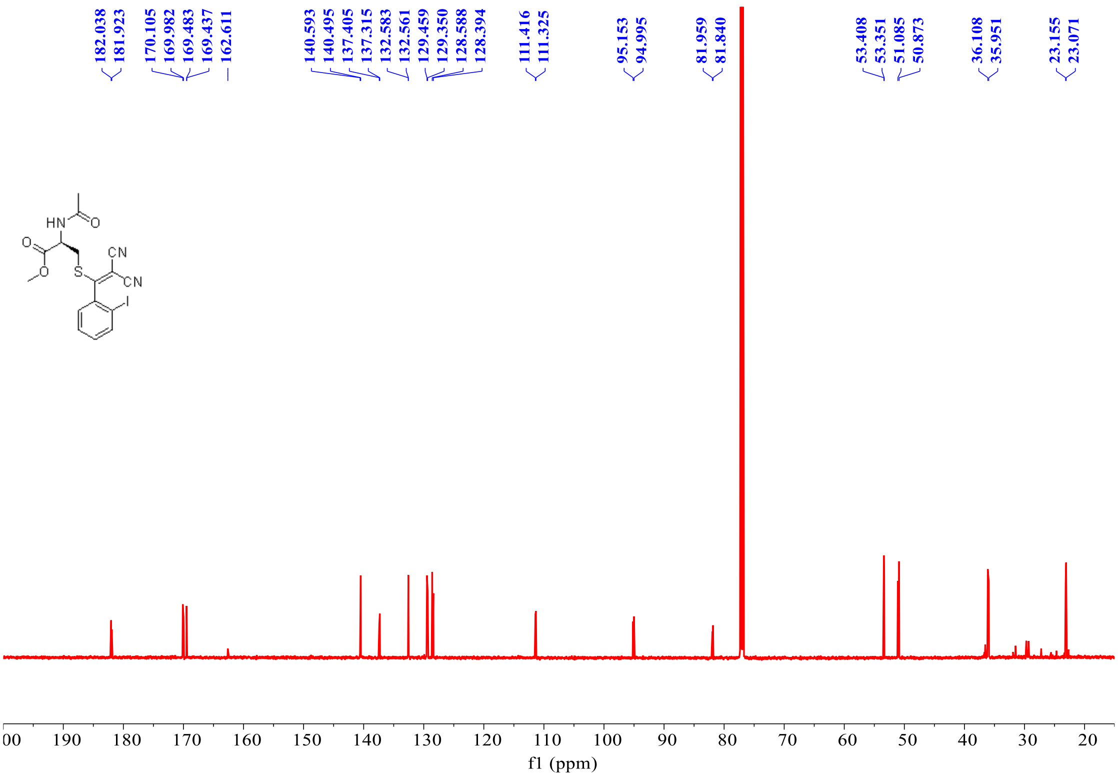
182.038
181.923
170.105
169.982
169.483
169.437
162.611
140.593
140.495
137.405
137.315
132.583
132.561
129.459
129.350
128.588
128.394
111.416
111.325
95.153
94.995
81.959
81.840
53.408
53.351
51.085
50.873
36.108
35.951
23.155
23.071
HN
O
CN
S
I
00 190 180 170 160 150 140 130 120 110 100 90 80 70 60 50 40 30 20
f1 (ppm)

Synthesis of TAMM 1f

Compound **1f'** (476 mg, 1.92 mmol, 96% yield) was obtained as a light yellow solid from silica gel column chromatography (DCM:MeOH=10:1). ^{1}H NMR (400 MHz, Methanol-*d*$_4$) δ 7.776 (dt, *J* = 7.60, 1.00 Hz, 1H), 7.563 – 7.470 (m, 3H). ^{13}C NMR (151 MHz, Methanol-*d*$_4$) δ 183.940, 134.047, 133.225, 132.769, 129.418, 127.841, 119.926, 113.247, 111.624, 64.141. ESI-(-)-HRMS (M-H)$^-$ calculated for $C_{10}H_5BrN_2O$: 246.95125; found: 246.95214 (-3.6 ppm). R_f (DCM:MeOH=10:1) = 0.4.

Compound **1f** (106 mg, 0.26 mmol, 26% yield) was obtained as a light yellow solid from silica gel column chromatography (EA:PE=2:1). ^{1}H NMR (400 MHz, Chloroform-*d*) δ 7.722 (d, *J* = 9.60 Hz, 1H), 7.501 (td, *J* = 7.40, 1.60 Hz, 1H), 7.706 (td, *J* = 7.80, 1.00 Hz, 1H), 7.279 – 7.184 (m, 1H), 6.518 (t, *J* = 6.60 Hz, 1H), 4.678 – 4.590 (m, 1H), 3.718 (d, *J* = 10.40 Hz, 3H), 3.176 – 2.915 (m, 2H), 1.986 (d, *J* = 2.80 Hz, 3H). ^{13}C NMR (101 MHz, Chloroform-*d*) δ 179.865, 179.627, 170.271, 170.183, 169.502, 169.462, 134.128, 134.034, 133.272, 133.170, 132.944, 129.399, 129.195, 128.739, 128.677, 120.811, 120.646, 111.546, 111.372, 81.793, 81.638, 53.297, 51.213, 50.973, 35.942, 35.812, 23.016, 22.987. ESI-(+)-HRMS (M+H)$^+$ calculated for $C_{16}H_{14}BrN_3O_3S$: 408.00120; found: 408.00100 (+0.5 ppm). R_f (EA:PE=3:1) = 0.45.

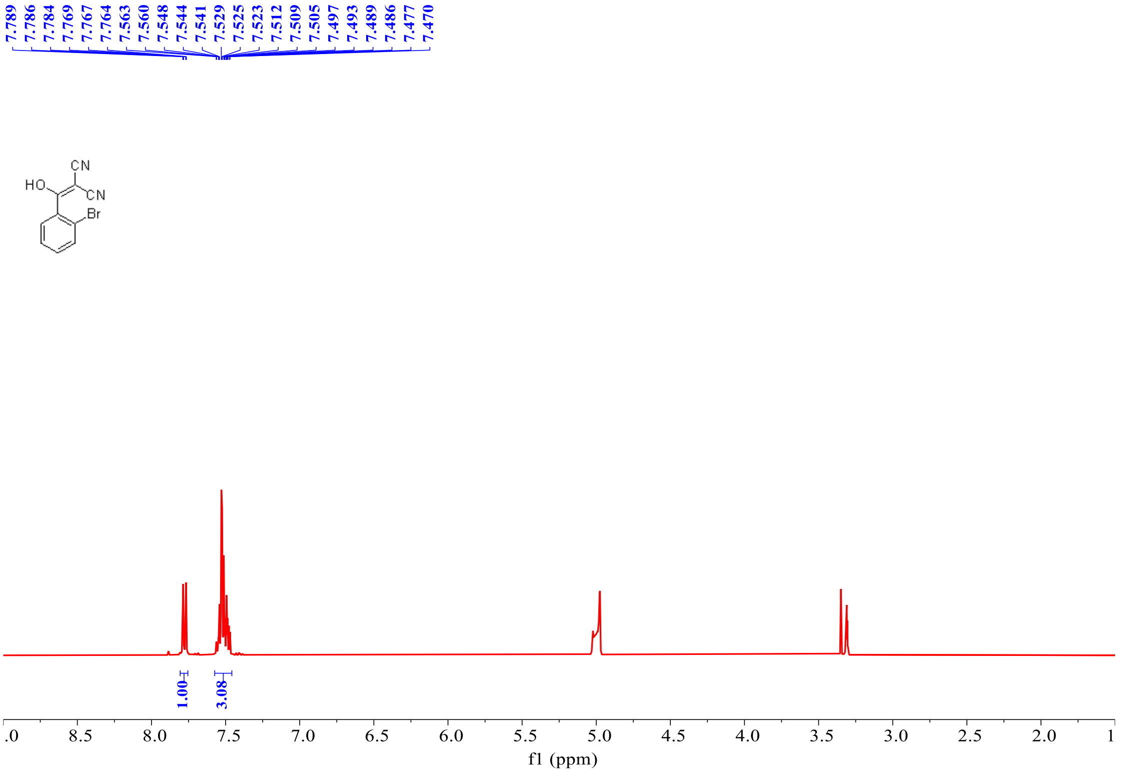

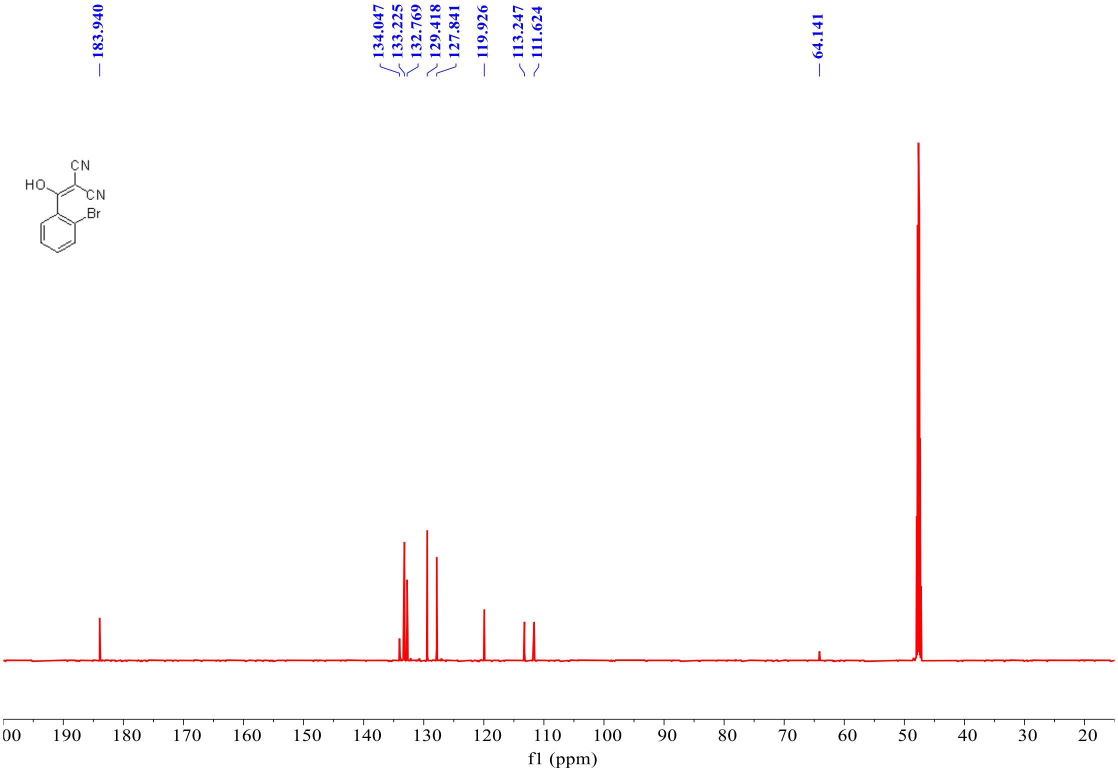

183.940
134.047
133.225
132.769
129.418
127.841
119.926
113.247
111.624
64.141
CN
HO
CN
Br
00 190 180 170 160 150 140 130 120 110 100 90 80 70 60 50 40 30 20
f1 (ppm)

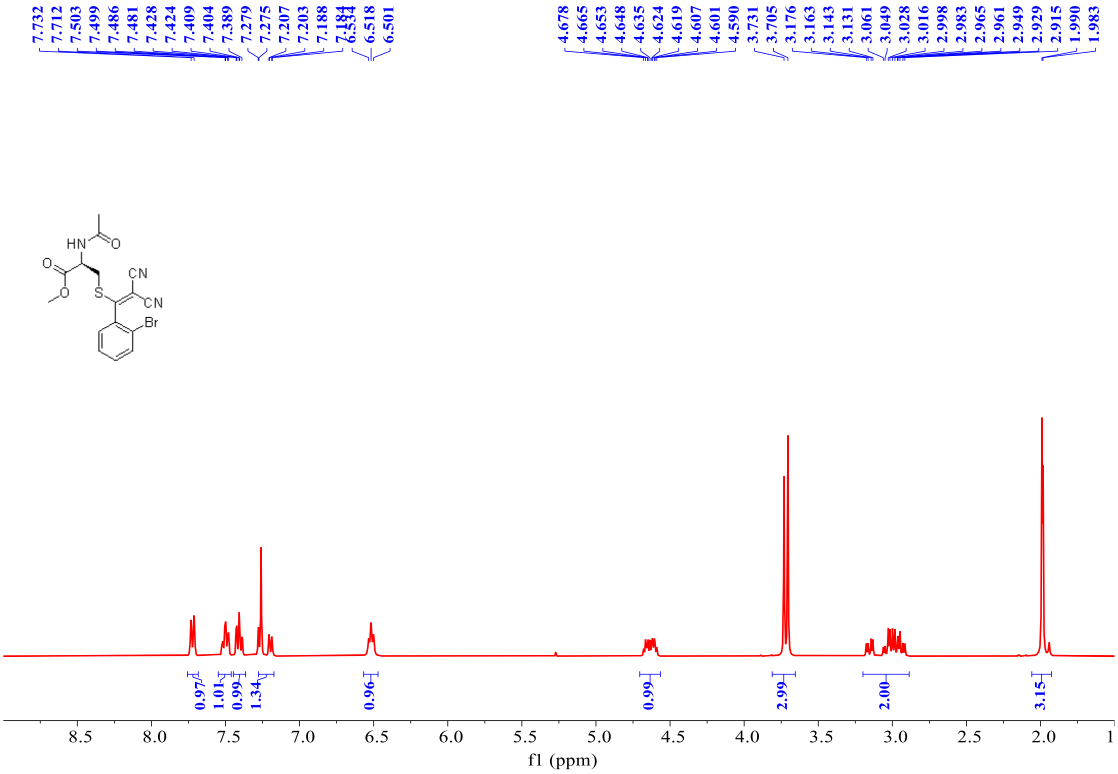

7.732
7.712
7.503
7.499
7.486
7.481
7.428
7.424
7.409
7.404
7.389
7.279
7.275
7.207
7.203
7.188
7.184
6.534
6.518
6.501
4.678
4.665
4.653
4.648
4.635
4.624
4.619
4.607
4.601
4.590
3.731
3.705
3.176
3.163
3.143
3.131
3.061
3.049
3.028
3.016
2.998
2.983
2.965
2.961
2.949
2.929
2.915
1.990
1.983
HN
O
O
O
S
CN
CN
Br
0.97
1.01
0.99
1.34
0.96
0.99
2.99
2.00
3.15
8.5 8.0 7.5 7.0 6.5 6.0 5.5 5.0 4.5 4.0 3.5 3.0 2.5 2.0 1
f1 (ppm)

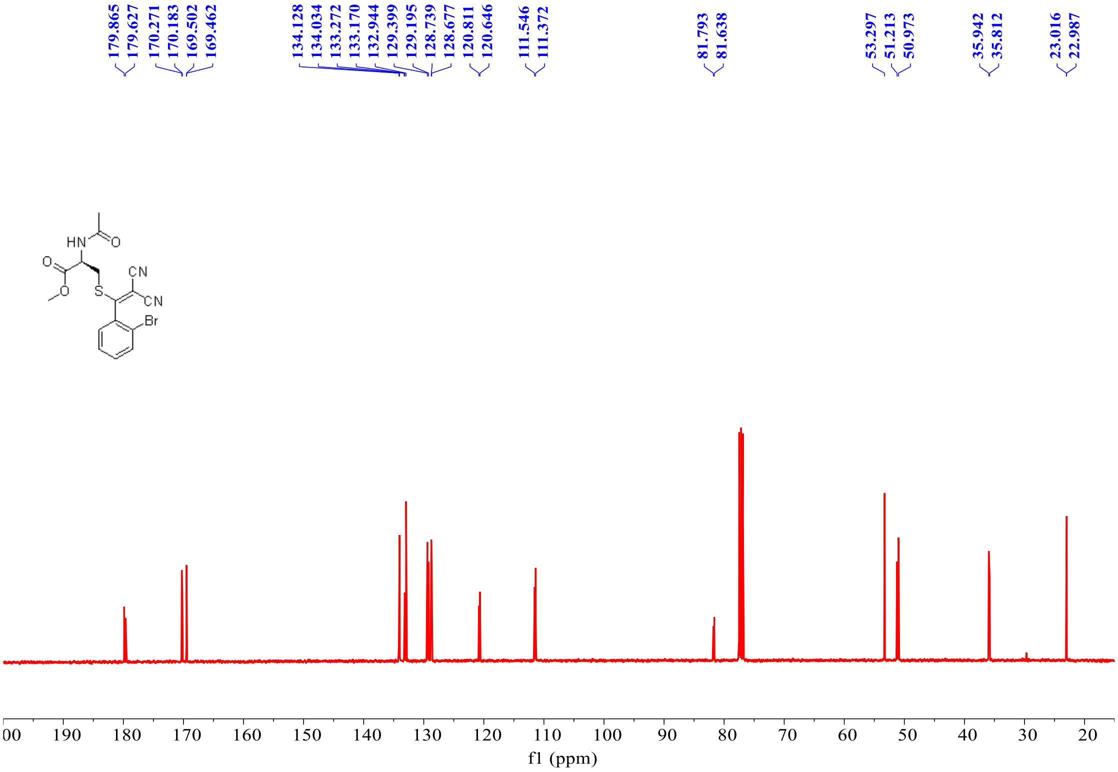
179.865
179.627
170.271
170.183
169.502
169.462
134.128
134.034
133.272
133.170
132.944
129.399
129.195
128.739
128.677
120.811
120.646
111.546
111.372
81.793
81.638
53.297
51.213
50.973
35.942
35.812
23.016
22.987
HN
O
O
O
S
CN
CN
Br
00
190
180
170
160
150
140
130
120
110
100
90
80
70
60
50
40
30
20
f1 (ppm)

Synthesis of TAMM 1g

Compound **1g'** (396 mg, 1.94 mmol, 97% yield) was obtained as a light yellow solid from silica gel column chromatography (DCM:MeOH=10:1). ^{1}H NMR (400 MHz, Methanol-d_4) δ 7.309 – 7.279 (m, 1H), 7.261 – 7.183 (m, 3H). ^{13}C NMR (151 MHz, Methanol-d_4) δ 190.039, 139.653, 130.278, 130.037, 129.341, 128.196, 126.483, 120.034, 118.450, 52.258. ESI-(-)-HRMS $(M-H)^{-}$ calculated for $C_{10}H_5ClN_2O$: 203.00176; found: 203.00239 (-3.1 ppm). R_f (DCM:MeOH=5:1) = 0.4.

Compound **1g** (113 mg, 0.31 mmol, 31% yield) was obtained as a light yellow solid from silica gel column chromatography (EA:PE=2:1). ^{1}H NMR (400 MHz, Chloroform-*d*) δ 7.565 – 7.429 (m, 3H), 7.294 – 7.199 (m, 1H), 6.510 (t, J = 6.80 Hz, 1H), 4.669 – 4.581 (m, 1H), 3.717 (d, J = 11.20 Hz, 3H), 3.165 – 2.47 (m, 2H), 1.984 (d, J = 3.0 Hz, 3H). ^{13}C NMR (101 MHz, Chloroform-*d*) δ 178.417, 178.142, 170.263, 170.189, 169.503, 169.453, 133.998, 131.619, 131.427, 131.153, 131.078, 130.968, 130.864, 129.492, 129.300, 128.180, 128.118, 111.585, 111.380, 81.937, 81.764, 53.295, 53.268, 51.300, 51.058, 35.869, 35.772, 22.975. ESI-(+)-HRMS $(M+H)^{+}$ calculated for $C_{16}H_{14}ClN_3O_3S$: 364.05172; found: 364.05138 (+0.9 ppm). R_f (EA:PE=3:1) = 0.60.

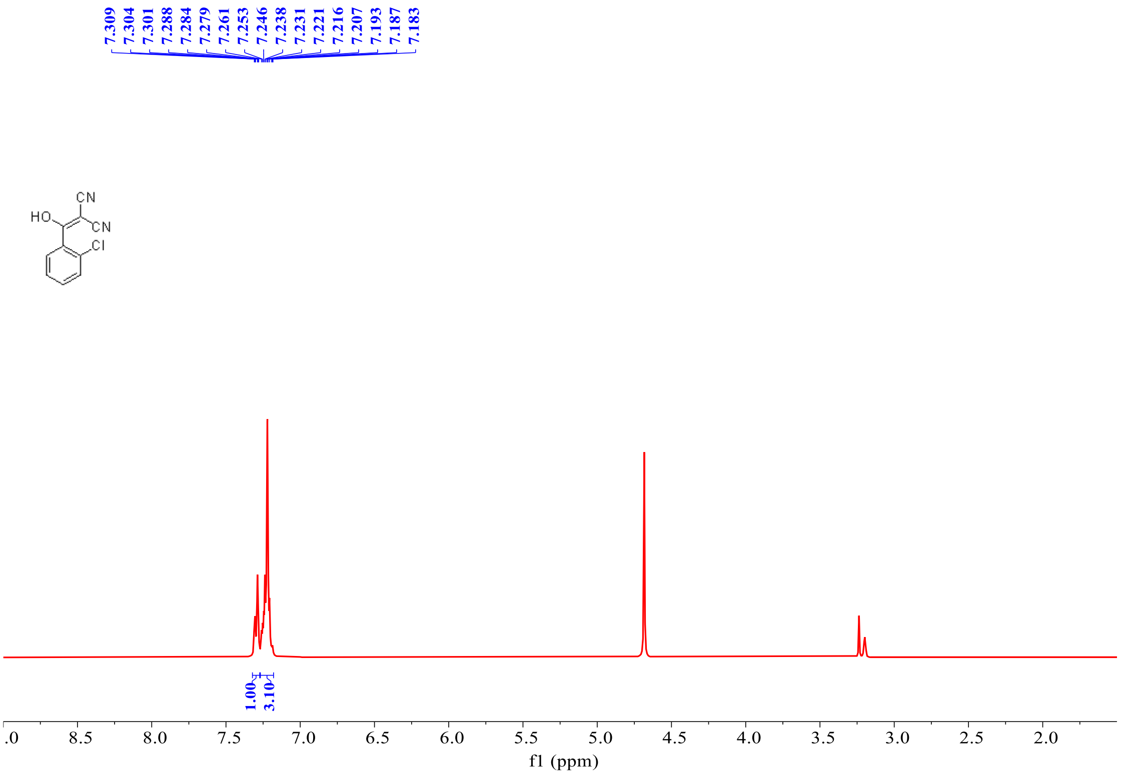

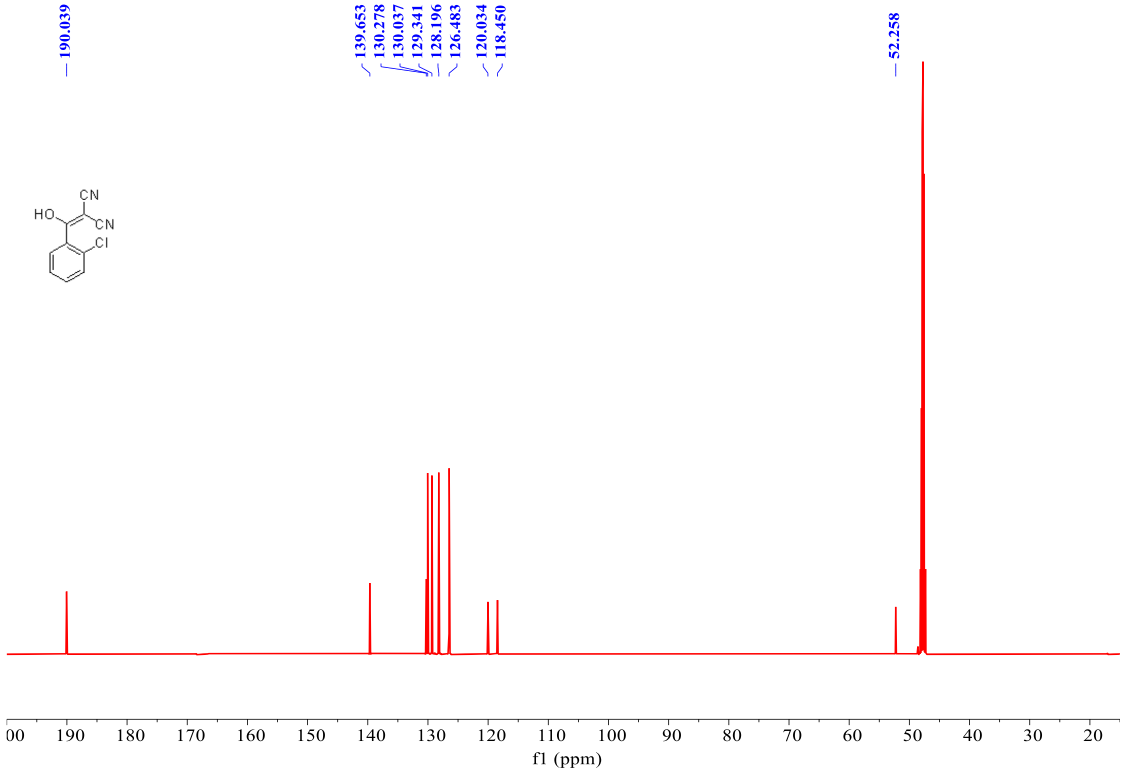

190.039
139.653
130.278
130.037
129.341
128.196
126.483
120.034
118.450
52.258
HO
CN
CN
Cl
f1 (ppm)

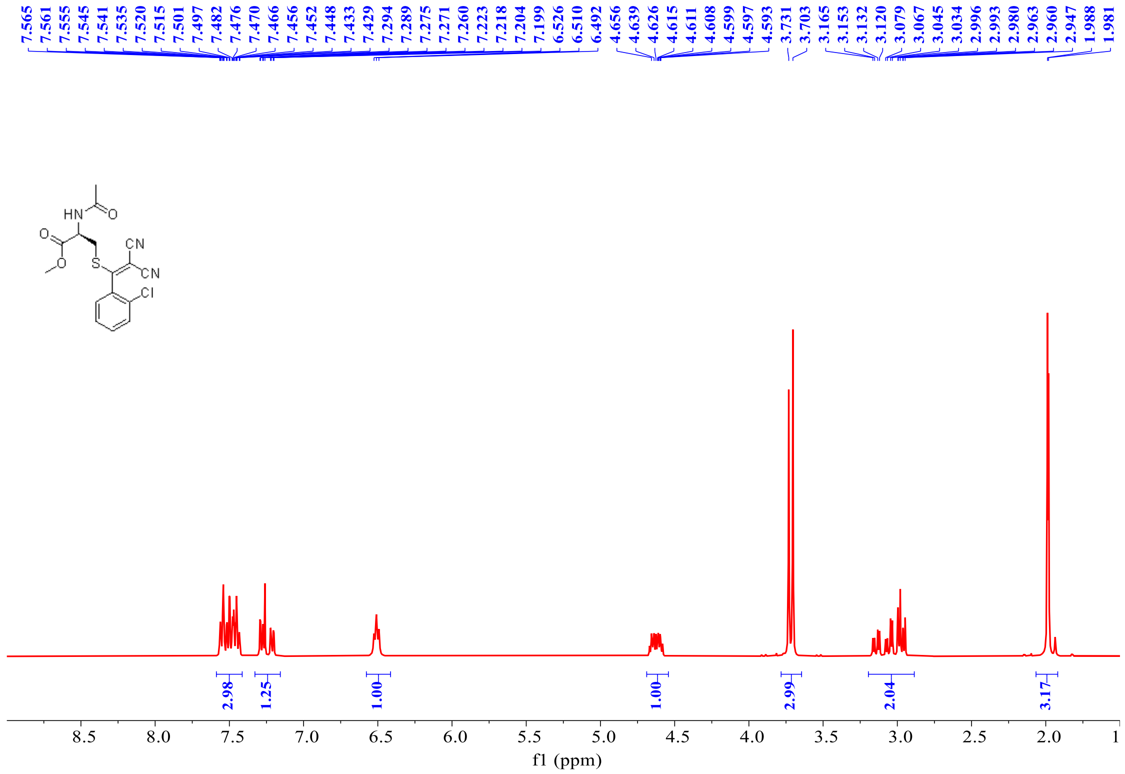

7.565
7.561
7.555
7.545
7.541
7.535
7.520
7.515
7.501
7.497
7.482
7.476
7.470
7.466
7.456
7.452
7.448
7.433
7.429
7.294
7.289
7.275
7.271
7.260
7.223
7.218
7.204
7.199
6.526
6.510
6.492
4.656
4.639
4.626
4.615
4.611
4.608
4.599
4.597
4.593
3.731
3.703
3.165
3.153
3.132
3.120
3.079
3.067
3.045
3.034
2.996
2.993
2.980
2.963
2.960
2.947
1.988
1.981
HN
O
O
O
S
CN
CN
Cl
2.98
1.25
1.00
1.00
2.99
2.04
3.17
f1 (ppm)

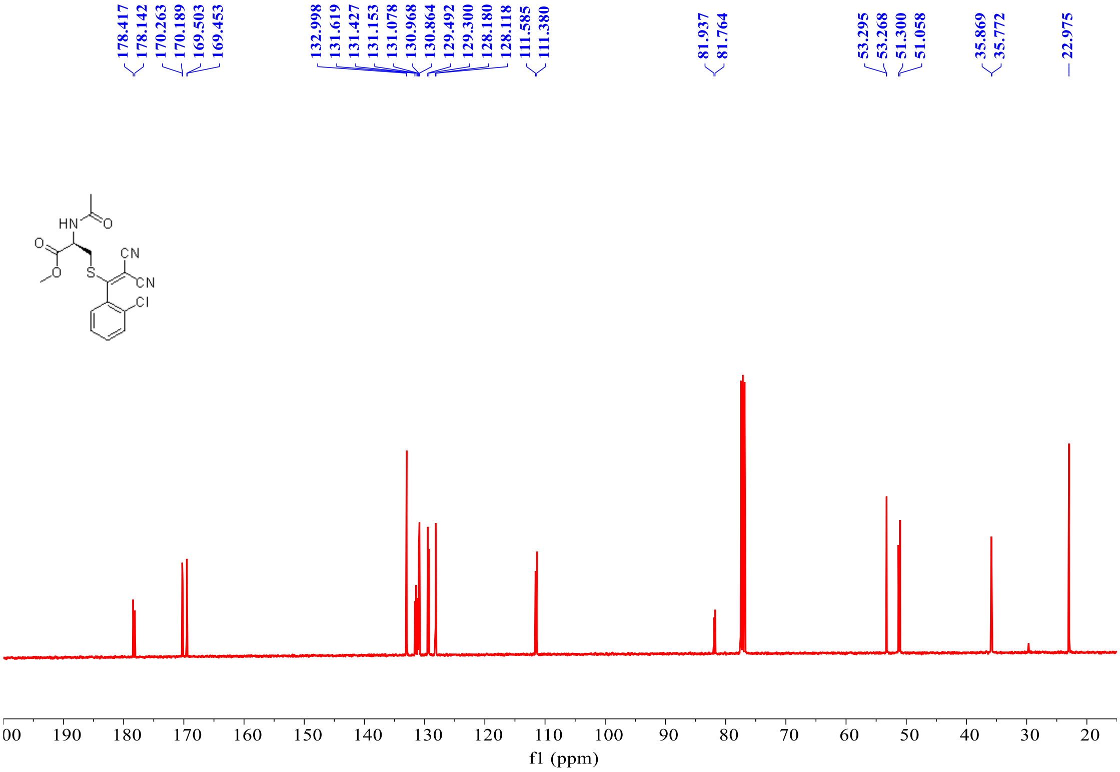
178.417
178.142
170.263
170.189
169.503
169.453
132.998
131.619
131.427
131.153
131.078
130.968
130.864
129.492
129.300
128.180
128.118
111.585
111.380
81.937
81.764
53.295
53.268
51.300
51.058
35.869
35.772
22.975
f1 (ppm)

Synthesis of TAMM 1h

Compound **1h′** (368 mg, 1.96 mmol, 98% yield) was obtained as a light yellow solid from silica gel column chromatography (DCM:MeOH=10:1). ^{1}H NMR (400 MHz, Methanol-*d*$_4$) δ 7.445 – 7.385 (m, 2H), 7.192 (td, *J* = 7.60, 1.20 Hz, 1H), 7.141 – 7.091 (m, 1H). ^{13}C NMR (151 MHz, Methanol-*d*$_4$) δ 188.973, 160.847, 159.203, 132.488, 132.435, 130.196, 130.173, 129.530, 129.418, 125.053, 125.029, 121.552, 120.043, 116.785, 116.640, 53.539. ^{19}F NMR (376 MHz, Methanol-*d*$_4$) δ -116.794 (dt, *J* = 9.75, 6.33 Hz). ESI-(-)-HRMS (M-H)$^-$ calculated for $C_{10}H_5FN_2O$: 187.03131; found: 187.03128 (+0.2 ppm). R$_f$ (DCM:MeOH=5:1) = 0.4.

Compound **1h** (87 mg, 0.25 mmol, 25% yield) was obtained as a light yellow solid from silica gel column chromatography (EA:PE=2:1). ^{1}H NMR (400 MHz, Chloroform-*d*) δ 7.604 – 7.547 (m, 1H), 7.356 – 7.232 (m, 3H), 6.545 (d, *J* = 7.20 Hz, 1H), 4.62 (dt, *J* = 7.20, 3.60 Hz, 1H), 3.727 (s, 3H), 3.178 (dd, *J* = 14.00, 4.80 Hz, 1H), 3.07 (dd, *J* = 14.00, 5.60 Hz, 1H), 1.979 (s, 3H). ^{13}C NMR (151 MHz, Chloroform-*d*) δ 174.453, 170.276, 169.472, 159.085, 157.405, 134.659, 134.604, 130.040, 125.576, 125.552, 119.922, 119.829, 111.889, 111.571, 82.534, 53.265, 51.675, 36.132, 22.900. ^{19}F NMR (376 MHz, Chloroform-*d*) δ -111.298 (q, *J* = 7.52, 6.77 Hz). ESI-(-)-HRMS (M-H)$^-$ calculated for $C_{16}H_{14}FN_3O_3S$: 346.06671; found: 346.06695 (-0.7 ppm). R$_f$ (EA:PE=3:1) = 0.40.

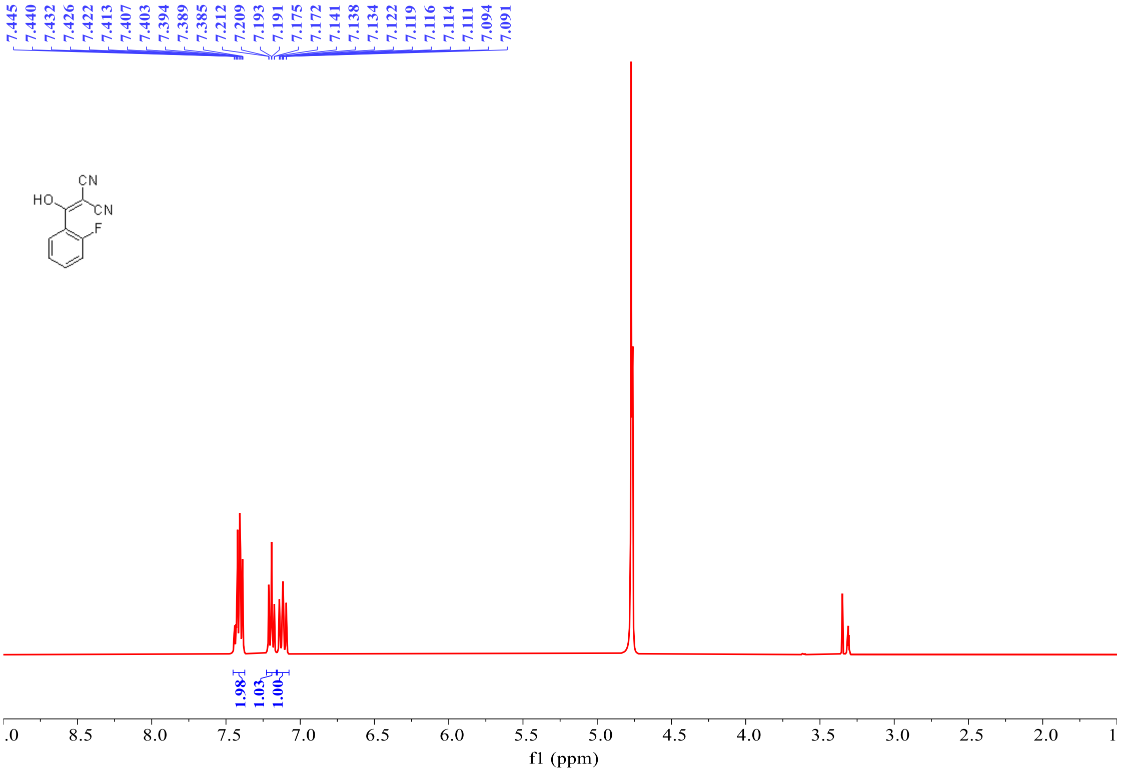

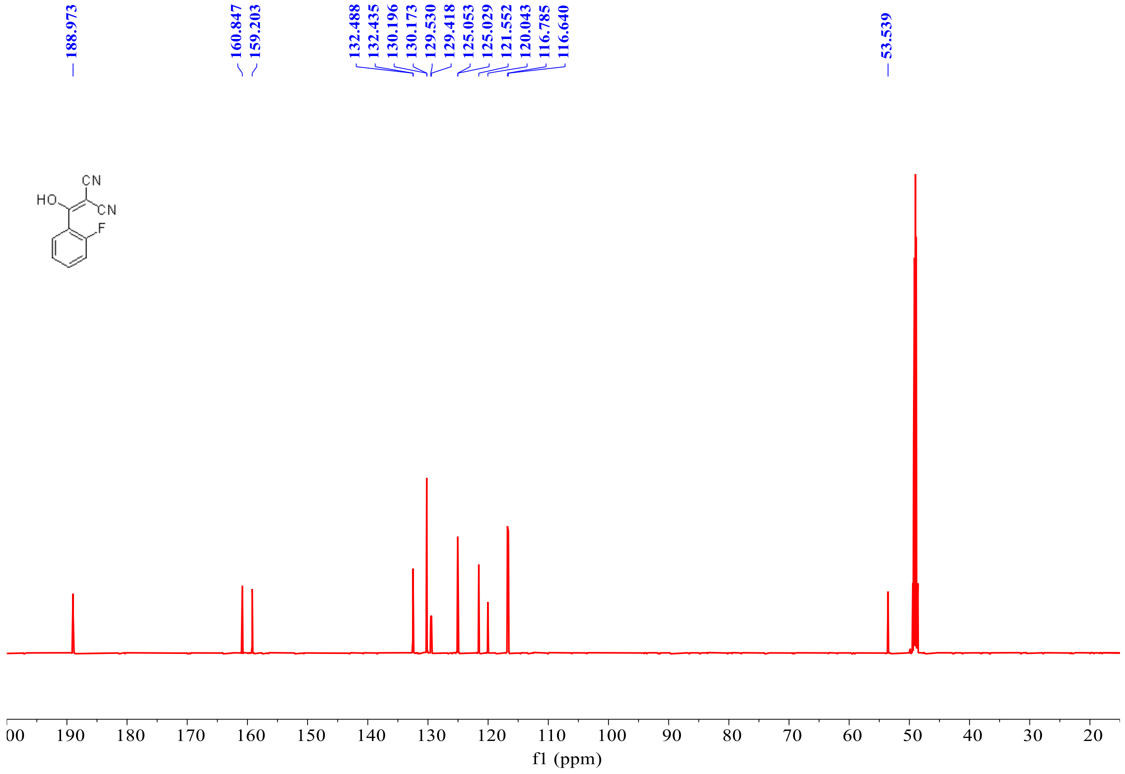
188.973
160.847
159.203
132.488
132.435
130.196
130.173
129.530
129.418
125.053
125.029
121.552
120.043
116.785
116.640
53.539
CN
HO
CN
F
00 190 180 170 160 150 140 130 120 110 100 90 80 70 60 50 40 30 20
f1 (ppm)

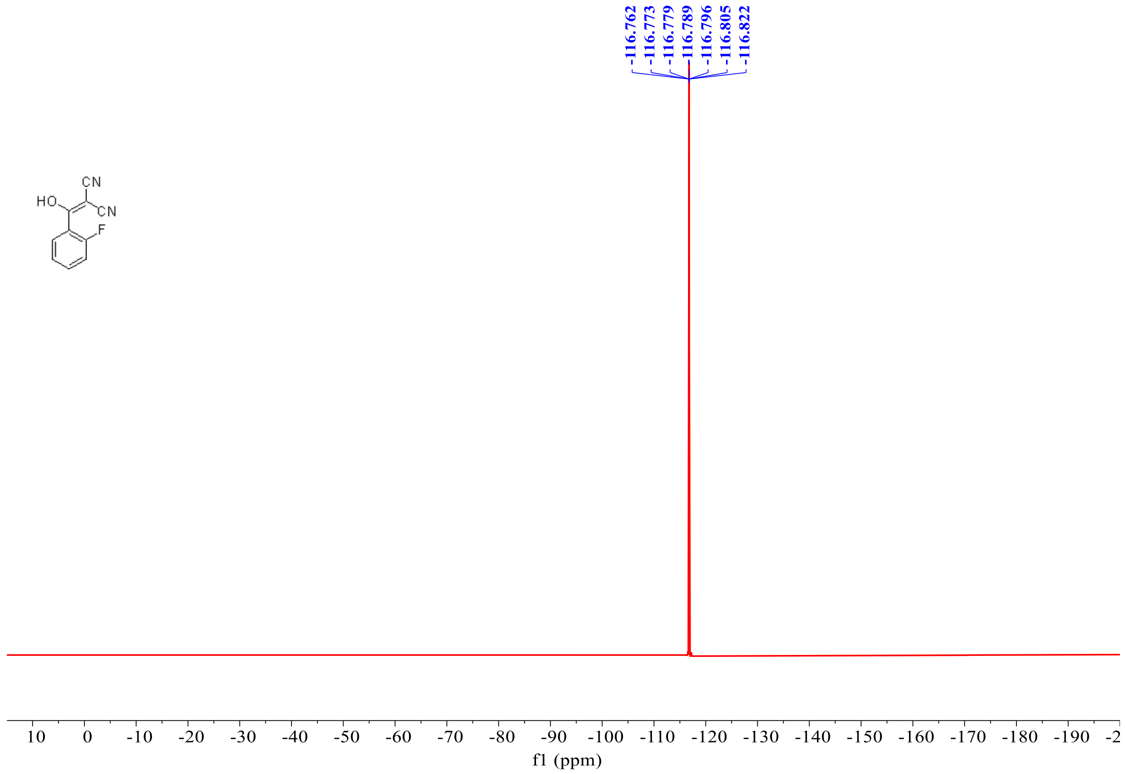
-116.762
-116.773
-116.779
-116.789
-116.796
-116.805
-116.822
CN
HO
CN
F
10 0 -10 -20 -30 -40 -50 -60 -70 -80 -90 -100 -110 -120 -130 -140 -150 -160 -170 -180 -190 -2
f1 (ppm)

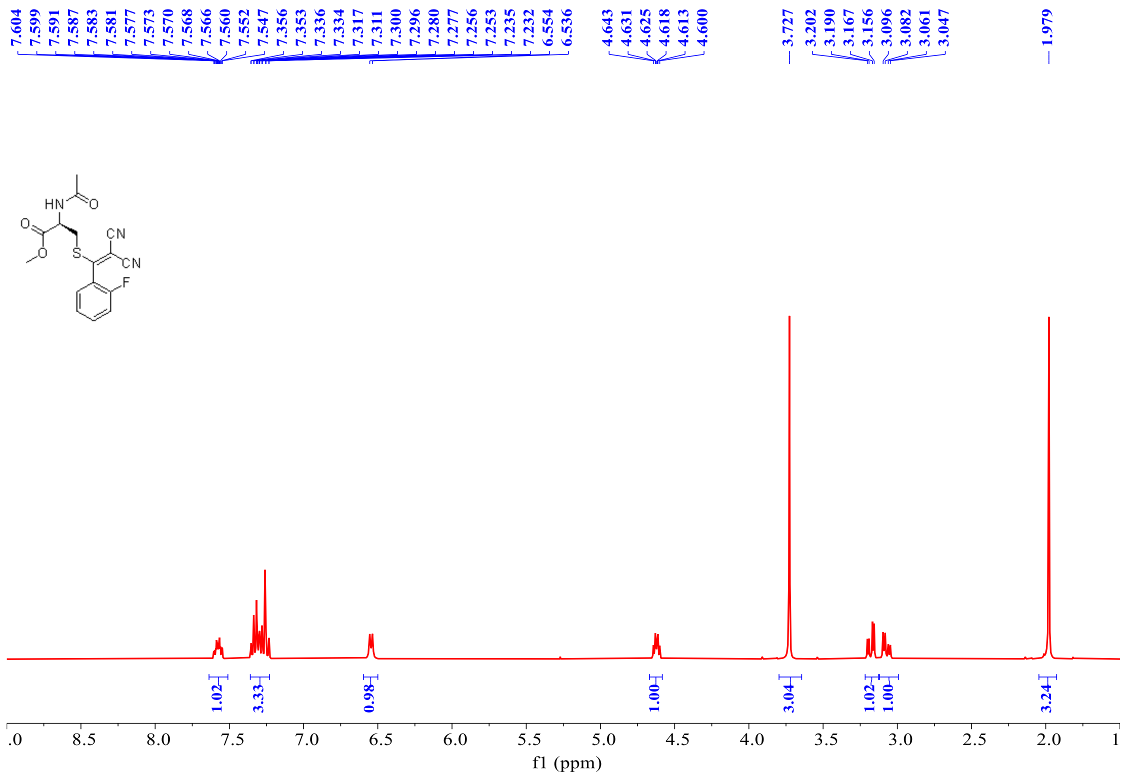

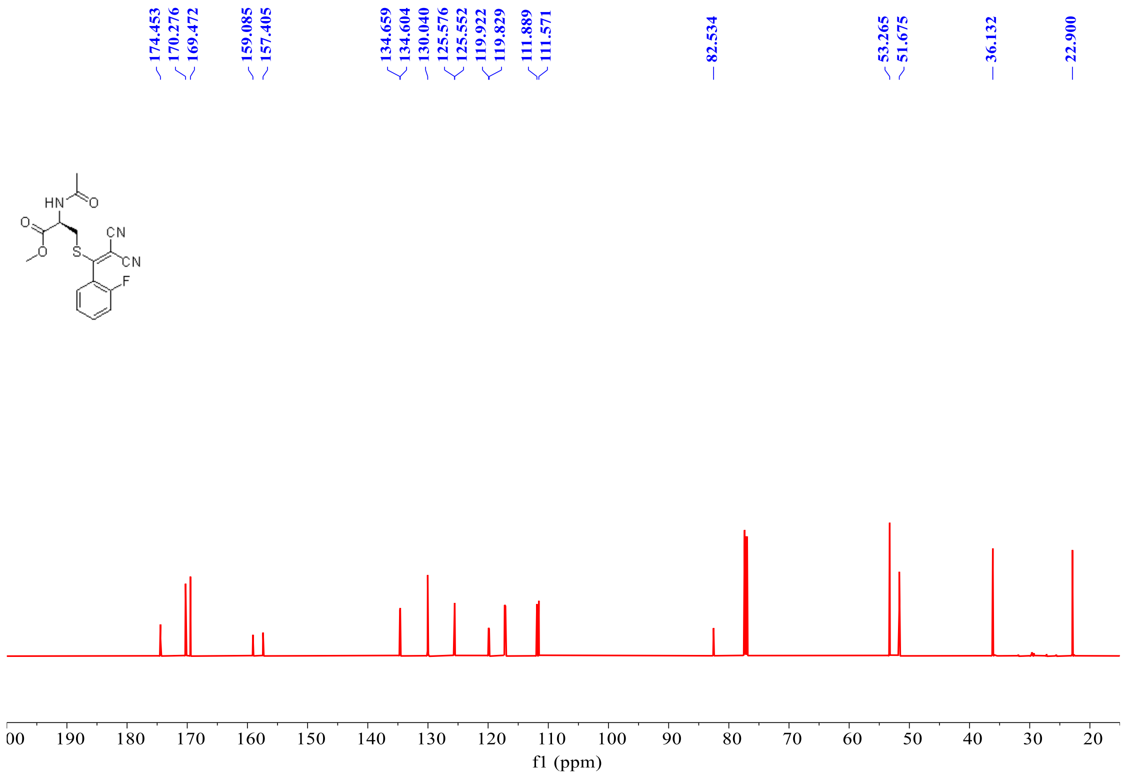

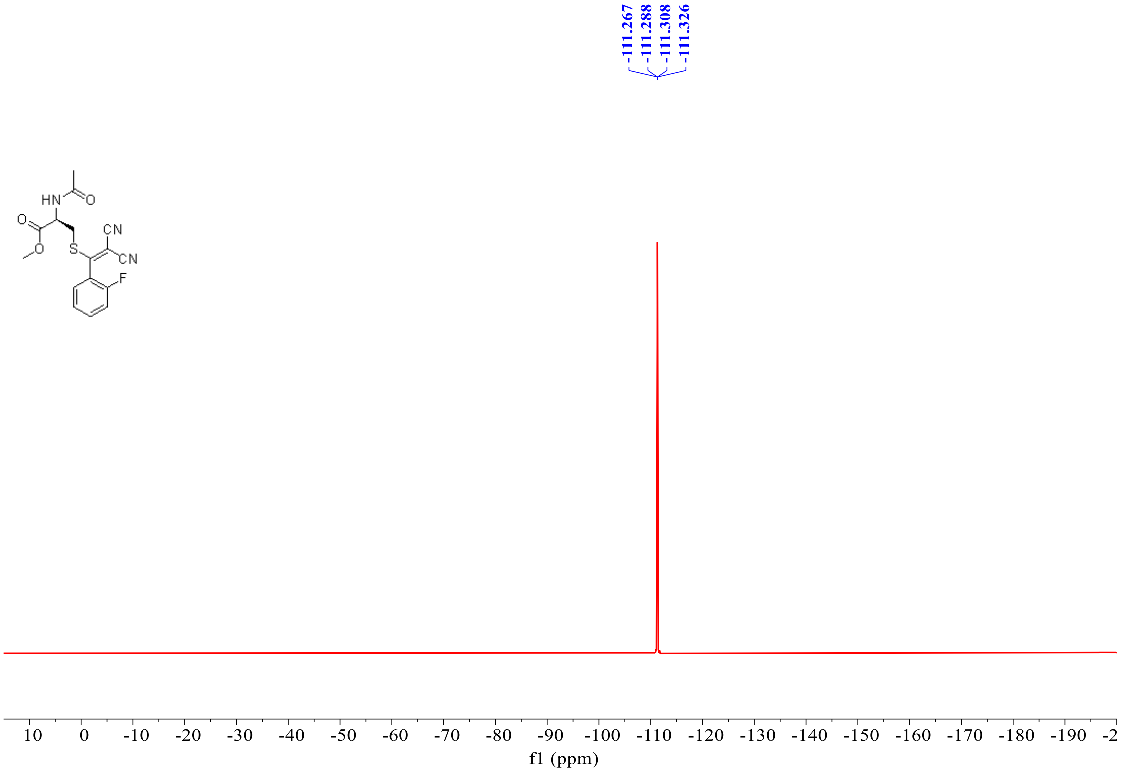
-111.267
-111.288
-111.308
-111.326
HN
O
O
O
S
CN
CN
F
10 0 -10 -20 -30 -40 -50 -60 -70 -80 -90 -100 -110 -120 -130 -140 -150 -160 -170 -180 -190 -2
f1 (ppm)

Synthesis of TAMM 1i

Compound **1i'** (380 mg, 1.9 mmol, 95% yield) was obtained as a light yellow solid from silica gel column chromatography (DCM:MeOH=10:1). ^{1}H NMR (400 MHz, Methanol-*d*$_4$) δ 7.574 (ddd, *J* = 9.20, 7.60, 1.60 Hz, 1H), 7.405 (dd, *J* = 7.60, 2.00 Hz, 1H), 7.172 (dd, *J* = 8.40, 0.80 Hz, 1H), 7.083 (td, *J* = 7.60, 0.80 Hz, 1H), 3.911 (s, 3H). ^{13}C NMR (151 MHz, Methanol-*d*$_4$) δ 185.104, 158.150, 134.994, 130.544, 122.104, 121.627, 115.386, 113.633, 112.948, 64.458, 56.376. ESI-(-)-HRMS $(M-H)^{-}$ calculated for $C_{11}H_8N_2O_2$: 199.05130; found: 199.05143 (-0.6 ppm). R_f (DCM:MeOH=10:1) = 0.25.

Compound **1i** (108 mg, 0.3 mmol, 30% yield) was obtained as a light yellow solid from silica gel column chromatography (EA:PE=2:1). ^{1}H NMR (600 MHz, Chloroform-*d*) δ 7.505 (ddd, *J* = 9.60, 7.20, 1.20 Hz, 1H), 7.143 (dd, *J* = 7.80, 1.80 Hz, 1H), 7.084 (t, *J* = 7.50 Hz, 1H), 7.039 (d, *J* = 8.40 Hz, 1H), 6.427 (d, *J* = 7.20 Hz, 1H), 4.616 – 4.585 (m, 1H),, 3.883 (s, 3H), 3.704 (s, 3H), 3.112 (dd, *J* = 13.80, 4.80 Hz, 1H), 2.986 (dd, *J* = 13.20, 5.40 Hz, 1H), 1.967 (s, 3H). ^{13}C NMR (151 MHz, Chloroform-*d*) δ 178.461, 170.036, 169.739, 155.793, 133.845, 129.478, 121.529, 120.627, 112.455, 112.228, 112.059, 81.209, 55.997, 53.119, 51.531, 35.766, 22.933. ESI-(+)-HRMS $(M+H)^{+}$ calculated for $C_{17}H_{17}N_3O_4S$: 360.10125; found: 360.10087 (+1.0 ppm). R_f (EA:PE=3:1) = 0.30.

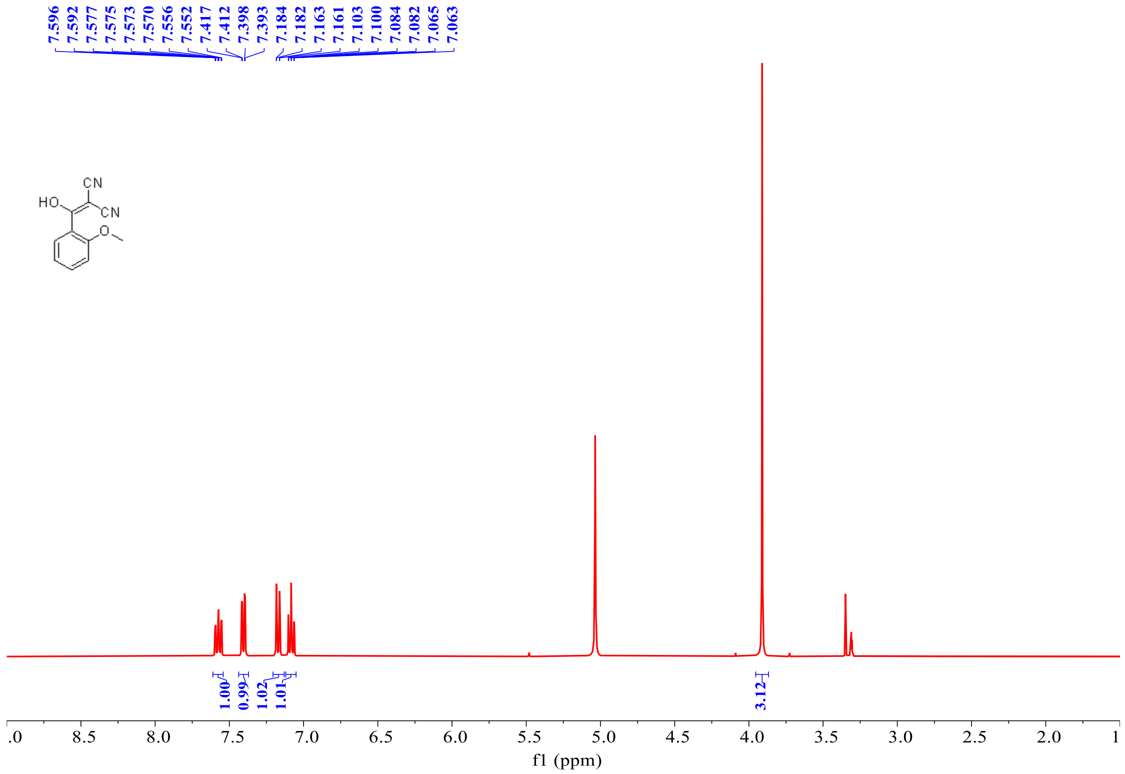

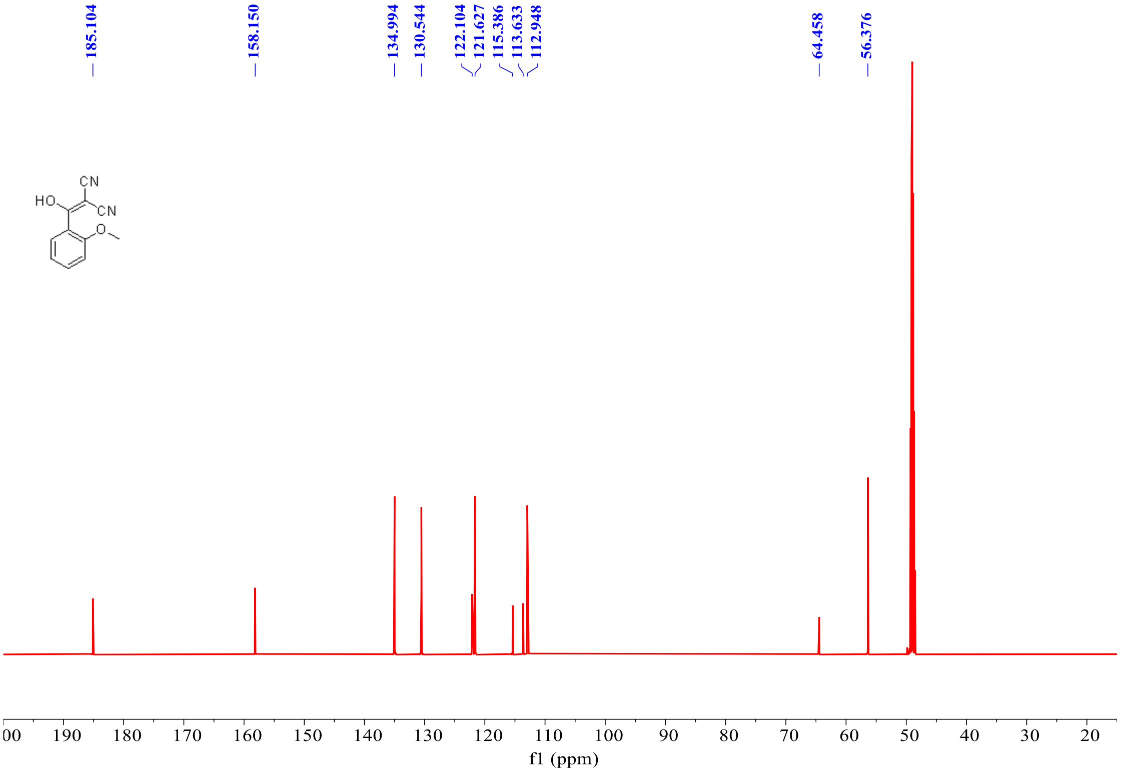
185.104
158.150
134.994
130.544
122.104
121.627
115.386
113.633
112.948
64.458
56.376
HO
CN
CN
O
f1 (ppm)

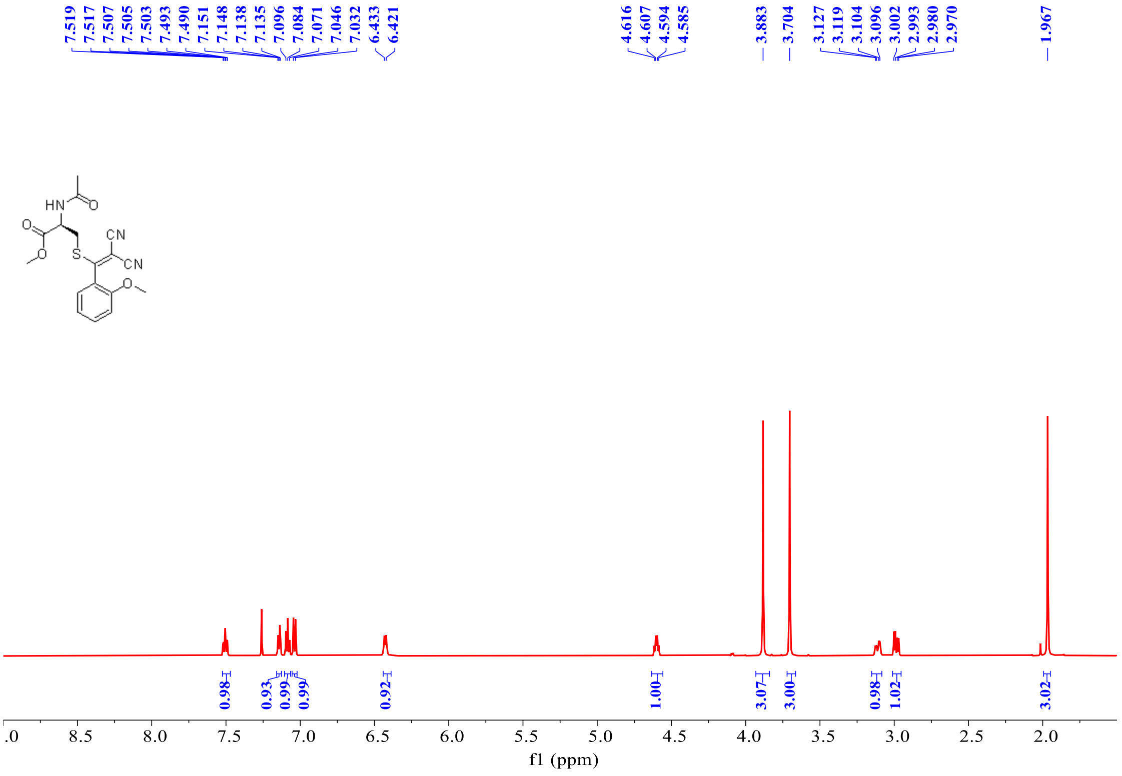
7.519
7.517
7.507
7.505
7.503
7.493
7.490
7.151
7.148
7.138
7.135
7.096
7.084
7.071
7.046
7.032
6.433
6.421
4.616
4.607
4.594
4.585
3.883
3.704
3.127
3.119
3.104
3.096
3.002
2.993
2.980
2.970
1.967
HN
O
O
O
S
CN
CN
O
0.98
0.93
0.99
0.99
0.92
1.00
3.07
3.00
0.98
1.02
3.02
f1 (ppm)

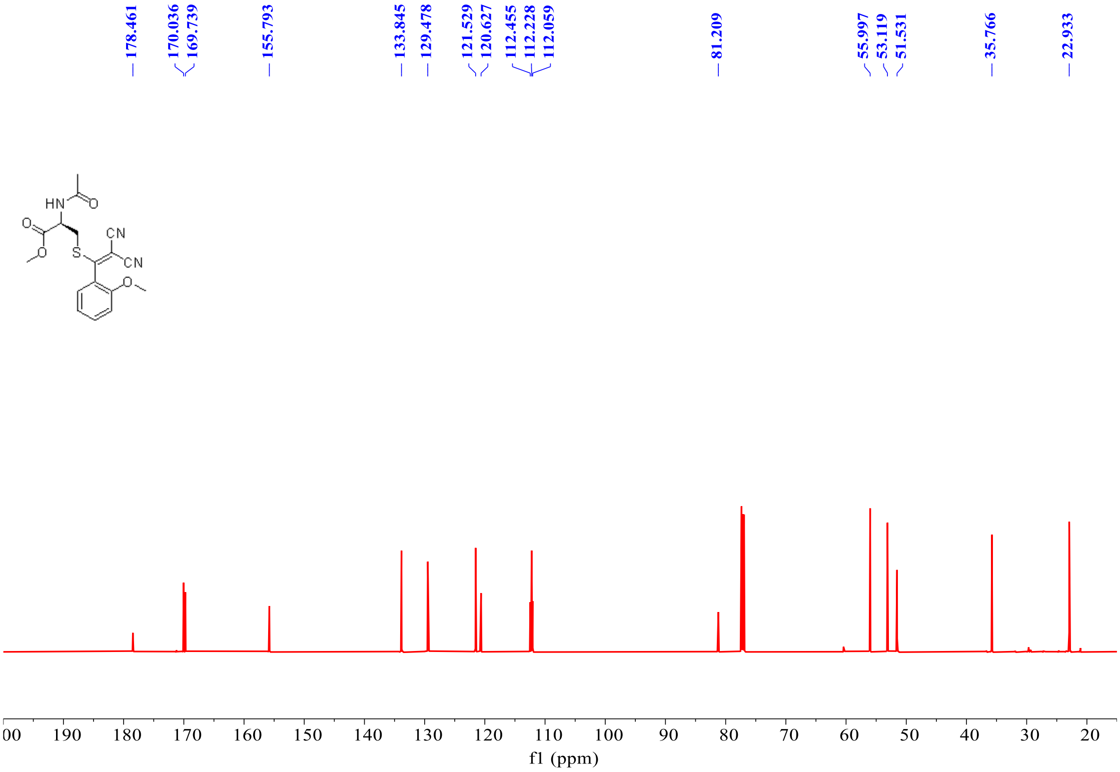
178.461
170.036
169.739
155.793
133.845
129.478
121.529
120.627
112.455
112.228
112.059
81.209
55.997
53.119
51.531
35.766
22.933
HN
O
O
O
S
CN
CN
O
00
190
180
170
160
150
140
130
120
110
100
90
80
70
60
50
40
30
20
f1 (ppm)

Synthesis of TAMM 1j

Compound **1j'** (297 mg, 1.98 mmol, 99% yield) was obtained as a light yellow solid from silica gel column chromatography (DCM:MeOH=10:1). ^{1}H NMR (400 MHz, Methanol-d_4) δ 1.263 (s, 9H). ^{13}C NMR (151 MHz, Methanol-d_4) δ 203.266, 50.431, 42.560, 27.978. ESI-(-)-HRMS $(M-H)^-$ calculated for $C_8H_{10}N_2O$: 149.07204; found: 149.07198 (+0.4 ppm). R_f (DCM:MeOH=5:1) = 0.3.

Compound **1j** (102 mg, 0.33 mmol, 33% yield) was obtained as a light yellow solid from silica gel column chromatography (EA:PE=2:1). ^{1}H NMR (400 MHz, Chloroform-*d*) δ 6.423 (d, *J* = 7.60 Hz, 1H), 4.957 – 4.912 (m, 1H), 3.876 – 3.831 (m, 1H), 3.826 (s, 3H), 3.656 (dd, *J* = 13.20, 5.60 Hz, 1H), 2.071 (s, 3H), 1.454 (s, 9H). ^{13}C NMR (151 MHz, Chloroform-*d*) δ 193.400, 170.054, 169.912, 113.949, 113.285, 82.104, 53.295, 51.782, 42.893, 39.817, 29.211, 23.030. ESI-(+)-HRMS $(M+H)^+$ calculated for $C_{14}H_{19}N_3O_3S$: 310.12199; found: 310.12164 (+1.1 ppm). R_f (EA:PE=2:1) = 0.40.

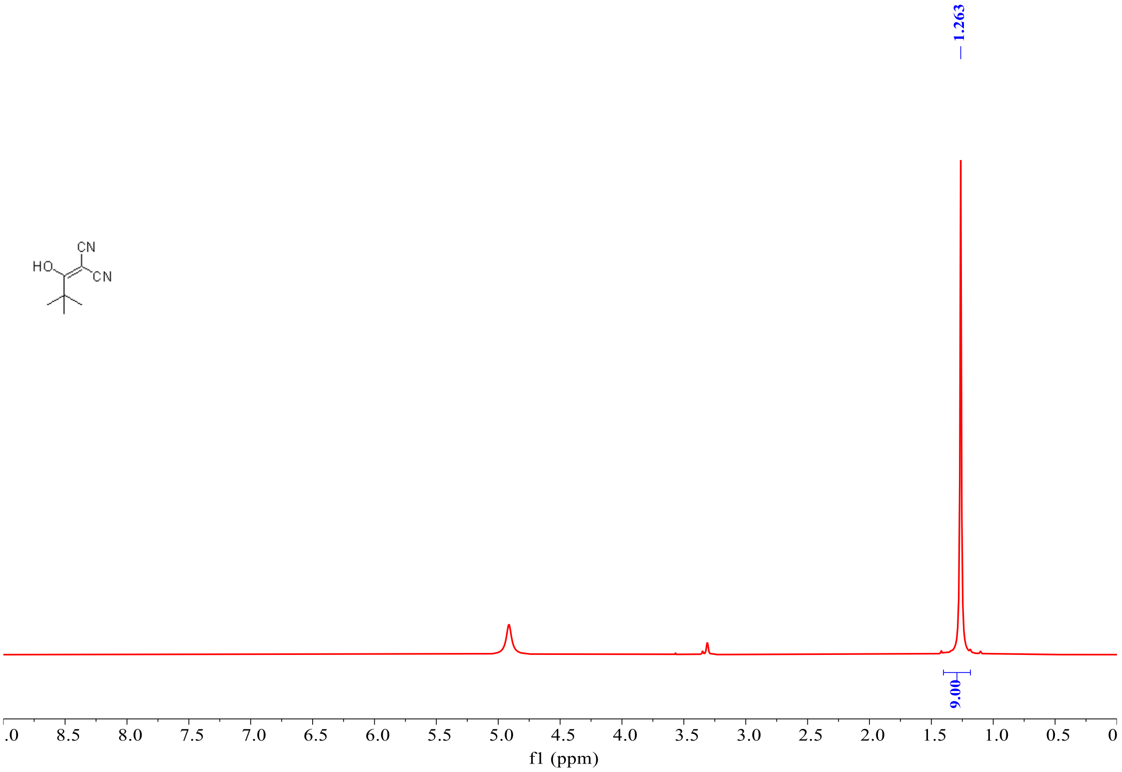

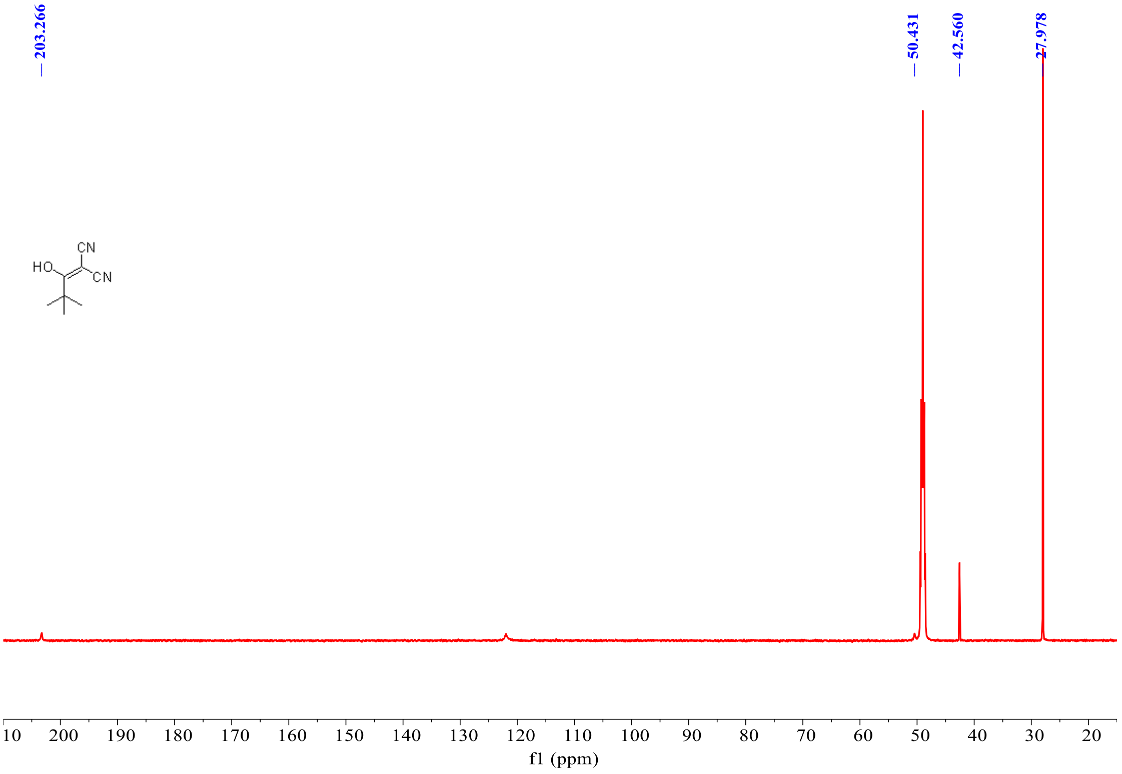
203.266
50.431
42.560
27.978
HO
CN
CN
10 200 190 180 170 160 150 140 130 120 110 100 90 80 70 60 50 40 30 20
f1 (ppm)

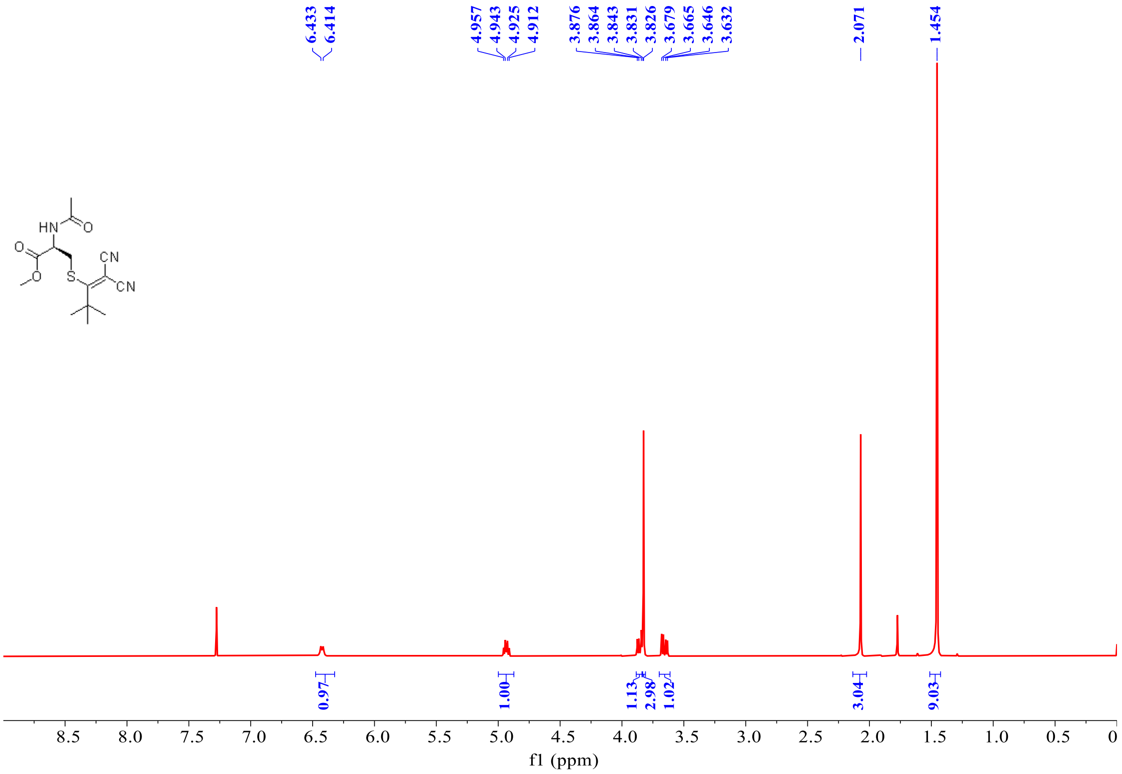
6.433
6.414
4.957
4.943
4.925
4.912
3.876
3.864
3.843
3.831
3.826
3.679
3.665
3.646
3.632
2.071
1.454
HN
O
O
O
S
CN
CN
0.97
1.00
1.13
2.98
1.02
3.04
9.03
8.5 8.0 7.5 7.0 6.5 6.0 5.5 5.0 4.5 4.0 3.5 3.0 2.5 2.0 1.5 1.0 0.5 0
f1 (ppm)

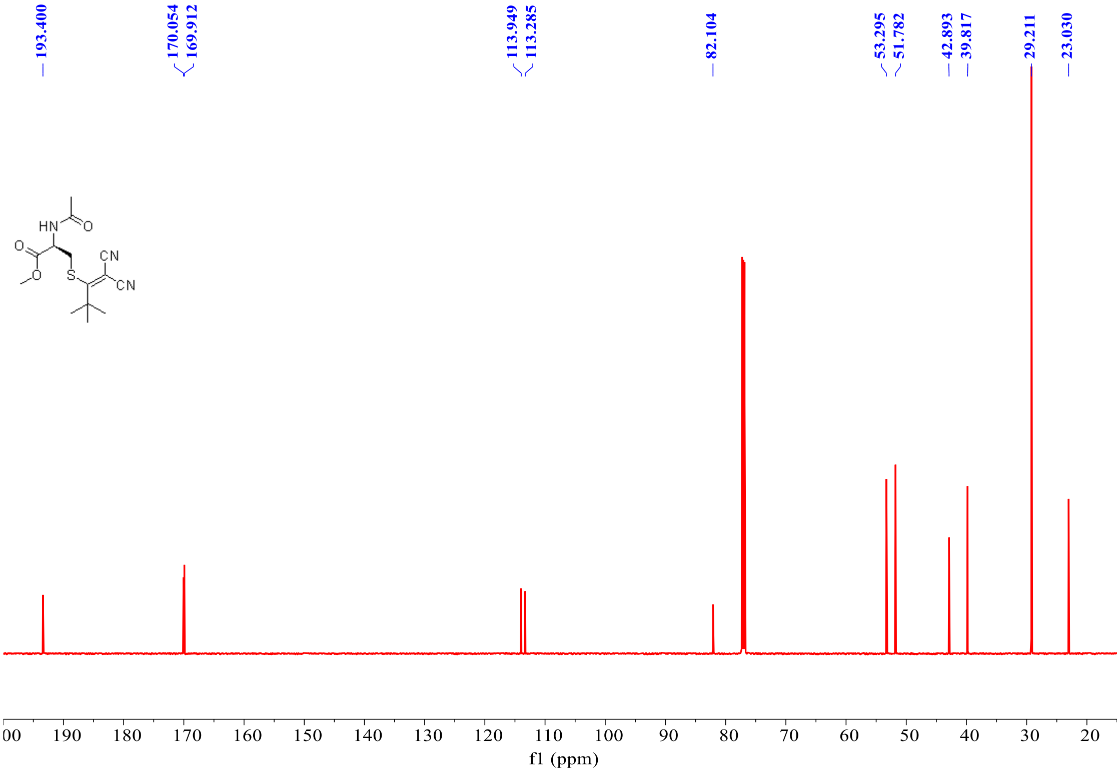

193.400
170.054
169.912
113.949
113.285
82.104
53.295
51.782
42.893
39.817
29.211
23.030
00
190
180
170
160
150
140
130
120
110
100
90
80
70
60
50
40
30
20
f1 (ppm)

Synthesis of TAMM 1k

Compound **1k'** (318 mg, 1.94 mmol, 97% yield) was obtained as a light yellow solid from silica gel column chromatography (DCM:MeOH=10:1). ^{1}H NMR (400 MHz, Methanol-d_4) δ 2.507 (s, 2H), 1.075 (s, 9H). ^{13}C NMR (151 MHz, Methanol-d_4) δ 188.621, 114.673, 112.015, 63.447, 46.695, 33.267, 28.827. ESI-(-)-HRMS $(M-H)^-$ calculated for $C_9H_{12}N_2O$: 163.08769; found: 163.08773 (-2.4 ppm). R_f (DCM:MeOH=10:1) = 0.2.

Compound **1k** (45 mg, 0.14 mmol, 14% yield) was obtained as a light yellow solid from silica gel column chromatography (EA:PE=2:1). ^{1}H NMR (400 MHz, Chloroform-*d*) δ 6.502 (d, *J* = 6.80 Hz, 1H), 4.833 – 4.792 (m, 1H), 3.807 (s, 3H), 3.665 (dd, *J* = 14.00, 5.20 Hz, 1H), 3.552 (dd, *J* = 13.60, 4.40 Hz, 1H), 2.809 – 2.700 (m, 2H), 2.042 (s, 3H), 1.077 (s, 9H). ^{13}C NMR (151 MHz, Chloroform-*d*) δ 180.690, 170.331, 169.536, 112.927, 112.031, 83.391, 53.339, 52.268, 46.906, 35.389, 35.343, 29.757, 22.953. ESI-(+)-HRMS $(M+H)^+$ calculated for $C_{15}H_{21}N_3O_3S$: 324.13764; found: 324.13724 (+1.2 ppm). Rf (EA:PE=3:1) = 0.55.

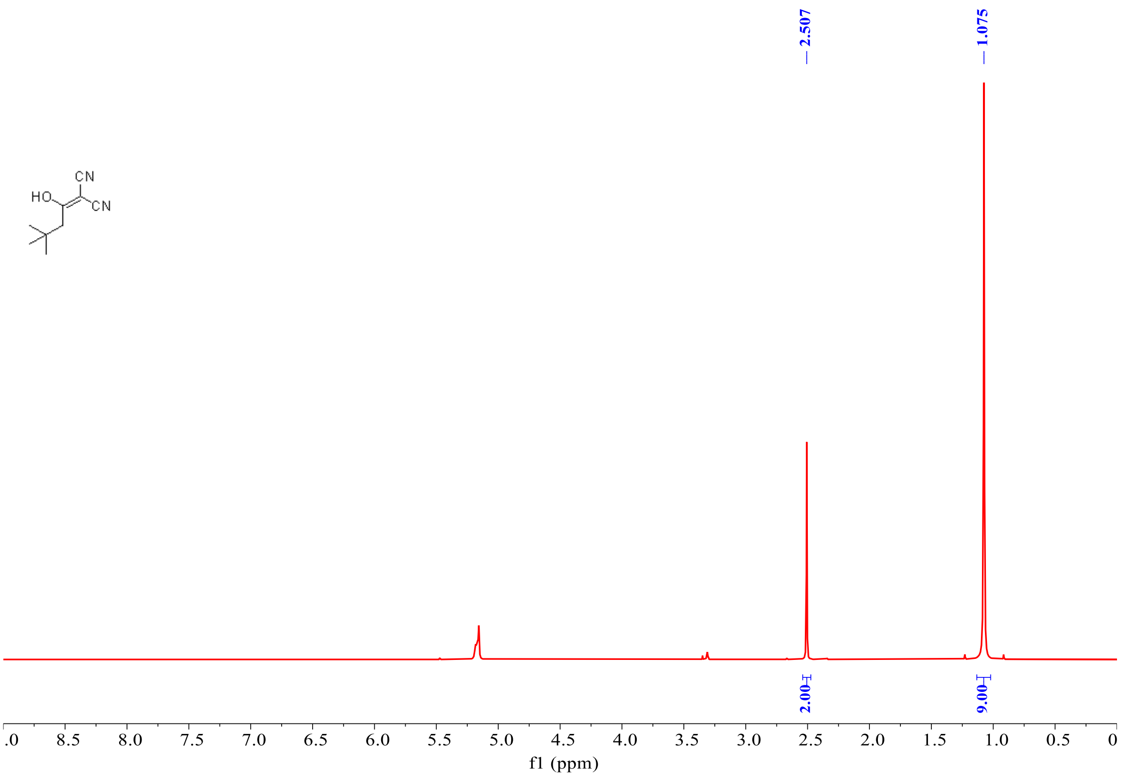

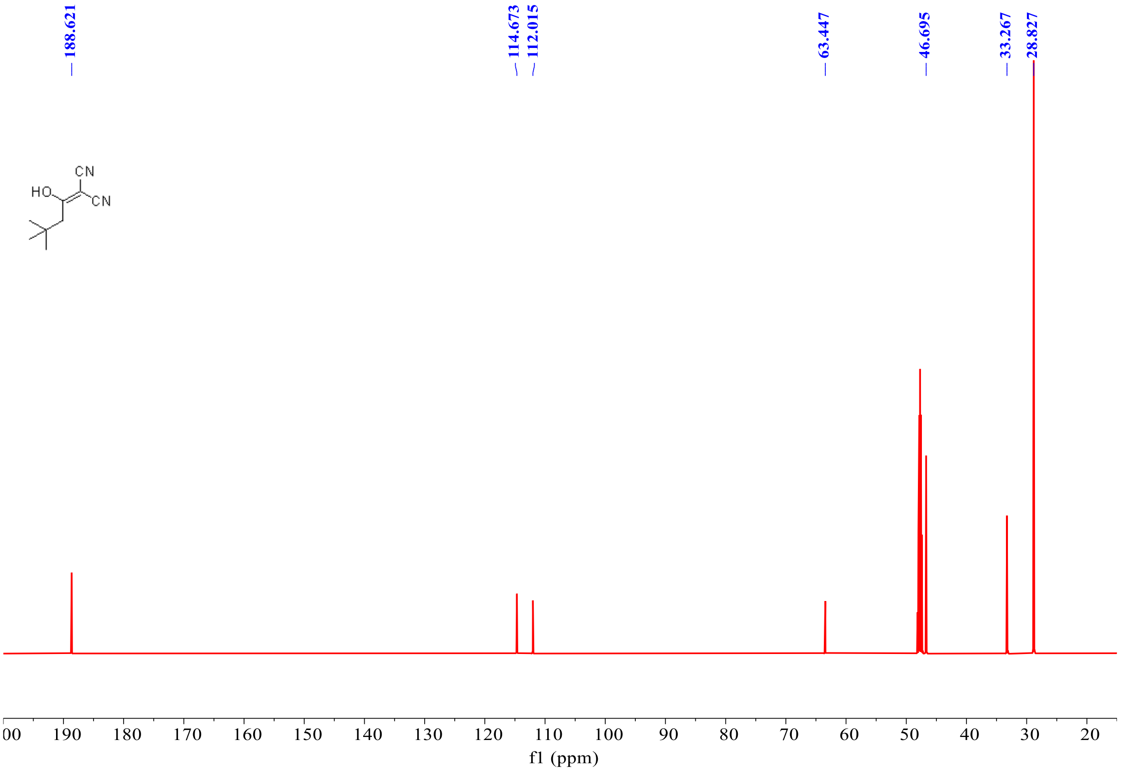
188.621
114.673
112.015
63.447
46.695
33.267
28.827
CN
HO
CN
00 190 180 170 160 150 140 130 120 110 100 90 80 70 60 50 40 30 20
f1 (ppm)

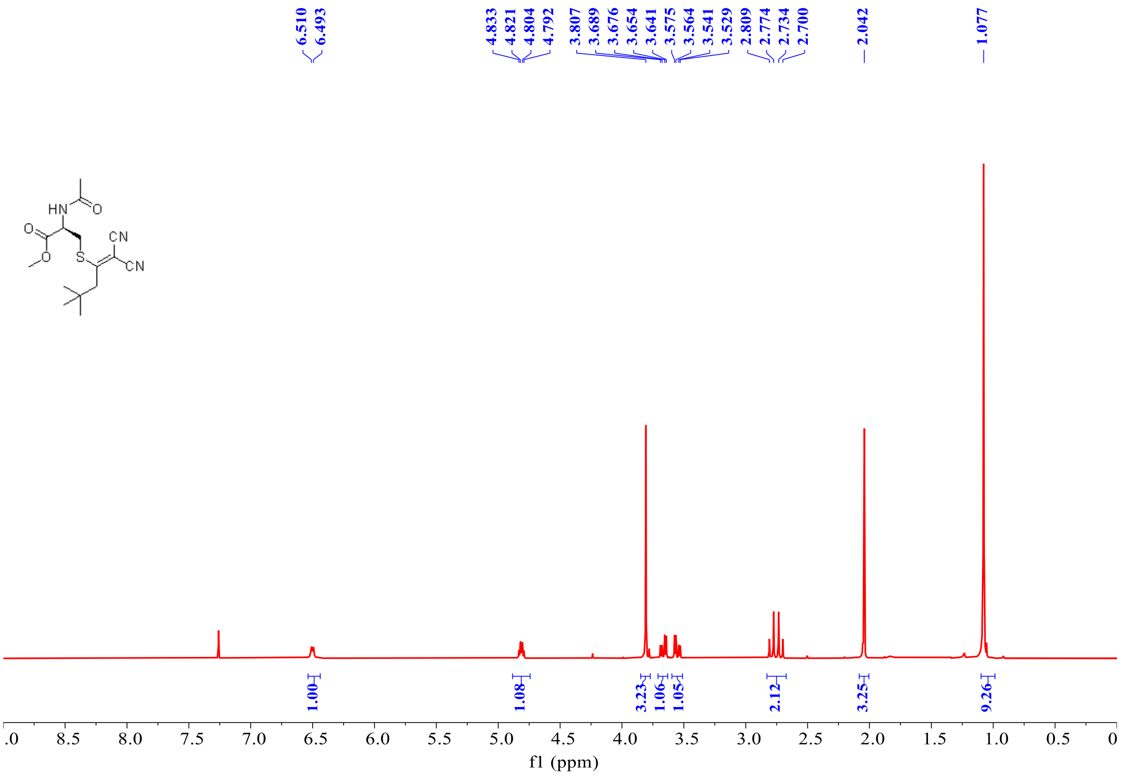
6.510
6.493
4.833
4.821
4.804
4.792
3.807
3.689
3.676
3.654
3.641
3.575
3.564
3.541
3.529
2.809
2.774
2.734
2.700
2.042
1.077
HN
O
O
O
S
CN
CN
1.00
1.08
3.23
1.06
1.05
2.12
3.25
9.26
.0 8.5 8.0 7.5 7.0 6.5 6.0 5.5 5.0 4.5 4.0 3.5 3.0 2.5 2.0 1.5 1.0 0.5 0
f1 (ppm)

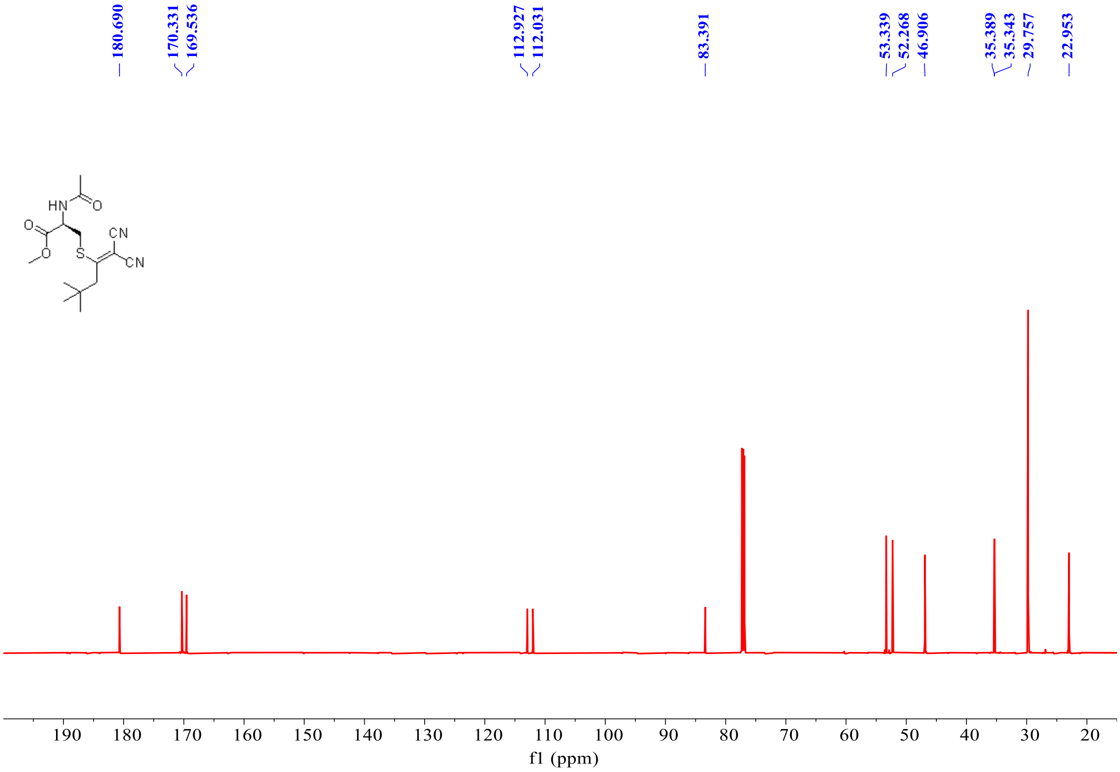
180.690
170.331
169.536
112.927
112.031
83.391
53.339
52.268
46.906
35.389
35.343
29.757
22.953
190
180
170
160
150
140
130
120
110
100
90
80
70
60
50
40
30
20
f1 (ppm)

Synthesis of TAMM 1l

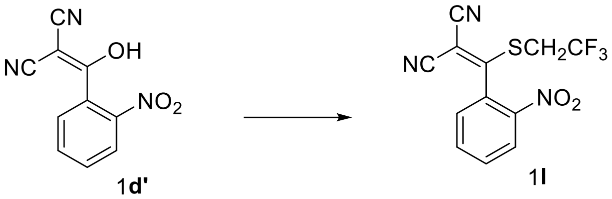

1d' 1l

To a solution of **1d'** (557 mg, 2.6 mmol, 1.0 eq) in anhydrous acetonitrile (30 mL) was added PCl_5 (1.62 g, 7.8 mmol, 3.0 eq). Under a nitrogen atmosphere, the reaction was stirred at 65 °C for 6 hours and then concentrated. The residue was dissolved in DCM (60 mL), washed with water (3 × 20 mL) and brine (20 mL), dried over Na_2SO_4, filtered and concentrated. The resulting yellow solid was then dissolved in acetonitrile (10 mL), followed by addition of 2,2,2-trifluoroethanthiol (225 μL, 2.6 mmol, 1.0 eq) and DIPEA (466 μL, 2.6 mmol, 1.0 eq). The reaction was stirred at room temperature overnight and then concentrated under reduced pressure. The crude mixture was purified by silica gel column chromatography (EA:PE=2:1) to give compound **1l** (150 mg, 0.48 mmol, 18% yield). ^{1}H NMR (400 MHz, Chloroform-d) δ 8.348 (dd, J = 8.0, 1.4 Hz, 1H), 7.924 (ddd, J = 7.8, 7.6, 1.4 Hz, 1H), 7.856 (ddd, J = 8, 7.8, 1.6 Hz, 1H), 7.438 (dd, J = 7.60, 1.60 Hz, 1H), 3.371 (dq, J = 15.4, 9.2 Hz, 1H), 3.035 (dq, J = 15.4, 9.2 Hz, 1H). ^{13}C NMR (151 MHz, Chloroform-d) δ 174.404, 146.518, 135.126, 133.506, 130.605, 126.641, 126.226, 126.042, 124.207, 122.373, 120.538, 110.845, 110.381, 84.102, 35.815, 35.584, 35.353, 35.123. ^{19}F NMR (376 MHz, Chloroform-d) δ-65.544 (t, J = 9.2 Hz). ESI-(-)-HRMS (M-H)$^-$ calculated for $C_{12}H_6F_3N_3O_2S$: 312.00601; found: 312.00587 (+0.45 ppm). R_f (EA:PE=1:3) = 0.3.

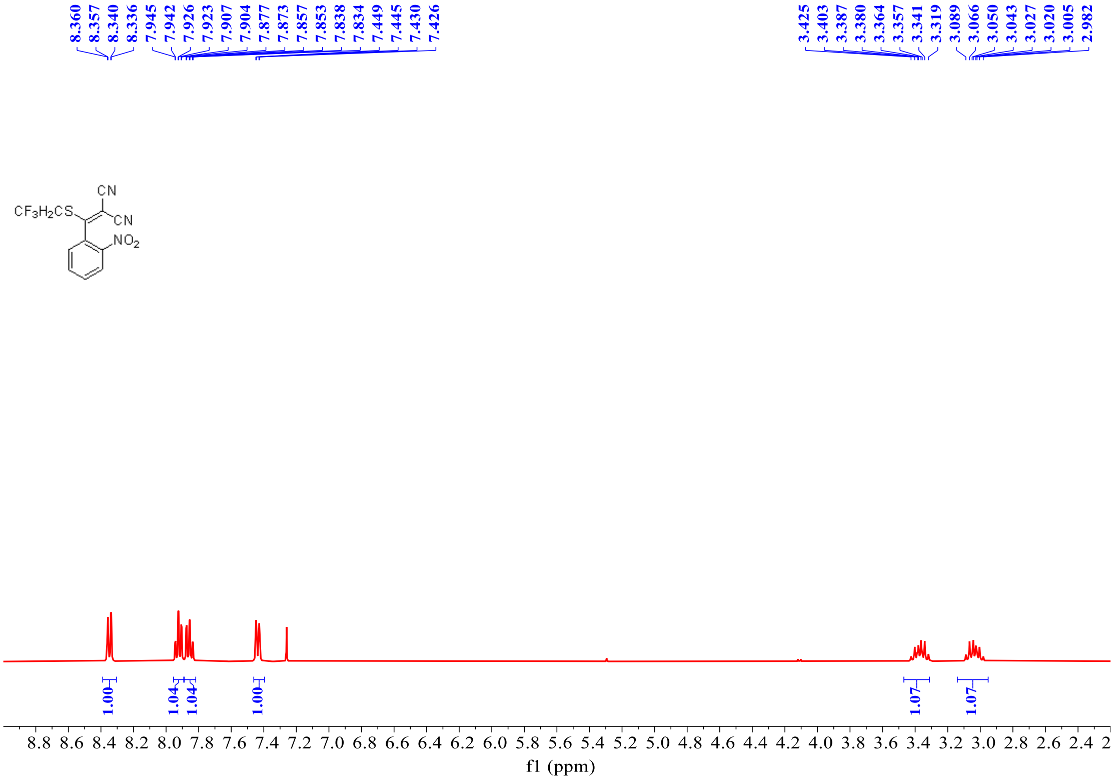

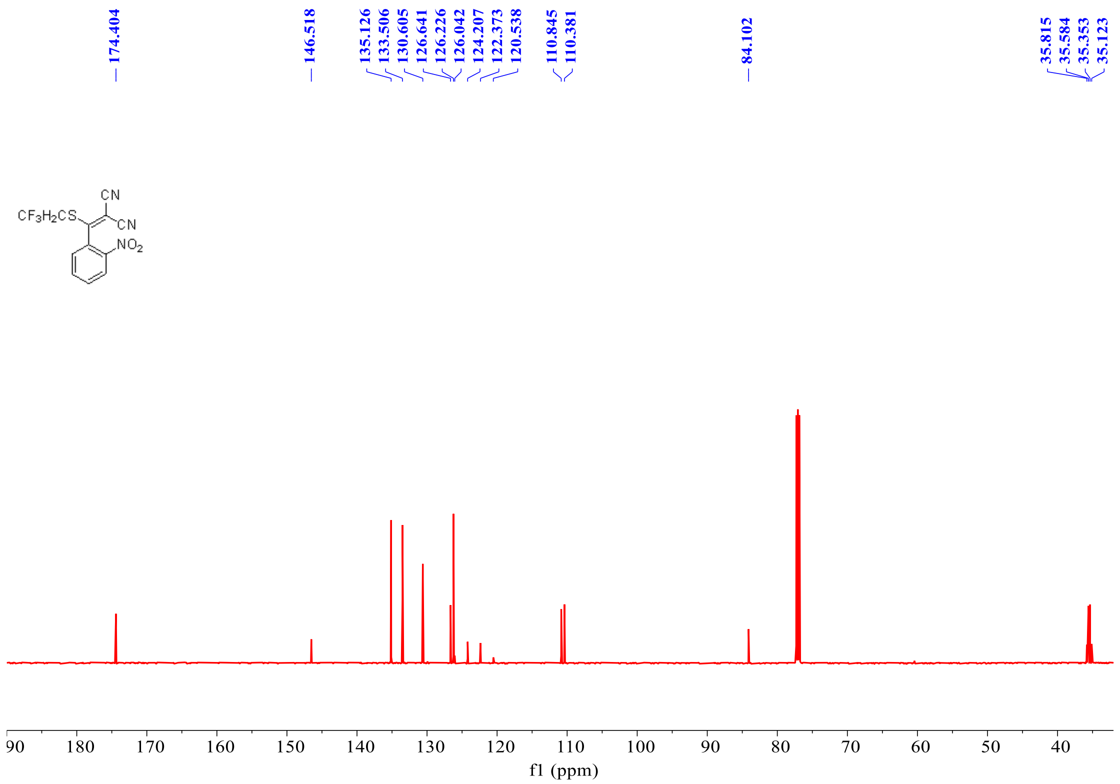
174.404
146.518
135.126
133.506
130.605
126.641
126.226
126.042
124.207
122.373
120.538
110.845
110.381
84.102
35.815
35.584
35.353
35.123
CN
CF_3H_2CS
CN
NO_2
90 180 170 160 150 140 130 120 110 100 90 80 70 60 50 40
f1 (ppm)

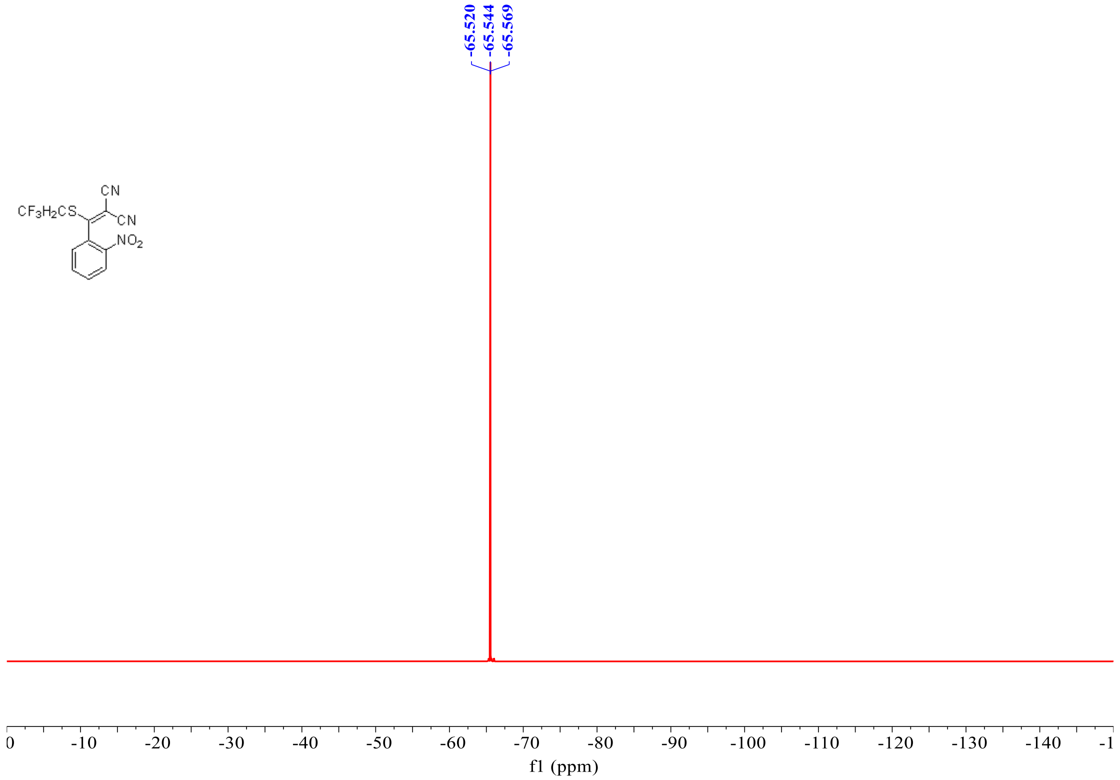
-65.520
-65.544
-65.569
CN
CF_3H_2CS
CN
NO_2
0 -10 -20 -30 -40 -50 -60 -70 -80 -90 -100 -110 -120 -130 -140 -1
f1 (ppm)

Synthesis of TAMM 1q

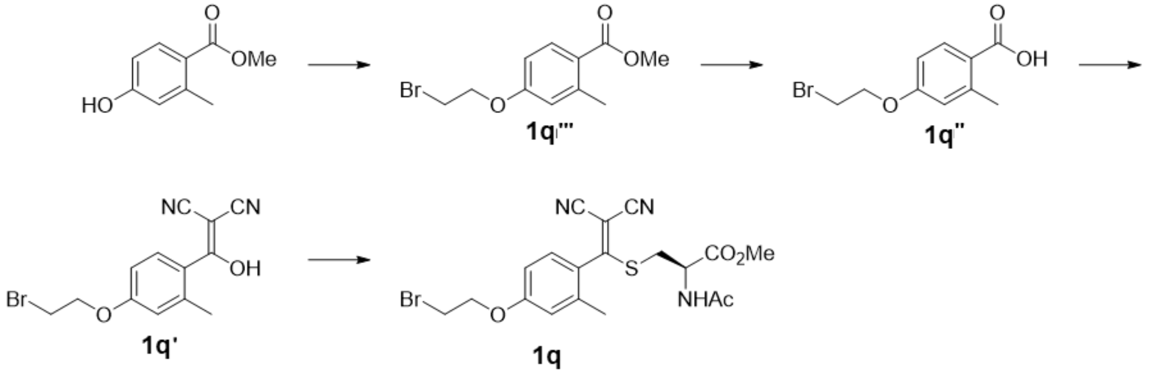

To a 2 mL acetone solution of methyl 4-hydroxy-2-methylbenzoate (1.66 g, 10 mmol, 1.0 eq) were added 1,2-dibromoethane (1.04 mL, 12 mmol, 1.2 eq) and K_2CO_3 (4.16 g, 30 mmol, 3.0 eq). The reaction mixture was refluxed for 6 hours before filtration to remove K_2CO_3. The solution was concentrated under reduced pressure before purification by silica gel column chromatography to afford **1q'''** (403 mg, 1.5 mmol, 15% yield). ^{1}H NMR (400 MHz, Chloroform-*d*) δ 7.926 (d, *J* = 8.40 Hz, 1H), 6.759-6.725 (m, 2H), 4.315 (t, *J* = 6.20 Hz, 2H), 3.855 (s, 3H), 3.637 (t, *J* = 6.20 Hz, 2H), 2.595 (s, 3H). ^{13}C NMR (101 MHz, Chloroform-*d*) δ 167.399, 160.717, 143.262, 133.032, 122.457, 117.608, 111.422, 67.667, 51.603, 28.753, 22.290. R_f (EA:PE=1:10) = 0.20.

To a 5-mL methanol solution of **1q'''** (404 mg, 1.5 mmol, 1.0 eq) was slowly added sodium hydroxide (178 mg, 4.4 mmol, 3.0 eq) aqueous solution (5 mL). The reaction mixture was refluxed and stirred at 80 °C for 3 hours. The mixture was acidified to pH 1–2 with concentrated aqueous HCl before extraction with EtOAc (3 × 100 mL). The combined organic phases were washed with brine (3 × 50 mL), dried over Na_2SO_4, filtered and concentrated. The crude mixture was purified by silica gel column chromatography (DCM:MeOH=20:1) to afford **1q''** (302 mg, 1.17 mmol, 79% yield). ^{1}H NMR (400 MHz, Chloroform-*d*) δ 7.856 (m, 1H), 6.653-6.634 (m, 2H), 4.217 (t, *J* = 6.10 Hz, 2H), 3.540 (t, *J* = 6.10 Hz, 2H), 2.482 (s, 3H).^{13}C NMR (151 MHz, Chloroform-*d*) δ 173.447, 164.775, 147.328, 137.406, 126.389, 121.509, 115.277, 71.619, 32.737, 26.095. ESI-(-)-HRMS $(M\text{-}H)^-$ calculated for $C_{10}H_{11}BrO_3$: 256.98188; found: 256.98278 (-3.5 ppm). R_f (DCM:MeOH=10:1) = 0.6.

To a mixture of **1q''** (302 mg, 1.17 mmol, 1.0 eq) and thionyl chloride (15 mL) was added DMF (50 μL) dropwise at the room temperature. After two hours, the reaction mixture was concentrated under reduced pressure to give a white solid. The white solid was then dissolved in anhydrous THF (20 mL) and added dropwise into a mixture of NaH and malononitrile. The mixture of NaH and malononitrile was prepared by dropwise addition of an anhydrous THF solution (10 mL) of malononitrile (232 mg, 3.5 mmol, 3.0 eq) into a 10-mL anhydrous THF suspension of NaH (168.5 mg, 7.0 mmol, 6.0 eq) at 0 °C, followed by one hour incubation at 0 °C. After two hours, THF was removed under reduced pressure. The mixture was acidified to pH 1–2 using concentrated $HCl_{(aq)}$. The reaction mixture was extracted with EtOAc (3 × 100 mL). The combined organic phases were washed with brine (3 × 50 mL), dried over Na_2SO_4, filtered and concentrated. The crude mixture was purified by silica gel column chromatography (DCM:MeOH=10:1) to give **1q'** (241 mg, 0.79 mmol, 67% yield). ^{1}H NMR (400 MHz, Methanol-*d*$_4$) δ 7.228 (d, *J* = 8.30 Hz, 1H), 6.811 (d, *J* = 2.40 Hz, 1H), 6.770 (dd, *J* = 8.30, 2.40 Hz, 1H), 4.289 (t, *J* = 5.60 Hz, 2H), 3.676 (t, *J* = 5.60 Hz, 2H), 2.349 (s, 3H). ^{13}C NMR (151 MHz, Methanol-*d*$_4$) δ 192.541, 159.229, 136.988, 131.837, 128.582, 116.272, 111.219, 67.921, 29.269, 18.340. ESI-(-)-HRMS $(M\text{-}H)^-$ calculated for $C_{13}H_{11}BrN_2O_2$: 304.99311; found: 304.99431 (-3.9 ppm). R_f (DCM:MeOH=1:1) = 0.2.

To a 20-mL acetonitrile solution of **1q'** (241 mg, 0.79 mmol, 1.0 eq) was added PCl_5 (492 mg, 2.4 mmol, 3.0 eq). The reaction mixture was stirred at 65 °C under a nitrogen atmosphere for six hours. The solvent was then removed under reduced pressure. The residue was dissolved in DCM (60 mL), washed with water (3 × 20 mL) and brine (20 mL), dried over Na_2SO_4, filtered, and

concentrated to give a yellow solid. To an acetonitrile solution (5 mL) of the yellow solid (32 mg, 0.1 mmol, 1.0 eq) were added Ac-Cys-OMe (18 mg, 0.1 mmol, 1.0 eq) and Et_3N (28 μL, 0.2 mmol, 2.0 eq) at 0 °C. After ten min, acetonitrile was removed under reduced pressure. The reaction mixture was purified by silica gel column chromatography (EA:PE=2:1) to give **1q** (24 mg, 0.05 mmol, 52% yield). ^{1}H NMR (400 MHz, Chloroform-*d*) δ 7.085-7.023 (m, 1H), 6.880-6.855 (m, 2H), 6.256 (br, 1H), 4.646 (m, 1H), 4.319 (t, *J* = 6.10 Hz, 2H), 3.762 (br, 3H), 3.652 (t, *J* = 6.10 Hz, 2H), 3.172-3.023 (m, 1H), 2.917 (dd, *J* = 13.20, 5.20 Hz, 1H), 2.295 (s, 3H), 2.026 (s, 3H). ^{13}C NMR (151 MHz, Chloroform-*d*) δ 181.297, 181.186, 169.986, 169.968, 169.588, 169.538, 160.428, 137.630, 137.315, 129.667, 129.553, 124.596, 117.664, 117.478, 113.246, 113.081, 112.110, 111.766, 81.052, 67.880, 53.305, 51.272, 51.210, 35.954, 28.677, 23.056, 19.409, 19.370. ESI-(+)-HRMS $(M+H)^+$ calculated for $C_{19}H_{20}BrN_3O_4S$: 466.04307; found: 466.04207 (+2.1 ppm). Rf (EA:PE=2:1) = 0.5.

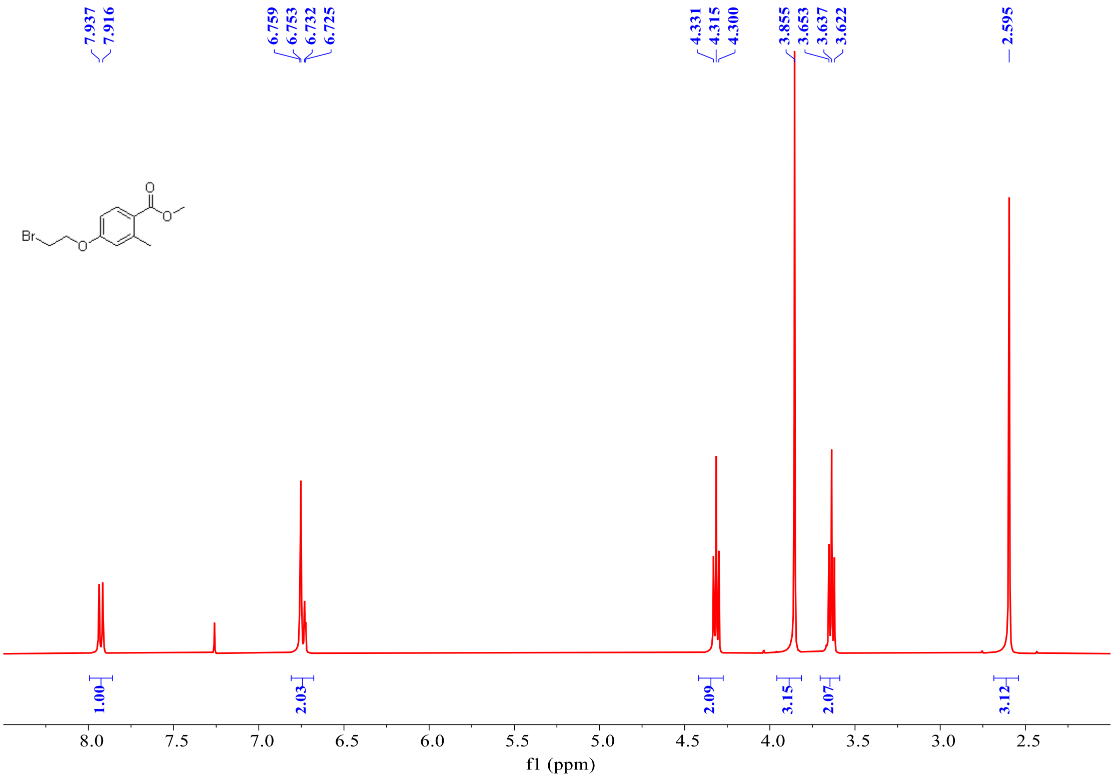

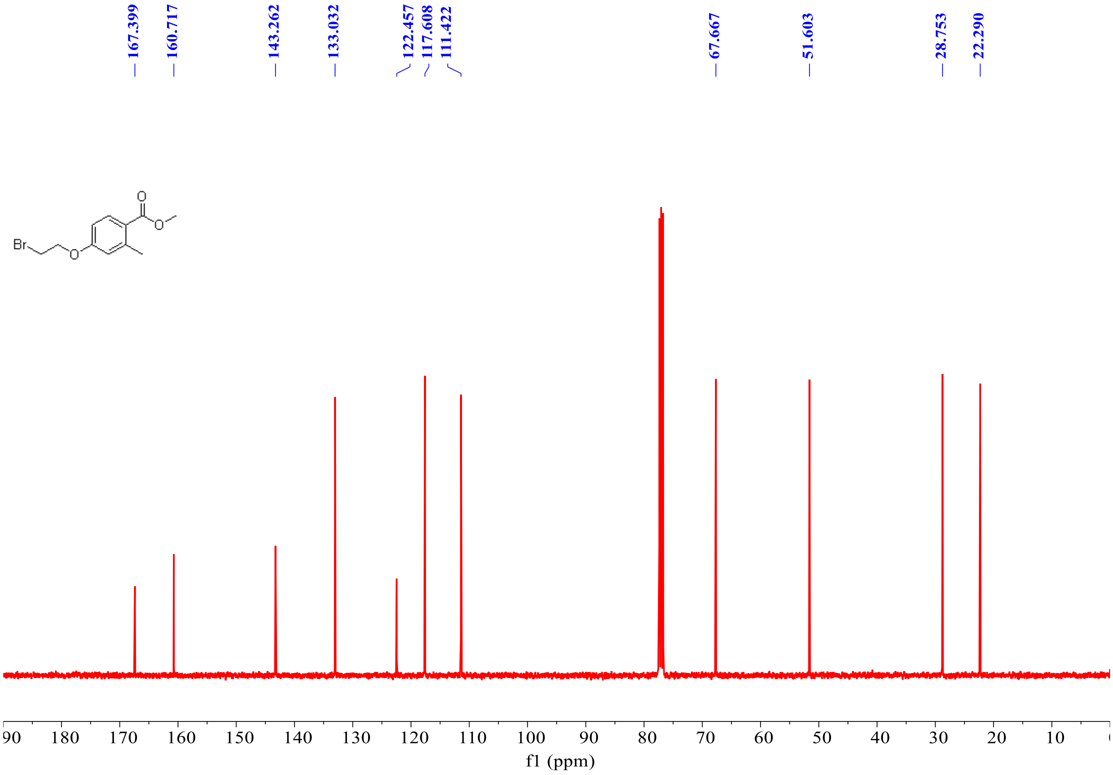
167.399
160.717
143.262
133.032
122.457
117.608
111.422
67.667
51.603
28.753
22.290
Br
O
O
O
90 180 170 160 150 140 130 120 110 100 90 80 70 60 50 40 30 20 10
f1 (ppm)

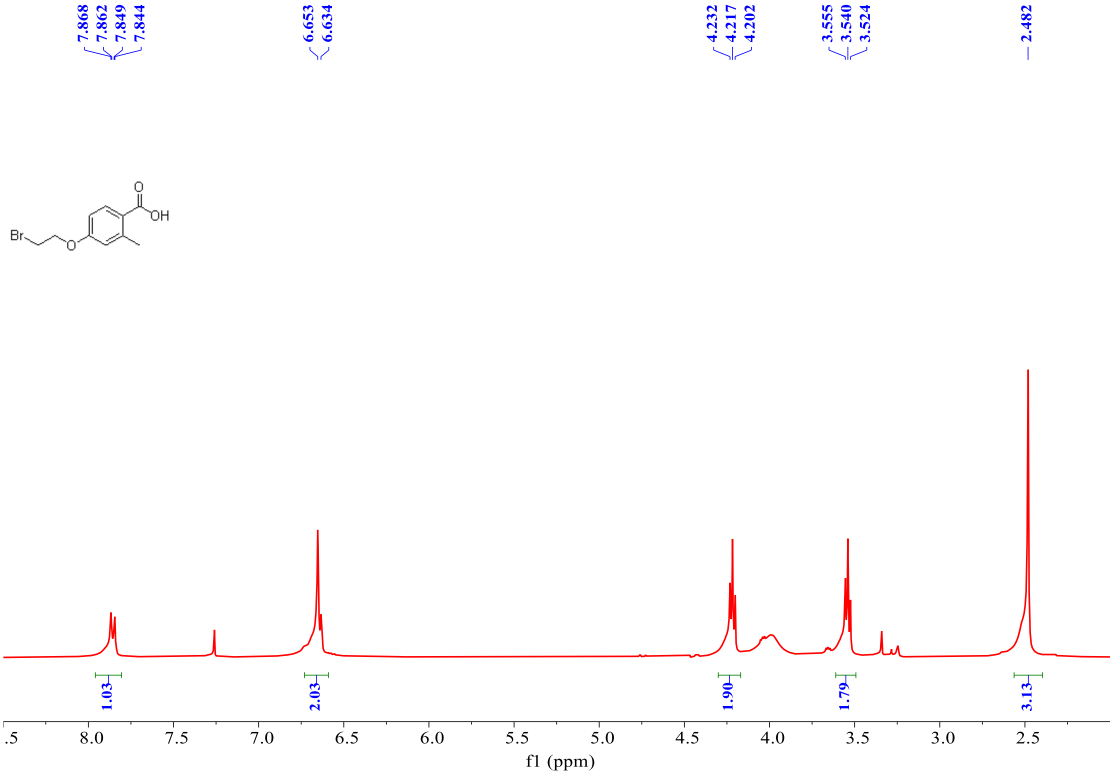
7.868
7.862
7.849
7.844
6.653
6.634
4.232
4.217
4.202
3.555
3.540
3.524
2.482
Br
O
O
OH
1.03
2.03
1.90
1.79
3.13
.5 8.0 7.5 7.0 6.5 6.0 5.5 5.0 4.5 4.0 3.5 3.0 2.5
f1 (ppm)

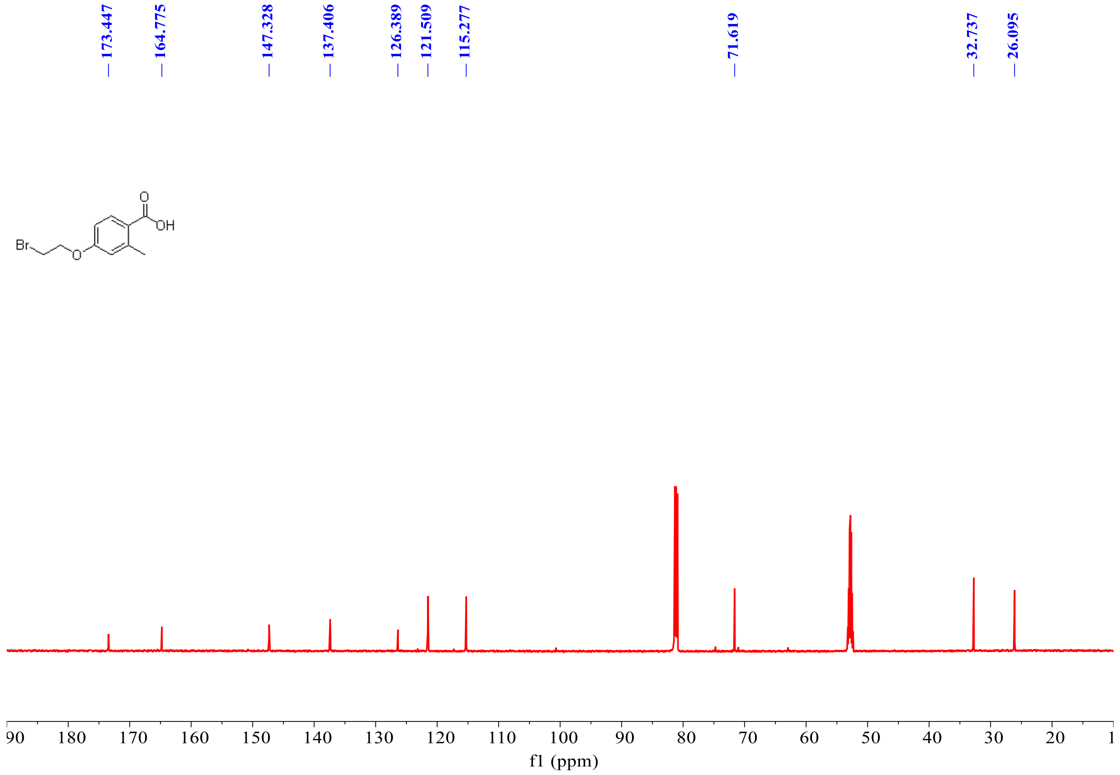

173.447
164.775
147.328
137.406
126.389
121.509
115.277
71.619
32.737
26.095
O
OH
Br
O
90
180
170
160
150
140
130
120
110
100
90
80
70
60
50
40
30
20
1
f1 (ppm)

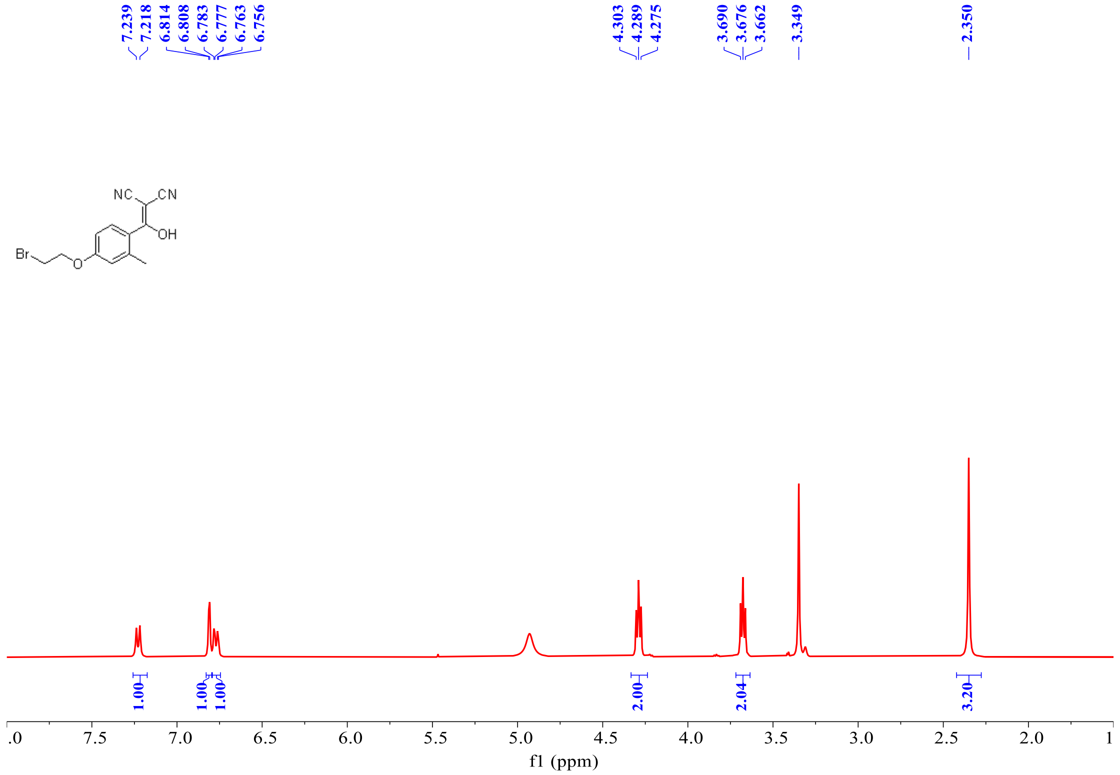

7.239
7.218
6.814
6.808
6.783
6.777
6.763
6.756
4.303
4.289
4.275
3.690
3.676
3.662
3.349
2.350
NC
CN
OH
Br
O
1.00
1.00
1.00
2.00
2.04
3.20
.0
7.5
7.0
6.5
6.0
5.5
5.0
4.5
4.0
3.5
3.0
2.5
2.0
1
f1 (ppm)

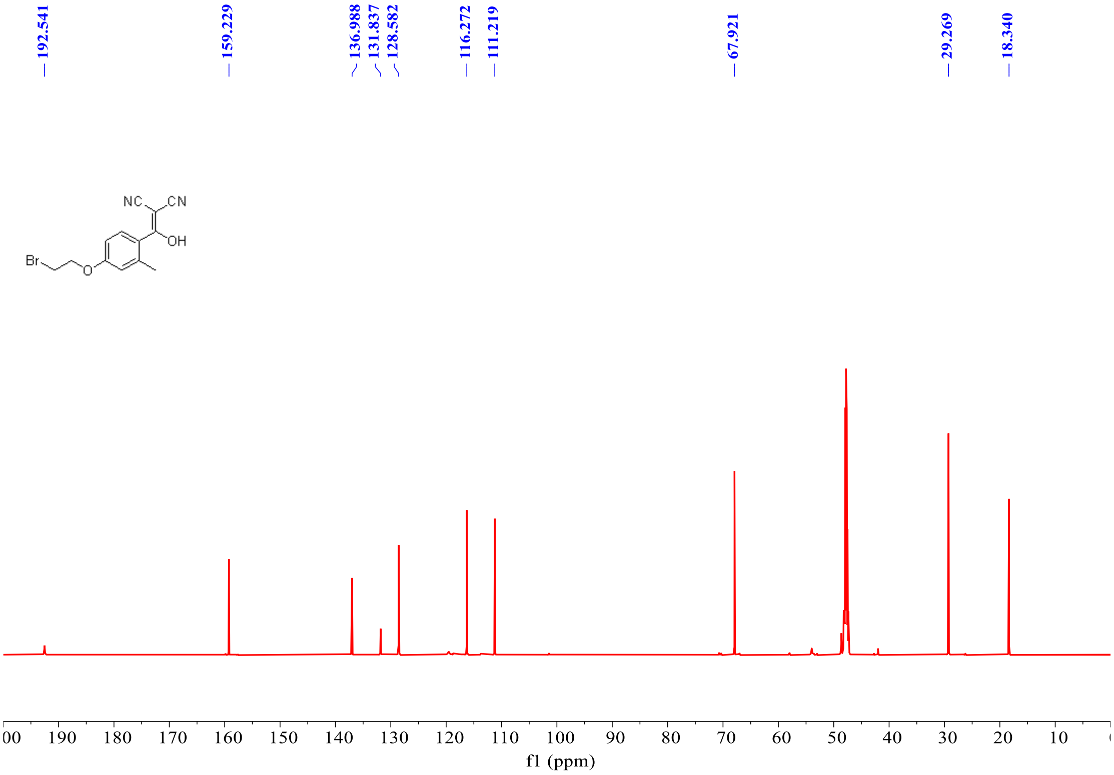
192.541
159.229
136.988
131.837
128.582
116.272
111.219
67.921
29.269
18.340
NC
CN
OH
Br
O
f1 (ppm)

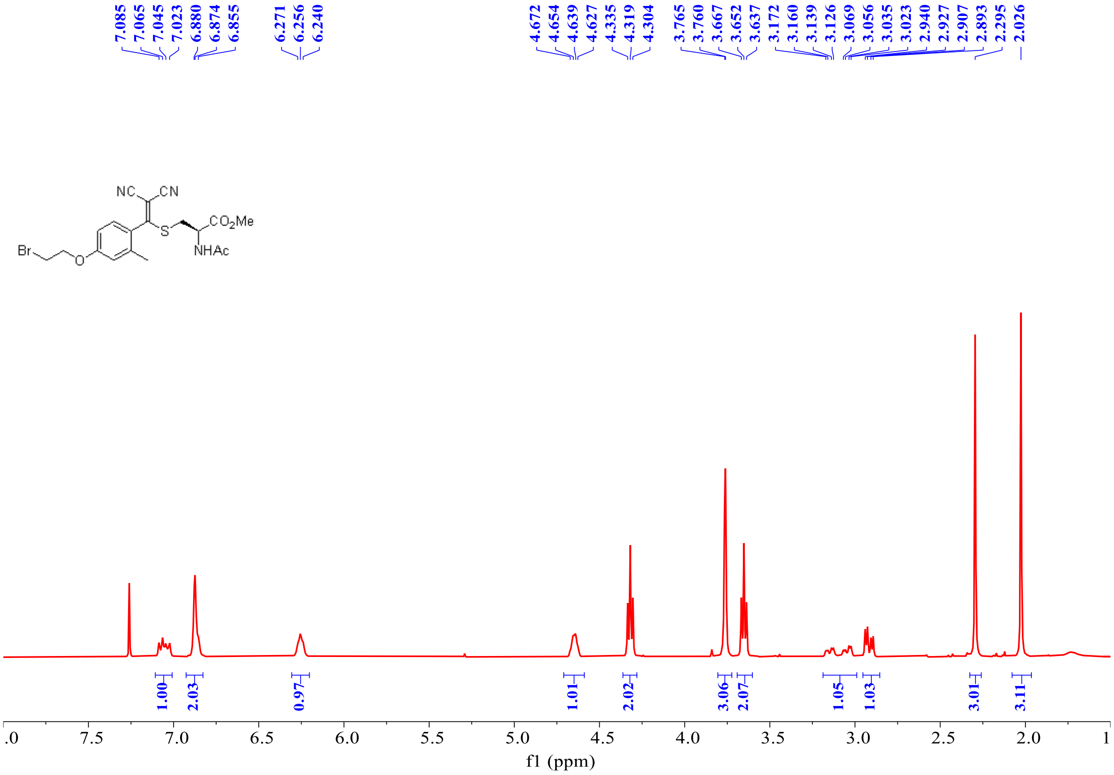
7.085
7.065
7.045
7.023
6.880
6.874
6.855
6.271
6.256
6.240
4.672
4.654
4.639
4.627
4.335
4.319
4.304
3.765
3.760
3.667
3.652
3.637
3.172
3.160
3.139
3.126
3.069
3.056
3.035
3.023
2.940
2.927
2.907
2.893
2.295
2.026
NC
CN
S
CO2Me
NHAc
Br
O
1.00
2.03
0.97
1.01
2.02
3.06
2.07
1.05
1.03
3.01
3.11
f1 (ppm)

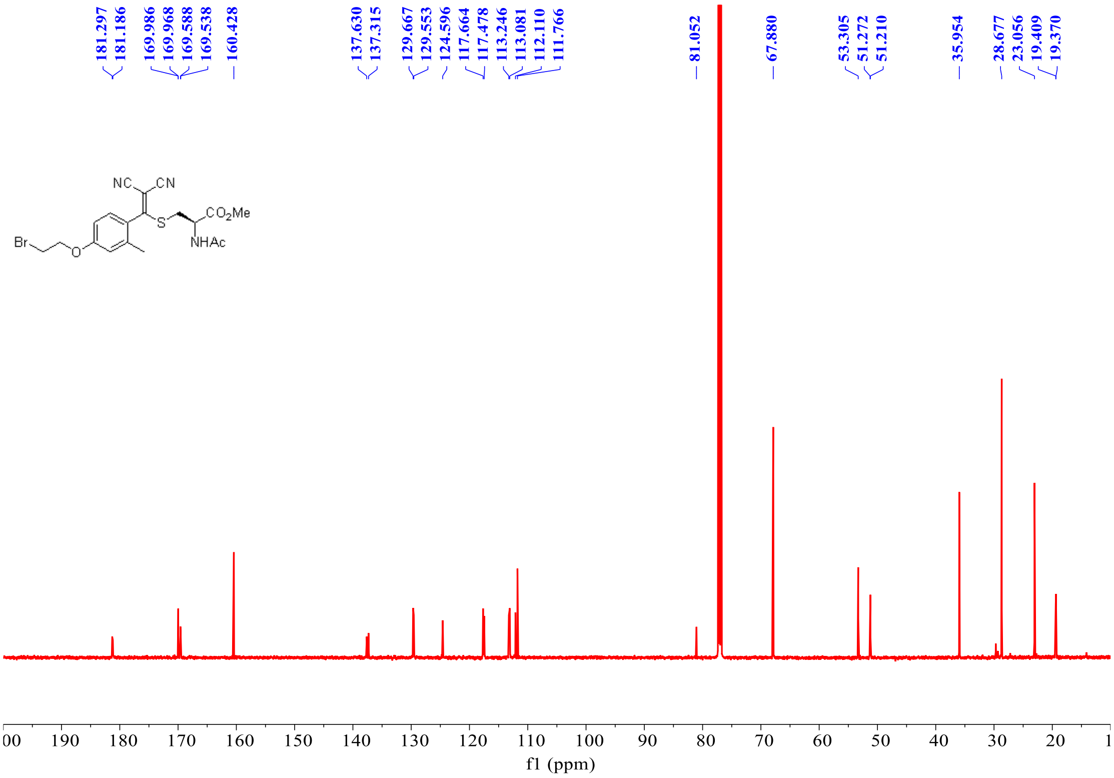
181.297
181.186
169.986
169.968
169.588
169.538
160.428
137.630
137.315
129.667
129.553
124.596
117.664
117.478
113.246
113.081
112.110
111.766
81.052
67.880
53.305
51.272
51.210
35.954
28.677
23.056
19.409
19.370
NC
CN
S
CO2Me
NHAc
Br
O
f1 (ppm)

Synthesis of TAMM 1p

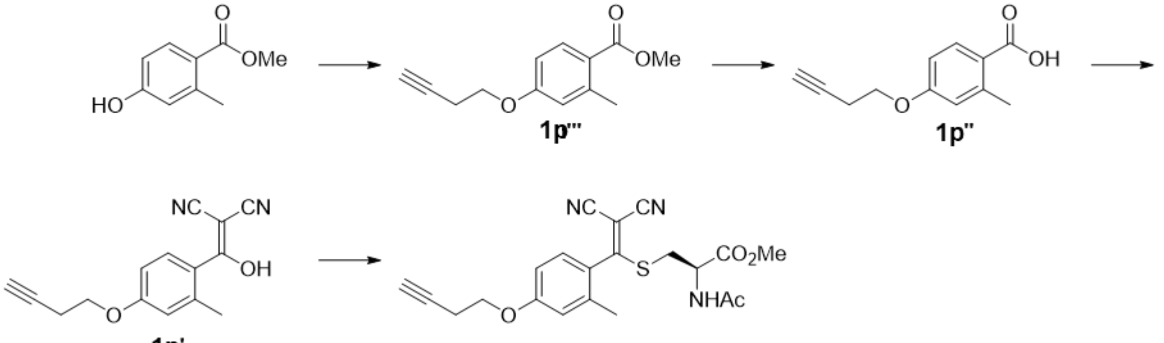

To a solution of methyl 4-hydroxy-2-methylbenzoate (166 mg,1.0 mmol, 1.0 eq), K_2CO_3 (414 mg, 3.0 mmol, 3.0 eq) in acetone (20 mL) was added but-3-yn-1-yl 4-methylbenzenesulfonate (530 μL, 3.0 mmol, 3.0 eq). After 24 hours at 80 °C, acetone was then removed under reduced pressure. The residue was purified by flash chromatography (SiO_2, 20% EtOAc in PE) to yield **1p'''** as a yellow powder (72 mg, 0.33 mmol, 33%). ^1H NMR (600 MHz, Chloroform-*d*) δ 7.92 (d, *J* = 8.4 Hz, 1H), 6.74 – 6.73 (m, 2H), 4.1 (t, *J* = 7.1 Hz, 2H), 3.85 (s, 3H), 2.68 (td, *J* = 7.1, 2.6 Hz, 2H), 2.59 (s, 3H), 2.0 (t, *J* = 2.6 Hz, 1H). ^{13}C NMR (151 MHz, Chloroform-*d*) δ 167.6, 161.2, 143.3, 133.1, 122.2, 117.6, 111.5, 80.2, 70.2, 66.0, 51.7, 22.4, 19.6. ESI-(+)-HRMS $(M+H)^+$ calculated for $C_{13}H_{15}O_3$: 219.1016; found:219.1016. R_f (EA:PE=1:10) = 0.20.

To a solution of **1p'''** (44 mg, 0.2 mmol, 1.0 eq) in MeOH (3 mL) was added a solution of NaOH (24 mg, 0.6 mmol, 3.0 eq) in water (3 mL). After two hours at 80 °C, complete conversion was confirmed by TLC. MeOH was then removed under reduced pressure. Then the mixture was acidified to pH 1–2 using 1 N $HCl_{(aq)}$. The mixture was extracted with EtOAc (3 ×20 mL). The combined organic phases were washed with brine (20 mL), dried over Na_2SO_4, filtered, and concentrated to **1p''** as a white solid (39 mg, 0.19 mmol, 95%). ^1H NMR (400 MHz, Methanol-*d4*) δ 7.92 (d, *J* = 9.60 Hz, 1H), 6.80 – 6.78 (m, 2H), 4.12 (t, *J* = 6.65 Hz, 2H), 2.66 (td, *J* = 6.7, 2.6 Hz, 2H), 2.57 (s, 3H), 2.34 (t, *J* = 2.6 Hz, 1H). ^{13}C NMR (151 MHz, Methanol-*d4*) δ 169.2, 161.5, 142.9, 133.0, 122.1, 117.7, 111.1, 79.9, 69.6, 65.9, 21.1, 18.7. ESI-(-)-HRMS $(M-H)^-$ calculated for $C_{12}H_{11}O_3$: 203.0714; found: 203.0714. Rf (DCM:MeOH=10:1) = 0.6.

To a solution of **1p''** (30 mg, 0.15 mmol, 1.0 eq) in $SOCl_2$ (2 mL) was added DMF (5 μL). After 1 hour at room temperature, $SOCl_2$ was removed under reduced pressure to yield 4-(but-3-yn-1-yloxy)-2-methylbenzoyl chloride as a colorless oil. The colorless oil was then dissolved in anhydrous THF (2 mL) and added dropwise into a mixture of NaH and malononitrile. The mixture of NaH and malononitrile was prepared by dropwise addition of an anhydrous THF solution (3 mL) of malononitrile (20 mg, 0.3 mmol, 2.0 eq) into a 10-mL anhydrous THF suspension of NaH (144 mg, 6.0 mmol, 2.0 eq) at 0 °C, followed by one hour incubation at 0 °C. After two hours, THF was removed under reduced pressure. The mixture was acidified to pH 1–2 using concentrated $HCl_{(aq)}$. The reaction mixture was extracted with EtOAc (3 × 100 mL). The combined organic phases were washed with brine (3 × 50 mL), dried over Na_2SO_4, filtered and concentrated. The crude mixture was purified by silica gel column chromatography (DCM:MeOH=10:1) to give **1p'** as a light-yellow solid (25 mg, 0.1 mmol, 68%). ^1H NMR (400 MHz, Methanol-*d4*) δ 7.30 (d, *J* = 8.4 Hz, 1H), 6.89 (s, 1H), 6.85 (d, *J* = 8.4 Hz, 1H), 4.12 (t, *J* = 6.7 Hz, 2H), 2.66 (td, *J* = 6.7, 2.7 Hz, 2H), 2.36 (s, 3H), 2.33 (t, *J* = 2.7 Hz, 1H). ^{13}C NMR (151 MHz, Methanol-*d4*) δ 162.0, 139.1, 130.7, 128.5, 117.8, 112.9, 81.3, 71.0, 67.4, 20.1, 19.5. ESI-(-)-HRMS $(M-H)^-$ calculated for $C_{15}H_{11}N_2O_2$: 251.0826; found: 251.0829. R_f (DCM:MeOH=10:1) = 0.2.

To a solution of **1p'** (25 mg, 0.1 mmol, 1.0 eq) in acetonitrile (6 mL) was added PCl_5 (62 mg, 0.3 mmol, 3.0 eq). After five hours at 60 ℃ under an argon atmosphere, the mixture was concentrated. The residue was dissolved in DCM (20 mL), washed with water (3 × 10 mL) and brine (3 × 10 mL), dried over Na_2SO_4, filtered, and concentrated. The resulting yellow solid was then dissolved in

acetonitrile (3 mL), followed by the addition of N-acetyl-L-cysteine methyl ester (35 mg, 0.2 mmol, 2.0 eq) and $NaHCO_3$ (25 mg, 0.3 mmol, 3.0 eq) were added. The reaction was stirred at room temperature for two hours. The acetonitrile was removed under reduced pressure. Brine (15 mL) was added. The mixture was extracted with EtOAc (3 × 20 mL). The combined organic phases were washed with brine (20 mL), dried over Na_2SO_4, filtered, and concentrated. The residue was purified by flash chromatography (SiO_2, 10% MeOH in EtOAc) to yield **1p** as an oil (9 mg, 0.023 mmol, 23%). ^{1}H NMR (400 MHz, Chloroform-*d*) δ 7.04 (m, 1H), 6.86 (s, 1H), 6.84 (s, 1H), 6.45 (d, *J* = 8.0 Hz, 1H), 4.92 – 4.46 (m, 1H), 4.10 (t, *J* = 6.8 Hz, 2H), 3.73 (s, 3H), 3.23 – 2.66 (m, 3H), 2.70 – 2.66 (m, 2H), 2.28 (s, 3H), 2.5 (m, 1H), 2.01 (s, 3H). ^{13}C NMR (101 MHz, Methanol-d_4) δ 181.7, 170.4, 169.7, 160.9, 137.6, 129.7, 124.3, 117.6, 113.2, 112.2, 111.9, 80.1, 70.3, 66.1, 53.3, 51.3, 36.0, 26.9, 23.1, 19.5, 19.4. ESI-(-)-HRMS $(M-H)^-$ calculated for $C_{21}H_{21}N_3O_4S$: 412.1326; found: 412.1314. Rf (PE: EA=1:1) = 0.3.

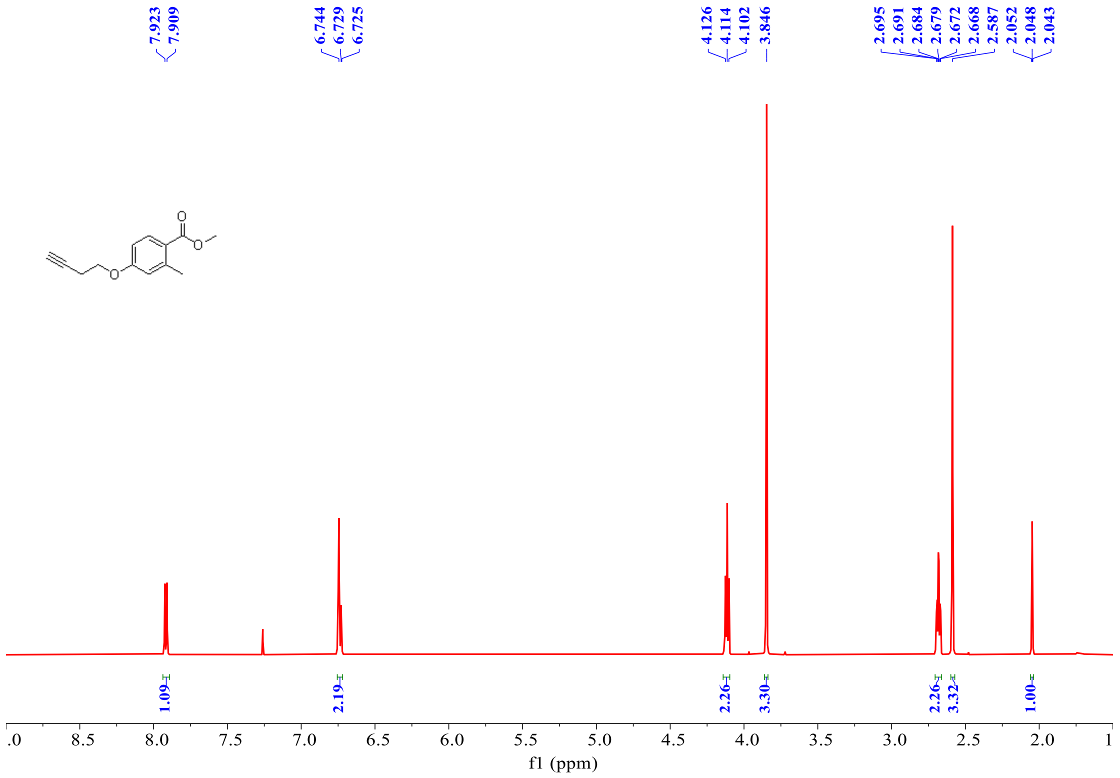

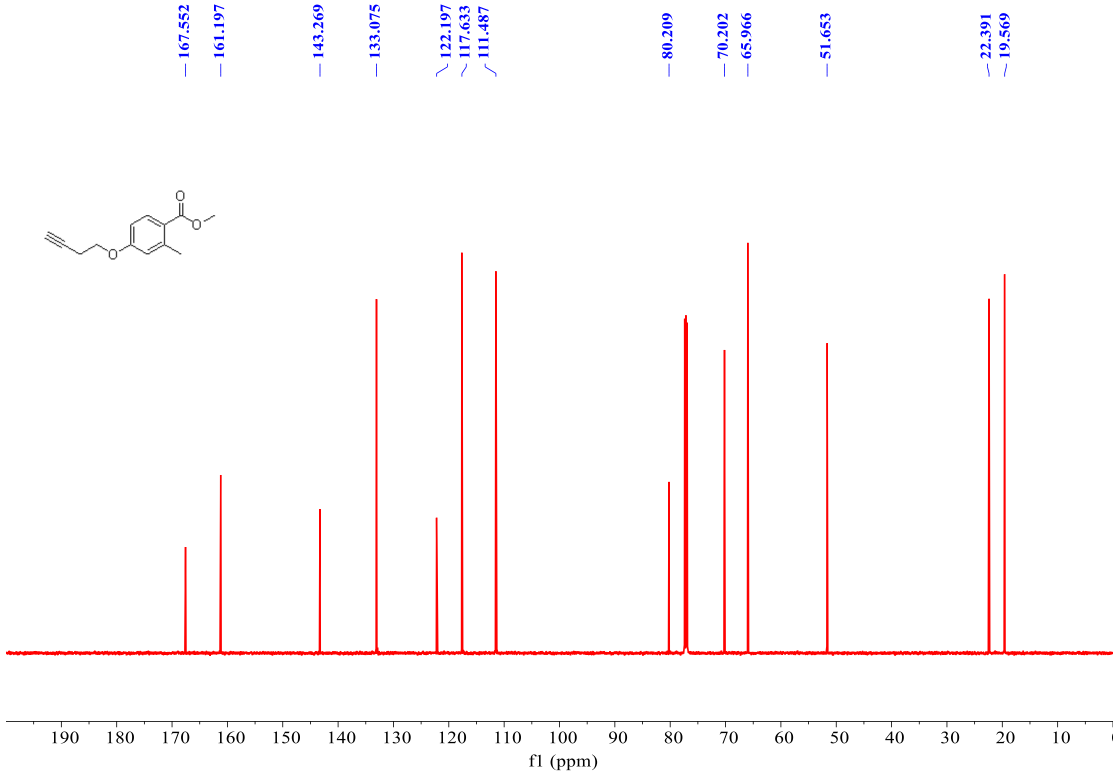
167.552
161.197
143.269
133.075
122.197
117.633
111.487
80.209
70.202
65.966
51.653
22.391
19.569
O
O
O
190 180 170 160 150 140 130 120 110 100 90 80 70 60 50 40 30 20 10
f1 (ppm)

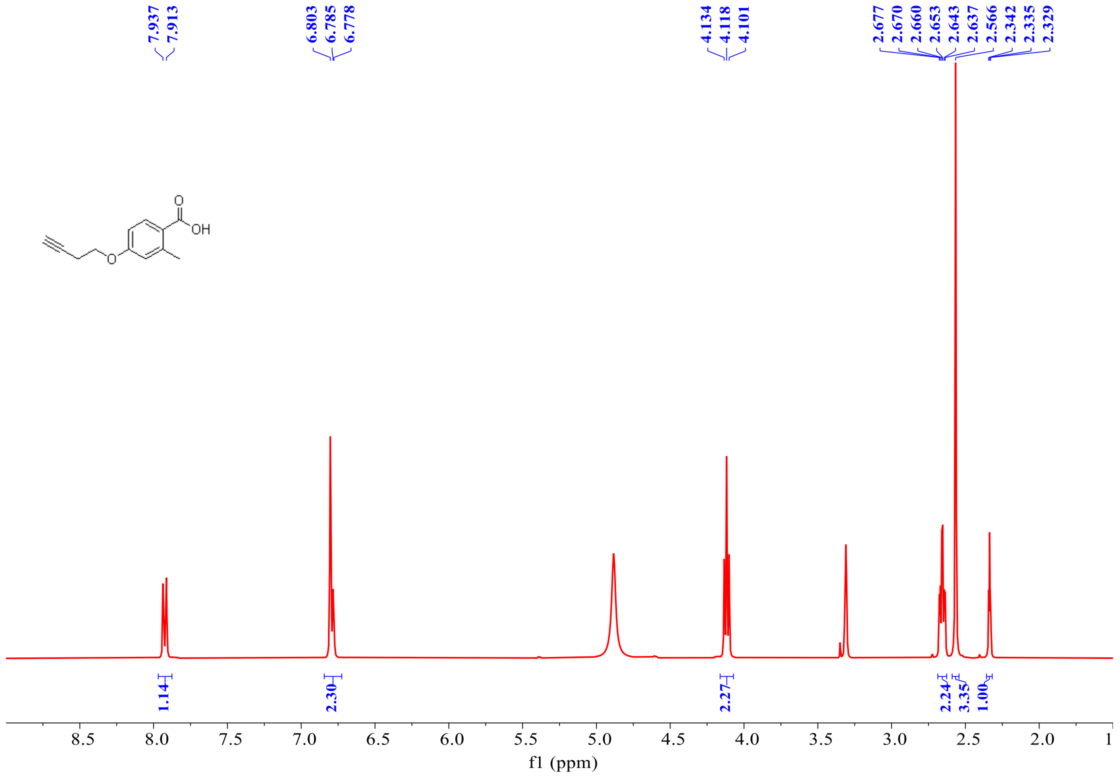
7.937
7.913
6.803
6.785
6.778
4.134
4.118
4.101
2.677
2.670
2.660
2.653
2.643
2.637
2.566
2.342
2.335
2.329
O
OH
O
1.14
2.30
2.27
2.24
3.35
1.00
8.5 8.0 7.5 7.0 6.5 6.0 5.5 5.0 4.5 4.0 3.5 3.0 2.5 2.0 1
f1 (ppm)

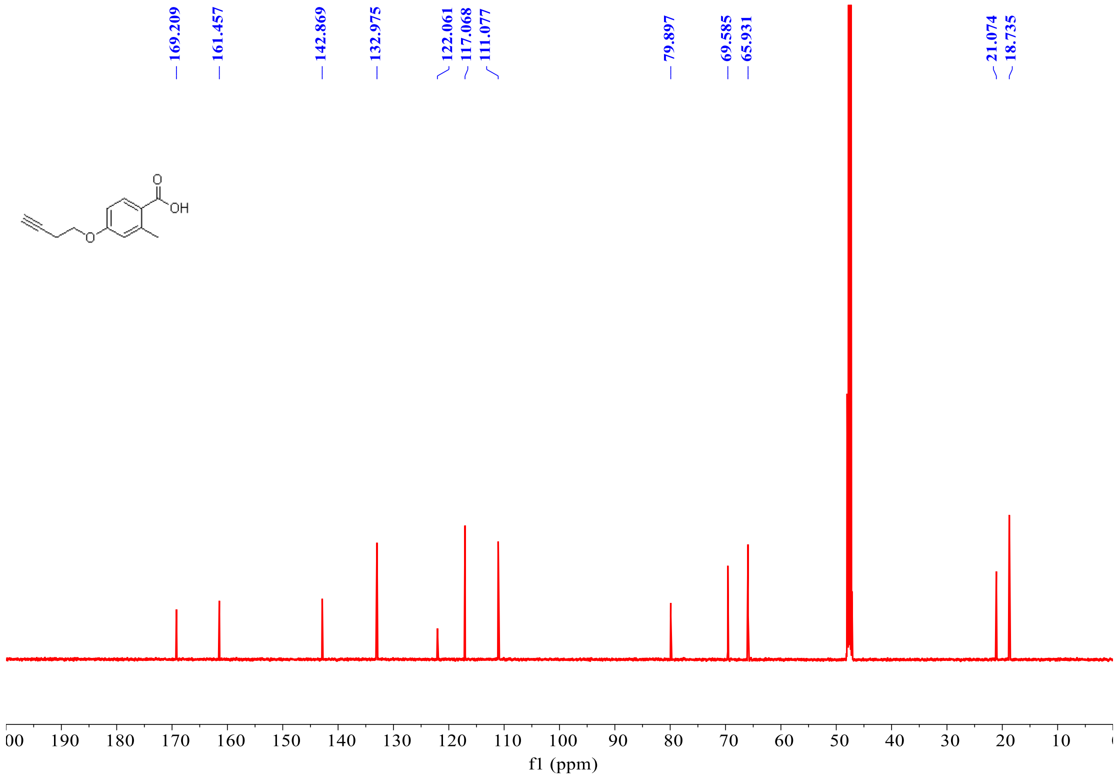
169.209
161.457
142.869
132.975
122.061
117.068
111.077
79.897
69.585
65.931
21.074
18.735
O
OH
O
f1 (ppm)

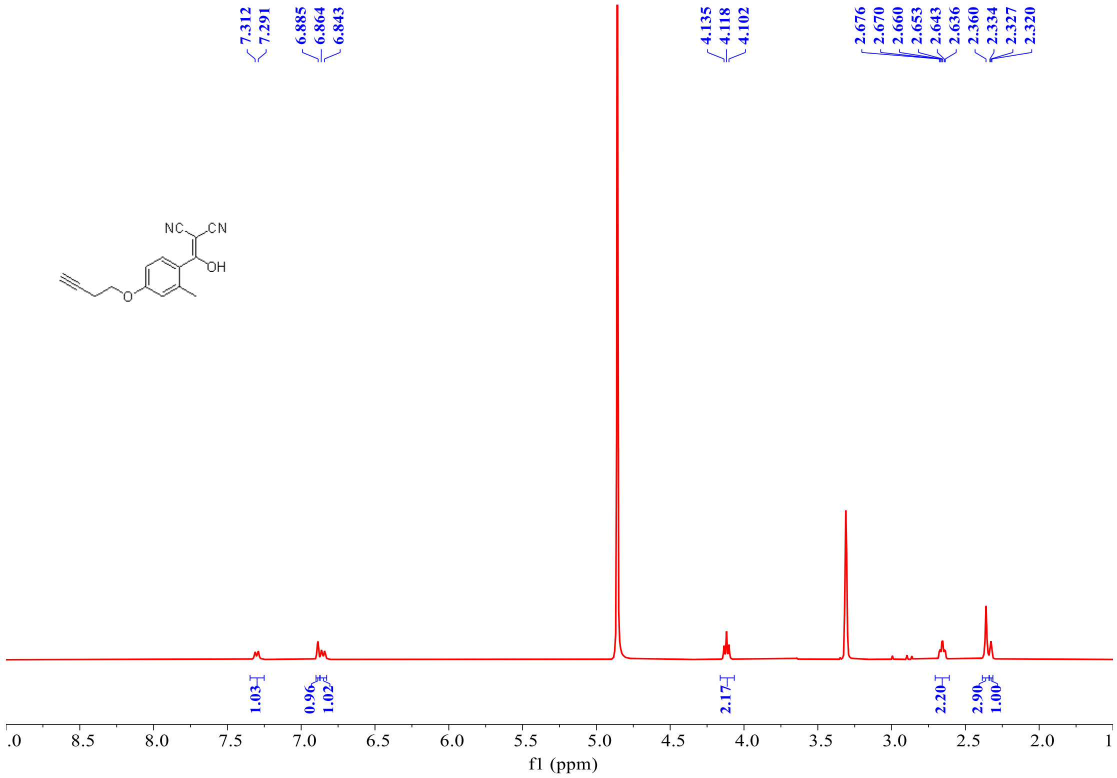
7.312
7.291
6.885
6.864
6.843
4.135
4.118
4.102
2.676
2.670
2.660
2.653
2.643
2.636
2.360
2.334
2.327
2.320
NC
CN
OH
O
1.03
0.96
1.02
2.17
2.20
2.90
1.00
f1 (ppm)

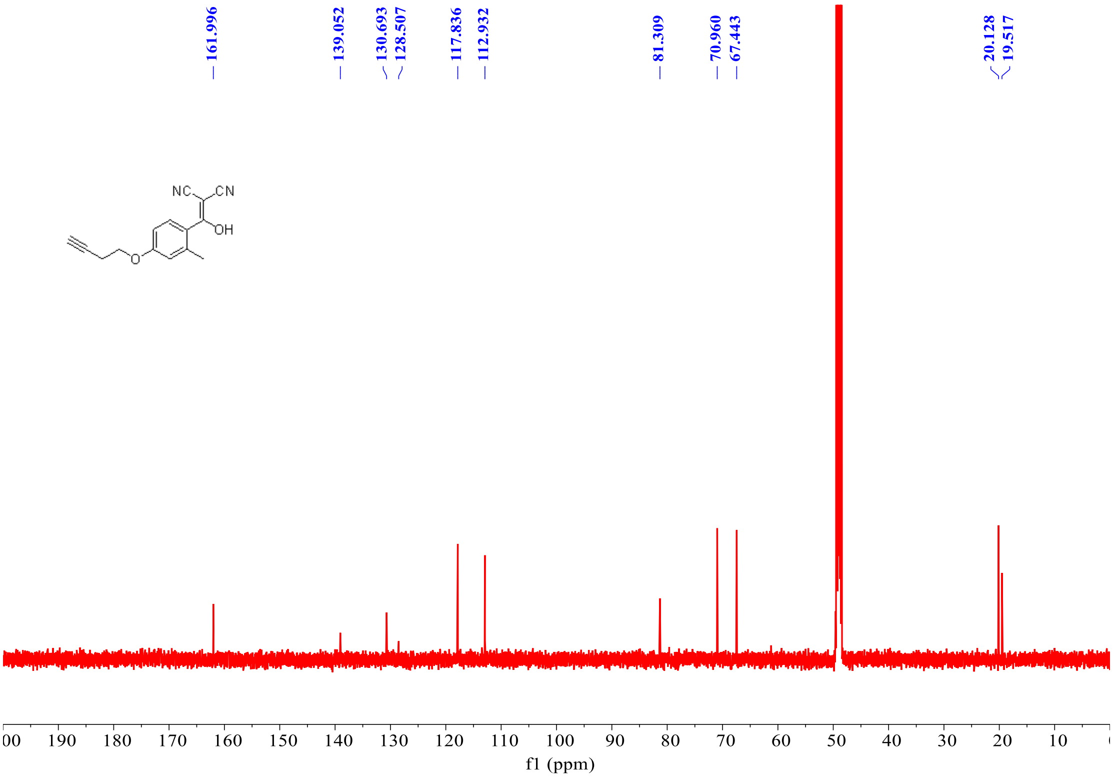
161.996
139.052
130.693
128.507
117.836
112.932
81.309
70.960
67.443
20.128
19.517
NC
CN
OH
O
200 190 180 170 160 150 140 130 120 110 100 90 80 70 60 50 40 30 20 10
f1 (ppm)

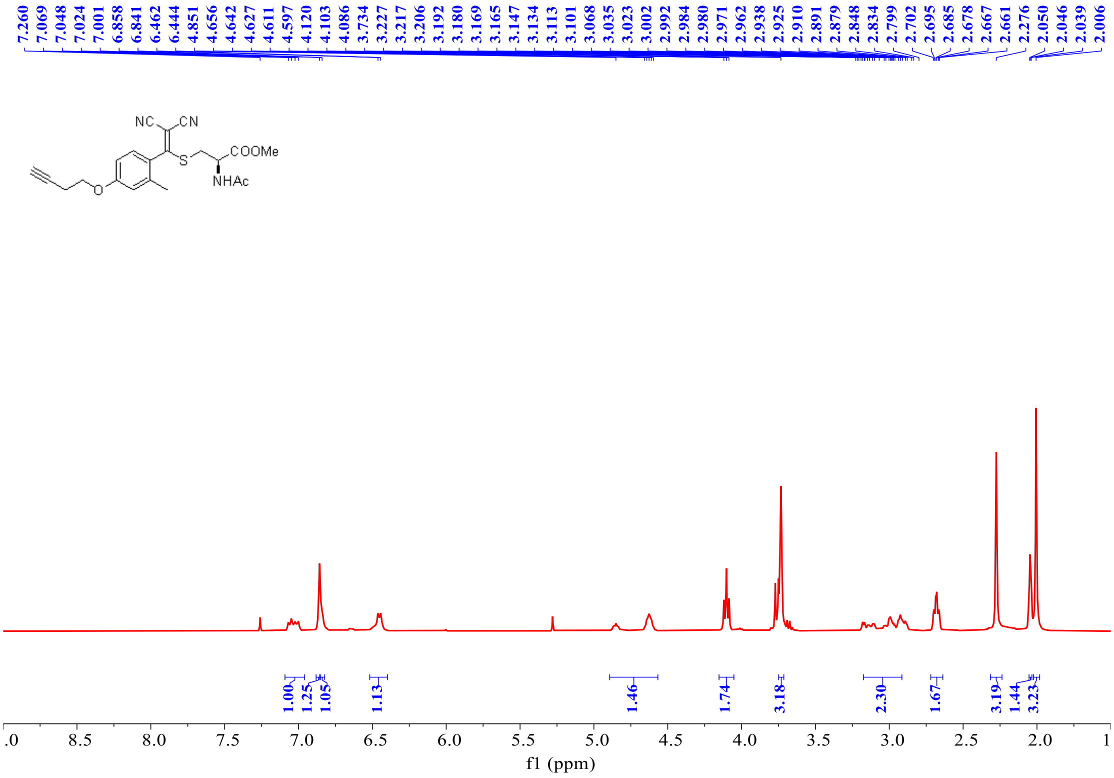
7.260 7.069 7.048 7.024 7.001 6.858 6.841 6.462 6.444 4.851 4.656 4.642 4.627 4.611 4.597 4.120 4.103 4.086 3.734 3.227 3.217 3.206 3.192 3.180 3.169 3.165 3.147 3.134 3.113 3.101 3.068 3.035 3.023 3.002 2.992 2.984 2.980 2.971 2.962 2.938 2.925 2.910 2.891 2.879 2.848 2.834 2.799 2.702 2.695 2.685 2.678 2.667 2.661 2.276 2.050 2.046 2.039 2.006
NC
CN
S
COOMe
NHAc
O
1.00
1.25
1.05
1.13
1.46
1.74
3.18
2.30
1.67
3.19
1.44
3.23
8.5 8.0 7.5 7.0 6.5 6.0 5.5 5.0 4.5 4.0 3.5 3.0 2.5 2.0
f1 (ppm)

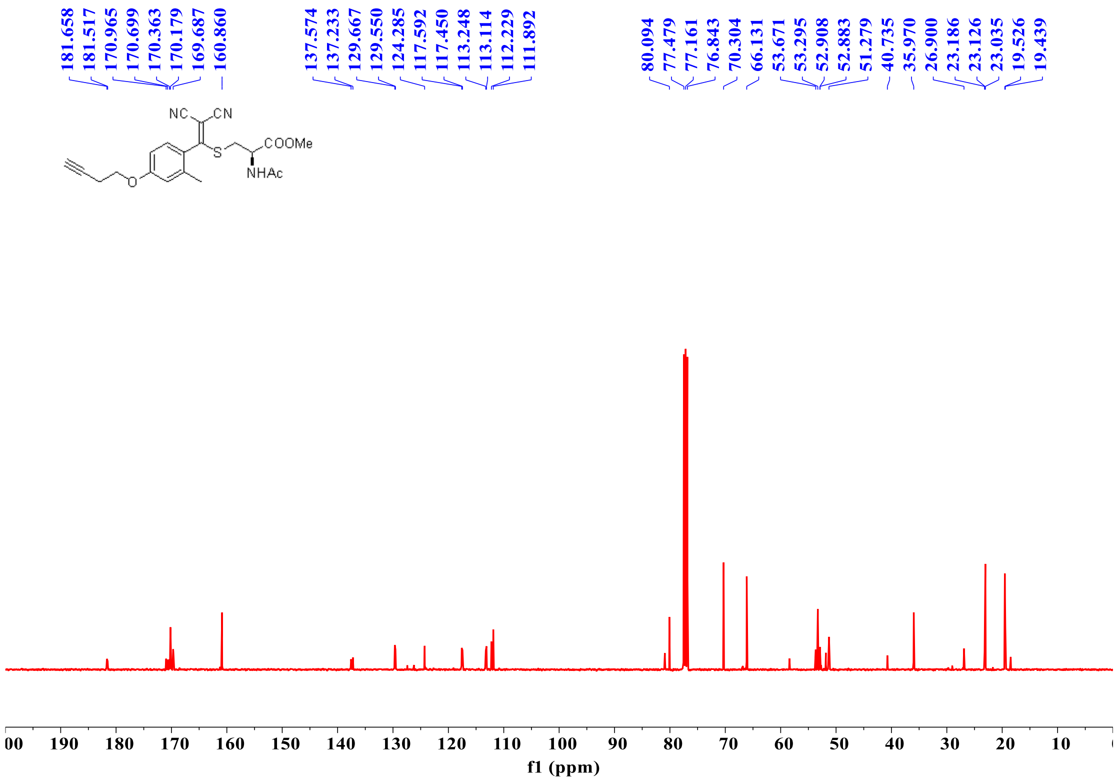
181.658
181.517
170.965
170.699
170.363
170.179
169.687
160.860
137.574
137.233
129.667
129.550
124.285
117.592
117.450
113.248
113.114
112.229
111.892
80.094
77.479
77.161
76.843
70.304
66.131
53.671
53.295
52.908
52.883
51.279
40.735
35.970
26.900
23.186
23.126
23.035
19.526
19.439
NC
CN
S
COOMe
NHAc
O
f1 (ppm)

Synthesis of TAMM 1m

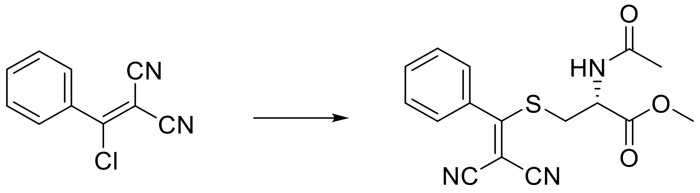

1m' **1m**

To a solution of **1m'** (35 mg, 0.19 mmol, 1.0 eq) in acetonitrile was added N-acetyl-L-cysteine methyl ester (39 mg, 0.22 mmol, 1.2 eq) and $NaHCO_3$ (50 mg, 0.60 mmol, 3.0 eq). The reaction was stirred at room temperature overnight and then concentrated under reduced pressure. The residue was purified by flash chromatography (SiO_2, 50% PE in EtOAc) to yield **1m** as an oil (53 mg, 0.16 mmol, 84%). ^{1}H NMR (600 MHz, Chloroform-*d*) δ 7.61 – 7.55 (m, 3H), 7.45 – 7.44 (m, 2H), 6.22 (d, *J* = 6.6 Hz, 1H), 4.64 (dt, *J* = 6.6, 5.1 Hz, 1H), 3.77 (s, 3H), 3.25 (dd, *J* = 13.8, 5.1 Hz, 1H), 3.05 (dd, *J* = 13.8, 5.1 Hz, 1H), 2.03 (s, 3H). ^{13}C NMR (151 MHz, Chloroform-*d*) δ 179.9, 170.0, 169.7, 133.0, 132.1, 129.80, 129.0, 112.6, 112.2, 81.3, 53.4, 51.8, 36.6, 23.2. Rf (PE: EA=1:2) = 0.3.

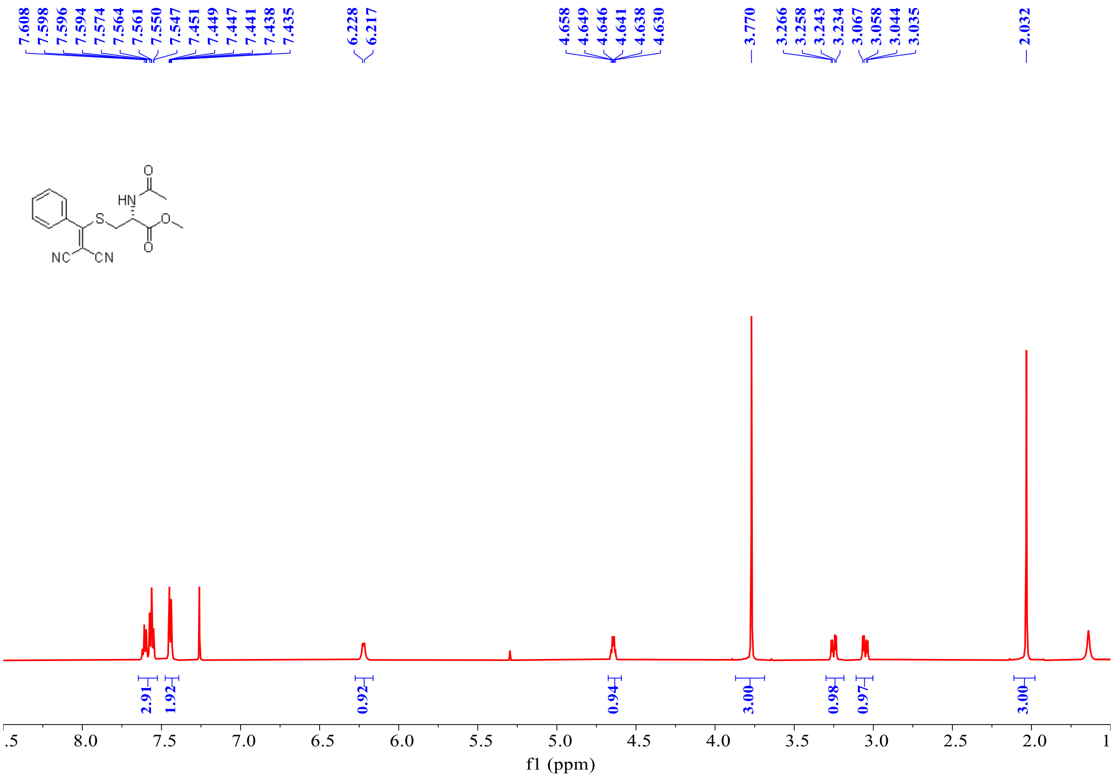

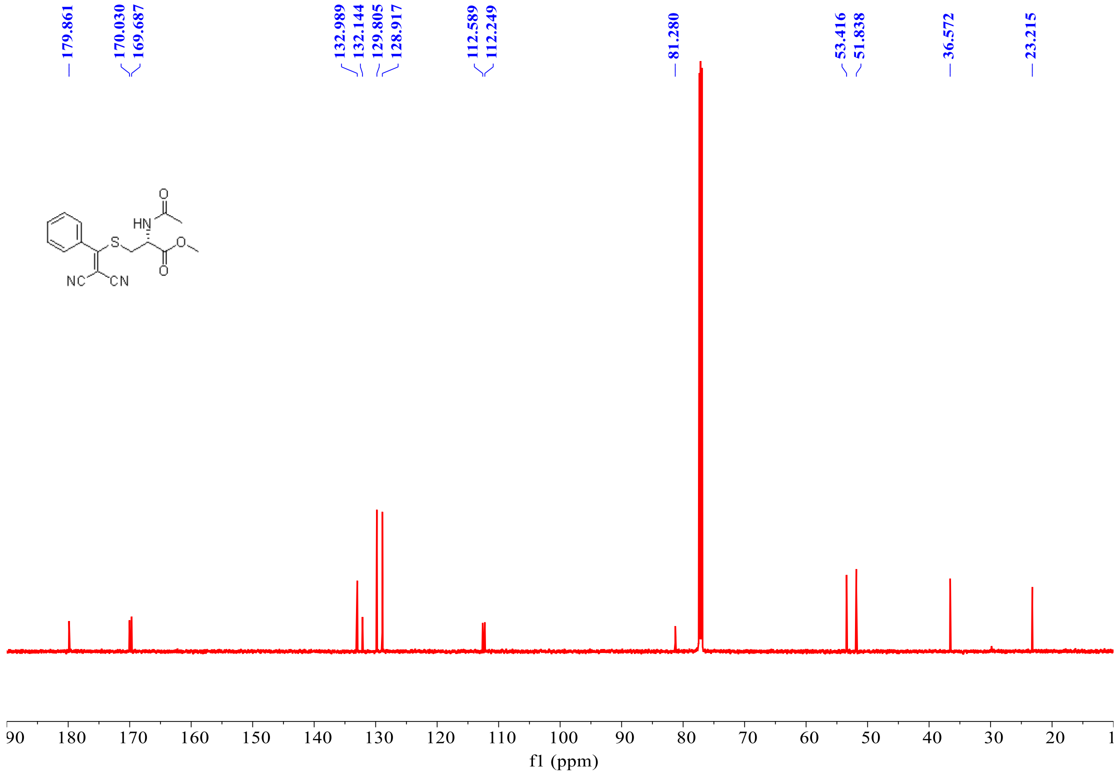
179.861
170.030
169.687
132.989
132.144
129.805
128.917
112.589
112.249
81.280
53.416
51.838
36.572
23.215
HN
O
S
O
O
NC
CN
90 180 170 160 150 140 130 120 110 100 90 80 70 60 50 40 30 20 1
f1 (ppm)

Synthesis of TAMM 1n

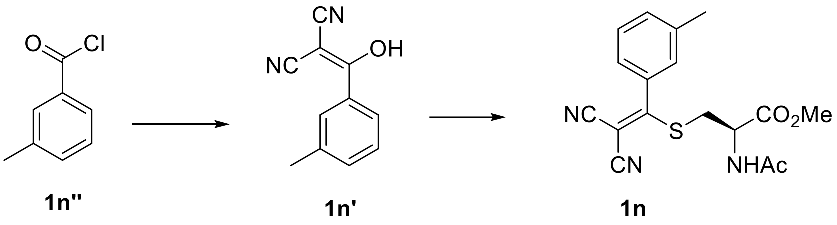

To a suspension of NaH (1440 mg, 60 mmol, 6 eq) in anhydrous THF (10 mL) under a nitrogen atmosphere at 0 °C was added a solution of malononitrile (1982 mg, 30 mmol, 3 eq) in anhydrous THF (10 mL) in a dropwise manner. After 1 hour at 0 °C, a solution of **1n″** (1550 mg, 10 mmol, 1 eq) in anhydrous THF (10 mL) was added to the mixture at 0 °C in a dropwise manner. After the addition, the reaction was allowed to warm up to room temperature. After 2 hours at room temperature, the solvent was removed under reduced pressure. The mixture was acidified to pH 1–2 using $HCl_{(aq)}$. The mixture was extracted with EtOAc (3 × 100 mL). The combined organic layers were washed with brine (3 × 50 mL), dried over Na_2SO_4, filtered and concentrated. Silica gel column chromatography (DCM:MeOH=10:1) was performed to isolate **1n′** (1830 mg, 9.9 mmol, 99%). ^{1}H NMR (600 MHz, Methanol-d_4) δ 7.46 – 7.43 (m, 2H), 7.29 (d, J = 4.8 Hz, 2H), 2.37 (s, 3H). ^{13}C NMR (151 MHz, Methanol-d_4) δ 191.9, 139.2, 139.1, 132.6, 129.1, 129.1, 125.8, 121.5, 119.9, 53.2, 21.4. R_f (DCM:MeOH=6:1) = 0.3.

To a solution of **1n′** (1800 mg, 9.8 mmol, 1.0 eq) in anhydrous acetonitrile (50 mL) under a nitrogen atmosphere was added PCl_5 (6115 mg, 3 mmol, 3 eq) was added. After 6 hours at 65 °C, the solvent was removed under reduced pressure. The residue was dissolved in DCM (60 mL), washed with water (3 × 20 mL) and brine (20 mL), dried over Na_2SO_4, filtered and concentrated. The resulting yellow solid chloride (199 mg, 0.98 mmol, 1.3 eq) was then dissolved in acetonitrile (15 mL), followed by addition of Ac-Cys-OMe (137 mg, 0.77 mmol, 1.0 eq) and $NaHCO_3$ (192 mg, 2.3 mmol, 3 eq). After stirring overnight at room temperature, the solvent was removed under reduced pressure. The mixture was purified by silica gel column chromatography (EA:PE=2:1) to afford **1n** (264 mg, 0.77 mmol, 99%). ^{1}H NMR (600 MHz, Chloroform-d) δ 7.45 – 7.39 (m, 2H), 7.22 (d, J = 6.6 Hz, 2H), 6.27 (d, J = 6.6 Hz, 1H), 4.66 (dt, J = 7.2, 5.1 Hz, 1H), 3.77 (s, 3H), 3.26 – 3.23 (m, 1H), 3.07 – 3.04 (m, 1H), 2.43 (s, 3H), 2.03 (s, 3H). ^{13}C NMR (151 MHz, Chloroform-d) δ 180.2, 170.0, 169.7, 139.9, 133.8, 132.1, 129.6, 129.2, 126.0, 112.7, 112.3, 80.9, 53.4, 51.8, 36.6, 23.2, 21.5. R_f (EA:PE=2:1) = 0.3.

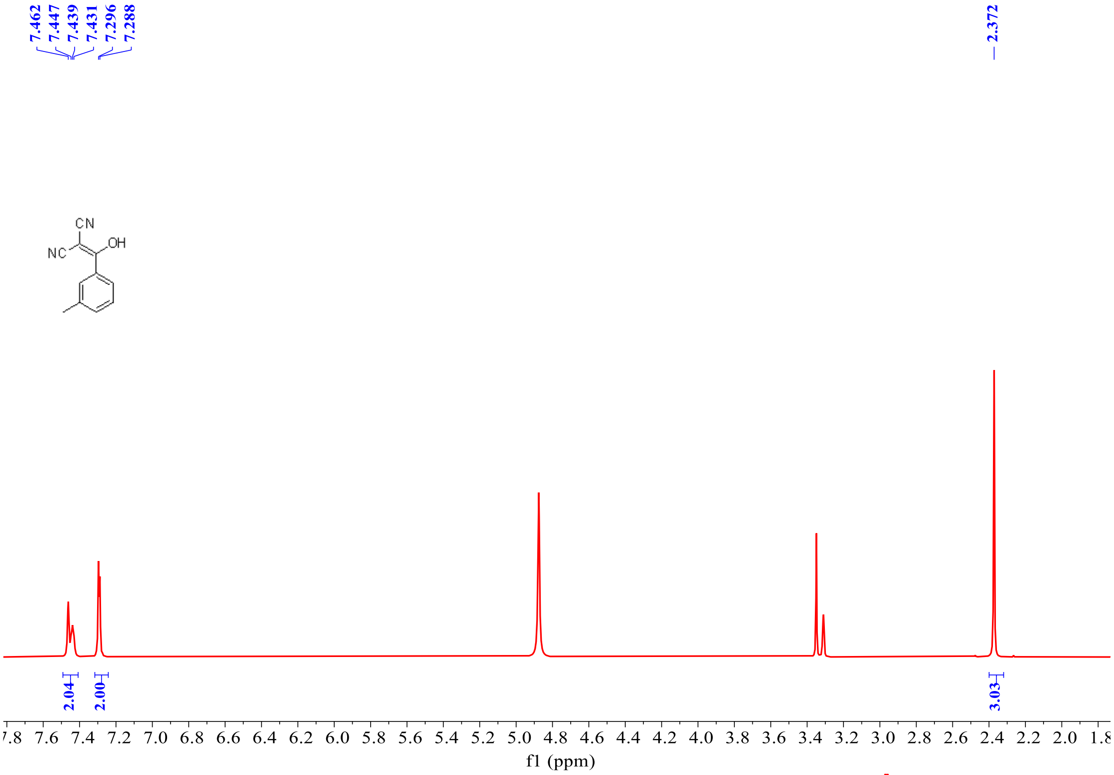
7.462
7.447
7.439
7.431
7.296
7.288
2.372
CN
NC
OH
2.04
2.00
3.03
f1 (ppm)

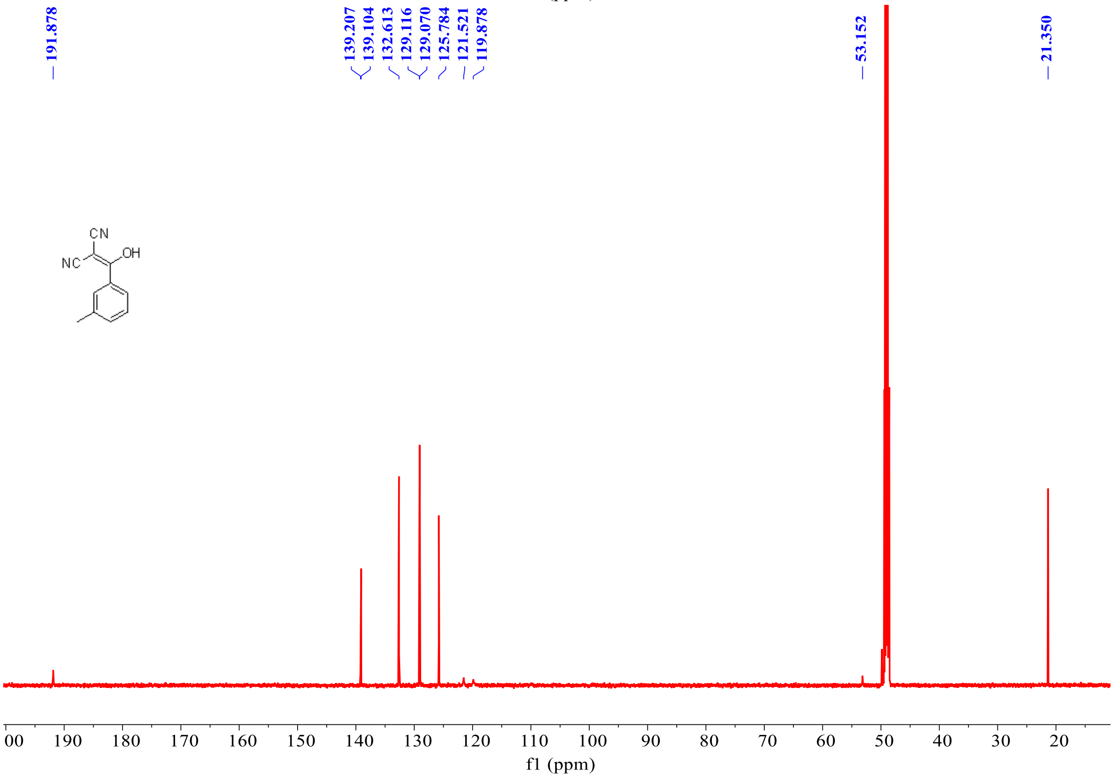
191.878
139.207
139.104
132.613
129.116
129.070
125.784
121.521
119.878
53.152
21.350
CN
NC
OH
f1 (ppm)

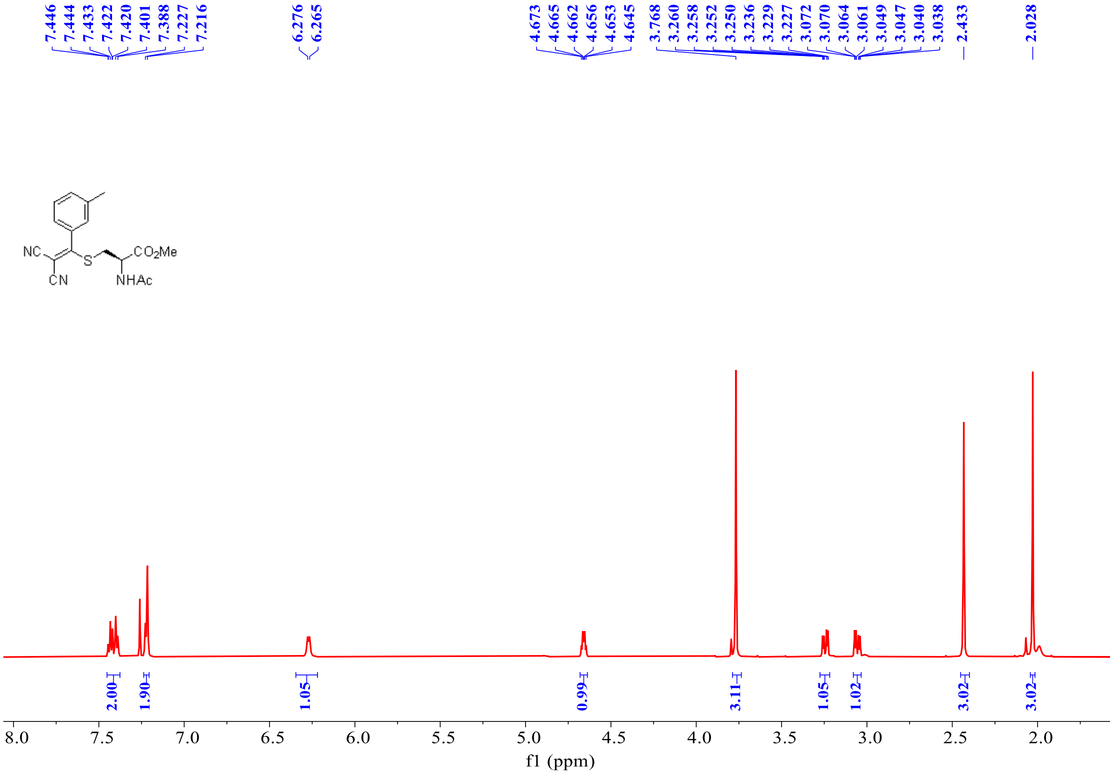
7.446
7.444
7.433
7.422
7.420
7.401
7.388
7.227
7.216
6.276
6.265
4.673
4.665
4.662
4.656
4.653
4.645
3.768
3.260
3.258
3.252
3.250
3.236
3.229
3.227
3.072
3.070
3.064
3.061
3.049
3.047
3.040
3.038
2.433
2.028
NC
CN
S
CO2Me
NHAc
2.00
1.90
1.05
0.99
3.11
1.05
1.02
3.02
3.02
f1 (ppm)

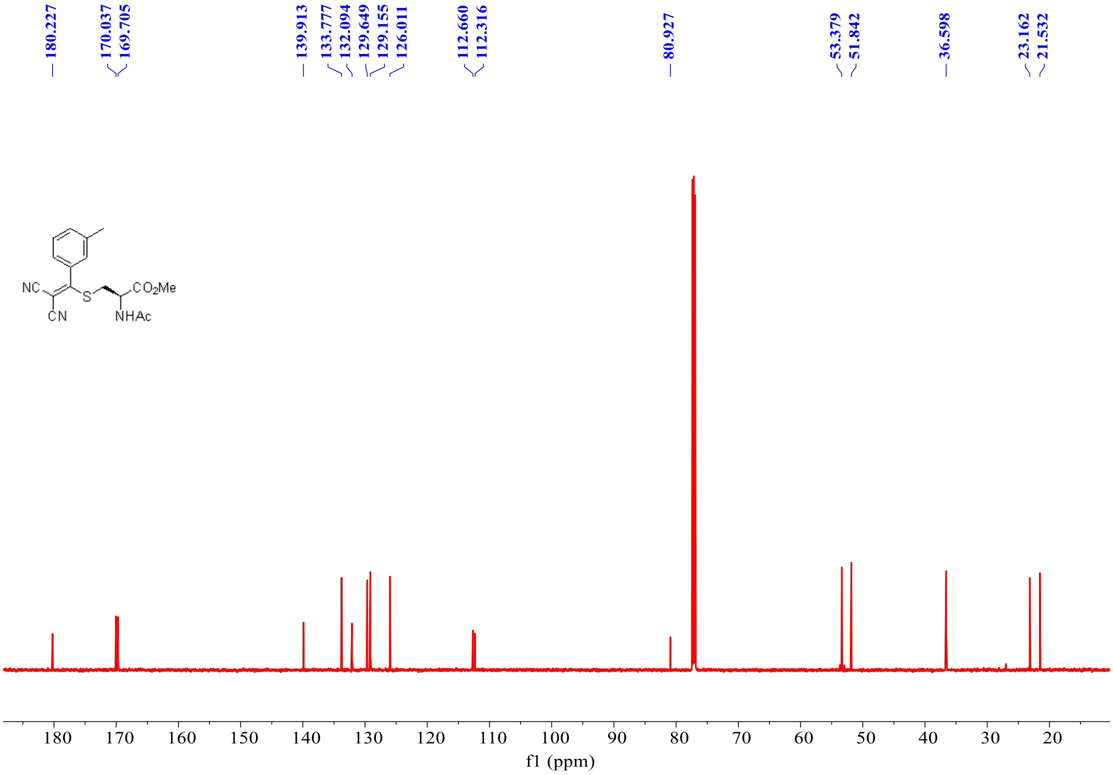
180.227
170.037
169.705
139.913
133.777
132.094
129.649
129.155
126.011
112.660
112.316
80.927
53.379
51.842
36.598
23.162
21.532
NC
CN
S
CO2Me
NHAc
f1 (ppm)

Synthesis of TAMM 1o

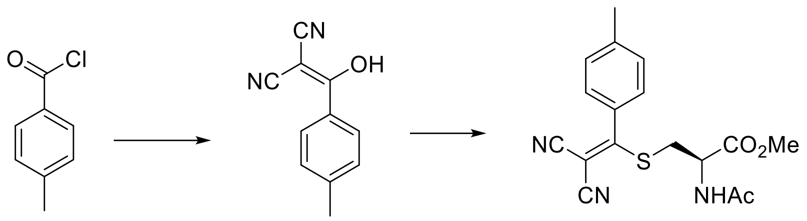

1o" **1o'** **1o**

To a suspension of NaH (1440 mg, 60 mmol, 6 eq) in anhydrous THF (10 mL) under a nitrogen atmosphere at 0 °C was added a solution of malononitrile (1982 mg, 30 mmol, 3 eq) in anhydrous THF (10 mL) in a dropwise manner. After 1 hour at 0 °C, a solution of **1o"** (1550 mg, 10 mmol, 1 eq) in anhydrous THF (10 mL) was added to the mixture at 0 °C in a dropwise manner. After the addition, the reaction was allowed to warm up to room temperature. After 2 hours at room temperature, the solvent was removed under reduced pressure. The mixture was acidified to pH 1–2 using $HCl_{(aq)}$. The mixture was extracted with EtOAc (3 × 100 mL). The combined organic layers were washed with brine (3 × 50 mL), dried over Na_2SO_4, filtered and concentrated. Silica gel column chromatography (DCM:MeOH=10:1) was performed to isolate **1o'** (1835 mg, 9.9 mmol, 99%). ^{1}H NMR (600 MHz, Methanol-*d*$_4$) δ 7.58 (d, *J* = 8.4 Hz, 2H), 7.35 (d, *J* = 7.8 Hz, 2H), 2.42 (s, 3H). ^{13}C NMR (151 MHz, Methanol-*d*$_4$) δ 187.2, 144.8, 131.3, 130.4, 129.2, 117.1, 115.3, 60.4, 21.6. R_f (DCM:MeOH=10:1) = 0.3.

To a solution of **1o'** (810 mg, 4.4 mmol, 1.0 eq) in anhydrous acetonitrile (50 mL) under a nitrogen atmosphere was added PCl_5 (2746 mg, 13.2 mmol, 3 eq) was added. After 6 hours at 65 °C, the solvent was removed under reduced pressure. The residue was dissolved in DCM (60 mL), washed with water (3 × 20 mL) and brine (20 mL), dried over Na_2SO_4, filtered and concentrated. The resulting yellow solid chloride (129 mg, 0.64 mmol, 1.5 eq) was then dissolved in acetonitrile (15 mL), followed by addition of Ac-Cys-OMe (75 mg, 0.42 mmol, 1.0 eq) and $NaHCO_3$ (107 mg, 1.3 mmol, 3 eq). After stirring overnight at room temperature, the solvent was removed under reduced pressure. The mixture was purified by silica gel column chromatography (EA:PE=2:1) to afford **1o** (102 mg, 0.30 mmol, 71%). ^{1}H NMR (600 MHz, Chloroform-*d*) δ 7.35 (s, 4H), 6.25 (d, *J* = 6.6 Hz, 1H), 4.66 (dt, *J* = 7.2, 4.8 Hz, 1H), 3.77 (s, 3H), 3.27 (dd, *J* = 14.1, 4.8 Hz, 1H), 3.08 (dd, *J* = 14.1, 4.8 Hz, 1H), 2.44 (s, 3H), 2.03 (s, 3H). ^{13}C NMR (151 MHz, Chloroform-*d*) δ 179.8, 169.9, 169.6, 144.1, 130.4, 129.1, 128.9, 112.8, 112.4, 80.5, 53.3, 51.8, 36.6, 23.1, 21.70. R_f (EA:PE=2:1) = 0.4.

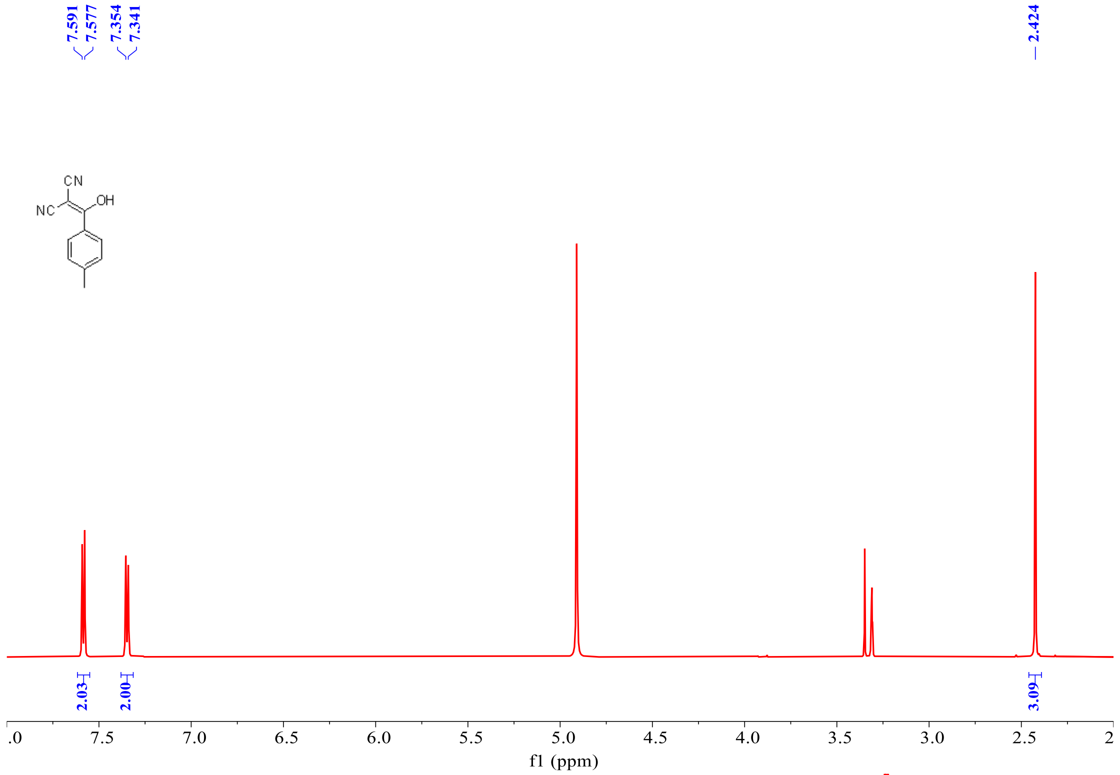
7.591
7.577
7.354
7.341
2.424
CN
NC
OH
2.03
2.00
3.09
f1 (ppm)

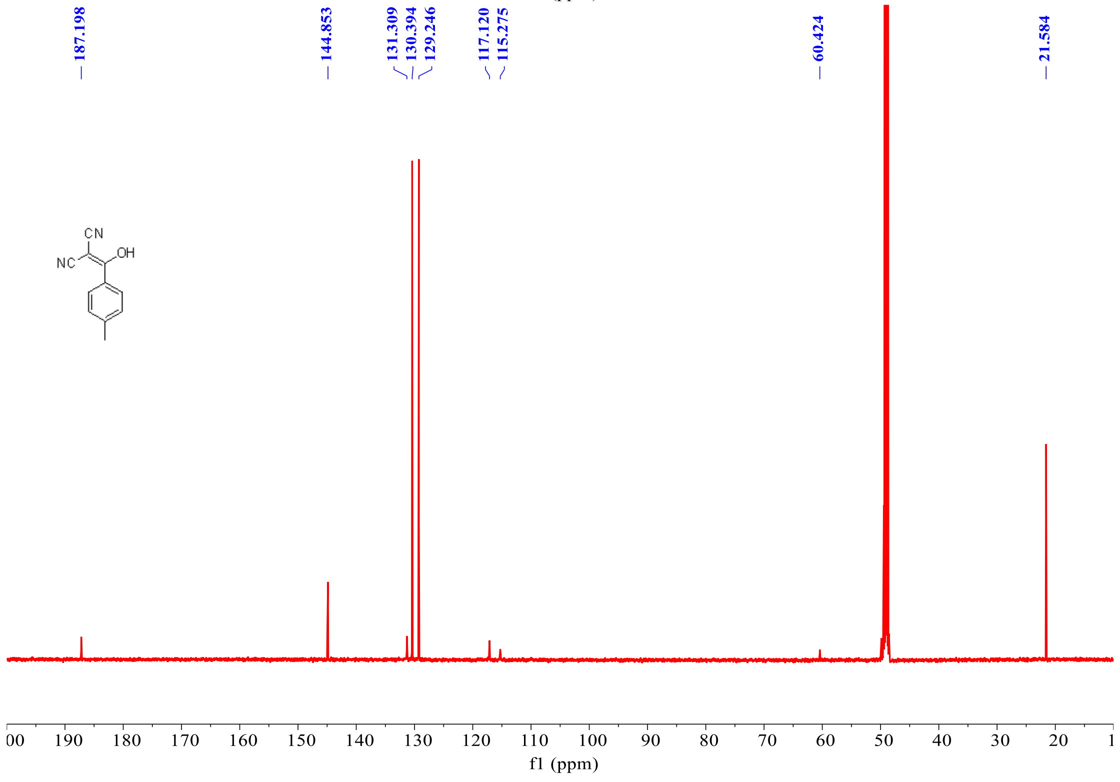
187.198
144.853
131.309
130.394
129.246
117.120
115.275
60.424
21.584
CN
NC
OH
f1 (ppm)

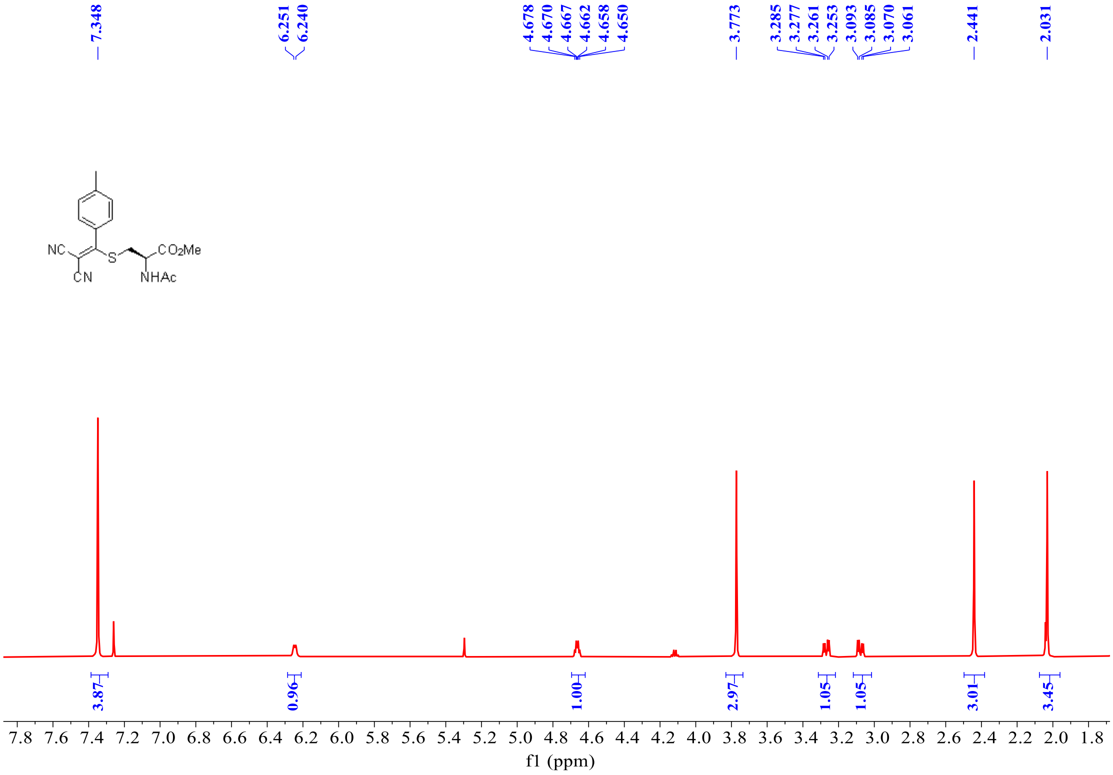
7.348
6.251
6.240
4.678
4.670
4.667
4.662
4.658
4.650
3.773
3.285
3.277
3.261
3.253
3.093
3.085
3.070
3.061
2.441
2.031
NC
CN
S
CO2Me
NHAc
3.87
0.96
1.00
2.97
1.05
1.05
3.01
3.45
f1 (ppm)

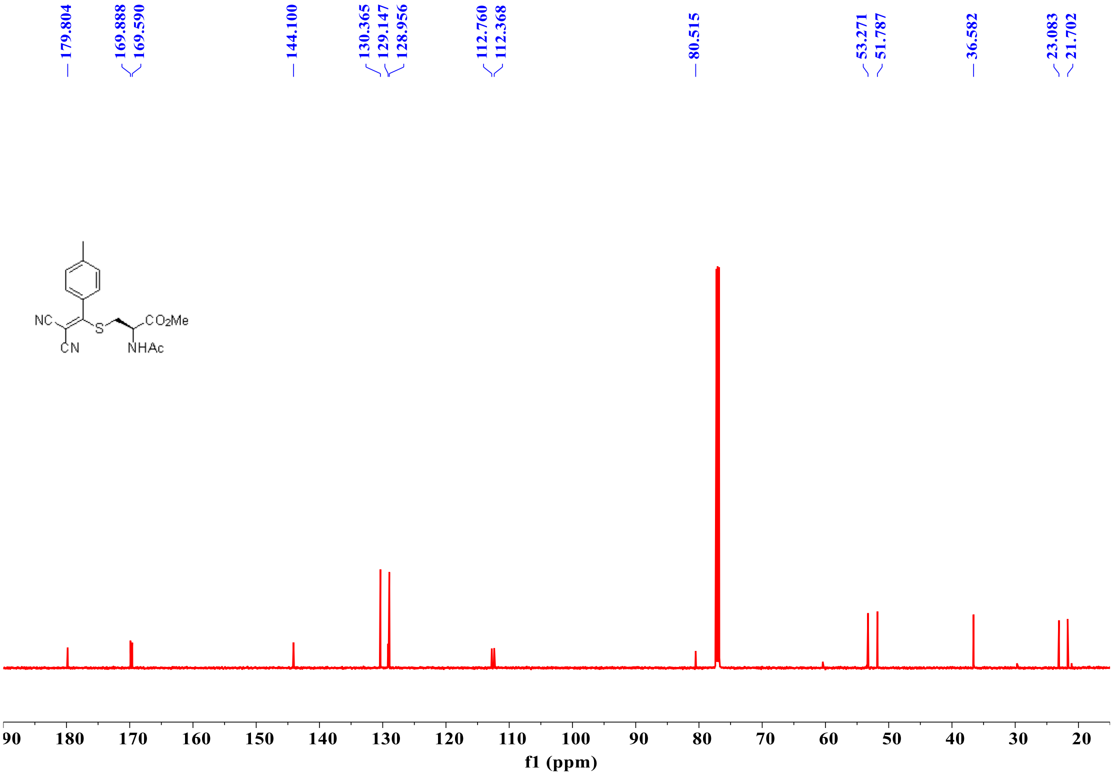
179.804
169.888
169.590
144.100
130.365
129.147
128.956
112.760
112.368
80.515
53.271
51.787
36.582
23.083
21.702
NC
CN
S
CO2Me
NHAc
f1 (ppm)

Synthesis of TAMM 1r

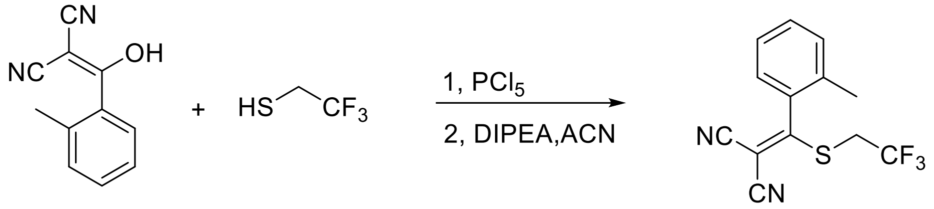

1a' **1r**

To a solution of **1a'** (56 mg, 0.3 mmol, 1.0 eq) in anhydrous acetonitrile (30 mL) was added PCl_5 (190 mg, 0.9 mmol, 3.0 eq). Under a nitrogen atmosphere, the reaction was stirred at 65 °C for 6 hours and then concentrated. The residue was dissolved in DCM (60 mL), washed with water (3 × 20 mL) and brine (20 mL), dried over Na_2SO_4, filtered and concentrated to afford crude product 1 (54 mg, 0.25 mmol, 1.0 eq). The crude product 1 was then dissolved in acetonitrile (10 mL), followed by addition of DIPEA (87 μL, 0.50mmol, 2.0 eq) and 2,2,2-trifluoroethane-1-thiol (27 μL, 0.25 mmol, 1.0 eq). The reaction was stirred at room temperature overnight and then concentrated under reduced pressure. The crude mixture was purified by silica gel column chromatography (PE: EA=5:1) to give compound **1r** (55 mg, 0.20 mmol, 80% yield). ^{1}H NMR (400 MHz, Chloroform-*d*) δ 7.49 (td, *J* = 7.6, 7.6, 1.6 Hz, 1H), 7.41 – 7.37 (m, 2H), 7.14 (dd, *J* = 7.2, 1.6 Hz, 1H), 3.23 – 3.03 (m, 2H), 2.33 (s, 3H). R_f (PE: EA=4:1) = 0.5. ^{13}C NMR (101 MHz, Chloroform-*d*) δ 178.28, 135.86, 132.18, 131.93, 130.34, 128.16, 127.58, 127.27, 124.83, 122.08, 119.33, 111.38, 110.90, 83.20, 35.53, 35.18, 34.83, 34.49, 18.98. ^{19}F NMR (376 MHz, Chloroform-*d*) δ -65.86, -65.88, -65.91.

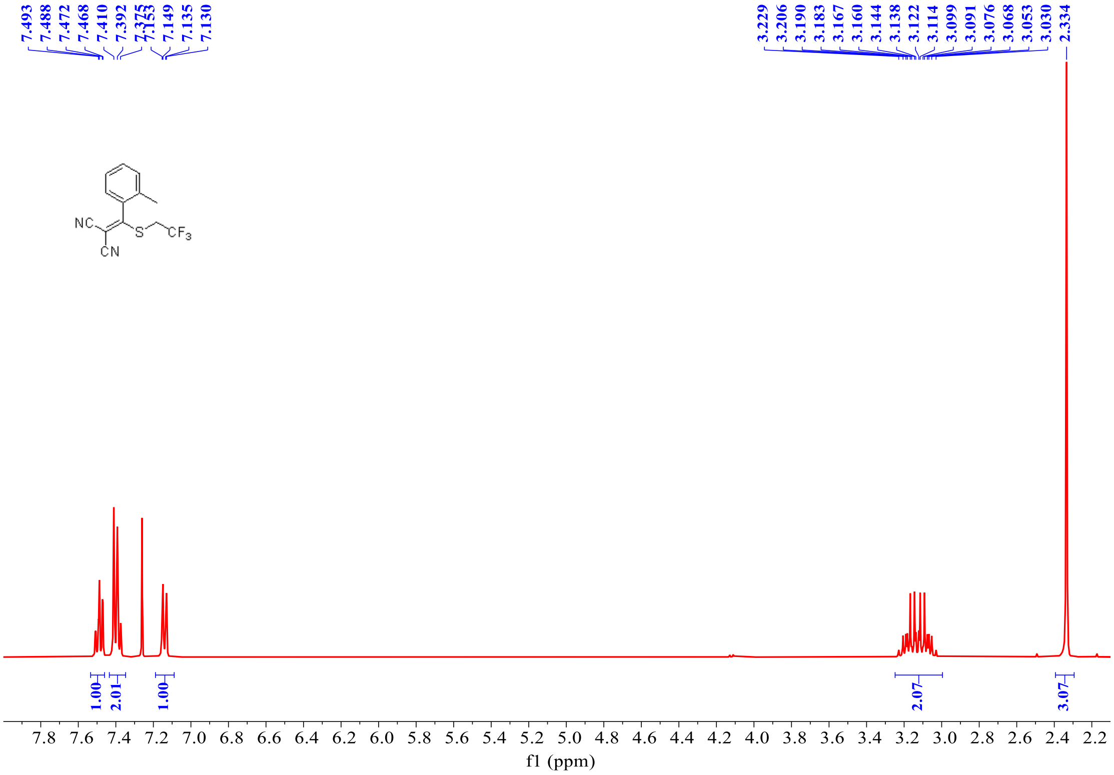

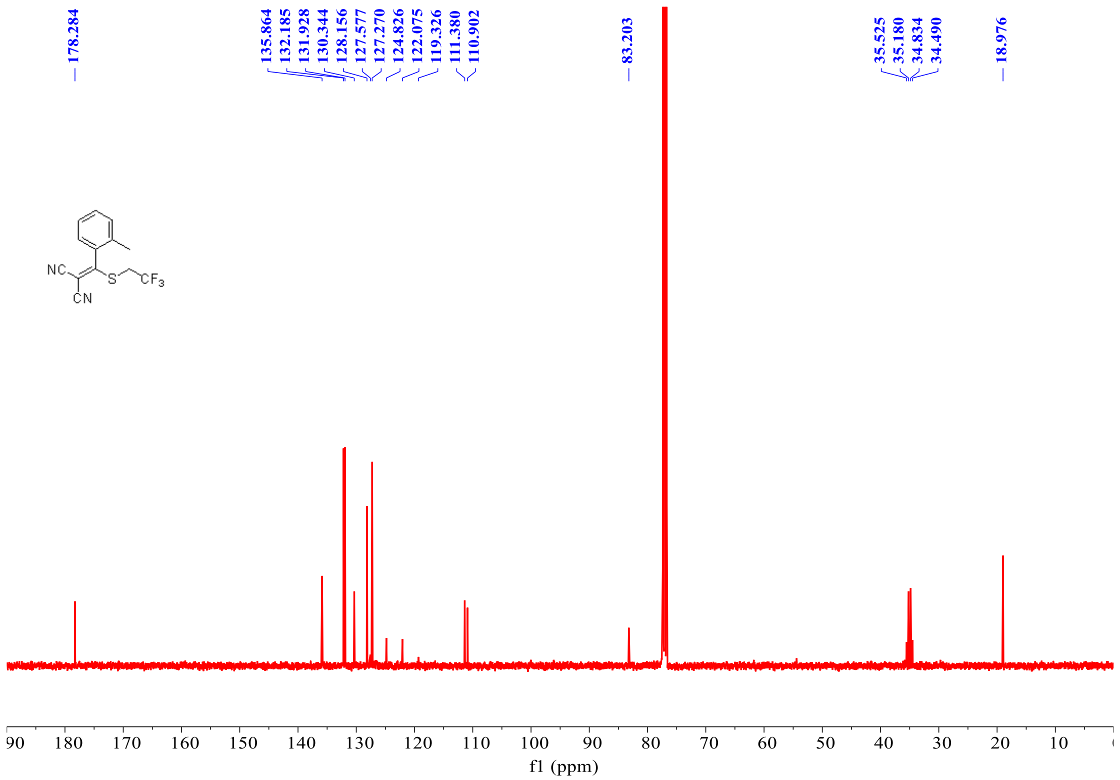
178.284
135.864
132.185
131.928
130.344
128.156
127.577
127.270
124.826
122.075
119.326
111.380
110.902
83.203
35.525
35.180
34.834
34.490
18.976
NC
S
CF3
CN
f1 (ppm)

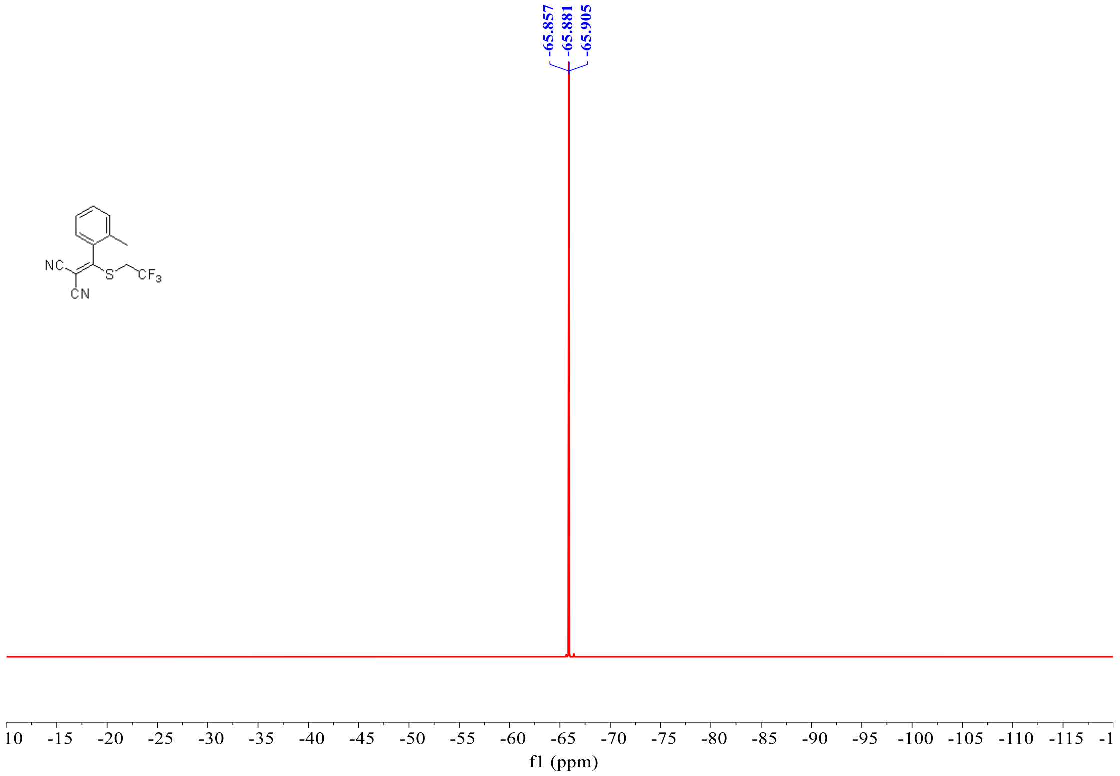
-65.857
-65.881
-65.905
NC
S
CF3
CN
f1 (ppm)

Synthesis of TAMM t1

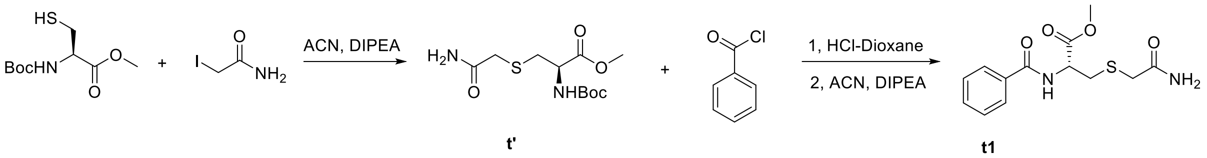

To a 10-mL acetonitrile solution of N-Boc-L-Cysteine methyl ester (1.18 g, 5 mmol, 1.0 eq) were added 2-Iodoacetamide (1.85 g, 10 mmol, 2.0 eq) and N,N-Diisopropylethylamine (2.18 mL, 12.5 mmol, 2.5 eq). The reaction was stirred at room temperature for 6 hours. The solution was concentrated under reduced pressure before purification by silica gel column chromatography to afford **t'** (1.4 g, 4.8 mmol, 95.9% yield). ^{1}H NMR (400 MHz, Chloroform-*d*) δ 6.77 (s, 1H), 6.07 (s, 1H), 5.49 (s, 1H), 4.55 (s, 1H), 3.77 (s, 3H), 3.24 (s, 2H), 3.06 (dd, *J* = 14.0, 5.6 Hz, 1H), 2.96 (dd, *J* = 14.0, 5.6 Hz, 1H), 1.44 (s, 9H). ^{13}C NMR (101 MHz, Chloroform-*d*) δ 173.3, 173.2, 157.3, 82.4, 55.2, 54.7, 37.9, 37.5, 30.2. R_f (DCM:MeOH=20:1) = 0.3.

Compound **t'** (215 mg, 0.74 mmol, 1.0 eq) was dissolved in a 1:1 mixture (10 mL) of HCl in dioxane (4 N) and alcohol. The reaction was stirred at room temperature for 30 min before concentration under reduced pressure. The residue was then dissolved in acetonitrile (10 mL), followed by addition of DIPEA (258 μL, 1.5 mmol, 2.0 eq) and benzoyl chloride (94 μL, 0.80 mmol, 1.1 eq). The reaction was stirred at room temperature overnight and then concentrated under reduced pressure. The crude mixture was purified by silica gel column chromatography (DCM:MeOH=20:1) to give compound **t1** (38 mg, 0.13 mmol, 17.6% yield). ^{1}H NMR (400 MHz, Chloroform-*d*) δ 7.87 – 7.84 (m, 2H), 7.56 – 7.51 (m, 1H), 7.48 – 7.44 (m, 2H), 7.32 (d, *J* = 7.2 Hz, 1H), 6.65 (s, 1H), 5.72 (s, 1H), 5.07 – 5.03 (m, 1H), 3.82 (s, 3H), 3.27 – 3.21 (m, 3H), 3.14 (dd, *J* = 14.4, 6.0 Hz, 1H). ^{13}C NMR (101 MHz, Chloroform-*d*) δ 171.4, 171.3, 167.5, 133.5, 132.2, 128.8, 127.4, 53.1, 52.9, 36.0, 35.2. R_f (DCM:MeOH=10:1) = 0.4. Impurities originate from raw materials and cannot be separated, which does not affect the next reaction step.

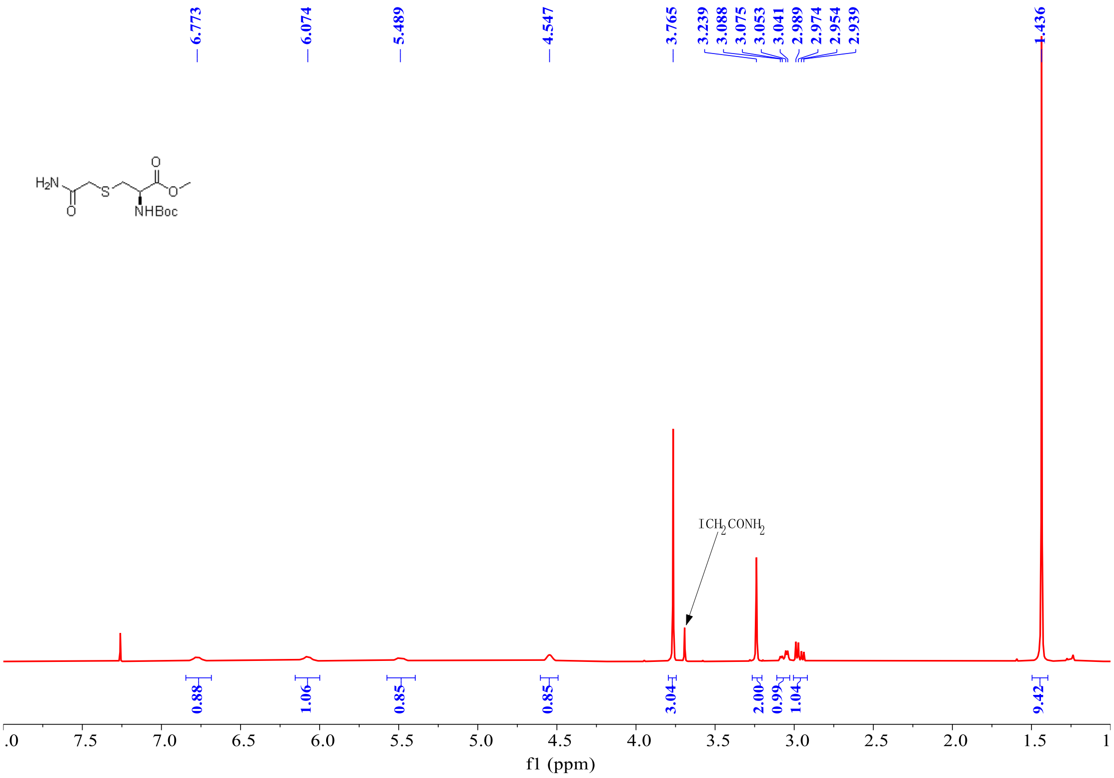

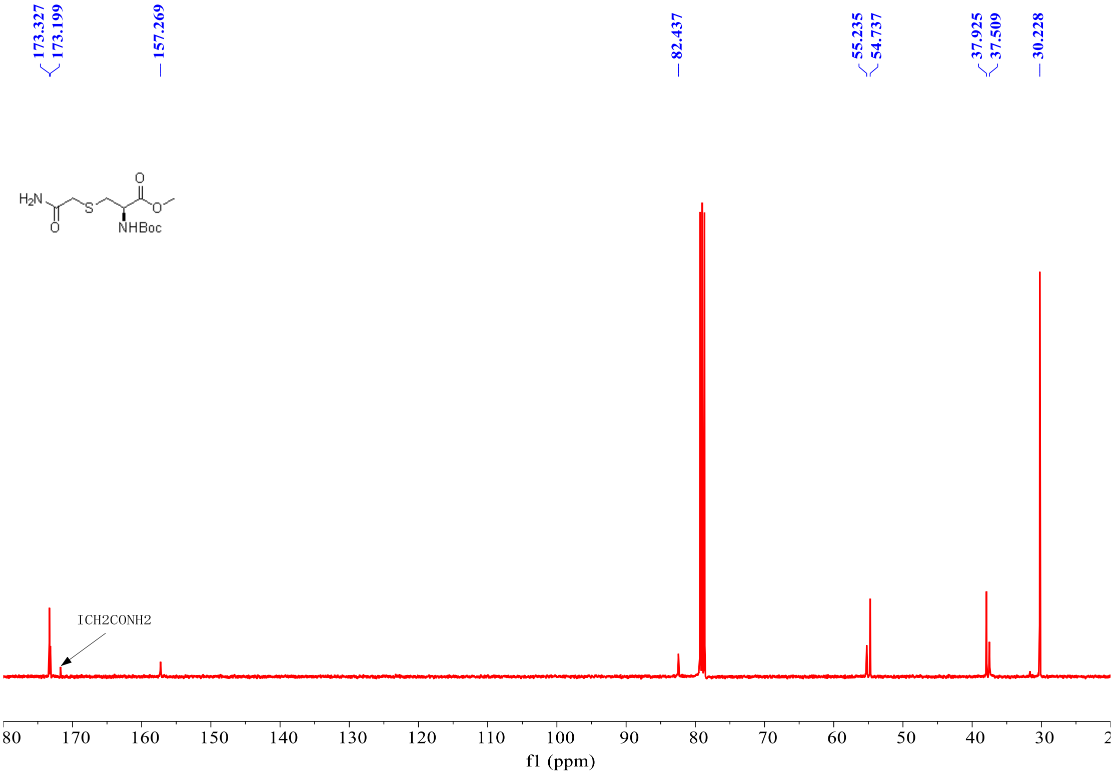
173.327
173.199
157.269
82.437
55.235
54.737
37.925
37.509
30.228
ICH2CONH2
NHBoc
f1 (ppm)

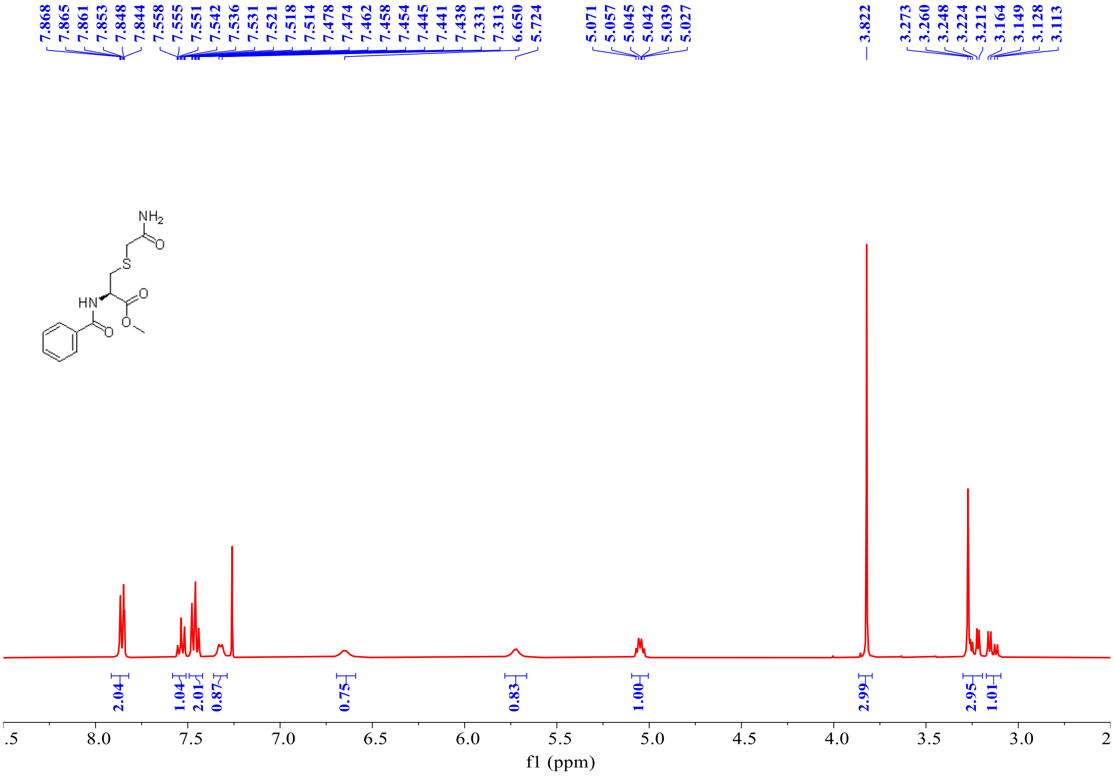
7.868
7.865
7.861
7.853
7.848
7.844
7.558
7.555
7.551
7.542
7.536
7.531
7.521
7.518
7.514
7.478
7.474
7.462
7.458
7.454
7.445
7.441
7.438
7.331
7.313
6.650
5.724
5.071
5.057
5.045
5.042
5.039
5.027
3.822
3.273
3.260
3.248
3.224
3.212
3.164
3.149
3.128
3.113
2.04
1.04
2.01
0.87
0.75
0.83
1.00
2.99
2.95
1.01
f1 (ppm)

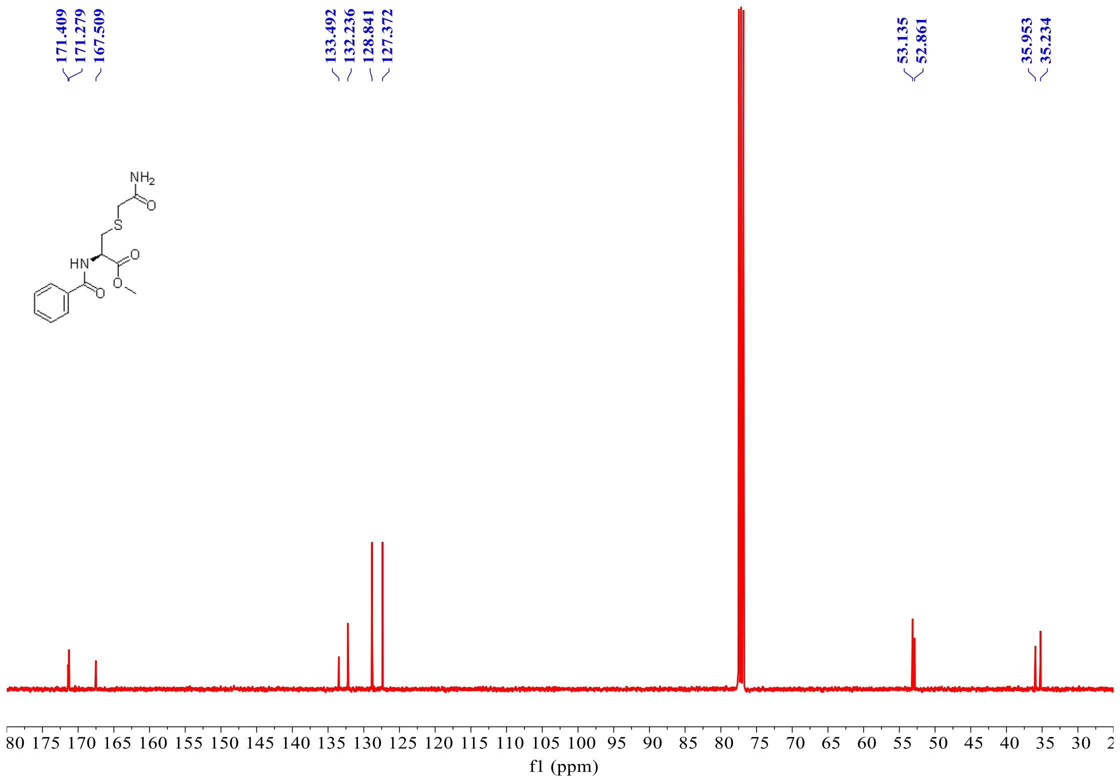

Synthesis of TAMM t2

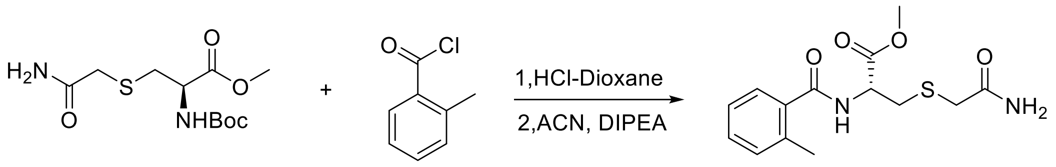

Compound **t'** (215 mg, 0.74 mmol, 1.0 eq) was dissolved in a 1:1 mixture (10 mL) of HCl in dioxane (4 N) and alcohol. The reaction was stirred at room temperature for 30 min before concentration under reduced pressure. The residue was then dissolved in acetonitrile (10 mL), followed by addition of DIPEA (258 µL, 1.5 mmol, 2.0 eq) and o-toluoyl chloride (103 µL, 0.80 mmol, 1.1 eq). The reaction was stirred at room temperature overnight and then concentrated under reduced pressure. The crude mixture was purified by silica gel column chromatography (DCM:MeOH=20:1) to give compound **t2** (36 mg, 0.12 mmol, 16.2% yield). ^{1}H NMR (400 MHz, Chloroform-*d*) δ 7.45 – 7.43 (m, 1H), 7.35 – 7.30 (m, 1H), 7.23 – 7.19 (m, 2H), 6.95 (d, *J* = 7.6 Hz, 1H), 6.81 (d, *J* = 10.4 Hz, 1H), 6.11 (s, 1H), 5.00 (td, *J* = 6.6, 4.8 Hz, 1H), 3.80 (s, 3H), 3.24 – 3.18 (m, 3H), 3.07 (dd, *J* = 14.0, 6.4 Hz, 1H), 2.45 (s, 3H). ^{13}C NMR (101 MHz, Chloroform-*d*) δ 171.7, 171.1, 170.2, 136.6, 135.3, 131.3, 130.5, 127.2, 126.0, 53.1, 52.3, 35.8, 35.2, 20.0. R_f (DCM:MeOH=10:1) = 0.45.

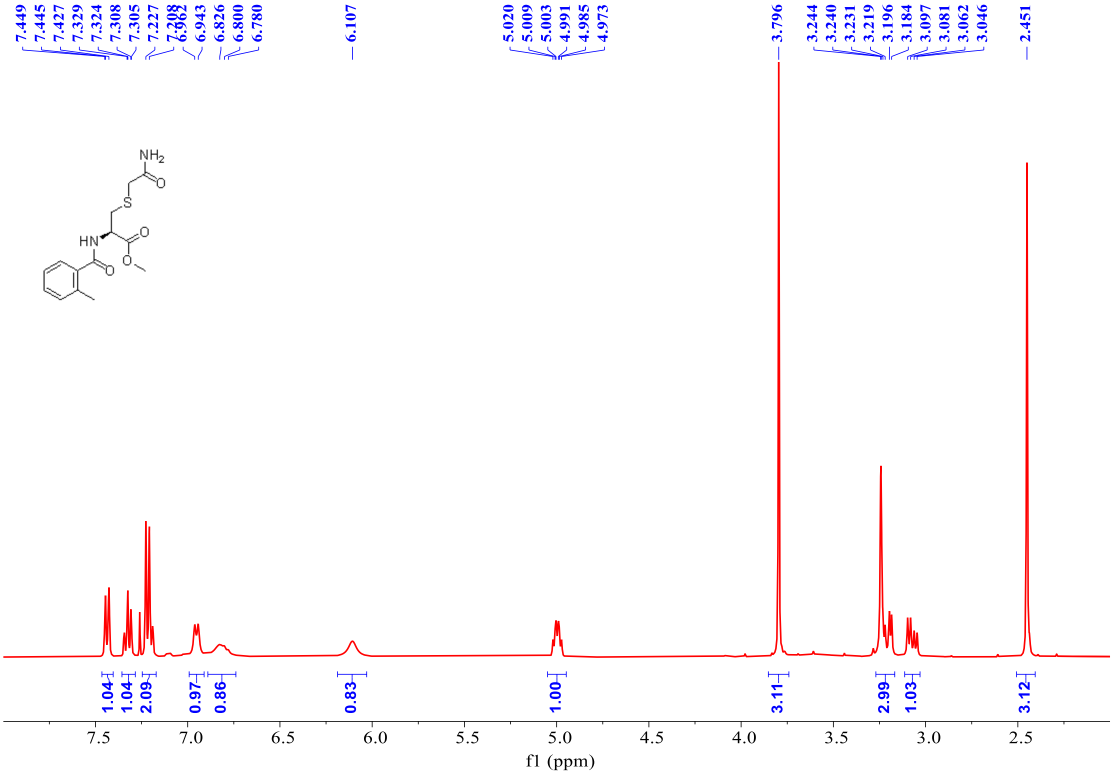
7.449
7.445
7.427
7.329
7.324
7.308
7.305
7.227
6.962
6.943
6.826
6.800
6.780
6.107
5.020
5.009
5.003
4.991
4.985
4.973
3.796
3.244
3.240
3.231
3.219
3.196
3.184
3.097
3.081
3.062
3.046
2.451
NH2
O
S
HN
O
O
O
1.04
1.04
2.09
0.97
0.86
0.83
1.00
3.11
2.99
1.03
3.12
7.5
7.0
6.5
6.0
5.5
5.0
4.5
4.0
3.5
3.0
2.5
f1 (ppm)

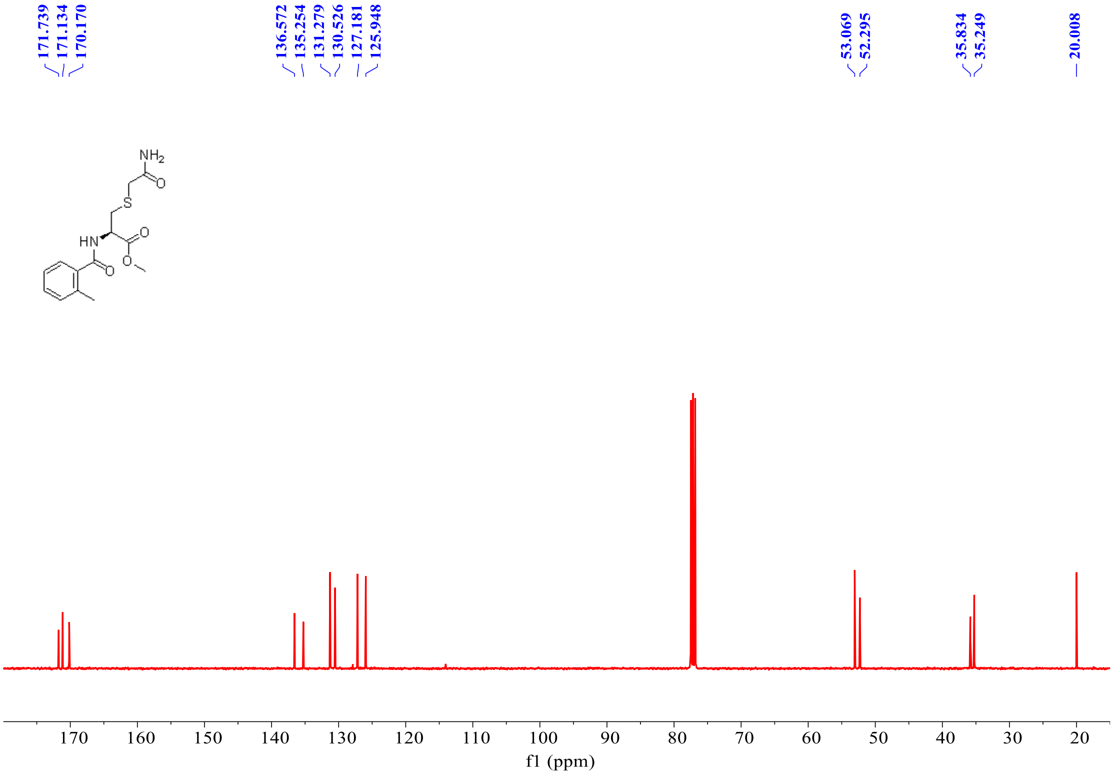
171.739
171.134
170.170
136.572
135.254
131.279
130.526
127.181
125.948
53.069
52.295
35.834
35.249
20.008
NH2
O
S
HN
O
O
O
170
160
150
140
130
120
110
100
90
80
70
60
50
40
30
20
f1 (ppm)

Synthesis of TAMM t3

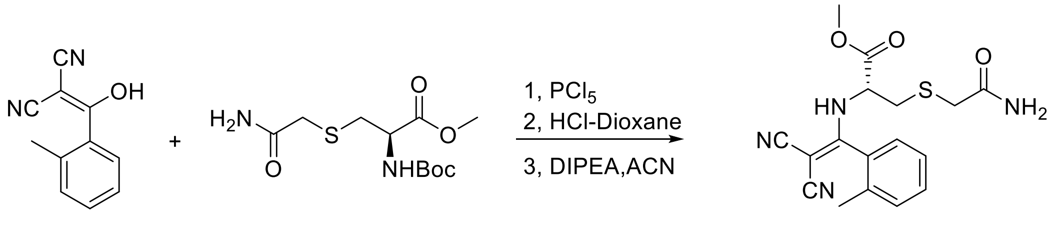

1a' **t'** **t3**

To a solution of **1a′** (56 mg, 0.3 mmol, 1.0 eq) in anhydrous acetonitrile (30 mL) was added PCl_5 (190 mg, 0.9 mmol, 3.0 eq). Under a nitrogen atmosphere, the reaction was stirred at 65 °C for 6 hours and then concentrated. The residue was dissolved in DCM (60 mL), washed with water (3 × 20 mL) and brine (20 mL), dried over Na_2SO_4, filtered and concentrated to afford crude product 1. Compound **t′** (90 mg, 0.31 mmol, 1.0 eq) was dissolved in a 1:1 10 mL solution of HCl dioxane (4 N) and alcohol, the reaction was stirred at room temperature for 30 minutes before being concentrated under reduced pressure to afford crude product 2. The crude products 1 and 2 were then dissolved in acetonitrile (10 mL), followed by addition of DIPEA (105 μL, 0.60 mmol, 2.0 eq). The reaction was stirred at room temperature overnight and then concentrated under reduced pressure. The crude mixture was purified by silica gel column chromatography (DCM:MeOH=20:1) to give compound **t3** (16 mg, 0.04 mmol, 13.3% yield). R_f (DCM:MeOH=10:1) = 0.5.

One-pot sequential diversification of 5px

To a solution of $NaHCO_3$ (0.1 M, pH 8.5), TAMM-Alkyne (200 μM), CGGGKGW (50 μM), Ac-Cys-OMe (500 μM), and TCEP (1 mM) were added sequentially. The mixture was incubated at room temperature for 3 h and analyzed by LC-MS using chromatography condition B. Subsequently, maleimide (1 mM) was added to the reaction mixture, and the reaction was continued for another 1 h, followed by LC-MS analysis. Finally, $CuSO_4$ (1 mM), sodium ascorbate (1 mM), BTTAA (1 mM) and 2-Azidoethanol (250 μM) were added. After 1 h, the mixture was analyzed by LC-MS using chromatography condition B.

One-pot sequential diversification of 5py

To a solution of $NaHCO_3$ (0.1 M, pH 8.5), TAMM-Alkyne (100 μM), CGGGKGW (50 μM) and TCEP (1 mM) were added sequentially. The mixture was incubated at room temperature for 3 h and analyzed by LC-MS using chromatography condition B. Subsequently, maleimide (1 mM) was added to the reaction mixture, and the reaction was continued for another 1 h, followed by LC-MS analysis using chromatography condition B. Finally, $CuSO_4$ (1 mM), sodium ascorbate (1 mM), BTTAA (1 mM) and 2-Azidoethanol (250 μM) were added. After 1 h, the mixture was analyzed by LC-MS using chromatography condition B.

Reaction of TAMM 1 with 2x

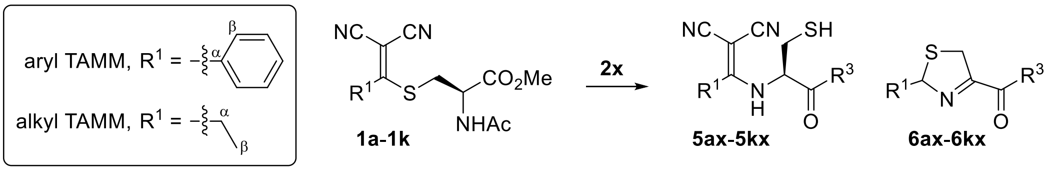

A 200-μL reaction mixture was prepared by mixing 186 μL of 0.1 M $NaHCO_{3(aq)}$ or PBS with Ac-Cys-OMe (2 μL of 50 mM stock solution, 10 eq, final concentration = 500 μM), TCEP (2 μL of 50 mM stock solution, 10 eq, final concentration = 500 μM), **TAMM 1** (8 μL of 5 mM stock solution, 4 eq, final concentration = 200 μM) and peptide **2x** (2 μL of 5 mM stock solution, 1 eq, final concentration = 50 μM). The reaction was incubated at 37 °C for the indicated time and analyzed by LC-MS using chromatography condition B.

Reaction of TAMM 1a/1p with NCys protein

A 20-μL reaction mixture was prepared by mixing 4.2 μL of 0.1 M $NaHCO_{3(aq)}$ with Ac-Cys-OMe (0.5 μL of 50 mM stock solution, 10 eq, final concentration = 500 μM), TCEP (0.5 μL of 50 mM stock solution, 10 eq, final concentration = 500 μM), TAMM 1a /1p (0.2 μL of 5 mM stock solution, 4 eq, final concentration = 200 μM) and NCys-zHER2 or NCys-SUMO (14.3 μL of 70 μM stock solution in 0.1 M $NaHCO_{3(aq)}$, 1 eq, final concentration = 50 μM). The reaction was incubated at 37 °C for the indicated time and analyzed by ESI-MS.

HPLC characterization of peptides

Analytic HPLC analyses were performed on a Shimadzu LC-MS-2020 with a C18 column (Agilent, #770450-902, 5 μm, 4.6×250 mm) at 40 °C and detection of absorbance at 280 nm.

- Chromatography condition A: water (containing 0.1% v/v formic acid) and methanol as the mobile phases at the flow rate of 1 mL/min, 10% to 40% methanol from 0 to 8 min, 40% to 50% methanol from 8 to 12 min, 50% to 90% methanol from 12 to 16 min, 90% methanol for 2 min, 90% to 10% methanol from 18 to 19 min, and 10% methanol from 19 to 24 min.
- Chromatography condition B: water (containing 0.1% v/v formic acid) and methanol as the mobile phases at the flow rate of 1 mL/min, 10% to 70% methanol from 0 to 6 min, 70% to 90% methanol from 6 to 9 min, 90% methanol for 3 min, 90% to 10% methanol from 12 to 13 min, and 10% methanol from 13 to 17 min.
- Chromatography condition C: water (containing 0.1% v/v formic acid) and acetonitrile (containing 0.1% v/v formic acid) as the mobile phases at the flow rate of 1 mL/min, isocratic 10% acetonitrile for 5 min, then 10% to 90% acetonitrile from 5 to 30 min, 90% to 10% acetonitrile from 30 to 31 min, and 10% acetonitrile from 31 to 40 min.
- Chromatography condition D: water (containing 0.1% v/v formic acid) and acetonitrile (containing 0.1% v/v formic acid) as the mobile phases at the flow rate of 1 mL/min, isocratic 5% acetonitrile for 2 min, then 5% to 95% acetonitrile from 2 to 10 min, 95% to 5% acetonitrile from 10 to 10.01 min, and 5% acetonitrile from 10.01 to 15 min.
- Chromatography condition E: water (containing 0.1% v/v formic acid) and acetonitrile (containing 0.1% v/v formic acid) as the mobile phases at the flow rate of 1 mL/min, isocratic 5% acetonitrile for 5 min, then 10% to 40% acetonitrile from 5 to 30 min, 40% to 10% acetonitrile from 30 to 31 min, and 10% acetonitrile from 31 to 40 min.

Mechanistic investigation of o-TAMM properties

Effective reaction barrier

The reaction system under consideration

$$A \underset{k_{-1}}{\overset{k_1}{\rightleftharpoons}} B \overset{k_2}{\rightarrow} C$$

This corresponds to the system of differential equations:

$$\frac{d[A]}{dt} = -k_1[A] + k_{-1}[B]$$
$$\frac{d[B]}{dt} = k_1[A] - (k_{-1} + k_2)[B]$$
$$\frac{d[C]}{dt} = k_2[B]$$

with initial conditions $[A](0) = a_0$, $[B](0) = 0$, and $[C](0) = 0$.

Derivation of the solution under general conditions

The system of differential equations can be written in matrix form as

$$\frac{d}{dt}\begin{bmatrix}[A]\\ [B]\end{bmatrix} = \mathbf{M}\begin{bmatrix}[A]\\ [B]\end{bmatrix}$$

where the coefficient matrix $\mathbf{M}$ is

$$\mathbf{M} = \begin{bmatrix}-k_1 & k_{-1}\\ k_1 & -(k_{-1} + k_2)\end{bmatrix}$$

(Note: we only need to consider the first two differential equations for $[A]$ and $[B]$ since the third one for $[C]$ is related through mass conservation, $[A] + [B] + [C] = a_0$.)

The eigenvalues λ_1, λ_2 are found from the characteristic equation $\det(\mathbf{M} - \lambda\mathbf{I}) = 0$:

$$\lambda_{1,2} = \frac{-(k_1 + k_{-1} + k_2) \pm \sqrt{(k_1 + k_{-1} + k_2)^2 - 4k_1k_2}}{2}$$

The general solution for $[A]$ and $[B]$ takes the form:

$$\begin{bmatrix}[A](t)\\ [B](t)\end{bmatrix} = c_1 e^{\lambda_1 t}\mathbf{v}_1 + c_2 e^{\lambda_2 t}\mathbf{v}_2$$

Here, $\mathbf{v}_1, \mathbf{v}_2$ are eigenvectors. For eigenvalue λ_1, we have:

$$\mathbf{M} - \lambda_1 \mathbf{I} = \begin{bmatrix} -k_1 - \lambda_1 & k_{-1} \\ k_1 & -(k_{-1} + k_2) - \lambda_1 \end{bmatrix}$$

For eigenvector $\mathbf{v}_1 = \begin{bmatrix} v_{11} \\ v_{12} \end{bmatrix}$: $(-k_1 - \lambda_1)v_{11} + k_{-1}v_{12} = 0$, giving $v_{11} = \frac{k_{-1}}{k_1 + \lambda_1} v_{12}$.

Choosing $v_{12} = k_1 + \lambda_1$ yields $v_{11} = k_{-1}$, resulting in:

$$\mathbf{v}_1 = \begin{bmatrix} k_{-1} \\ k_1 + \lambda_1 \end{bmatrix}$$

Similarly, for λ_2:

$$\mathbf{v}_2 = \begin{bmatrix} k_{-1} \\ k_1 + \lambda_2 \end{bmatrix}$$

This gives the solution:

$$[A](t) = c_1 k_{-1} e^{\lambda_1 t} + c_2 k_{-1} e^{\lambda_2 t}$$
$$[B](t) = c_1 (k_1 + \lambda_1) e^{\lambda_1 t} + c_2 (k_1 + \lambda_2) e^{\lambda_2 t}$$

The coefficients c_i can be determined from the initial conditions $[A](0) = a_0$ and $[B](0) = 0$:

$$c_1 = \frac{a_0(k_1 + \lambda_2)}{k_{-1}(\lambda_2 - \lambda_1)}, \qquad c_2 = -\frac{a_0(k_1 + \lambda_1)}{k_{-1}(\lambda_2 - \lambda_1)}$$

Therefore,

$$[A](t) = -\frac{a_0(k_1 + \lambda_2)}{\lambda_1 - \lambda_2} e^{\lambda_1 t} + \frac{a_0(k_1 + \lambda_1)}{\lambda_1 - \lambda_2} e^{\lambda_2 t} = -\frac{a_0}{\lambda_1 - \lambda_2}\left[(k_1 + \lambda_2)e^{\lambda_1 t} - (k_1 + \lambda_1)e^{\lambda_2 t}\right]$$
$$[B](t) = -\frac{a_0(k_1 + \lambda_2)(k_1 + \lambda_1)}{k_{-1}(\lambda_1 - \lambda_2)} e^{\lambda_1 t} + \frac{a_0(k_1 + \lambda_1)(k_1 + \lambda_2)}{k_{-1}(\lambda_1 - \lambda_2)} e^{\lambda_2 t} = \frac{a_0 k_1}{\lambda_1 - \lambda_2}\left[e^{\lambda_1 t} - e^{\lambda_2 t}\right]$$
$$[C](t) = a_0 - [A](t) - [B](t)$$

(The expression in terms of a_0 and k_i without eigenvalues λ_i is too complicated to show in full.)

(Re-)evaluating the solution under the rapid pre-equilibrium conditions

Let's now examine the effect of $k_1, k_{-1} \gg k_2$ on the eigenvalues, which are determined from $\det(\mathbf{M} - \lambda\mathbf{I}) = 0$:

$$\lambda^2 + (k_1 + k_{-1} + k_2)\lambda + k_1 k_2 = 0$$

$$s = k_1 + k_{-1} + k_2$$

$$\Delta = s^2 - 4k_1 k_2$$

$$\lambda_{1,2} = \frac{-s \pm \sqrt{\Delta}}{2}$$

Under $k_1, k_{-1} \gg k_2$,

$$s = k_1 + k_{-1} + k_2 \approx k_1 + k_{-1}$$

$$\Delta = (k_1 + k_{-1} + k_2)^2 - 4k_1 k_2 \approx (k_1 + k_{-1})^2 - 4k_1 k_2$$

$$\sqrt{\Delta} \approx (k_1 + k_{-1})(1 - \frac{4k_1 k_2}{(k_1 + k_{-1})^2})^{1/2} \approx (k_1 + k_{-1}) - \frac{2k_1 k_2}{k_1 + k_{-1}}$$

$$\lambda_1 \approx -\frac{k_1 k_2}{k_1 + k_{-1}}$$

$$\lambda_2 \approx -(k_1 + k_{-1})$$

(Binomial approximation $(1 + x)^n \approx 1 + nx$ for $|x| \ll 1$ is used.)

As $(k_1 + k_{-1})$ is large, the $e^{\lambda_2 t}$ term decays rapidly (***fast mode***), whereas the $e^{\lambda_1 t} \approx e^{-k_{\text{eff}} t}$ term dominates the decay behavior of $[A](t)$, $[B](t)$, and $[C](t)$ (***slow mode***). Consequently, while $[A](t)$, $[B](t)$, and $[C](t)$ are originally biexponential, under $k_1, k_{-1} \gg k_2$, they simplify to single exponential functions (i.e., omitting the fast mode):

$$[A](t) = \frac{a_0 k_{-1}}{k_1 + k_{-1}} e^{-\frac{k_1 k_2}{k_1 + k_{-1}} t}$$

$$[B](t) = \frac{a_0 k_1}{k_1 + k_{-1}} e^{-\frac{k_1 k_2}{k_1 + k_{-1}} t}$$

$$[C](t) = a_0 (1 - e^{-\frac{k_1 k_2}{k_1 + k_{-1}} t})$$

It should be noted that this result could be similarly obtained under quasi-steady-state approximation (QSSA, not discussed here).

Effective reaction barrier under the rapid pre-equilibrium conditions

Following the above discussion, under $k_1, k_{-1} \gg k_2$, the formation of C follows:

$$\frac{d[C]}{dt} = k_2[B] = k_2 \cdot \frac{a_0 k_1 e^{-k_{\text{eff}} t}}{k_1 + k_{-1}} = a_0 k_{\text{eff}} \cdot e^{-k_{\text{eff}} t}$$

with

$$k_{\text{eff}} = \frac{k_1 k_2}{k_1 + k_{-1}}$$

Thus, **k_{eff} is the effective rate constant for C's formation**. To find its activation energy $\Delta G_{\text{eff}}^{\ddagger}$, transition state theory is considered here: $k_i = \frac{k_B T}{h} e^{-\Delta G_i^{\ddagger}/RT}$. Substituting these k_i into k_{eff} gives:

$$\begin{aligned}
k_{\text{eff}} &= \frac{(\frac{k_B T}{h} e^{-\Delta G_1^{\ddagger}/RT})(\frac{k_B T}{h} e^{-\Delta G_2^{\ddagger}/RT})}{\frac{k_B T}{h} e^{-\Delta G_1^{\ddagger}/RT} + \frac{k_B T}{h} e^{-\Delta G_{-1}^{\ddagger}/RT}} \\
&= \frac{k_B T}{h} \cdot \frac{e^{-(\Delta G_1^{\ddagger} + \Delta G_2^{\ddagger})/RT}}{e^{-\Delta G_1^{\ddagger}/RT} + e^{-\Delta G_{-1}^{\ddagger}/RT}} \\
&= \frac{k_B T}{h} \cdot \frac{e^{-\Delta G_2^{\ddagger}/RT}}{e^{\Delta G_1^{\ddagger}/RT}(e^{-\Delta G_1^{\ddagger}/RT} + e^{-\Delta G_{-1}^{\ddagger}/RT})} \\
&= \frac{k_B T}{h} \cdot \frac{e^{-(\Delta G_1^{\ddagger} + \Delta G_2^{\ddagger})/RT}}{e^{-\Delta G_1^{\ddagger}/RT} + e^{-\Delta G_{-1}^{\ddagger}/RT}}
\end{aligned}$$

Since $k_{\text{eff}} = \frac{k_B T}{h} e^{-\Delta G_{\text{eff}}^{\ddagger}/RT}$, we focus on:

$$e^{-\Delta G_{\text{eff}}^{\ddagger}/RT} = \frac{e^{-\Delta G_2^{\ddagger}/RT}}{1 + e^{-(\Delta G_{-1}^{\ddagger} - \Delta G_1^{\ddagger})/RT}}$$

or

$$\Delta G_{\text{eff}}^{\ddagger} = \Delta G_2^{\ddagger} + RT\ln(1 + e^{-(\Delta G_{-1}^{\ddagger} - \Delta G_1^{\ddagger})/RT})$$

or equivalently,

$$\Delta G_{\text{eff}}^{\ddagger} = \Delta G_2^{\ddagger} + RT\ln(1 + \frac{1}{K})$$

where K is the equilibrium constant for $A \rightleftharpoons B$.

Case 1: Moderate K:

- Either $k_1 > k_{-1}$ or $k_1 < k_{-1}$ (i.e., $K > 1$ or $K < 1$)
- $\Delta G^{\ddagger}_{\text{eff}}$ depends primarily on $\Delta G^{\ddagger}_2$ (slow step) and is further modulated by the difference in $(\Delta G^{\ddagger}_{-1} - \Delta G^{\ddagger}_1)$
- $\Delta G^{\ddagger}_{\text{eff}}$ is generally larger than $\Delta G^{\ddagger}_2$

Case 2: Large K:

- $k_1 \gg k_{-1}$ and $K \gg 1$ (i.e., the equilibrium strongly lies on B)
- $\ln\left(1 + e^{-(\Delta G^{\ddagger}_{-1} - \Delta G^{\ddagger}_1)/RT}\right) \approx \ln(1 + 0) = 0$
- $\Delta G^{\ddagger}_{\text{eff}} \approx \Delta G^{\ddagger}_2$. In other words, only the barrier from the reactive intermediate B is important; the precursor A is not contributing to the reaction kinetics as it is much higher in energy.

Case 3: Small K:

- $k_1 \ll k_{-1}$ and $K \ll 1$ (i.e., $\frac{1}{K} \gg 1$ and $1 + \frac{1}{K} \approx \frac{1}{K}$)
- $\Delta G^{\ddagger}_{\text{eff}} \approx \Delta G^{\ddagger}_2 + RT \cdot \frac{\Delta G^{\ddagger}_1 - \Delta G^{\ddagger}_{-1}}{RT} = \Delta G^{\ddagger}_2 + \Delta G^{\ddagger}_1 - \Delta G^{\ddagger}_{-1} = \Delta G^{\ddagger}_2 + \Delta G_{\text{B-A}}$, as $\Delta G_{\text{B-A}} = \Delta G^{\ddagger}_1 - \Delta G^{\ddagger}_{-1}$. In other words, the effective barrier is the sum of the barrier of the slow step ($\Delta G^{\ddagger}_2$) and the energy difference between the reactive intermediate and its lower-energy precursor ($\Delta G_{\text{B-A}}$), i.e., $\Delta G^{\ddagger}_{\text{eff}} \approx \Delta G^{\ddagger}_2 + \Delta G_{\text{B-A}}$.
- This scenario applies to the TAMM reactions studied here.

Conformation scan and Cartesian coordinates of optimized geometries

Two-dimensional conformational scans of the dihedral angles were conducted at the GFN2-Xtb [1] level using Gaussian 09 [2] through the `external` command.

The Global Optimizer Algorithm (GOAT) implemented in ORCA 6.0 [1, 3] was used to find a set of low-energy conformers for each species. These structures were then further optimized at the level of ωB97X-D/6-31+G(d,p) in vacuum, and the electronic energies in water were calculated at the same level using the SMD model in Gaussian 09.[2] Thermal correction, used for free energy calculation, was evaluated using Shermo 2.3 [4] with a scaling factor for zero-point energy = 0.9523, low-frequency vibration modes (< 100 cm^{-1}) raised to 100 cm^{-1}, and temperature = 298.15 K. Electronic energies are given in Hartrees and coordinates in Ångstroms. Transition states were labelled with a superscript double dagger.

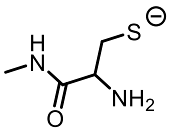

E (vac) = -740.733367673

E(H_2O) = -740.825573221

S	2.149419	-1.109188	-0.101728
C	1.493598	0.518197	0.439333
H	2.281458	1.273408	0.340515
H	1.216552	0.480797	1.504901
C	0.284500	1.069167	-0.342489
N	0.106600	2.497045	-0.058229
H	-0.878974	2.713167	-0.199363
H	0.274188	2.645970	0.934391
H	0.510966	0.954675	-1.411273
C	-1.030908	0.300065	-0.098652
O	-2.112666	0.893886	0.028856
N	-0.930412	-1.037378	-0.078666
H	0.030956	-1.431374	-0.127673
C	-2.081432	-1.877951	0.129341
H	-2.842408	-1.703790	-0.640282
H	-1.759604	-2.921277	0.085276
H	-2.550369	-1.690204	1.103376

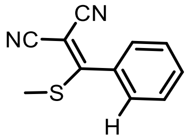

E (vac) = -931.445722924

E (H_2O) = -931.457429057

C	0.677127	0.231324	-0.042621
C	1.556220	-0.815138	-0.066333
C	1.102973	-2.170674	-0.173384
N	0.751786	-3.272947	-0.256103
C	2.974266	-0.615789	-0.017041
N	4.120928	-0.445771	0.021839
C	-2.949129	0.193149	-1.086098
C	-1.579123	0.427742	-1.098194
C	-0.786228	-0.004947	-0.029844
C	-1.377101	-0.662214	1.052315
C	-2.751467	-0.882567	1.065530
C	-3.537606	-0.457299	-0.001980
H	-3.557630	0.514930	-1.924831
H	-1.115458	0.928896	-1.943105
H	-3.204657	-1.394791	1.907613
H	-4.607704	-0.637001	0.006693
S	1.301426	1.863930	-0.066242
C	-0.099379	2.852972	0.531581
H	-0.486348	2.453165	1.469394
H	-0.898081	2.918899	-0.206518
H	0.314974	3.847202	0.708761
H	-0.760235	-1.002522	1.878123

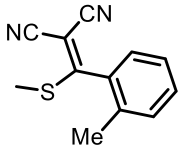

E (vac) = -970.757532500

E (H_2O) = -970.768597255

C	-0.744520	0.250878	-0.168330
C	-1.614434	-0.800877	-0.198672
C	-1.130462	-2.147133	-0.265910
N	-0.739263	-3.238449	-0.303814
C	-3.032869	-0.613354	-0.145981
N	-4.178206	-0.435242	-0.101295
C	2.748487	-0.124258	-1.524614
C	1.375746	0.081108	-1.448076
C	0.723031	0.016164	-0.215097
C	1.432617	-0.255682	0.965272
C	2.811717	-0.451955	0.864695
C	3.466901	-0.387898	-0.361622
H	3.250452	-0.082425	-2.485335
H	0.799550	0.284275	-2.346224
H	3.378940	-0.666539	1.765892
H	4.538987	-0.549609	-0.408669
S	-1.365149	1.880024	-0.080420
C	0.137275	2.883029	0.087681
H	0.699662	2.609084	0.981053
H	-0.221441	3.909166	0.187519
H	0.773230	2.804283	-0.793972
C	0.729197	-0.363172	2.293942
H	0.033996	0.466800	2.458304
H	0.146670	-1.289048	2.348600
H	1.448502	-0.371641	3.115592

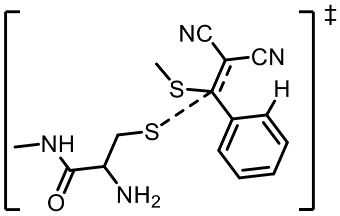

E (vac) = -1672.20217180

E (H_2O) = -1672.27971003

C	-0.456857	0.637024	-0.122876
C	0.031987	1.927354	-0.415400
C	-0.064897	2.466048	-1.728356
N	-0.149027	2.939807	-2.789397
C	0.768409	2.714941	0.503330
N	1.347758	3.393730	1.253897
S	1.316390	-0.803359	-1.294353
C	1.472131	-2.368959	-0.371767
C	-3.122444	-1.661721	-1.584221
C	-1.932595	-1.223877	-1.017801
C	-1.690851	0.141549	-0.826236
C	-2.684965	1.053444	-1.195449
C	-3.875225	0.615190	-1.772838
C	-4.097935	-0.742724	-1.972326
H	-3.282958	-2.724933	-1.735578
H	-1.167644	-1.937985	-0.739807
H	-4.626712	1.343096	-2.063240
H	-5.023505	-1.086313	-2.425512
S	-0.439693	-0.026285	1.526281
C	1.2530	0.205444	2.133944
H	1.432499	-0.597190	2.852867
H	1.394991	1.177577	2.602929
H	1.932865	0.089268	1.288929
H	0.891235	-3.152172	-0.870803
H	1.037404	-2.236578	0.631973
C	2.897328	-2.943526	-0.222981
N	2.796258	-4.321007	0.250016
H	3.713239	-4.614907	0.576529
H	2.215210	-4.331729	1.085162
H	3.353513	-2.961079	-1.220699
C	3.823375	-2.097773	0.679135
O	4.368573	-2.579778	1.675388
N	4.034671	-0.826649	0.277920
H	3.378972	-0.461321	-0.421030
C	4.803976	0.091817	1.088160
H	4.855882	1.052184	0.572734
H	4.346600	0.243700	2.073699
H	5.815227	-0.294316	1.243682
H	-2.531265	2.112657	-1.023565

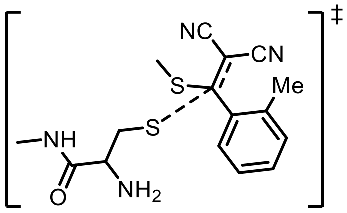

E (vac) = -1711.50955342

E (H_2O) = -1711.58351215

C	-0.400605	0.544206	-0.268926
C	0.178524	1.765292	-0.666750
C	-0.161119	2.333652	-1.923735
N	-0.458265	2.837679	-2.931776
C	1.236193	2.408841	0.021655
N	2.100706	2.961188	0.575692
S	1.337728	-1.011656	-1.285084
C	1.532801	-2.496197	-0.245642
C	-3.116522	-1.411355	-2.119754
C	-1.886469	-1.039386	-1.589101
C	-1.725713	0.167287	-0.904097
C	-2.842200	1.016440	-0.724074
C	-4.068214	0.619927	-1.267583
C	-4.215359	-0.575099	-1.962766
H	-3.207381	-2.351040	-2.656097
H	-1.010763	-1.662884	-1.724077
H	-4.928016	1.272461	-1.137103
H	-5.182370	-0.847423	-2.376536
S	-0.455288	-0.070675	1.400518
C	1.167801	0.229876	2.144725
H	1.245431	-0.478934	2.972802
H	1.280596	1.249226	2.509578
H	1.942870	0.004840	1.412070
H	0.980375	-3.332781	-0.685849
H	1.082251	-2.299834	0.739918
C	2.978422	-2.999491	-0.047510
N	2.931308	-4.349439	0.509289
H	3.829819	-4.538540	0.947687
H	2.264395	-4.358325	1.277620
H	3.448557	-3.057776	-1.037812
C	3.870635	-2.069614	0.808516
O	4.479813	-2.496978	1.793412
N	3.974268	-0.794696	0.384628
H	3.323381	-0.501509	-0.349986
C	4.711502	0.203566	1.129751
H	4.386521	1.192809	0.801165
H	4.517789	0.100170	2.202253
H	5.790864	0.100001	0.972556
C	-2.783363	2.329675	0.019552
H	-2.174751	2.262448	0.924395
H	-2.354750	3.114634	-0.611240
H	-3.792590	2.639912	0.305874

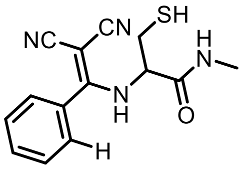

E (vac) = -1234.07614451

E (H_2O) = -1234.09961488

C	4.164869	-2.679351	-0.868737
N	3.407031	-1.453812	-0.687129
C	2.152063	-1.475371	-0.208975
O	1.536794	-2.508851	0.041865
C	1.498462	-0.098249	0.030689
C	2.141428	0.629072	1.236096
S	3.732246	1.440630	0.866284
N	0.097776	-0.373767	0.272581
C	-0.979855	0.365130	-0.028131
C	-0.977005	1.688897	-0.455170
C	0.183791	2.512273	-0.516393
N	1.119174	3.201625	-0.563372
C	-2.183535	2.317707	-0.891712
N	-3.143691	2.859480	-1.256555
C	-2.267215	-0.368079	0.123487
C	-3.305247	0.161995	0.893921
C	-4.488859	-0.551176	1.047406
C	-4.645201	-1.790174	0.429662
C	-3.612161	-2.321353	-0.339195
C	-2.422229	-1.616619	-0.488463
H	4.422415	-3.135843	0.091954
H	3.575000	-3.396158	-1.442881
H	5.078842	-2.445455	-1.414945
H	3.882882	-0.561055	-0.733541
H	1.606537	0.511834	-0.869904
H	1.448419	1.382470	1.612739
H	2.328068	-0.082668	2.044937
H	3.194981	2.465480	0.177426
H	-0.062253	-1.340559	0.546563
H	-3.186507	1.127581	1.374062
H	-5.290745	-0.135693	1.648270
H	-5.572980	-2.340900	0.546313
H	-3.732071	-3.283149	-0.826559
H	-1.620739	-2.028029	-1.095487

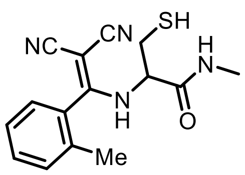

E (vac) = -1273.38671562

E (H_2O) = -1273.4095775

C	4.162058	-2.783059	-0.607481
N	3.450944	-1.521082	-0.502767
C	2.193452	-1.466972	-0.035476
O	1.540344	-2.460952	0.274503
C	1.585796	-0.055530	0.104847
C	2.270420	0.757470	1.228973
S	3.873273	1.492353	0.762690
N	0.183069	-0.266310	0.394022
C	-0.873609	0.479705	0.045194
C	-0.848915	1.762517	-0.484441
C	0.330746	2.540367	-0.668892
N	1.286753	3.185708	-0.816073
C	-2.068951	2.389589	-0.886823
N	-3.043805	2.921650	-1.225380
C	-2.186414	-0.192181	0.272400
C	-2.549274	-1.305379	-0.504513
C	-3.779012	-1.910049	-0.234654
C	-4.616979	-1.435685	0.770141
C	-4.245353	-0.328205	1.527082
C	-3.030535	0.295428	1.271556
H	5.099214	-2.610778	-1.136958
H	4.374761	-3.203510	0.380070
H	3.559854	-3.503012	-1.164991
H	3.956340	-0.650208	-0.612807
H	1.692587	0.471225	-0.847455
H	1.602752	1.559024	1.548013
H	2.454955	0.116339	2.095223
H	3.349932	2.456488	-0.018516
H	-0.006609	-1.212748	0.715536
H	-4.086706	-2.763869	-0.831575
H	-5.567231	-1.926686	0.954333
H	-4.899180	0.054124	2.303380
C	-1.664277	-1.828952	-1.609955
H	-2.237650	-2.463692	-2.288999
H	-1.234695	-1.011652	-2.197765
H	-0.834249	-2.425014	-1.215162
H	-2.732863	1.169795	1.842244

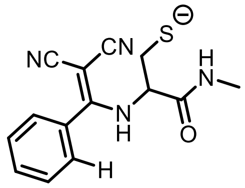

E (vac) = -1233.54834848

E (H_2O) = -1233.62741613

C	3.636064	-1.322352	-1.271548
C	2.720666	-0.291595	-1.085201
C	1.716496	-0.410092	-0.120696
C	1.634009	-1.567064	0.657481
C	2.556911	-2.592309	0.471184
C	3.558086	-2.473109	-0.490027
H	4.413125	-1.221631	-2.022758
H	2.784617	0.611229	-1.684316
H	0.829200	-1.683321	1.375354
H	2.481854	-3.490702	1.075555
H	4.274912	-3.276609	-0.632254
C	0.755387	0.717256	0.062593
C	1.253888	1.957165	0.482201
C	0.396211	3.074306	0.681388
C	2.628458	2.140599	0.795183
N	-0.306563	3.983924	0.860447
N	3.748941	2.321913	1.054350
N	-0.533446	0.527289	-0.190744
H	-1.186549	1.326332	-0.214393
C	-1.190169	-0.682793	-0.678442
H	-0.428114	-1.398625	-0.991316
C	-2.089052	-0.309008	-1.876749
H	-2.683673	-1.196155	-2.126719
H	-1.423190	-0.114946	-2.725942
C	-1.934396	-1.361346	0.493312
S	-3.158965	1.149003	-1.579235
O	-1.426137	-2.308456	1.104045
N	-3.129378	-0.839627	0.797950
H	-3.469109	-0.065159	0.196699
C	-3.924417	-1.360146	1.883664
H	-3.374569	-1.311364	2.829603
H	-4.832654	-0.759990	1.964574
H	-4.199051	-2.407019	1.711236

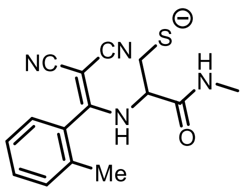

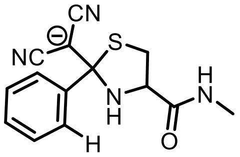

E (vac) = -1272.86037406

E (H_2O) = -1272.94118219

C	2.83267	-1.98506	0.430254
C	1.893322	-0.978547	0.662049
C	1.545421	-0.149596	-0.411971
C	2.130582	-0.317006	-1.667144
C	3.067586	-1.322126	-1.876240
C	3.415234	-2.160752	-0.820433
H	3.103915	-2.647018	1.247992
H	1.843868	0.346667	-2.477915
H	3.519541	-1.449522	-2.854838
H	4.140173	-2.955242	-0.971151
C	0.531567	0.933730	-0.233219
C	0.958311	2.169609	0.258683
C	0.059011	3.249755	0.475955
C	2.324629	2.377488	0.592704
N	-0.671551	4.136699	0.656905
N	3.437182	2.559947	0.883216
N	-0.725130	0.675562	-0.563969
H	-1.481308	1.373697	-0.444568
C	-1.270351	-0.610736	-0.992188
H	-0.544251	-1.123901	-1.626019
C	-2.572404	-0.328414	-1.782468
H	-3.069657	-1.291845	-1.942161
H	-2.277577	0.052127	-2.767499
C	-1.515114	-1.558864	0.194672
S	-3.680612	0.873416	-0.956374
O	-0.968873	-2.660883	0.255863
N	-2.350066	-1.079601	1.139788
H	-2.955082	-0.298994	0.831111
C	-2.757768	-1.912101	2.247678
H	-3.347127	-2.776632	1.917457
H	-1.877796	-2.290474	2.776033
H	-3.357457	-1.310484	2.933505
C	1.300322	-0.775071	2.031489
H	1.442566	-1.665961	2.647730
H	1.781886	0.073565	2.530467
H	0.227933	-0.565691	1.984436

E (vac) = -1233.54166315

E (H_2O) = -1233.62580049

C	-1.74413	3.094296	0.419772
C	-1.017257	2.855295	-0.748391
C	-0.119704	1.797928	-0.806814
C	0.076764	0.954948	0.295560
C	-0.639326	1.211744	1.459964
C	-1.548451	2.270564	1.521294
H	-2.446391	3.921818	0.469775
H	-1.143149	3.502064	-1.612177
H	0.456392	1.614614	-1.709027
H	-0.476769	0.586042	2.330695
H	-2.099227	2.447971	2.440617
C	2.346682	0.177348	-0.447091
C	3.116963	-0.769164	-1.135874
C	2.899888	1.428589	-0.139983
N	3.683817	-1.605437	-1.733116
N	3.347765	2.483479	0.105075
C	1.016366	-0.247131	0.102712
C	-0.613946	-2.050309	-0.122651
C	-0.309199	-2.173229	1.403405
H	-0.564097	-3.044284	-0.580166
H	-1.145585	-1.774914	1.981615
H	-0.169933	-3.217036	1.690940
S	1.228401	-1.262395	1.702446
N	0.355946	-1.198678	-0.798869
H	1.073273	-1.762940	-1.244083
C	-2.067555	-1.594860	-0.338096
O	-2.984486	-2.062026	0.335373
N	-2.235834	-0.713636	-1.345296
H	-1.370550	-0.335754	-1.714075
C	-3.484729	-0.027756	-1.576336
H	-3.674047	0.063596	-2.649957
H	-4.285586	-0.609430	-1.116741
H	-3.468932	0.973097	-1.128925

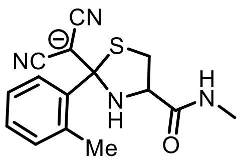

E (vac) =	-1272.84996728
E (H_2O) =	-1272.93154578

C	-1.64171	3.022355	0.932328
C	-1.066518	2.821497	-0.319300
C	-0.195833	1.760114	-0.579304
C	0.094640	0.851574	0.466690
C	-0.473589	1.076908	1.719431
C	-1.335449	2.144307	1.961460
H	-2.313303	3.860504	1.096646
H	-1.288960	3.516907	-1.125701
H	-1.759550	2.279765	2.952002
C	2.348128	0.005803	-0.288770
C	3.079171	-0.917863	-1.045805
C	2.925226	1.234775	0.052095
N	3.609334	-1.730114	-1.706834
N	3.391022	2.278130	0.314743
C	0.982853	-0.382003	0.195291
C	-0.730713	-2.089003	-0.152334
C	-0.482591	-2.264863	1.378451
H	-0.695087	-3.071033	-0.636387
H	-1.279770	-1.777697	1.944404
H	-0.471043	-3.320549	1.654965
S	1.134955	-1.529625	1.716817
N	0.292261	-1.251069	-0.767803
H	0.990889	-1.830768	-1.223079
C	-2.159628	-1.579429	-0.399160
O	-3.113226	-2.039495	0.225963
N	-2.264714	-0.655362	-1.376885
H	-1.376038	-0.305019	-1.712665
C	-3.477195	0.086940	-1.623936
H	-4.308856	-0.455531	-1.171199
H	-3.417617	1.086873	-1.177645
H	-3.652420	0.184782	-2.699595
H	-0.223539	0.399272	2.527788
C	0.405545	1.680836	-1.963844
H	0.533978	0.654368	-2.309786
H	-0.221700	2.227568	-2.676414
H	1.400202	2.137723	-1.962412

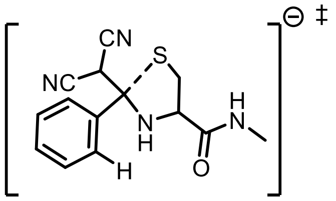

E (vac) =	-1233.53618432
E (H_2O) =	-1233.61438582

C	-1.83176	3.178237	-0.12407
C	-0.976064	2.955713	0.955445
C	-0.081913	1.892381	0.934018
C	-0.027174	1.036104	-0.167278
C	-0.875206	1.268891	-1.251543
C	-1.777695	2.330781	-1.226683
H	-2.534154	4.006880	-0.103332
H	-1.004837	3.613080	1.819291
H	0.571854	1.710704	1.780086
H	-0.833341	0.603157	-2.106962
H	-2.438622	2.489544	-2.073955
C	2.352003	0.304972	-0.345831
C	3.321828	-0.682386	-0.631367
C	2.810045	1.612433	-0.068223
N	4.066465	-1.540883	-0.902973
N	3.213794	2.684221	0.156557
C	0.977081	-0.080415	-0.255344
C	-0.343750	-2.160667	-0.496311
C	0.132712	-2.620953	0.900733
H	-0.332259	-3.002382	-1.195238
H	-0.654349	-3.212338	1.380633
H	1.008032	-3.265864	0.766875
S	0.561602	-1.169686	1.892440
N	0.604827	-1.187315	-1.024398
H	1.420360	-1.665873	-1.389980
C	-1.799500	-1.657957	-0.566345
O	-2.455388	-1.801598	-1.597913
N	-2.297553	-1.095194	0.552551
H	-1.623202	-0.873147	1.290815
C	-3.590286	-0.451398	0.546048
H	-3.580107	0.450753	-0.077707
H	-4.348770	-1.132938	0.151976
H	-3.847810	-0.174025	1.570082

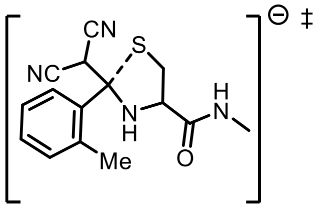

E (vac) = -1272.84327303

E (H_2O) = -1272.92006458

C	2.143453	2.899911	-0.33946
C	1.807288	2.397703	0.911688
C	0.803394	1.435697	1.084673
C	0.149126	0.944352	-0.063484
C	0.468017	1.481522	-1.312078
C	1.454398	2.447902	-1.460412
H	2.924364	3.649404	-0.434117
H	2.323322	2.769484	1.793595
H	1.675711	2.845951	-2.446048
C	-2.287531	0.535456	0.168460
C	-3.380018	-0.263165	0.568083
C	-2.546060	1.860657	-0.243455
N	-4.228092	-0.977424	0.939585
N	-2.779597	2.955556	-0.575515
C	-0.980226	-0.062744	0.075615
C	0.088106	-2.234758	0.531650
C	-0.430617	-2.766008	-0.825612
H	0.002438	-3.007920	1.300754
H	0.315561	-3.423739	-1.283237
H	-1.336262	-3.353239	-0.640519
S	-0.796612	-1.350409	-1.889055
N	-0.758481	-1.133965	0.954954
H	-1.633242	-1.485674	1.328712
C	1.575365	-1.836540	0.572157
O	2.202583	-1.899375	1.628662
N	2.128150	-1.436663	-0.592580
H	1.475338	-1.228734	-1.350408
C	3.428875	-0.804093	-0.606513
H	3.741815	-0.668599	-1.643554
H	3.395902	0.173774	-0.110760
H	4.155139	-1.432890	-0.086239
H	-0.066529	1.122951	-2.183264
C	0.457790	1.010064	2.490859
H	-0.625049	0.969282	2.634476
H	0.858236	0.017476	2.712922
H	0.876373	1.725754	3.205153

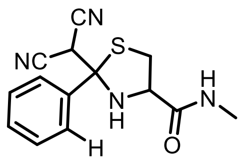

E (vac) = -1234.06363389

E (H_2O) = -1234.09425124

C	2.556464	-2.463676	-1.028872
C	1.573106	-1.486066	-0.890443
C	1.635679	-0.569321	0.159048
C	2.703166	-0.634737	1.058472
C	3.690736	-1.602695	0.912630
C	3.617659	-2.524160	-0.131094
C	0.587676	0.538994	0.264728
C	1.215708	1.866652	-0.277852
C	1.686778	1.715983	-1.666249
N	2.072666	1.594174	-2.749189
C	0.283881	3.003630	-0.188905
N	-0.459498	3.887545	-0.131700
H	2.495735	-3.170678	-1.850104
H	0.756415	-1.415689	-1.600350
H	2.758368	0.058464	1.893726
H	4.513344	-1.642280	1.619425
H	4.386071	-3.282371	-0.243137
C	-1.613121	0.266591	1.739971
C	-1.526677	-0.501526	0.401293
N	-0.611079	0.220691	-0.460158
S	0.088407	0.811828	2.069490
H	-1.099907	1.041937	-0.816063
H	-2.276299	1.129260	1.637719
H	-1.954495	-0.369220	2.559442
H	-1.127614	-1.506777	0.583282
H	2.090092	2.099212	0.340365
C	-2.892778	-0.558617	-0.279008
O	-3.328323	0.412019	-0.882258
N	-3.587657	-1.707540	-0.119325
C	-4.909397	-1.875111	-0.696973
H	-5.368683	-2.769435	-0.274443
H	-5.525544	-1.005769	-0.459910
H	-4.857626	-1.971938	-1.785641
H	-3.132086	-2.499870	0.304028

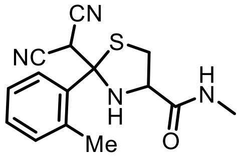

E (vac) =	-1273.36823406		
E (H_2O) =	-1273.39862664		
C	-3.689931	-1.613101	-0.425838
C	-2.805539	-0.546423	-0.615181
C	-1.537871	-0.613600	-0.001074
C	-1.225820	-1.718948	0.796026
C	-2.120700	-2.767782	0.970847
C	-3.362525	-2.718375	0.350391
C	-0.473856	0.488658	-0.133170
H	-4.665950	-1.567699	-0.901650
C	-3.311865	0.622770	-1.432820
H	-1.848020	-3.610435	1.597879
H	-4.076423	-3.526521	0.473683
C	1.674344	0.386989	-1.723900
C	1.681268	-0.486512	-0.453319
N	0.770771	0.122558	0.494434
S	-0.073358	0.804303	-1.960455
H	1.255452	0.905725	0.930556
H	2.272078	1.288873	-1.567906
H	2.033823	-0.151443	-2.603512
H	1.326799	-1.494378	-0.703084
C	3.074295	-0.528339	0.172235
O	3.471167	0.390507	0.875015
N	3.835453	-1.598439	-0.150356
H	3.416636	-2.362459	-0.655758
C	5.190413	-1.738339	0.352908
H	5.194779	-1.959396	1.424492
H	5.685657	-2.546612	-0.186272
H	5.738976	-0.808065	0.193533
C	-1.003320	1.811287	0.517298
H	-1.908025	2.121726	-0.012071
C	-1.374177	1.601898	1.927888
C	-0.033940	2.913575	0.403332
N	-1.690216	1.434581	3.027344
N	0.736328	3.771321	0.312941
H	-0.267589	-1.731921	1.301587
H	-4.057022	0.280361	-2.154653
H	-2.536493	1.137220	-2.002953
H	-3.811310	1.359433	-0.791010

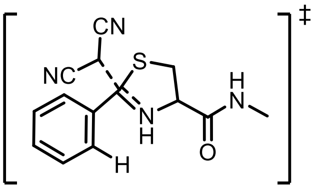

E (vac) =	-1234.03091663		
E (H_2O) =	-1234.06601201		
C	4.055247	-0.903384	-0.168448
C	2.761750	-1.185115	0.252421
C	1.664401	-0.607867	-0.398597
C	1.888844	0.283558	-1.460869
C	3.183424	0.571863	-1.865500
C	4.269200	-0.027056	-1.226475
C	0.305998	-0.922202	0.044138
C	0.294605	1.005475	1.677328
C	1.628920	1.076206	2.131672
N	2.737860	1.038825	2.489378
C	-0.175674	1.867020	0.681410
N	-0.645229	2.416940	-0.240402
H	4.895206	-1.351078	0.350410
H	2.613126	-1.841293	1.103450
H	3.345735	1.272914	-2.677074
H	5.280560	0.202187	-1.545501
C	-1.814103	-1.896728	1.027180
C	-2.009266	-1.342767	-0.388391
N	-0.771318	-0.633134	-0.687424
S	-0.029361	-2.233776	1.167806
H	-0.703826	-0.020677	-1.487423
H	-2.098294	-1.158192	1.781661
H	-2.382338	-2.812008	1.182235
H	-2.127038	-2.167021	-1.102122
H	-0.442012	0.676571	2.404924
C	-3.306224	-0.528461	-0.462187
O	-4.363650	-1.144804	-0.403038
N	-3.208303	0.806276	-0.573752
C	-4.401838	1.633721	-0.596817
H	-4.934348	1.580845	0.357361
H	-4.099836	2.663371	-0.787940
H	-5.079426	1.296936	-1.384480
H	-2.308071	1.274858	-0.511612
H	1.066990	0.789938	-1.956491

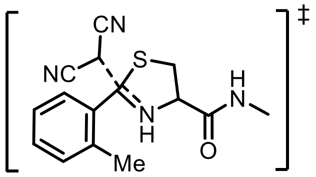

E (vac) = -1273.33738894

E (H_2O) = -1273.37027838

C	3.991534	-1.405419	-0.314461
C	2.680218	-1.449333	0.128911
C	1.717769	-0.541460	-0.341878
C	2.105413	0.470329	-1.249065
C	3.431362	0.484821	-1.686762
C	4.367402	-0.437458	-1.240289
C	0.337122	-0.704175	0.145620
C	0.621948	1.121282	1.838416
C	1.848809	1.779764	1.585652
N	2.872320	2.263883	1.310243
C	-0.564448	1.779262	1.498735
N	-1.571325	2.224150	1.103175
H	4.712987	-2.122534	0.061472
H	2.400457	-2.214943	0.844802
H	3.739574	1.265476	-2.375312
H	5.390862	-0.384552	-1.596358
C	-1.853351	-1.673968	1.047106
C	-1.937871	-1.193180	-0.396314
N	-0.747951	-0.358718	-0.561799
S	-0.075306	-1.999984	1.280643
H	-0.651164	0.204871	-1.393283
H	-2.183419	-0.903874	1.749312
H	-2.418070	-2.592709	1.199840
H	-1.856968	-2.054411	-1.071255
H	0.592301	0.463170	2.699963
C	-3.293067	-0.554951	-0.727830
O	-4.196895	-1.305803	-1.078757
N	-3.405604	0.775578	-0.605780
C	-4.688719	1.422134	-0.814222
H	-5.433479	1.065243	-0.096083
H	-4.551653	2.496437	-0.690142
H	-5.059145	1.213553	-1.820470
H	-2.683632	1.304769	-0.110755
C	1.186177	1.562561	-1.738758
H	1.775561	2.413566	-2.083748
H	0.579087	1.236413	-2.594944
H	0.522310	1.936945	-0.954867

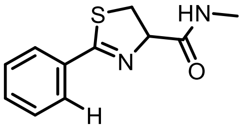

E (vac) = -1009.15254093

E (H_2O) = -1009.17036291

C	4.155393	-0.134154	0.677601
C	2.874068	-0.668482	0.573805
C	1.861012	0.051402	-0.066268
C	2.147711	1.311831	-0.604438
C	3.427550	1.842051	-0.499519
C	4.434424	1.120455	0.142029
C	0.489607	-0.487602	-0.185547
H	4.934282	-0.698518	1.179921
H	2.660678	-1.641412	1.005702
H	3.642815	2.818010	-0.922699
H	5.434341	1.535294	0.221725
C	-1.594767	-1.899856	0.072086
C	-1.743353	-0.532018	-0.621270
N	-0.522399	0.241366	-0.451763
S	0.194097	-2.238775	0.003416
H	-1.919644	-1.859661	1.114558
H	-2.151072	-2.681311	-0.442542
H	-1.890130	-0.682308	-1.701077
C	-2.994187	0.192890	-0.124899
O	-4.064067	-0.402751	-0.064205
N	-2.812241	1.488034	0.208065
C	-3.883147	2.337387	0.682590
H	-4.000342	3.211182	0.034910
H	-3.690264	2.674314	1.705704
H	-4.806216	1.756778	0.668626
H	-1.868452	1.841883	0.130098
H	1.357592	1.858939	-1.107068

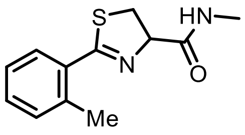

E (vac) = -1048.45967782

E (H_2O) = -1048.47639835

C	3.853104	0.429887	-1.263685
C	2.549643	0.817350	-0.979181
C	1.763932	0.097303	-0.070961
C	2.299269	-1.027587	0.590192
C	3.616745	-1.389629	0.296419
C	4.388045	-0.681336	-0.620193
C	0.374239	0.548028	0.169851
H	4.443892	0.993231	-1.978266
H	2.123935	1.682521	-1.477850
H	4.047817	-2.247721	0.804695
H	5.407129	-0.994806	-0.824386
C	-1.742780	1.941479	0.185483
C	-1.853391	0.470370	0.631155
N	-0.619365	-0.236298	0.318528
S	0.039536	2.301183	0.289468
H	-2.084954	2.075907	-0.843578
H	-2.307236	2.607503	0.835745
H	-1.988451	0.427637	1.722118
C	-3.094835	-0.187841	0.029908
O	-4.178639	0.382700	0.083859
N	-2.888982	-1.401635	-0.523374
C	-3.947872	-2.181245	-1.126948
H	-4.042045	-3.153871	-0.635062
H	-3.758657	-2.337148	-2.193384
H	-4.881580	-1.630092	-1.009027
H	-1.936186	-1.739724	-0.518564
C	1.523078	-1.832519	1.601684
H	0.994740	-1.188586	2.309472
H	0.764461	-2.450412	1.114853
H	2.197042	-2.484991	2.162443

Phage Display Screening

Modification of phage surface proteins

A mixture of TCEP (2 μL, 50 mM) and CX_9CX_4C phage library (98 μL in PBS, pH 7.4, ~10^{13} pfu/mL) was gently shaken at 37 °C for 30 min. Then an ice-cold PEG/NaCl solution (20% v/v) was added to the reaction mixture, and the precipitated phages were collected by centrifugation and resuspended in $NaHCO_{3(aq)}$ (93 μL, 0.1 M). The CX_9CX_4C library solution was then prepared by addition of TCEP (2 μL, 50 mM), Ac-Cys-OMe (4 μL, 50 mM) and **1q** (1 μL, 50 mM). The resulting mixture was incubated at 37 °C for 1 h. An ice-cold PEG/NaCl solution (20% v/v) was then added to the reaction mixture, and the precipitated phages were collected by centrifugation and resuspended in PBS (99.5 μL, pH 7.4). After that, GSSG (0.5 μL, 100 mM) was added to the phage solution, and the oxidation reaction was carried out at 37 °C for 2 h. Finally, an ice-cold PEG/NaCl solution (20% v/v) was added to the reaction mixture, and the precipitated phages were collected by centrifugation and resuspended in PBS (1 mL, pH 7.4). The obtained modified phage solution was stored at 4 °C for the phage selection.

Binder selection by phage display

CX_9CX_4C phage library was modified by the *o*-TAMM **1q**. After the reaction, the modified phage was incubated with 0.5 mM GSSG at 37 °C for two hours. The oxidized phages were recovered through precipitation with ice-cold PEG/NaCl solution (20% v/v). The precipitated phages were resuspended in 1 mL PBS. The methods of phage screening and protein biotinylation were the same as described previously.[5-6]

(1) Protein biotinylation

After thawing the stored protein at 4 °C, an aliquot of the protein stock solution was transferred into a 1.5 mL centrifuge tube. A 5-fold molar excess of Sulfo-NHS-LC-Biotin (ThermoFisher, #21335) dissolved in 1× PBS was then added, and the mixture was incubated on a shaker at room temperature for 30 min. Excess biotin was removed by ultrafiltration. The resulting biotinylated target protein was quantified using a NanoDrop and stored at −80 °C for future use.

(2) Phage screening

100 μL streptavidin-coated magnetic beads (ThermoFisher, #11205D; neutravidin-coated magnetic beads for the second round, Biomag Biotechnology, #BMJ2800-2) were washed three times with binding buffer (10 mM Tris-Cl, 150 mM NaCl, 10 mM $MgCl_2$, 1 mM $CaCl_2$, pH 7.4) in a 1.5 mL microcentrifuge tube and the washed beads were resuspended completely (the tube was removed from the magnet) with 100 μL binding buffer, followed by equal distribution into two 1.5 mL microcentrifuge tubes. Then the biotinylated target protein (1st round 5 μg, 2nd round 5 μg and 3rd round 2 μg) was added to one of the two microcentrifuge tubes and the same volume of 1× PBS (without protein) was added to the other one. The tubes were incubated on a slowly rotating wheel for 15 min at room temperature. These beads were washed three times with the binding buffer to remove unbound proteins and resuspended with 1 mL blocking buffer (binding buffer with 0.3% v/v Tween-20 and 3% w/v BSA), then incubated on a slowly rotating wheel at room temperature for 2 hours. In parallel, the modified phage library (10^{11}~10^{12} pfu.) dissolved in 900 μL 1× PBS was blocked by addition of 2 mL blocking buffer and incubated on a slowly rotating wheel at room temperature for 2 hours. Then the blocked phages were split equally into two 10 mL tubes, into which the blocked beads with and without immobilized proteins were added, respectively. The two tubes were incubated on a slowly rotating wheel at room temperature for 30 min. After that, the unbound phages in the supernatant were removed (the co-incubated solution was transferred to 1.5 mL

tubes placed in a magnet). The beads were washed nine times with the washing buffer (binding buffer with 0.1% v/v Tween-20) and twice with binding buffer. During the period, the tubes were replaced at least three times. The buffer was removed completely in the last washing step, and then 200 μL×2 elution buffer (50 mM glycine, pH 2.2) was added to elute the phages bound to the beads by resuspending the beads with a mini-vortex mixer and incubating for 5 min twice. Then the supernatant was transferred into a 1.5 mL microcentrifuge tube containing 25 μL×2 neutralization buffer (1 M Tris-Cl, pH 8.0). The eluted phages were diluted and used to infect exponentially growing TG1 cells for monitoring the phage titer, followed by amplification and purification. The purified phage was further modified with Me-TAMM and oxidized by GSSG for the next round of selection.

Preparation of next-generation sequencing (NGS) samples

Phage vectors were extracted from E. coli TG1 cells using a commercial plasmid purification kit (CWBIO, #29124). First, the phage vector DNA was amplified by PCR using the junction primers (primer sequences, 5′→3′:
NGS-F1:
TCGTCGGCAGCGTCAGATGTGTATAAGAGACAGTTCTATGCGGCCCAGCCGGCCATG
NGS-R1:
GTCTCGTGGGCTCGGAGATGTGTATAAGAGACAGCTTTCAACAGTCTATGCGGC).
The purified PCR products were then further amplified by PCR using primers designed to introduce distinct barcodes. (Primers sequence 5'→3':
i5-index-1:
AATGATACGGCGACCACCGAGATCTACACCCAACTTCTCGTCGGCAGCGTCAGATGT
i7-index-1:
CAAGCAGAAGACGGCATACGAGATGTTGTTCGGTCTCGTGGGCTCGGAGATG
i5-index-2:
AATGATACGGCGACCACCGAGATCTACACGTGGTATGTCGTCGGCAGCGTCAGATGT
i7-index-2:
CAAGCAGAAGACGGCATACGAGATCGGTTGTTGTCTCGTGGGCTCGGAGATG
i5-index-3:
AATGATACGGCGACCACCGAGATCTACACGTCAACAGTCGTCGGCAGCGTCAGATGT
i7-index-3:
CAAGCAGAAGACGGCATACGAGATACTGAGGTGTCTCGTGGGCTCGGAGATG).
The PCR reaction mixture consisted of PrimeSTAR DNA polymerase (GenStar, #A064-10), 500 nM of each primer, 10 ng of phage vector DNA, and sterile water to a final volume of 20 μL. The PCR amplification was carried out under the following conditions: initial denaturation at 95 °C for 5 min, followed by 35 cycles of 98 °C for 15 seconds, 55 °C for 1 minute, 72 °C for 30 seconds, and a final extension at 72 °C for 5 min. The PCR products were separated by 3% agarose gel electrophoresis and purified using a commercial gel extraction kit (Omega Bio-tek, #D2500-03). Sequencing was performed by Novogene.

Surface plasmon resonance (SPR) assay

Biotinylated proteins were immobilized onto the CAPture chip (Cytiva, #28920234). A range of peptide concentrations in the running buffer (50 μM EDTA, 0.05% v/v Tween 20, PBS, pH 7.4) were then introduced at a flow rate of 30 μL/min using the automated sample handling system. Following the experiment, sensorgram data were analyzed using the Biacore 8K Evaluation software, applying a 1:1 binding model for K_D estimation.

Supplementary Figures

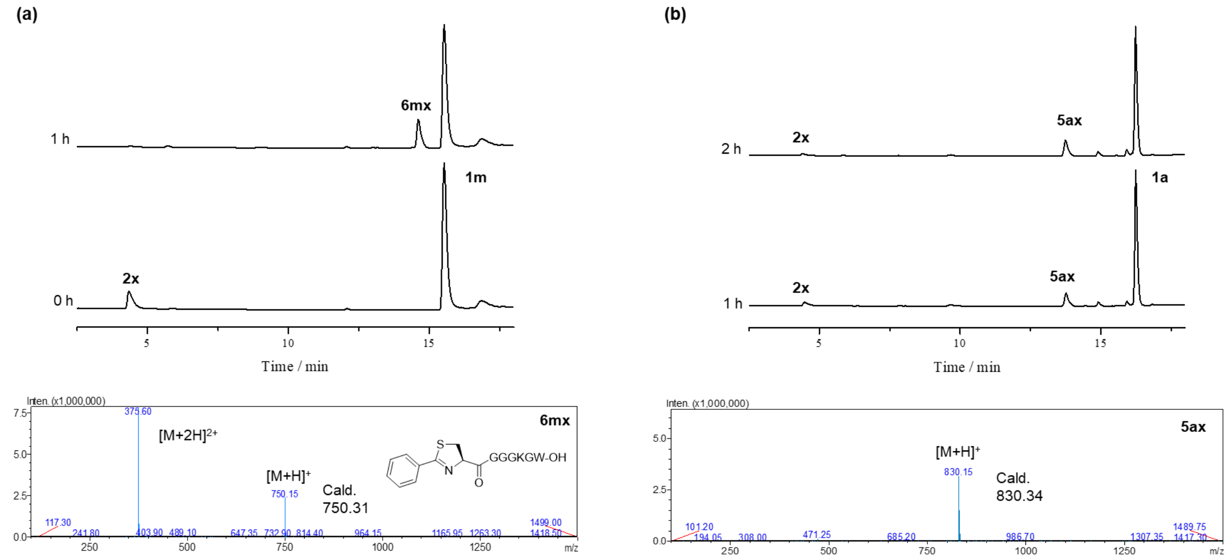

Figure S1. Different reactivity of unsubstituted and ortho-substituted TAMMs

HPLC chromatograms of reactions between 50 μM of **2x** and 400 μM of unsubstituted TAMM **1m** (a) or **1a** (b) at 37 °C in PBS containing 500 μM TCEP and 500 μM Ac-Cys-OMe under chromatography condition A.

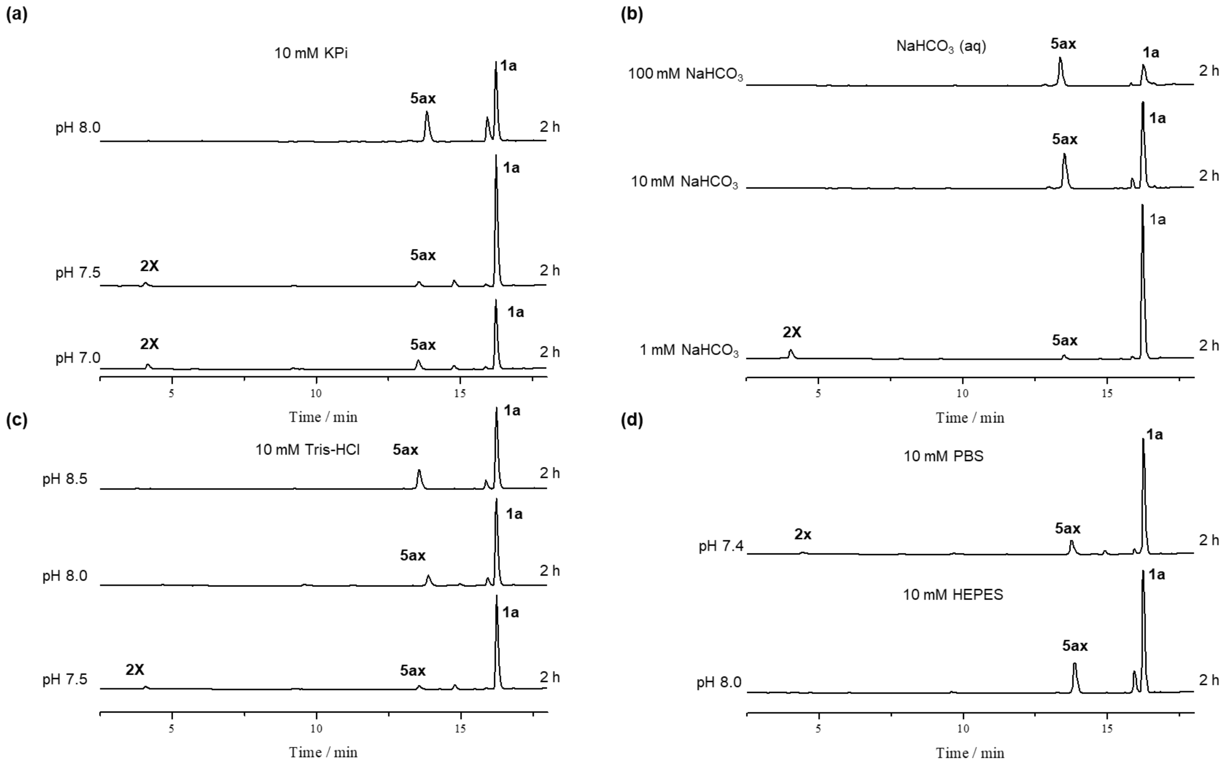

Figure S2. Effect of media compositions and pH values for reactions with 1a

HPLC chromatograms of reactions between 50 μM **2x** and 400 μM **1a** at 37 °C in 10 mM KPi (a), 10 mM Tris-HCl (b), the indicated concentrations of $NaHCO_{3(aq)}$ (c) or 10 mM PBS / HEPES (d) containing 500 μM TCEP and 500 μM Ac-Cys-OMe under chromatography condition A.

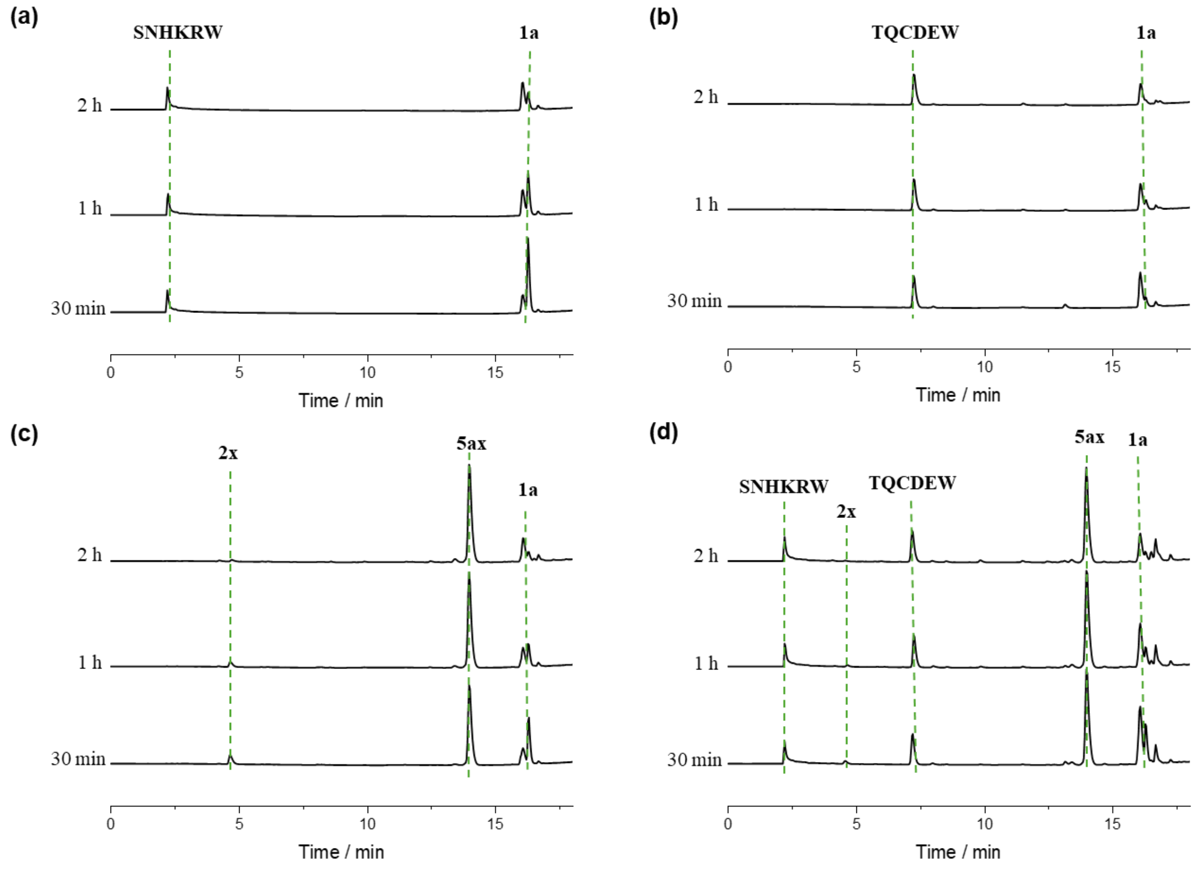

Figure S3. Reaction of 1a with peptides SNHKRW, TQCDEW, and 2x

Conversions were quantified by HPLC for reactions of 200 μM 1a with 50 μM peptide SNHKRW (a), peptide TQCDEW (b), or peptide **2x** (c) in the presence of 500 μM TCEP and 500 μM Ac-Cys-OMe under 37 °C in 0.1 M $NaHCO_{3(aq)}$. (d) HPLC chromatograms of the reaction of 600 μM 1a and 50 μM each of peptides SNHKRW, TQCDEW, and **2x** in the presence of 1.5 mM TCEP and 1.5 mM Ac-Cys-OMe under 37 °C in 0.1 M $NaHCO_{3(aq)}$. HPLC chromatograms showing the reactions under chromatography condition A.

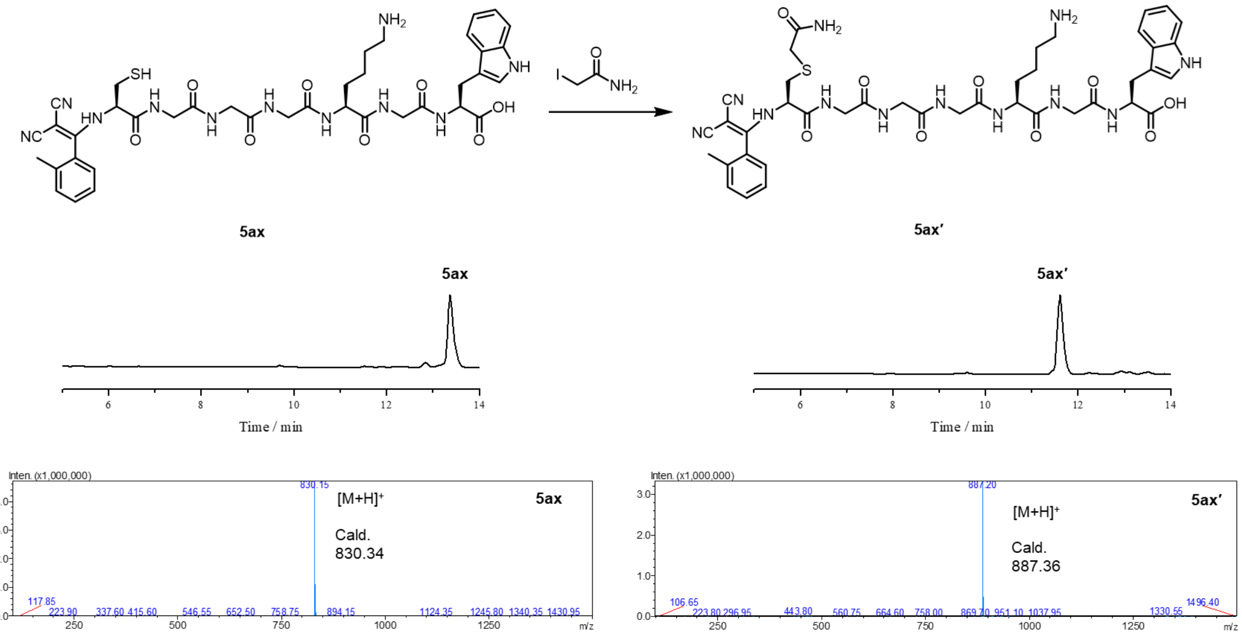

Figure S4. Reaction of 5ax with iodoacetamide

HPLC chromatograms and mass spectra of the reaction between 50 μM **5ax** and 1 mM iodoacetamide in 0.1 M $NaHCO_{3(aq)}$ containing 500 μM TCEP under chromatography condition A.

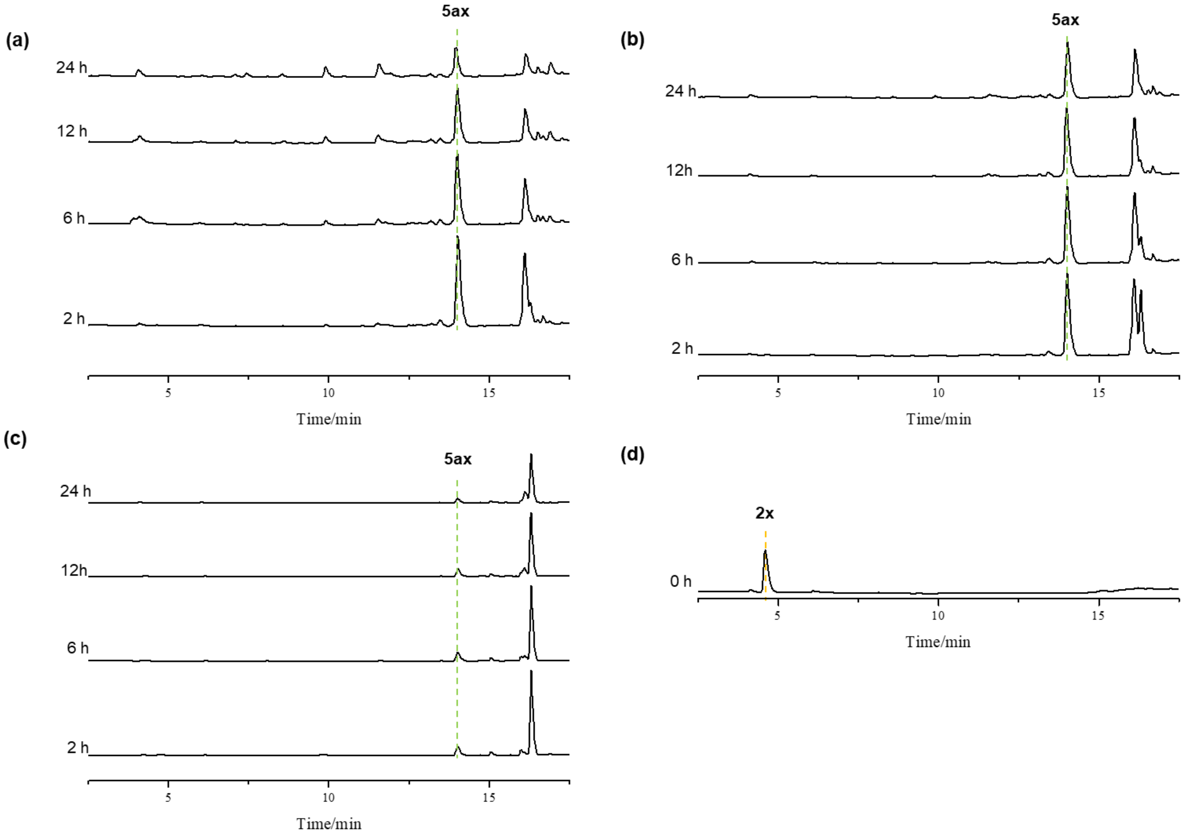

Figure S5. Reaction of 1a and 2x and stability of 5ax in the reaction system

Conversions were quantified by HPLC for reactions of 50 µM peptide **2x** and 200 µM **1a** in the presence of 500 µM TCEP and 500 µM Ac-Cys-OMe under 37 °C in 0.1 M $NaHCO_{3(aq)}$ (a), 25 °C in 0.1 M $NaHCO_{3(aq)}$ (b), or 37 °C in PBS (c). (d) HPLC chromatogram of the starting material peptide 2x. HPLC chromatograms showing the reactions under chromatography condition A.

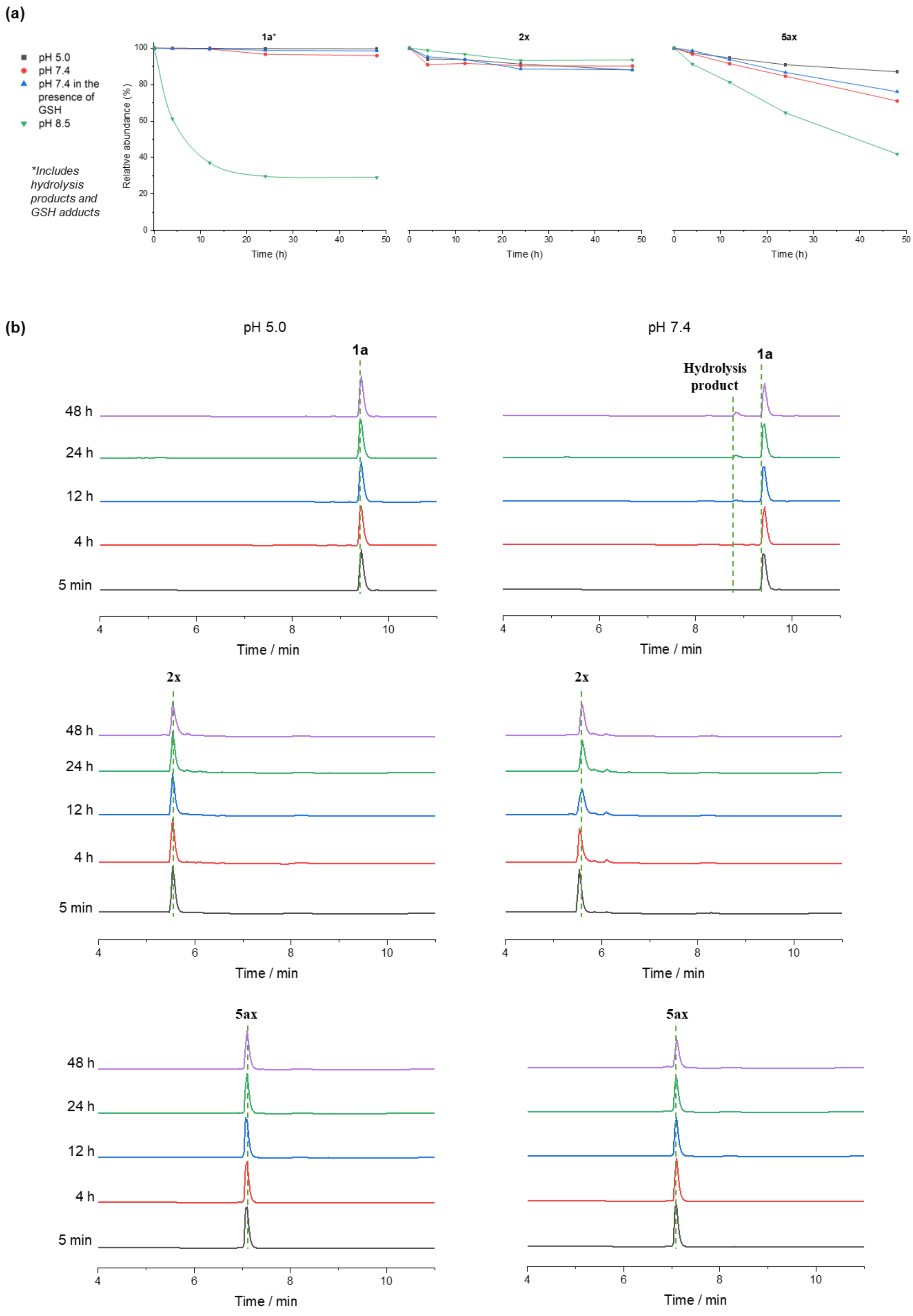
(a)
pH 5.0
pH 7.4
pH 7.4 in the presence of GSH
pH 8.5
*Includes hydrolysis products and GSH adducts
1a*
2x
5ax
Relative abundance (%)
Time (h)
(b)
pH 5.0
pH 7.4
1a
Hydrolysis product
48 h
24 h
12 h
4 h
5 min
Time / min
2x
5ax

(c)

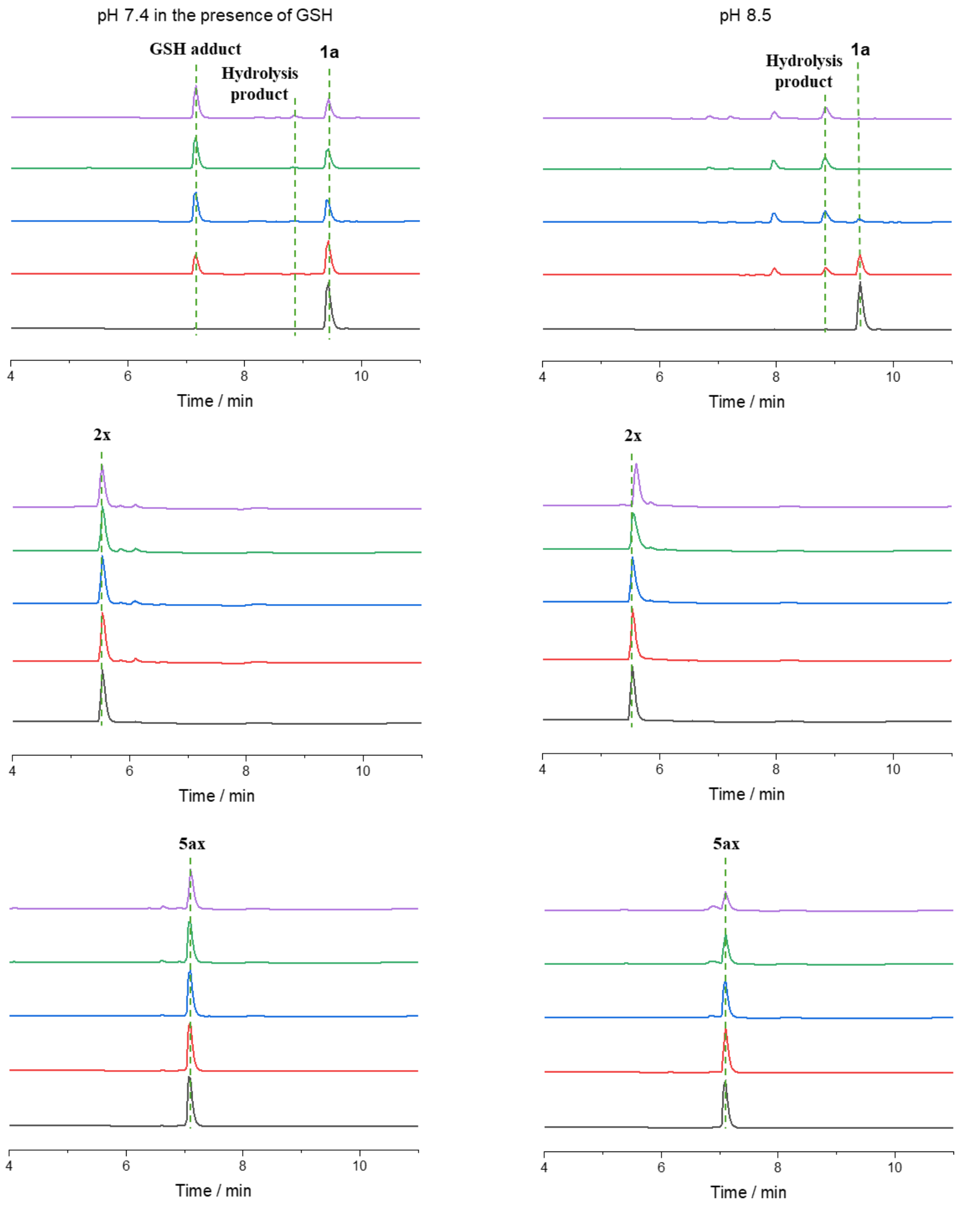

(d)

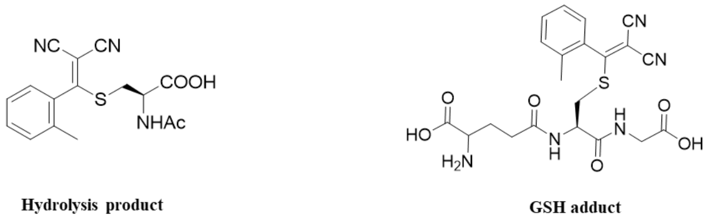

Figure S6. Time-dependent stability of 1a, 2x, and 5ax under different pH conditions

The stability of 50 μM **1a**, **2x**, and **5ax** in aqueous buffers at pH 5.0 (0.1 M sodium acetate/acetic acid buffer), pH 7.4 (PBS), pH 7.4 in the presence of 500 μM glutathione (GSH), or pH 8.5 (0.1 M Tris-HCl buffer) was evaluated by HPLC under chromatography condition D. The samples were kept at 25 °C for 48 h. The relative abundance (%) of the parent compounds was plotted over time, with the abundance at 5 min normalized to 100% (a). Representative HPLC chromatograms are shown in (b,c). Chemical structures of hydrolysis product and GSH adduct of **1a** are shown in (d).

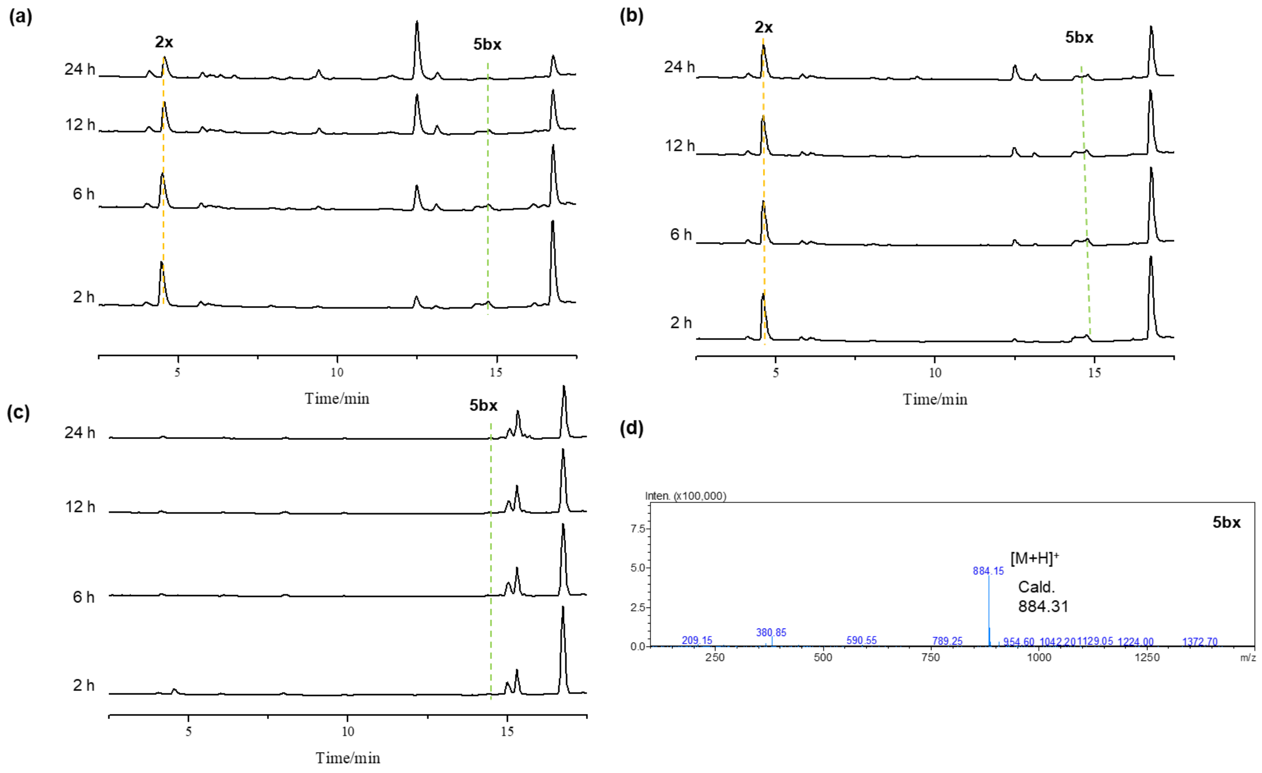

Figure S7. Reaction of 1b and 2x

Conversions were quantified by HPLC for reactions of 50 μM peptide **2x** and 200 μM **1b** in the presence of 500 μM TCEP and 500 μM Ac-Cys-OMe under 37 °C in 0.1 M $NaHCO_{3(aq)}$ (a), 25 °C in 0.1 M $NaHCO_{3(aq)}$ (b), or 37 °C in PBS (c). (d) The mass spectrum of **5bx**. HPLC chromatograms showing the reactions under chromatography condition A.

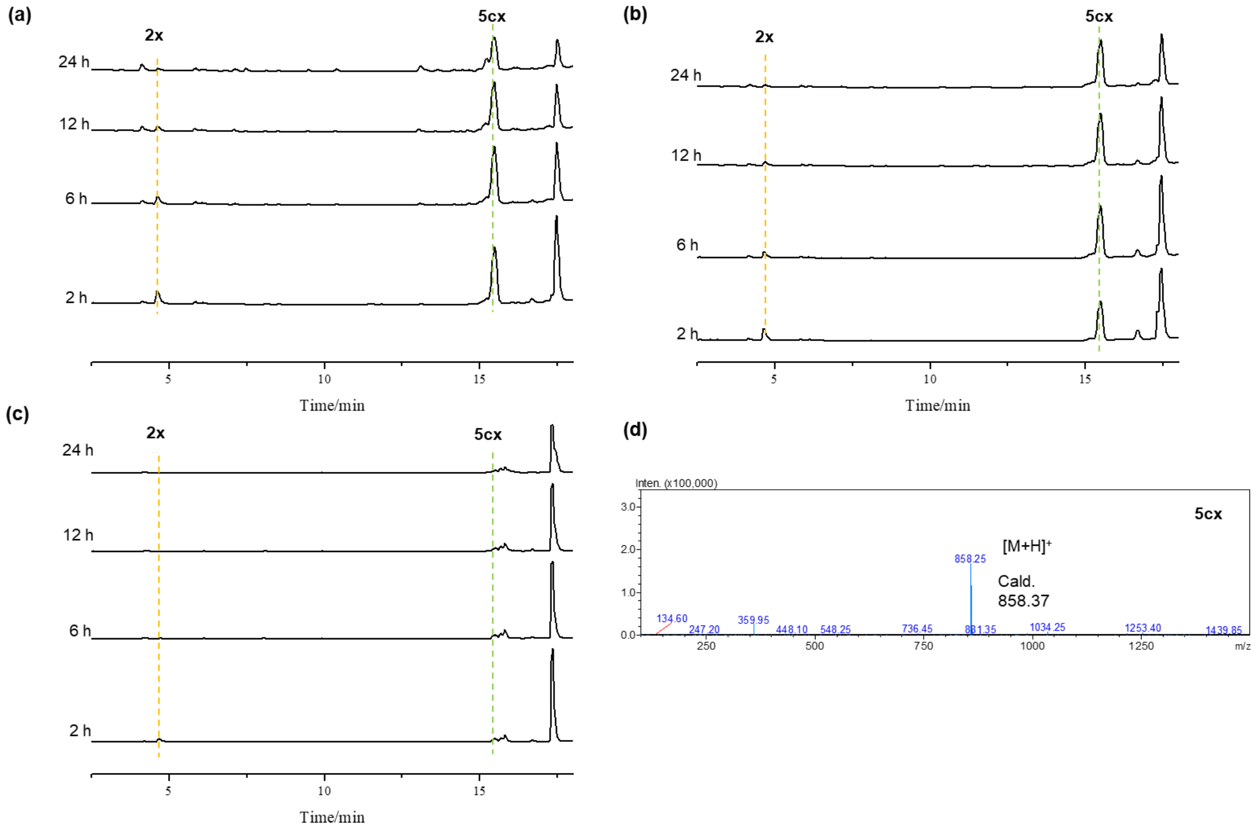

Figure S8. Reaction of 1c and 2x

Conversions were quantified by HPLC for reactions of 50 μM peptide **2x** and 200 μM **1c** in the presence of 500 μM TCEP and 500 μM Ac-Cys-OMe under 37 °C in 0.1 M $NaHCO_{3(aq)}$ (a), 25 °C in 0.1 M $NaHCO_{3(aq)}$ (b), or 37 °C in PBS (c). (d) The mass spectrum of **5cx**. HPLC chromatograms showing the reactions under chromatography condition A.

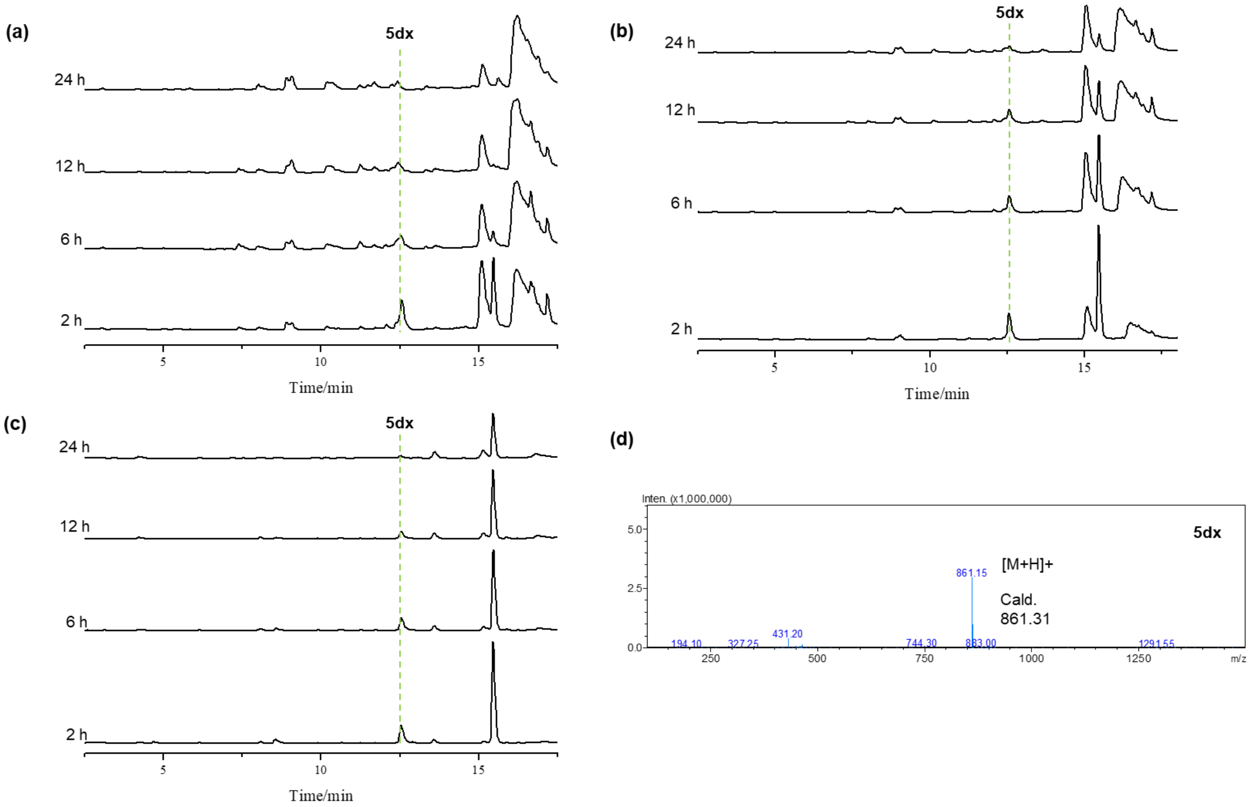

Figure S9. Reaction of 1d and 2x

Conversions were quantified by HPLC for reactions of 50 μM peptide **2x** and 200 μM **1d** in the presence of 500 μM TCEP and 500 μM Ac-Cys-OMe under 37 °C in 0.1 M $NaHCO_{3(aq)}$ (a), 25 °C in 0.1 M $NaHCO_{3(aq)}$ (b), or 37 °C in PBS (c). (d) The mass spectrum of **5dx**. HPLC chromatograms showing the reactions under chromatography condition A.

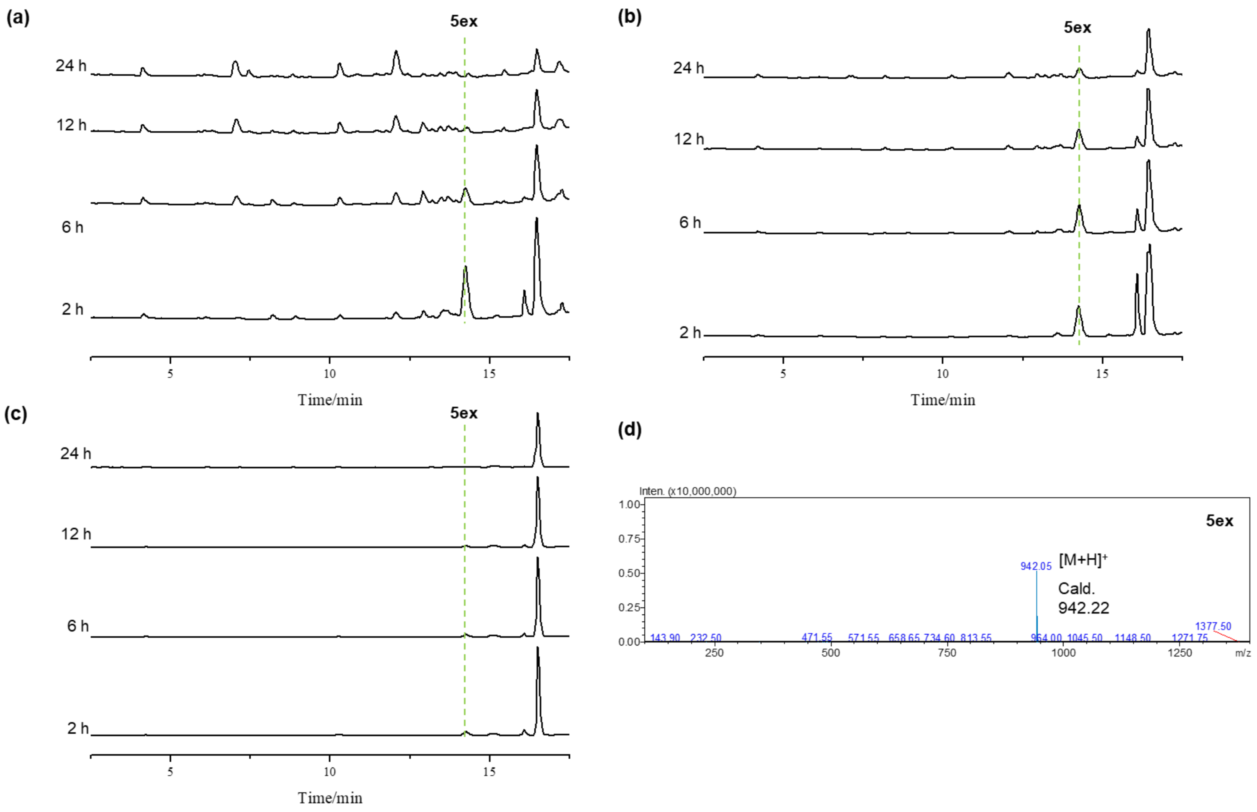

Figure S10. Reaction of 1e and 2x

Conversions were quantified by HPLC for reactions of 50 μM peptide **2x** and 200 μM **1e** in the presence of 500 μM TCEP and 500 μM Ac-Cys-OMe under 37 °C in 0.1 M $NaHCO_{3(aq)}$ (a), 25 °C in 0.1 M $NaHCO_{3(aq)}$ (b), or 37 °C in PBS (c). (d) The mass spectrum of **5ex**. HPLC chromatograms showing the reactions under chromatography condition A.

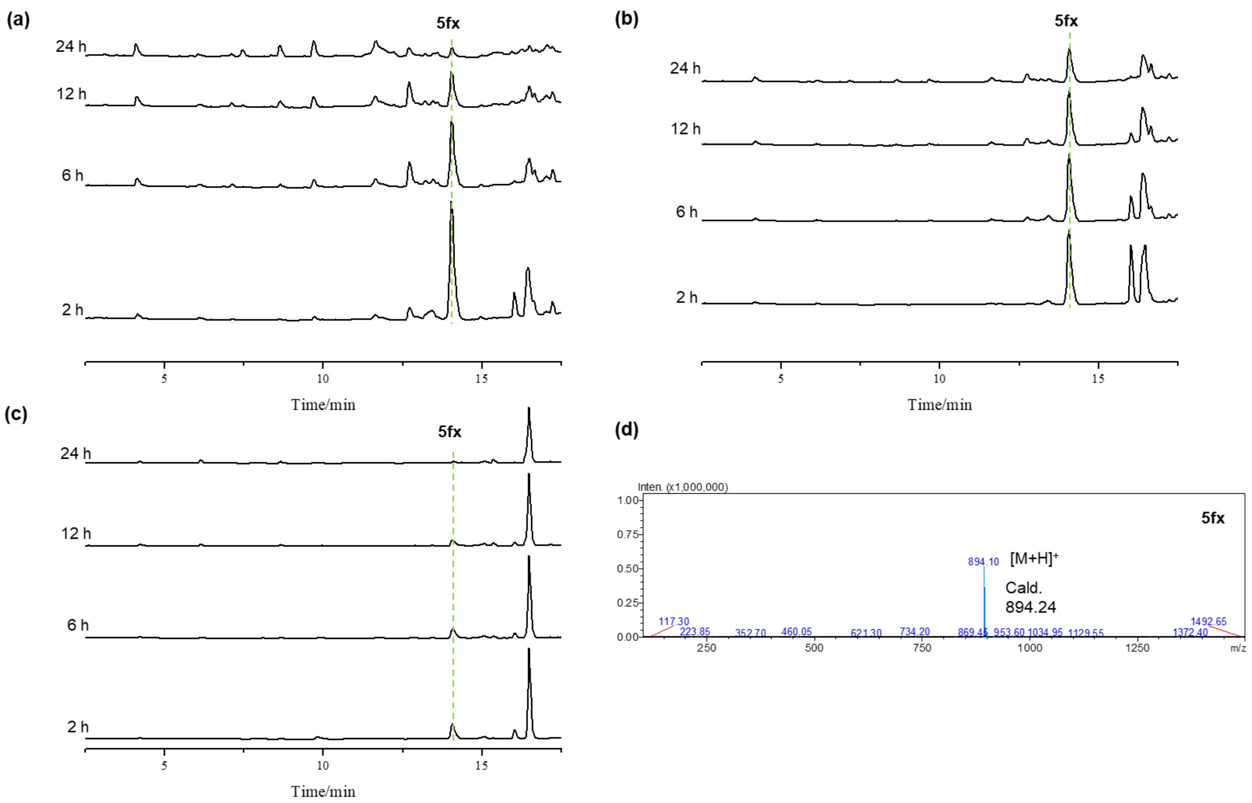

Figure S11. Reaction of 1f and 2x

Conversions were quantified by HPLC for reactions of 50 μM peptide **2x** and 200 μM **1f** in the presence of 500 μM TCEP and 500 μM Ac-Cys-OMe under 37 °C in 0.1 M $NaHCO_{3(aq)}$ (a), 25 °C in 0.1 M $NaHCO_{3(aq)}$ (b), or 37 °C in PBS (c). (d) The mass spectrum of **5fx**. HPLC chromatograms showing the reactions under chromatography condition A.

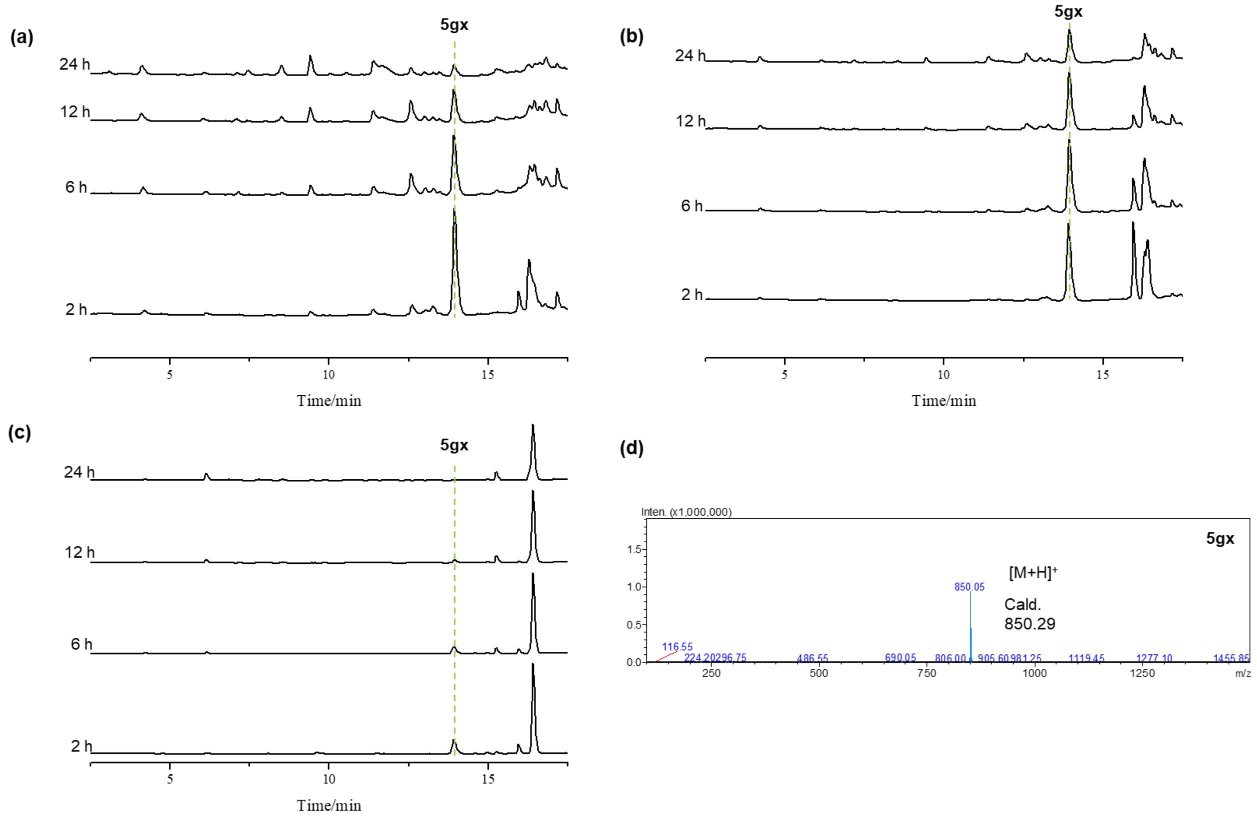

Figure S12. Reaction of 1g and 2x

Conversions were quantified by HPLC for reactions of 50 μM peptide **2x** and 200 μM **1g** in the presence of 500 μM TCEP and 500 μM Ac-Cys-OMe under 37 °C in 0.1 M $NaHCO_{3(aq)}$ (a), 25 °C in 0.1 M $NaHCO_{3(aq)}$ (b), or 37 °C in PBS (c). (d) The mass spectrum of **5gx**. HPLC chromatograms showing the reactions under chromatography condition A.

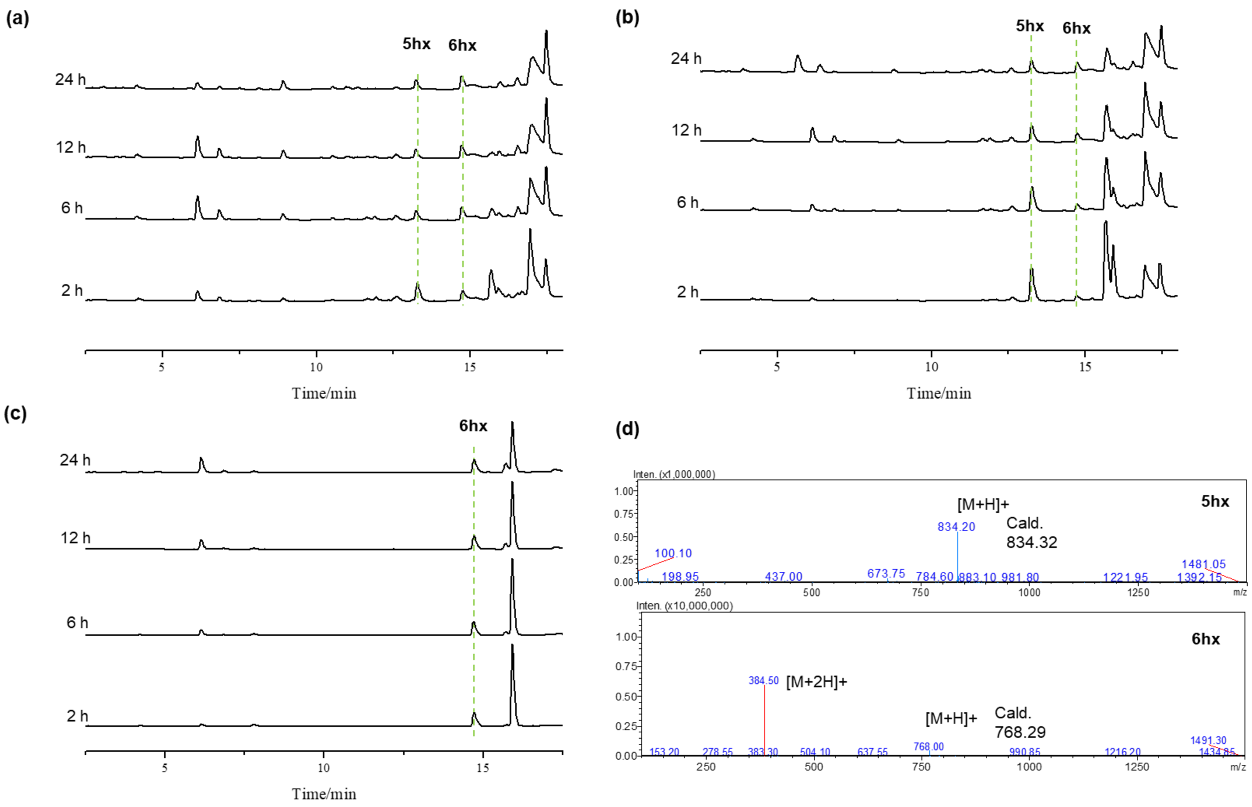

Figure S13. Reaction of 1h and 2x

Conversions were quantified by HPLC for reactions of 50 μM peptide **2x** and 200 μM **1h** in the presence of 500 μM TCEP and 500 μM Ac-Cys-OMe under 37 °C in 0.1 M $NaHCO_{3(aq)}$ (a), 25 °C in 0.1 M $NaHCO_{3(aq)}$ (b), or 37 °C in PBS (c). (d) The mass spectrum of **5hx** and **6hx**. HPLC chromatograms showing the reactions under chromatography condition A.

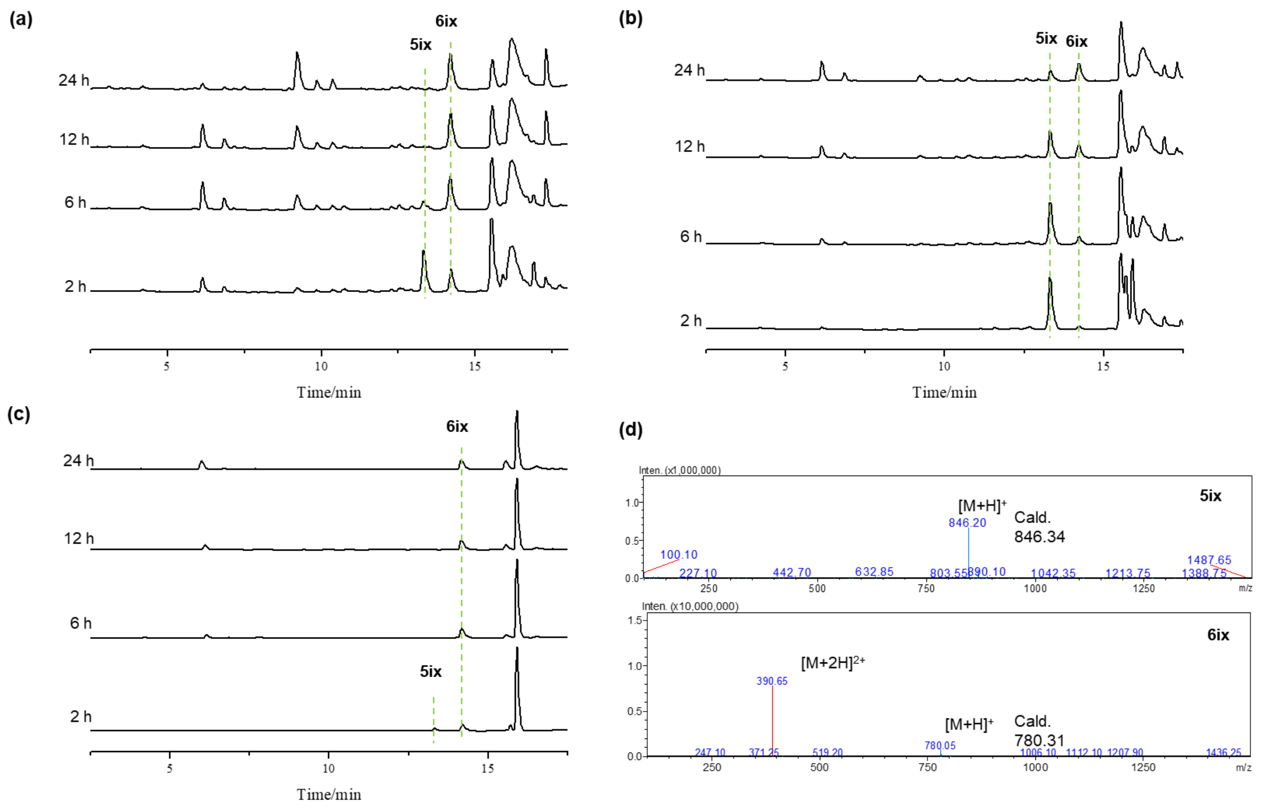

Figure S14. Reaction of 1i and 2x

Conversions were quantified by HPLC for reactions of 50 μM peptide **2x** and 200 μM **1i** in the presence of 500 μM TCEP and 500 μM Ac-Cys-OMe under 37 °C in 0.1 M $NaHCO_{3(aq)}$ (a), 25 °C in 0.1 M $NaHCO_{3(aq)}$ (b), or 37 °C in PBS (c). (d) The mass spectrum of **5ix** and **6ix**. HPLC chromatograms showing the reactions under chromatography condition A.

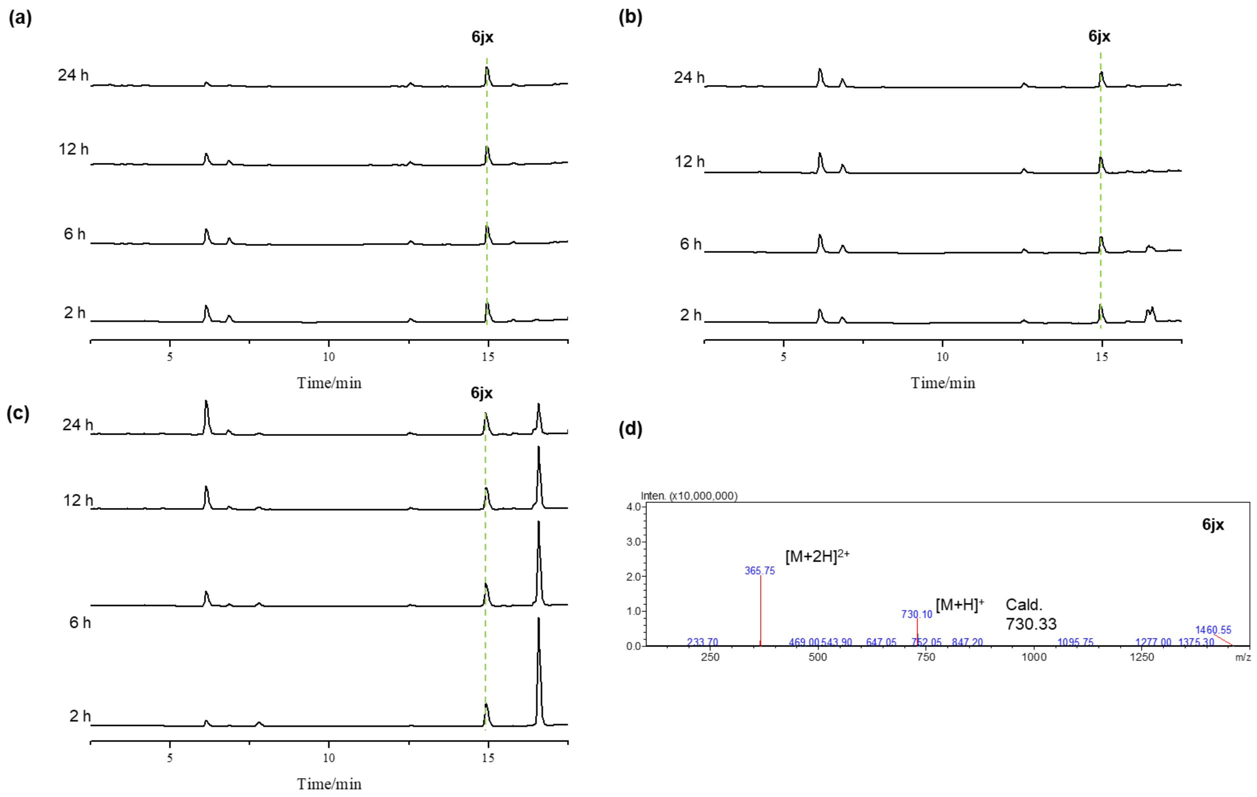

Figure S15. Reaction of 1j and 2x

Conversions were quantified by HPLC for reactions of 50 µM peptide **2x** and 200 µM **1j** in the presence of 500 µM TCEP and 500 µM Ac-Cys-OMe under 37 °C in 0.1 M $NaHCO_{3(aq)}$ (a), 25 °C in 0.1 M $NaHCO_{3(aq)}$ (b), or 37 °C in PBS (c). (d) The mass spectrum of **6jx**. HPLC chromatograms showing the reactions under chromatography condition A.

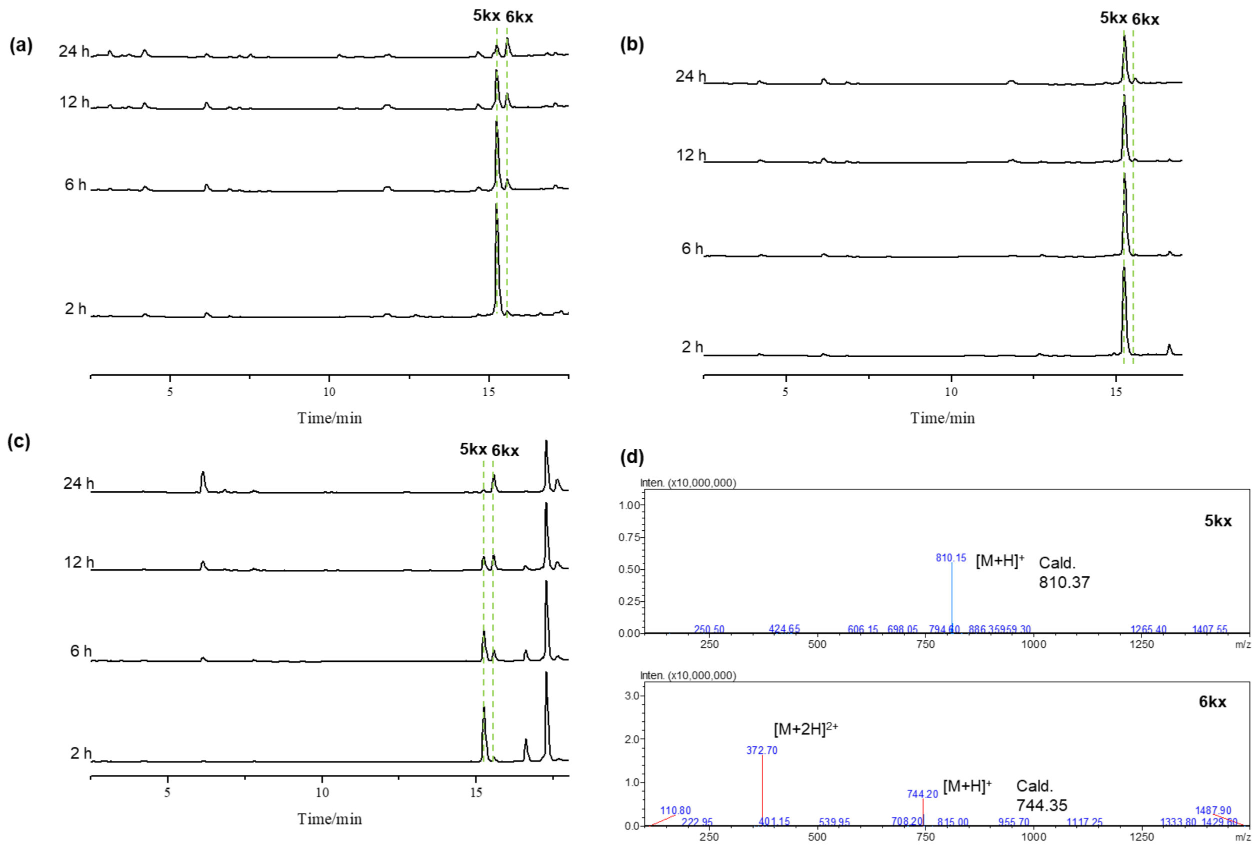

Figure S16. Reaction of 1k and 2x

Conversions were quantified by HPLC for reactions of 50 μM peptide **2x** and 200 μM **1k** in the presence of 500 μM TCEP and 500 μM Ac-Cys-OMe under 37 °C in 0.1 M $NaHCO_{3(aq)}$ (a), 25 °C in 0.1 M $NaHCO_{3(aq)}$ (b), or 37 °C in PBS (c). (d) The mass spectrum of **5kx** and **6kx**. HPLC chromatograms showing the reactions under chromatography condition A.

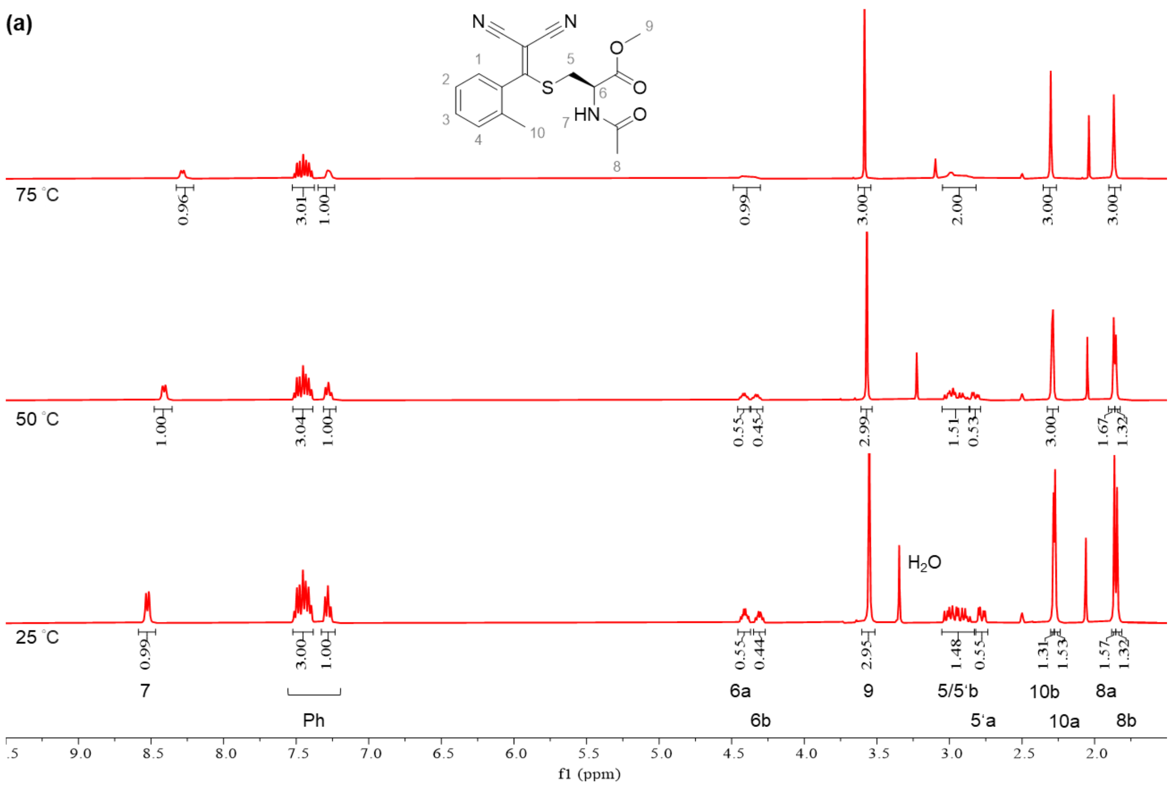

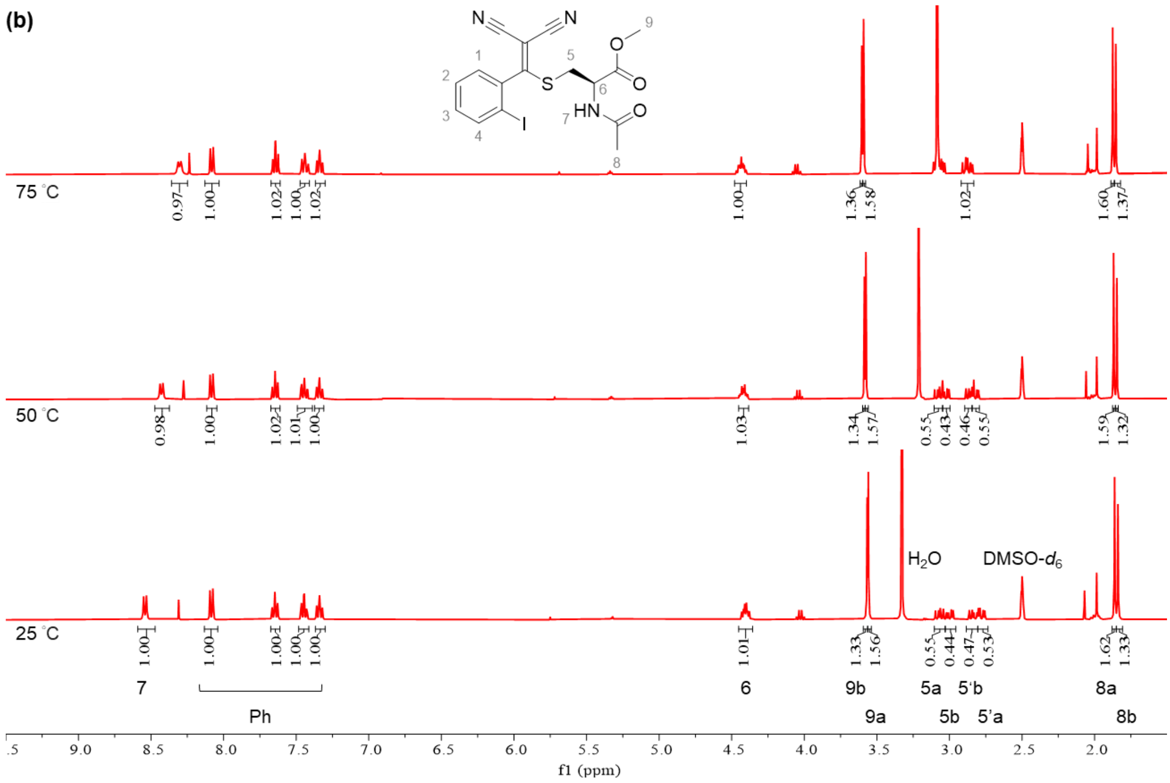

Figure S17. ^{1}H NMR of 1a and 1e at different temperatures

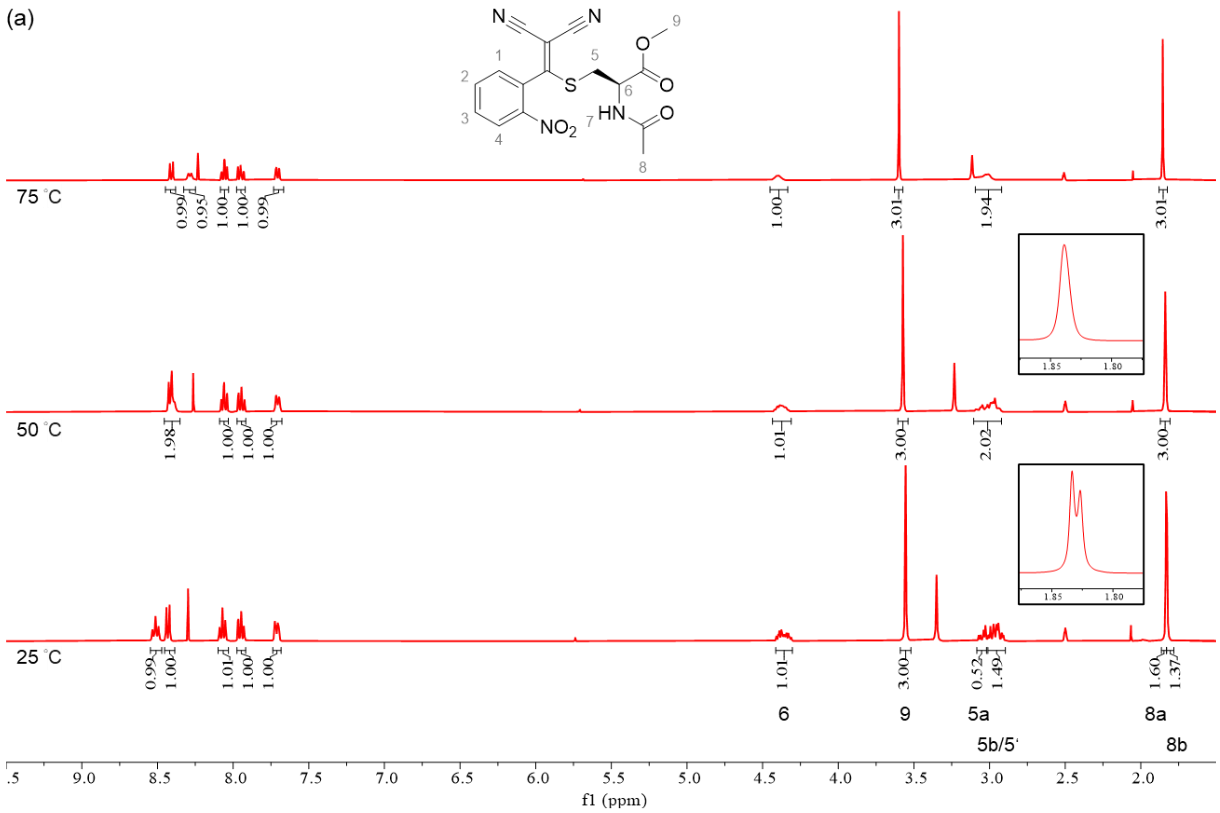

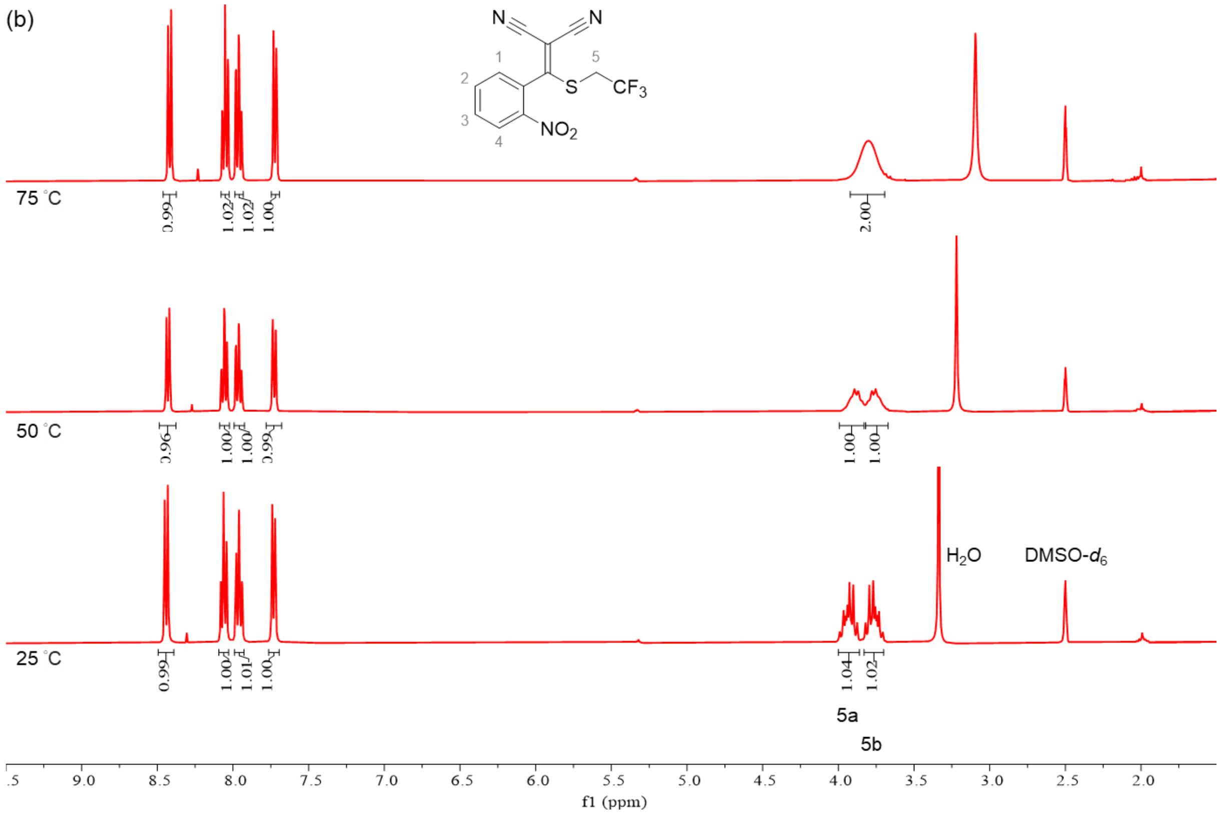

Figure S18. ^{1}H NMR of 1d and 1l at different temperatures

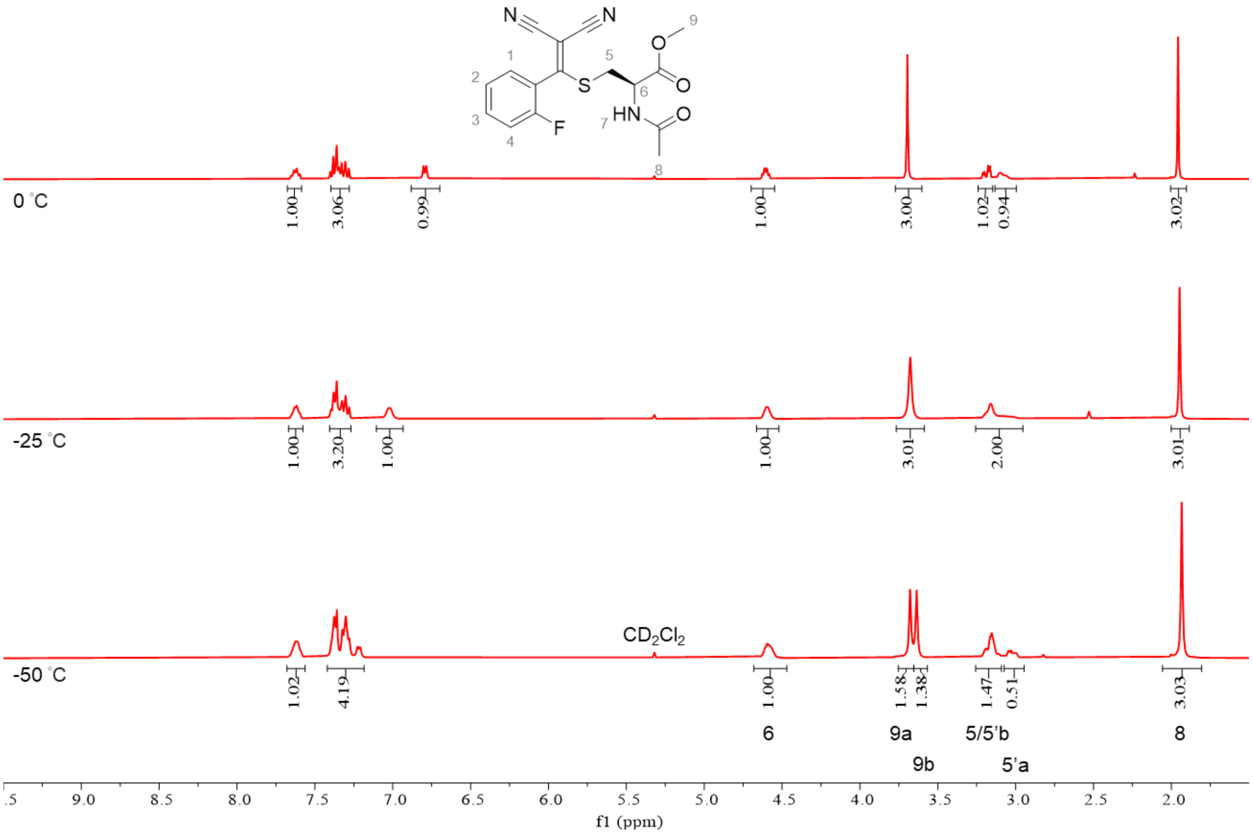

Figure S19. ^{1}H NMR of 1h at different temperatures

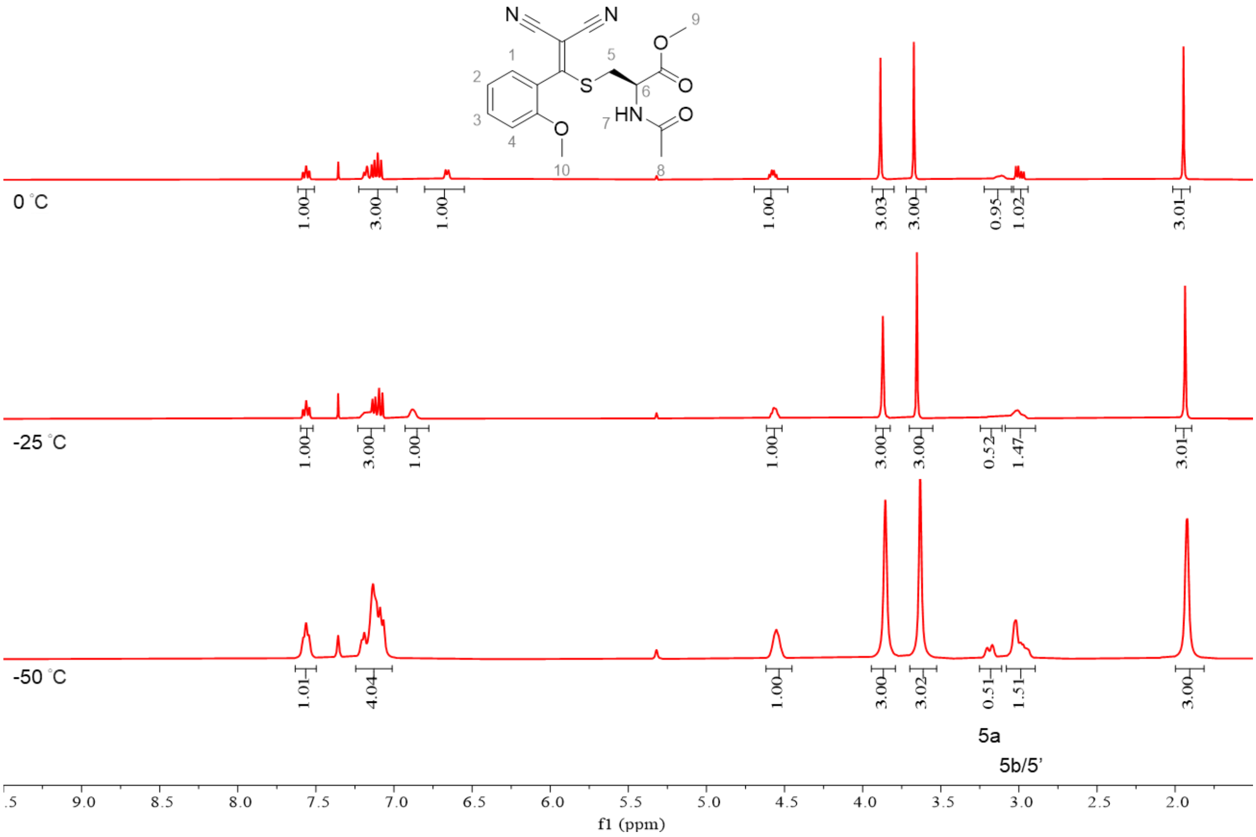

Figure S20. ^{1}H NMR of 1i at different temperatures

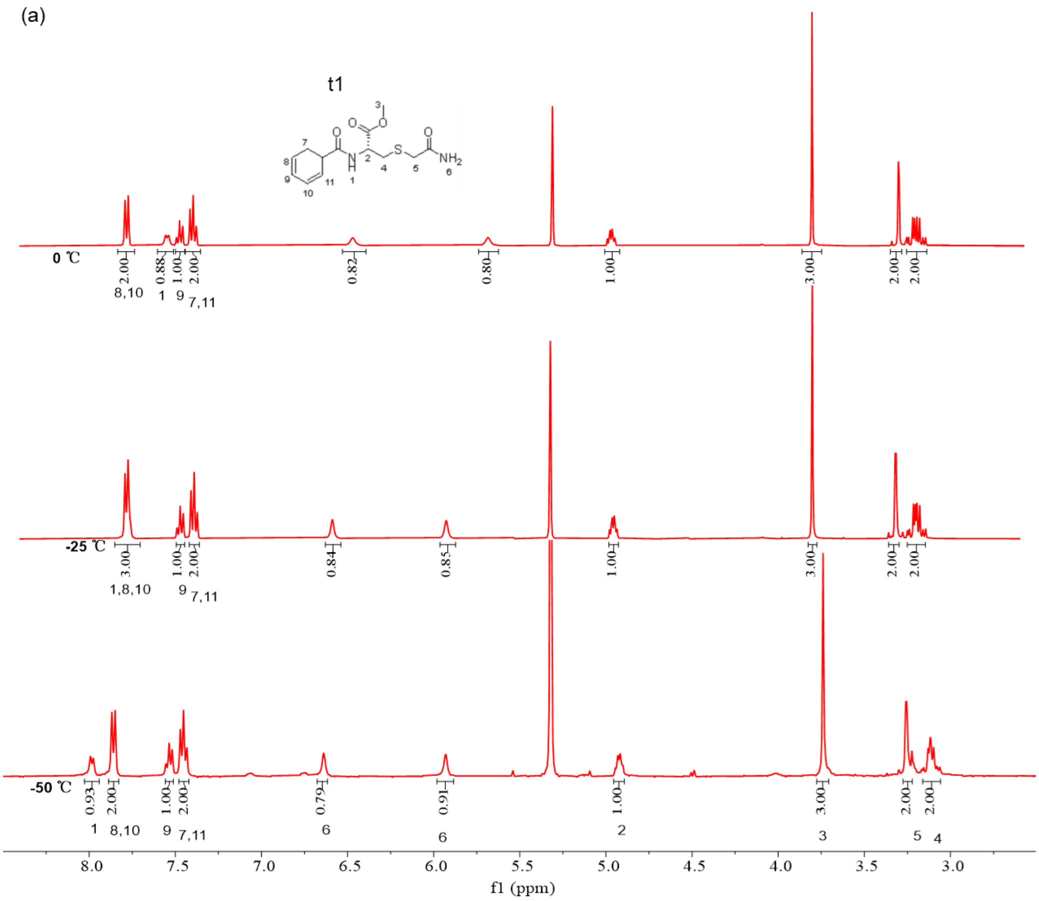
(a)
t1
0 ℃
-25 ℃
-50 ℃
2.00 0.88 1.00 2.00 0.82 0.80 1.00 3.00 2.00 2.00
8,10 1 9 7,11
3.00 1.00 2.00 0.84 0.85 1.00 3.00 2.00 2.00
1,8,10 9 7,11
0.93 2.00 1.00 2.00 0.79 0.91 1.00 3.00 2.00 2.00
1 8,10 9 7,11 6 6 2 3 5 4
8.0 7.5 7.0 6.5 6.0 5.5 5.0 4.5 4.0 3.5 3.0
f1 (ppm)

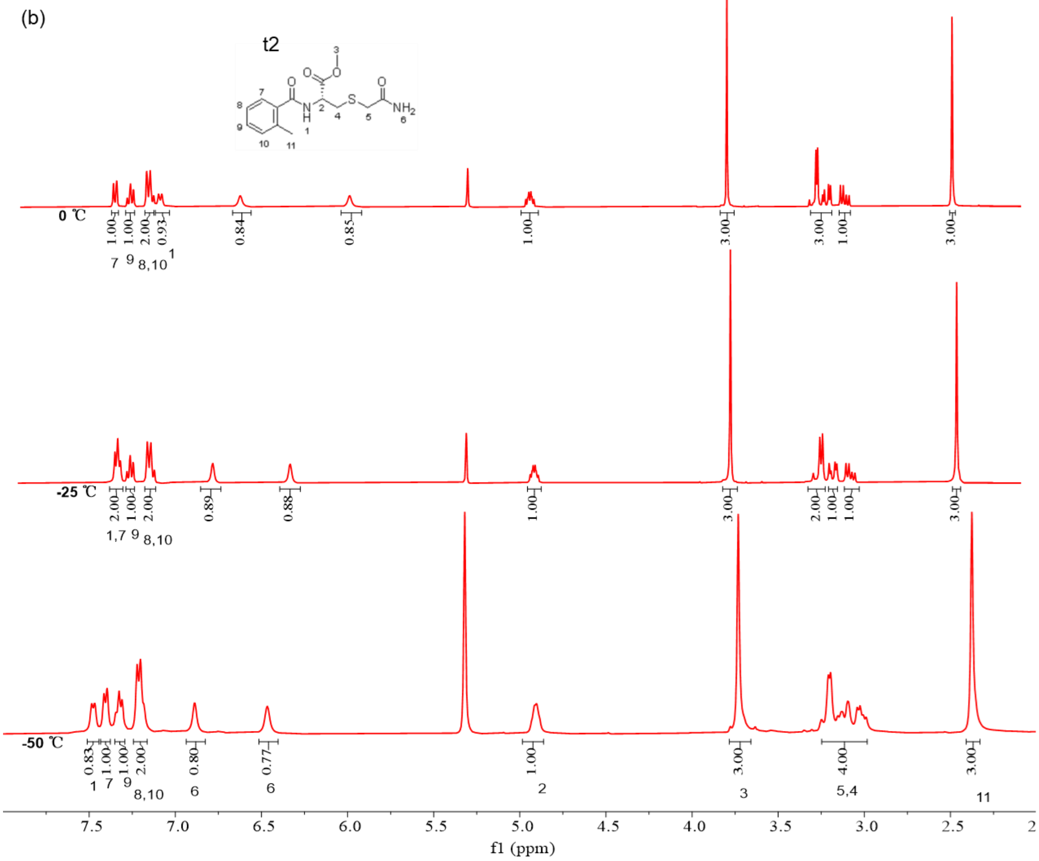
(b)
t2
0 ℃
-25 ℃
-50 ℃
1.00 1.00 2.00 0.93 0.84 0.85 1.00 3.00 3.00 1.00 3.00
7 9 8,10 1
2.00 1.00 2.00 0.89 0.88 1.00 3.00 2.00 1.00 1.00 3.00
1,7 9 8,10
0.83 1.00 1.00 2.00 0.80 0.77 1.00 3.00 4.00 3.00
1 7 9 8,10 6 6 2 3 5,4 11
7.5 7.0 6.5 6.0 5.5 5.0 4.5 4.0 3.5 3.0 2.5 2
f1 (ppm)

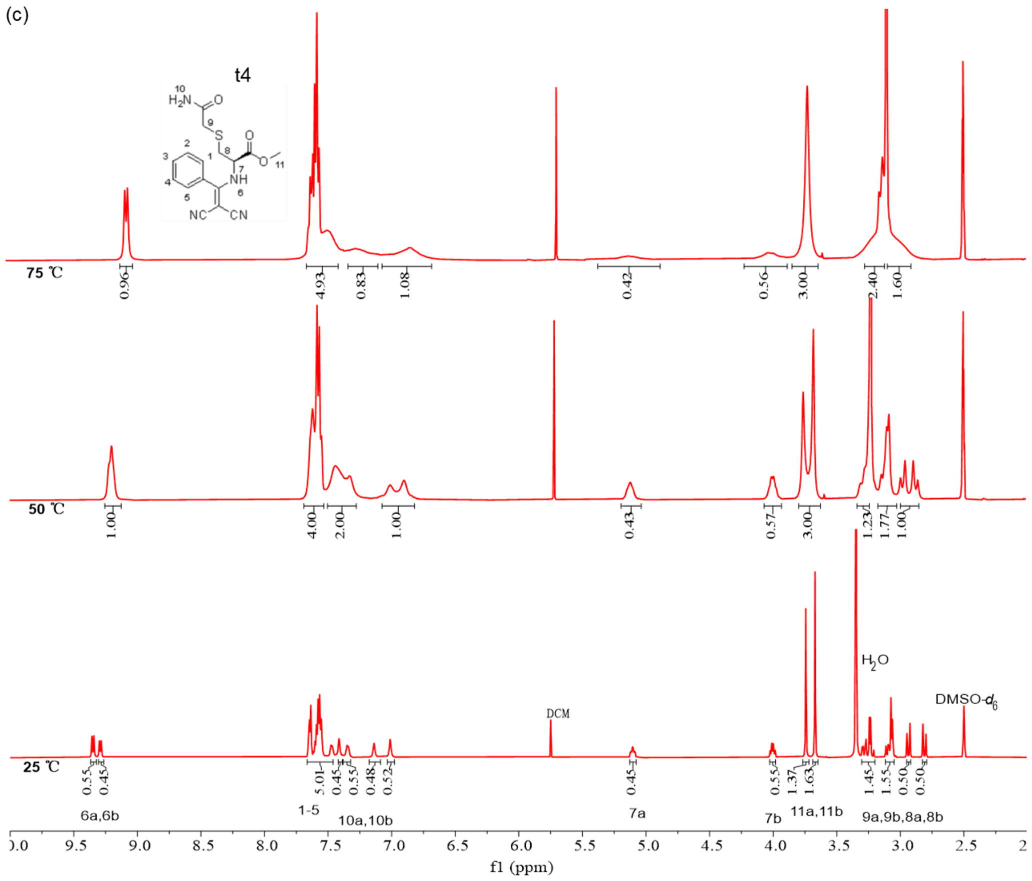

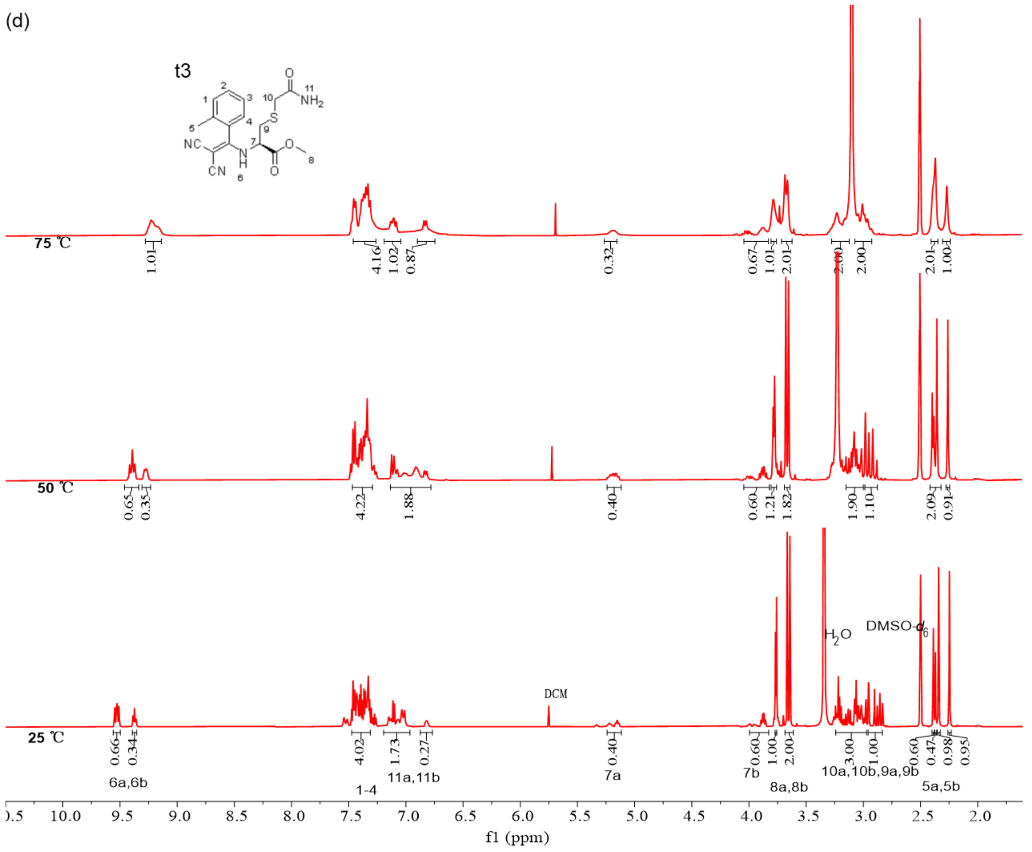

Figure S21. ^{1}**H NMR of t1, t2, t3, t4 at different temperatures**

Compounds **t1**, **t2** and **t3** were newly synthesized in the present work, and **t4** was prepared according to our previous work.[7]

(a)

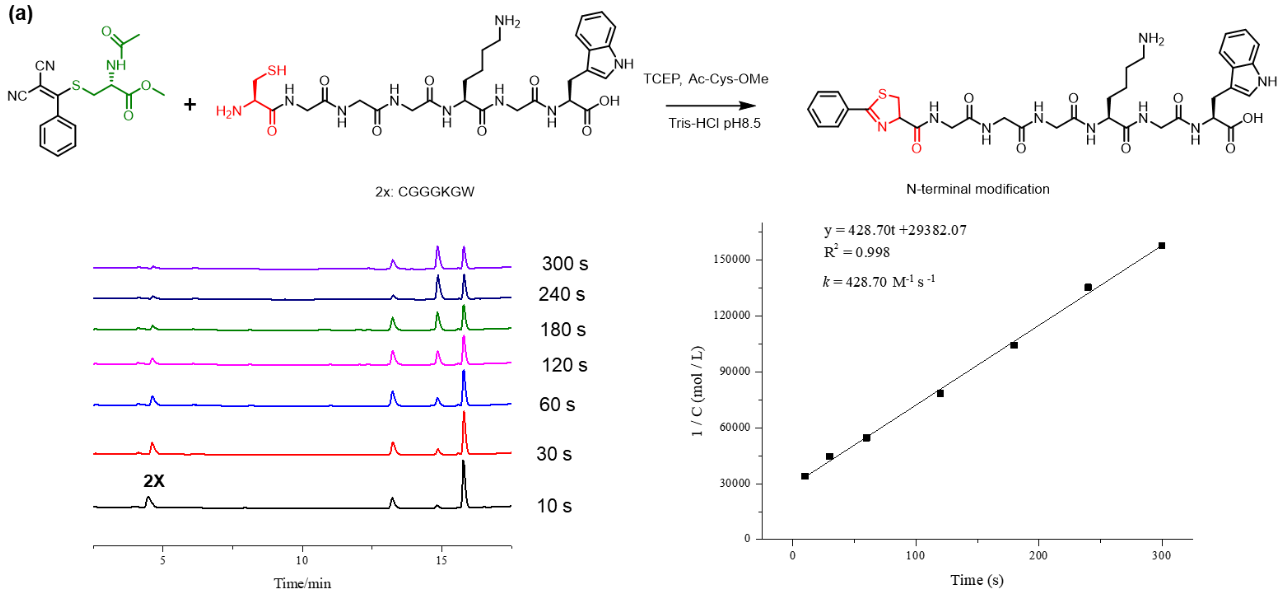
TCEP, Ac-Cys-OMe
Tris-HCl pH8.5
2x: CGGGKGW
N-terminal modification
300 s
240 s
180 s
120 s
60 s
30 s
10 s
2X
Time/min
y = 428.70t +29382.07
R^2 = 0.998
k = 428.70 $M^{-1} s^{-1}$
1 / C (mol / L)
Time (s)

(b)

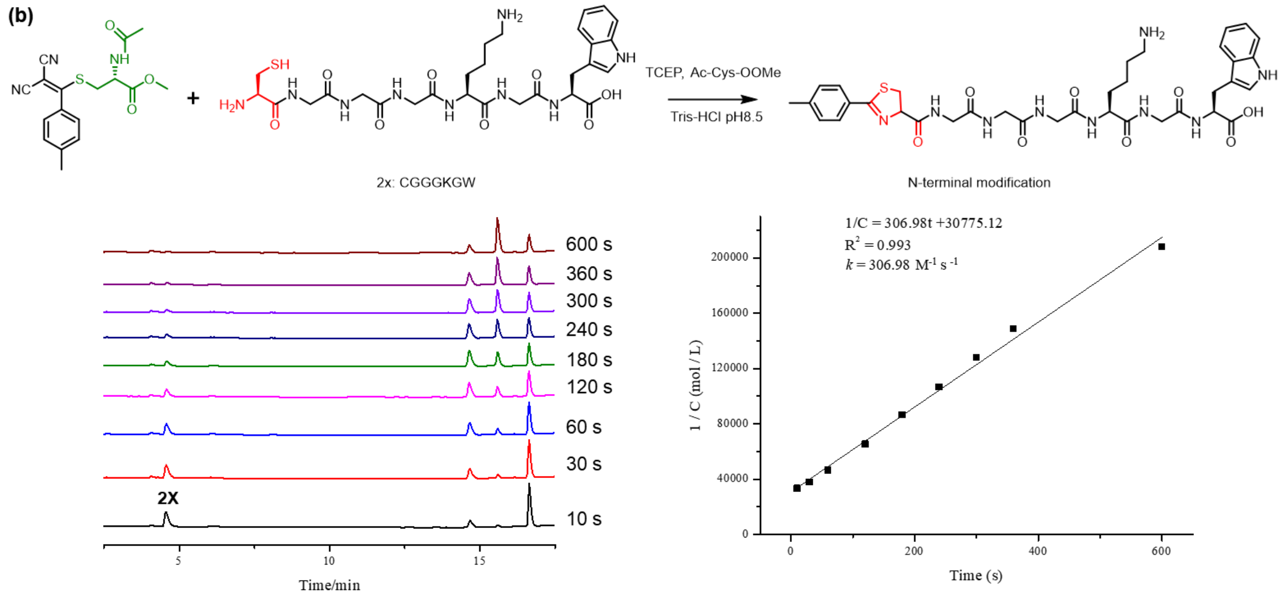
TCEP, Ac-Cys-OOMe
Tris-HCl pH8.5
2x: CGGGKGW
N-terminal modification
600 s
360 s
300 s
240 s
180 s
120 s
60 s
30 s
10 s
2X
Time/min
1/C = 306.98t +30775.12
R^2 = 0.993
k = 306.98 $M^{-1} s^{-1}$
1 / C (mol / L)
Time (s)

(c)

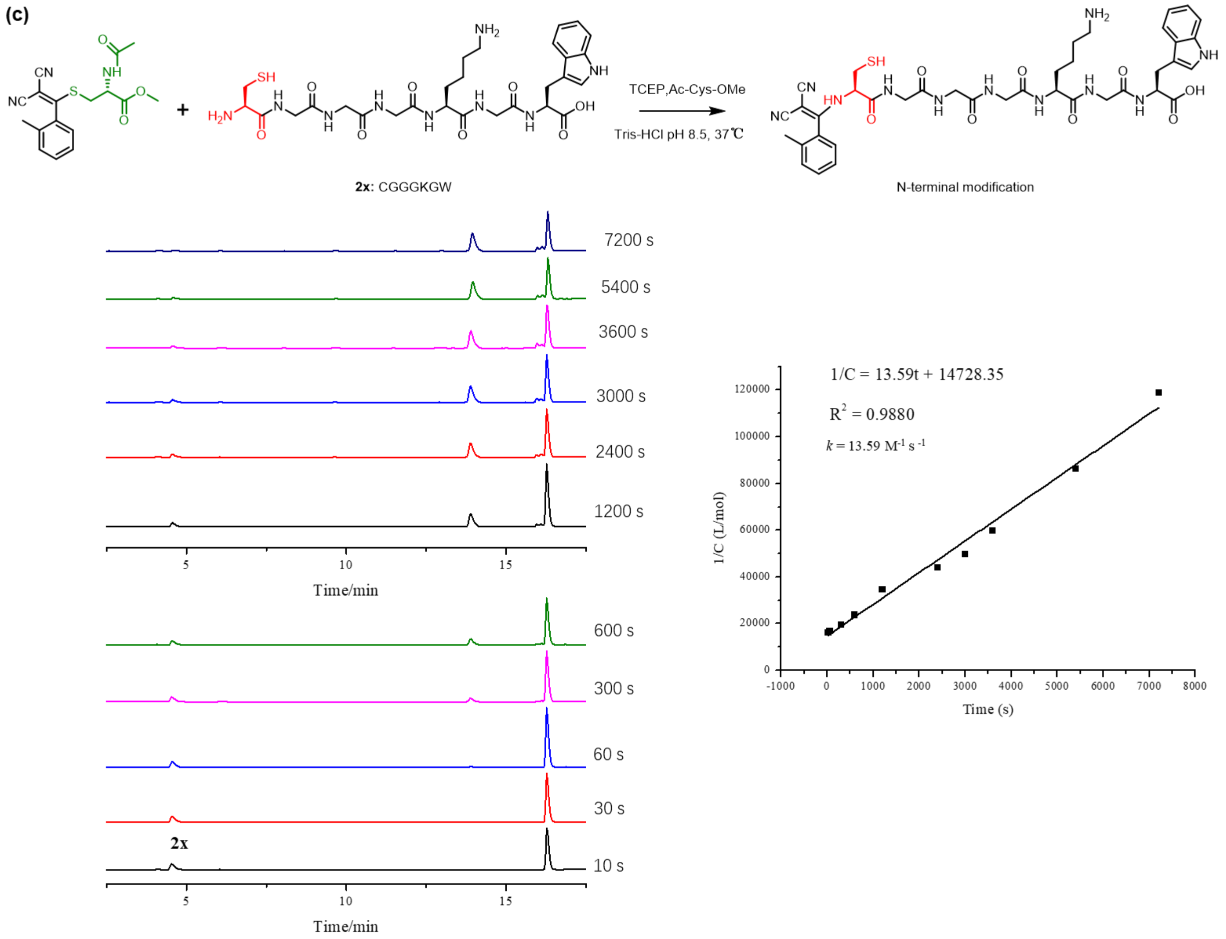

(d)

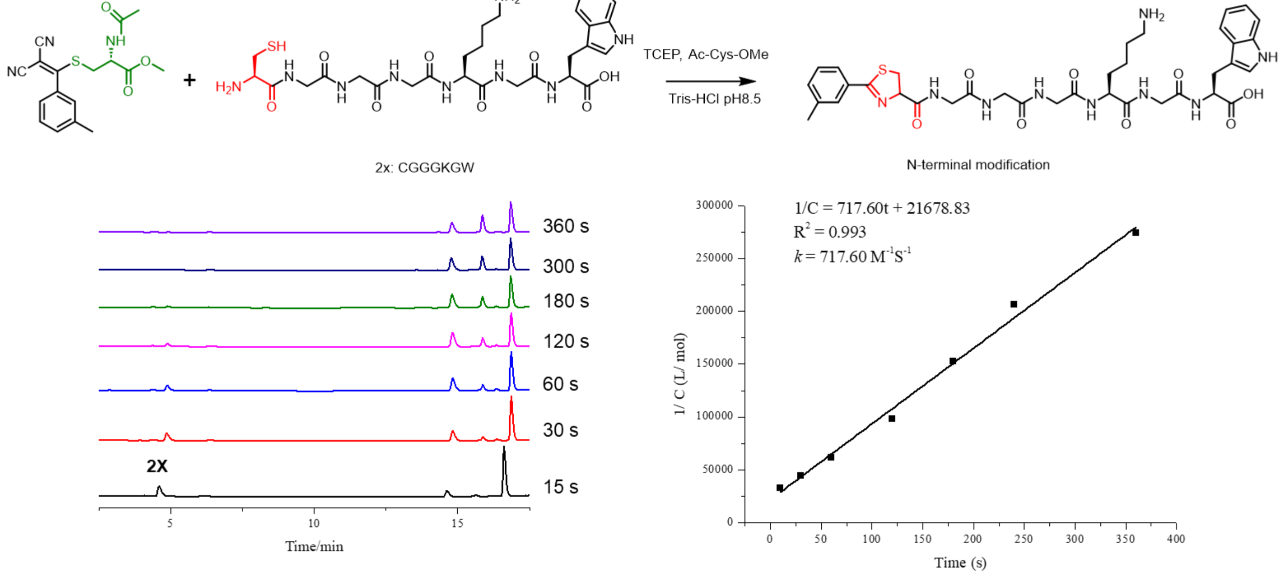

Figure S22. Rate constants of different TAMM molecules reacting with 2x

HPLC chromatograms and the reaction rate equation showing the reaction of 50 μM peptide **2x** with 100 μM of different **TAMM** compounds in 10 mM Tris-HCl (pH 8.5) containing 500 μM TCEP and 500 μM Ac-Cys-OMe under chromatography condition A.

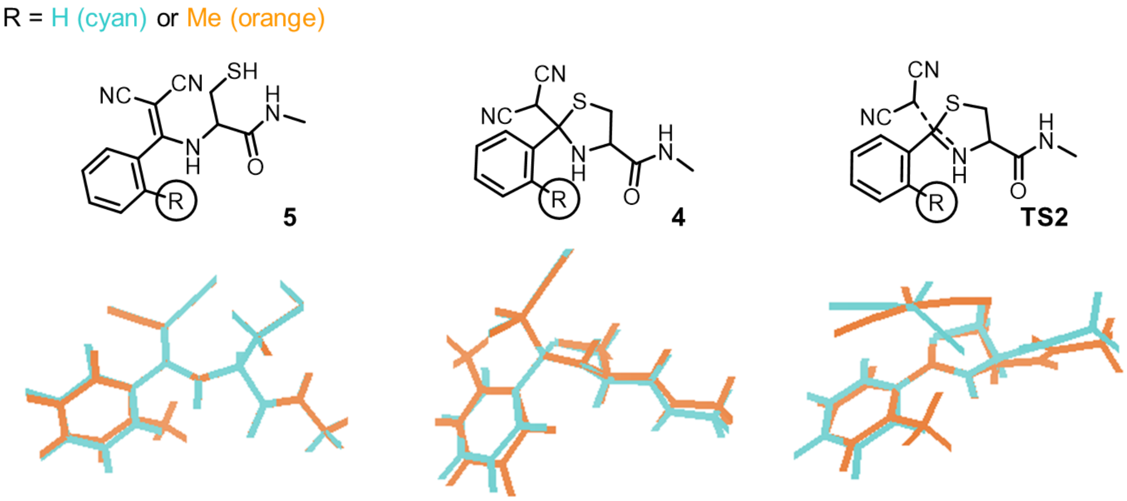

Figure S23. Lowest energy conformers of 4, 5, and TS2 by DFT calculation

The lowest energy states/conformers of unsubstituted **4xa**/**5xa**/TS2-**xa** and *ortho*-methyl **4ya**/**5ya**/**TS2-ya**.

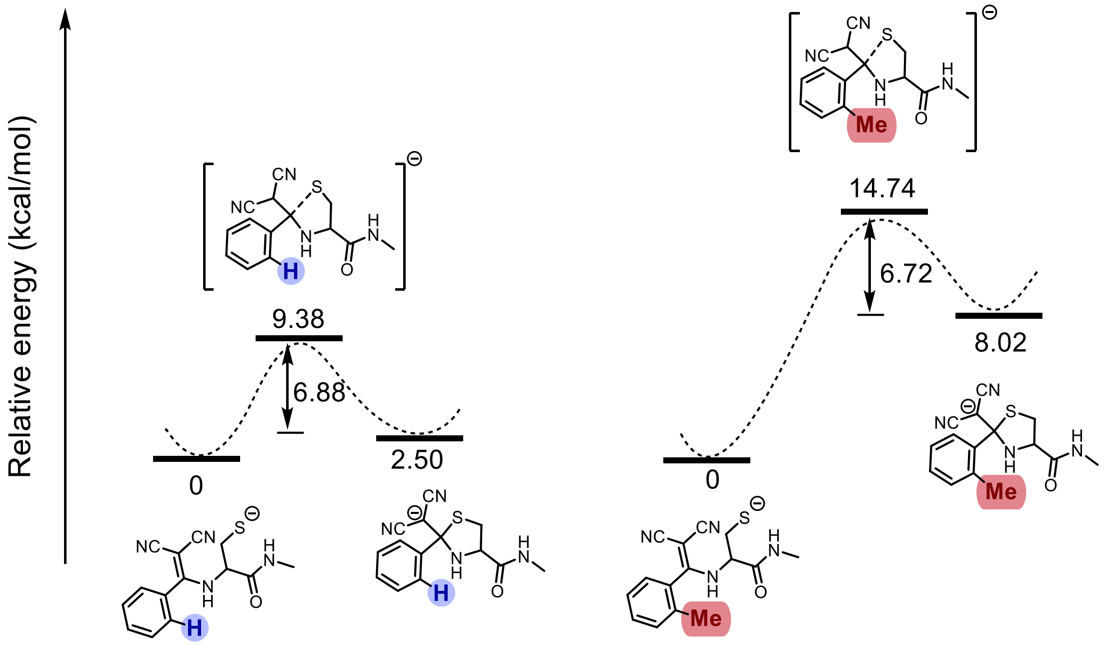

Figure S24. Computed free energy profiles for deprotonated anionic 5 and 4

To complete the relative energy profile for neutral-species interconversion in **Fig. 2g**, the computed activation energies for the deprotonated **5** and **4** were adopted as estimates. These calculations show that the *ortho*-methyl substituent significantly stabilizes the enamine **5** relative to the cyclic **4**, thereby increasing the barrier for interconversion and favoring persistence of the thiol-retaining enamine **5**. This result is consistent with the overall conclusion that *ortho* substitution disfavors progression toward the thiol-consuming dihydrothiazole pathway. A transition state for the corresponding neutral species could not be located, which is also consistent with our previous mechanistic analysis indicating that thiolate, rather than thiol, is the reactive species in this transformation.

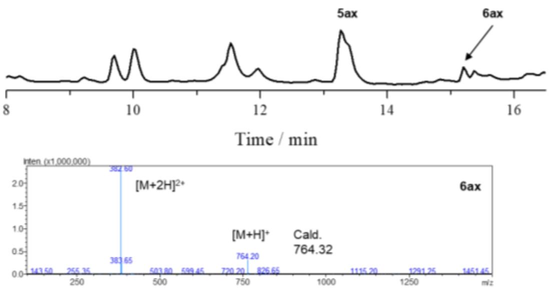

Figure S25. Transformation of 5ax to 6ax

HPLC chromatograms showing the reaction of 50 µM peptide **2x** and 400 µM **1j** in 0.1 M $NaHCO_{3(aq)}$ containing 500 µM TCEP and 1 mM Ac-Cys-OMe under chromatography condition A.

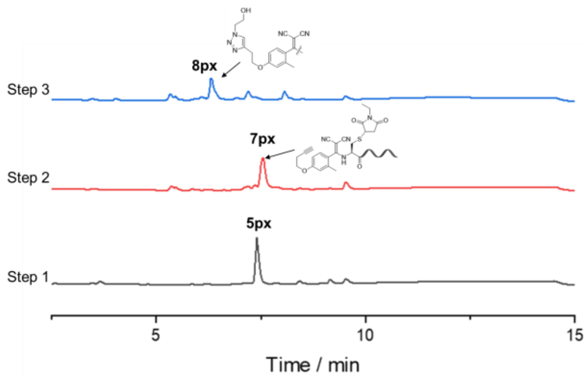

Figure S26. HPLC chromatograms for the dual functionalization of 2x

HPLC chromatograms showing step 1, reaction of **2x** (50 µM) with **1p** (100 µM, 2 eq) in 0.1 M $NaHCO_{3(aq)}$ in the presence of TCEP (1 mM, 20 eq) and Ac-Cys-OMe (0.5 mM, 10 eq) to form 5px; step 2, reaction of the obtained **5px** with maleimide (1 mM) in 0.1 M $NaHCO_{3(aq)}$ to form **7px**; and step 3, reaction of the obtained **7px** with 2-azidoethanol in the presence of 1 mM $CuSO_4$ and 1 mM BTTAA to form **8px**. Chromatography condition B.

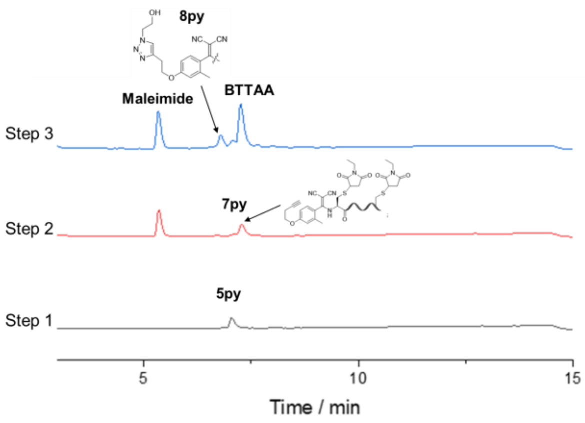

Figure S27. HPLC chromatograms for the dual functionalization of 2y

HPLC chromatograms showing step 1, reaction of **2y** (50 μM) with **1n** (100 μM, 2 eq) in 0.1 M $NaHCO_{3(aq)}$ in the presence of TCEP (1 mM, 20 eq) and Ac-Cys-OMe (0.5 mM, 10 eq) to form **5py**; step 2, reaction of the obtained **5py** with maleimide (1 mM) in 0.1 M $NaHCO_{3(aq)}$ to form **7py**; and step 3, reaction of the obtained **7py** with 2-azidoethanol in the presence of 1 mM $CuSO_4$ and 1 mM BTTAA to form **8py**. Chromatography condition B.

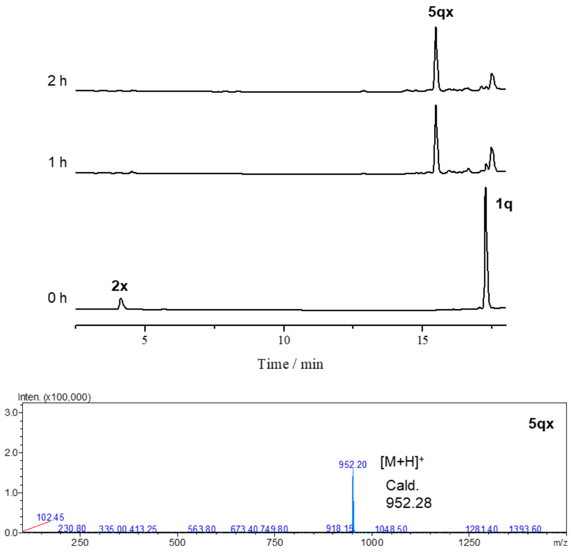

Figure S28. Reaction between 2x and 1q

HPLC chromatograms showing the reaction of **2x** (50 μM) and **1q** (200 μM, 4 eq) in 0.1 M $NaHCO_{3(aq)}$ in the presence of TCEP (0.5 mM, 10 eq) and Ac-Cys-OMe (1 mM, 20 eq) to form **5qx**. Chromatography condition A.

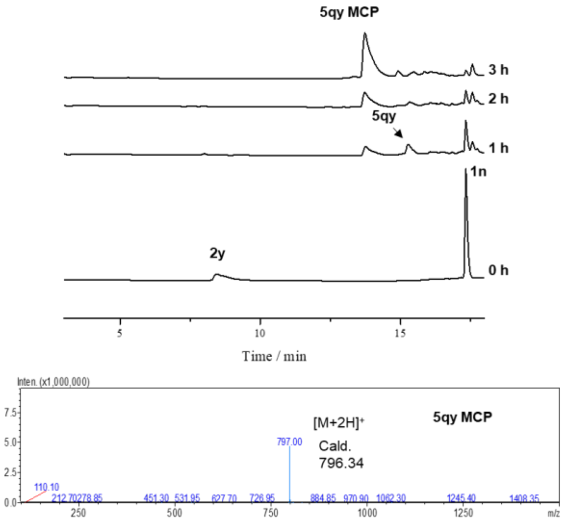

Figure S29. Reaction between 2y and 1q

HPLC chromatograms showing the reaction of **2y** (50 μM) and **1q** (200 μM, 4 eq) in 0.1 M $NaHCO_{3(aq)}$ in the presence of TCEP (0.5 mM, 10 eq) and Ac-Cys-OMe (1 mM, 10 eq) to form **5qy** and **5qy MCP**. Chromatography condition A.

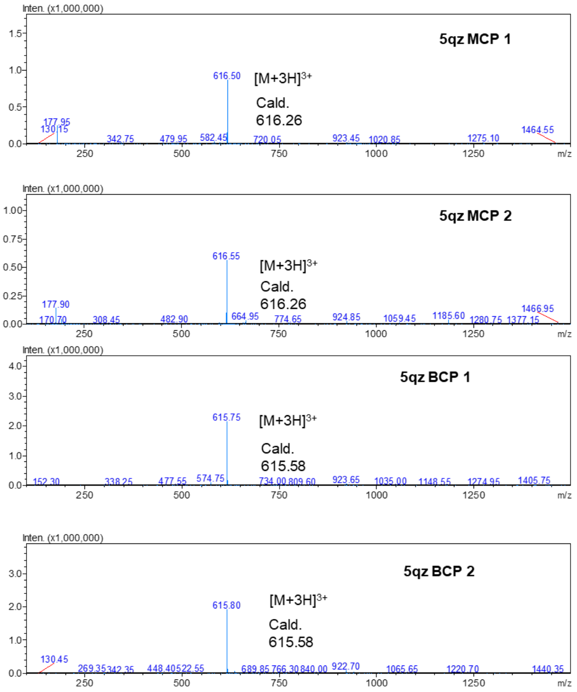

Figure S30. Mass spectra of bicyclic peptide construction

Mass spectra of **5qz MCPs** and **5qz BCPs**.

Round	Recovery rate*		Enrichment
	Control	Experimental	
1st	1.20×10^{-7}	2.40×10^{-8}	0.2
2nd	1.79×10^{-7}	1.06×10^{-4}	593.2
3rd	2.09×10^{-6}	3.43×10^{-3}	1641.1

*Recovery rate refers to the ratio of the phage titer recovered from either the experimental group (magnetic beads with immobilized target proteins) or the control group (magnetic beads without target proteins) relative to the initial phage titer that was introduced into the system. Enrichment = Experimental / Control.

Figure S31. Enrichment from KEAP1 selection

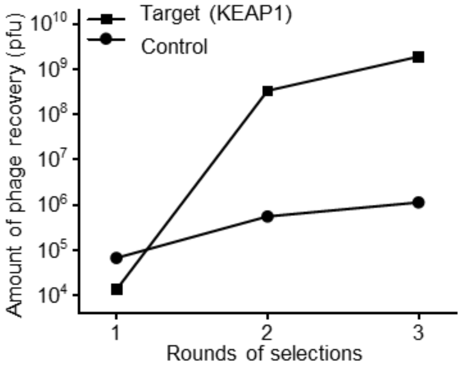

Figure S32. Recovered phages after iterative selection against KEAP1

Target protein KEAP1 (squares) and negative control (circles).

next-generation sequencing of the 1st round

NO.	Sequence	Abundance	Percentage(%)
A1-1	CTGWEPETGECRETQC	730195	6.03%
A1-2	CHNPATGELVCESACC	103814	0.86%
A1-3	CETSPETGETCGDSEC	55648	0.46%
A1-4	CVNGRYMLRICGRTGL	39389	0.33%
A1-5	CRAPHPVTGVCLTYEC	38619	0.32%
A1-6	CPALLPDTGECEEELC	23728	0.20%
A1-7	CTGHIRMRHECSPCCC	18248	0.15%
A1-8	CQVLVVDTGECECRSC	17764	0.15%
A1-9	CRDVETGELTCPYPEC	15169	0.13%
A1-10	CQGFRGRKQSCTSWEC	14354	0.12%
A1-11	CAGPKESMPTCGDGEC	12891	0.11%
A1-12	CHIQLETGESCAGERG	12683	0.10%
A1-13	CGDKAEWRAGCLFAEC	12641	0.10%
A1-14	CSGDCCELGACSEQPC	12416	0.10%
A1-15	CNMYDRDTGECGTYEC	10723	0.09%
A1-16	CHGLMRREWACGHGMC	10140	0.08%
A1-17	CNNSDPETGECRREQC	9940	0.08%
A1-18	CVRIVGWLEECFGRTC	9790	0.08%
A1-19	CLARNPEDGVCHVELC	9647	0.08%
A1-20	CNLETGIDCECTRPEC	9328	0.08%

next-generation sequencing of the 2nd round

NO.	Sequence	Abundance	Percentage(%)
A2-1	CTGWEPETGECRETQC	8092190	58.83%
A2-2	CETSPETGETCGDSEC	1135923	8.26%
A2-3	CHNPATGELVCESACC	546924	3.98%
A2-4	CNNSDPETGECRREQC	494210	3.59%
A2-5	CRDVETGELTCPYPEC	435808	3.17%
A2-6	CLRDPETGENCPESSC	206927	1.50%
A2-7	CRWEHPSTGECEVQAC	172876	1.26%
A2-8	CPALLPDTGECEEELC	158791	1.15%
A2-9	CMDLETGERSCNRVEC	105732	0.77%
A2-10	CYSESPETGECMAWDC	95988	0.70%
A2-11	CEREMETGEACGCFEC	88349	0.64%
A2-12	CNMYDRDTGECGTYEC	82765	0.60%
A2-13	CYTVHPENGECLCREC	65038	0.47%
A2-14	CQVLVVDTGECECRSC	62313	0.45%
A2-15	CSWLDHENGECVEERC	62194	0.45%
A2-16	CRDEATGELVCGELGC	54911	0.40%
A2-17	CLARNPEDGVCHVELC	46969	0.34%
A2-18	CMPDTGEGCECIPSTC	46451	0.34%
A2-19	CIDQESGEDVCKSFEC	46283	0.34%
A2-20	CESYDPEDGTCSALRC	41605	0.30%

next-generation sequencing of the 3rd round

NO.	Sequence	Abundance	Percentage(%)
A3-1	CRDVETGELTCPYPEC	2159914	18.49%
A3-2	CETSPETGETCGDSEC	1807262	15.47%
A3-3	CNNSDPETGECRREQC	1679681	14.38%
A3-4	CTGWEPETGECRETQC	1348847	11.55%
A3-5	CLRDPETGENCPESSC	605379	5.18%
A3-6	CMPDTGEGCECIPSTC	362892	3.11%
A3-7	CHNPATGELVCESACC	251011	2.15%
A3-8	CMDLETGERSCNRVEC	207638	1.78%
A3-9	CYSESPETGECMAWDC	141414	1.21%
A3-10	CRWEHPSTGECEVQAC	110853	0.95%
A3-11	CINLETGESQCEMAEC	100605	0.86%
A3-12	CSRDPETGEGCEYKNC	92353	0.79%
A3-13	CYTVHPENGECLCREC	88666	0.76%
A3-14	CYDVGPETGECEGECC	76175	0.65%
A3-15	CIDQESGEDVCKSFEC	63227	0.54%
A3-16	CEREMETGEACGCFEC	62832	0.54%
A3-17	CNMYDRDTGECGTYEC	54936	0.47%
A3-18	CNLETGIDCECTRPEC	52089	0.45%
A3-19	CSWLDHENGECVEERC	47400	0.41%
A3-20	CDIETGVECDCLYDRC	35205	0.30%

Figure S33. HTS results of phages enriched after three rounds of KEAP1 selection

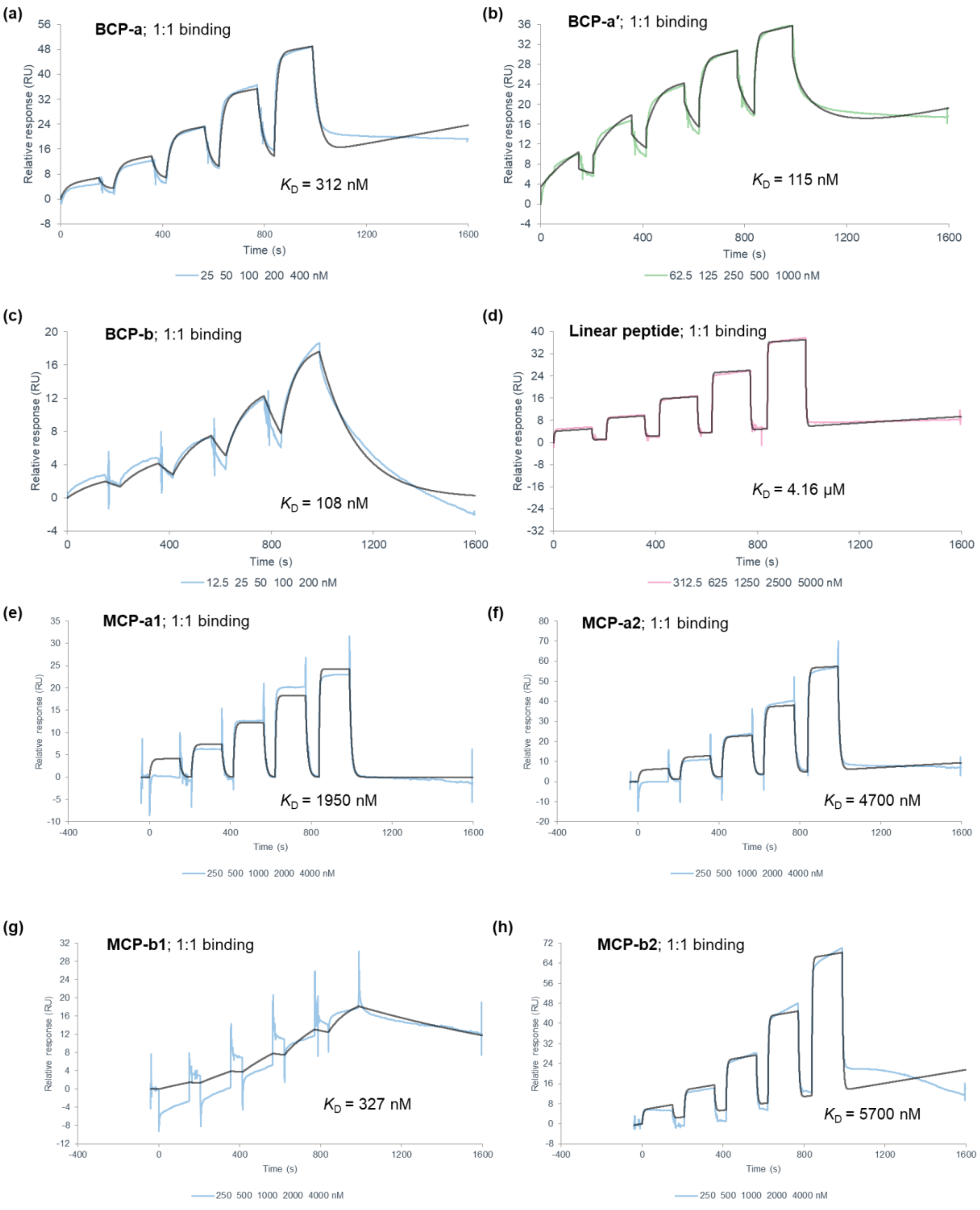

Figure S34. SPR results of different peptide variants binding to KEAP1

Type		Control (pfu)	Experimental (pfu)	Input (pfu)
**A3-4 (unmodified)**	**Bound phage**	N.D.	7.38E+07	4.35E+09
	**Unbound phage**	5.12E+09	4.64E+09	
**A3-4 (bicyclic)**	**Bound phage**	N.D.	1.85E+08	3.84E+09
	**Unbound phage**	4.64E+09	4.68E+09	

Figure S35. Validation of A3-4 bicyclization on phage surface

The experimental methods were consistent with the phage display screening. The experimental group consists of phages harboring the A3-4 sequence modified by bicyclization; the control group comprises the unmodified A3-4 sequence, with all other treatments identical to those of the experimental group.

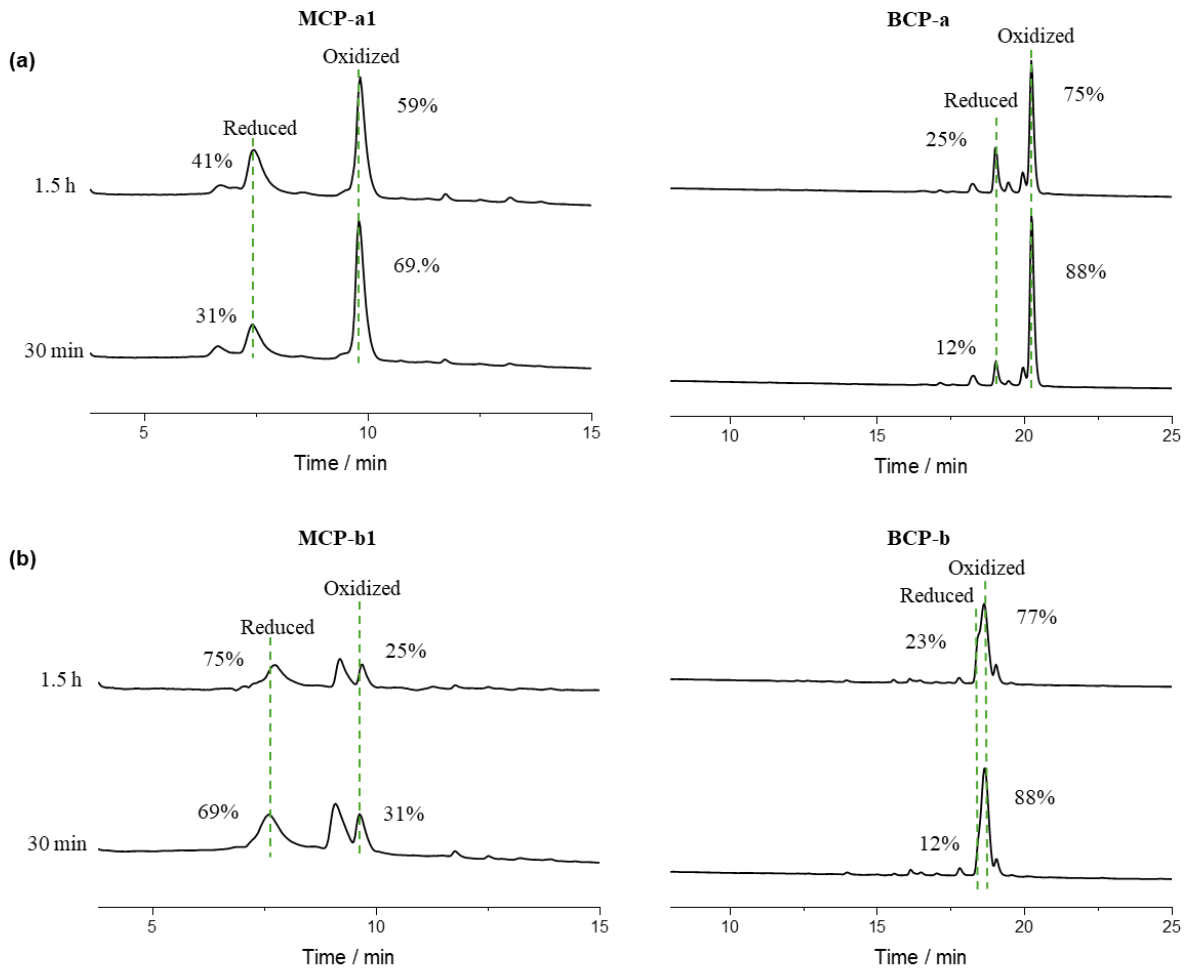

Figure S36. Ring opening of BCPs and MCPs

BCP-a (a) and **BCP-b** (b), along with their corresponding **MCPs**, were evaluated by HPLC using reactions of 50 μM peptides and 1.5 mM GSH under 37 °C in PBS. Upon sample collection, 5 mM iodoacetamide was added and allowed to react with the reduced peptides for 5 min, followed by quenching with 1 N HCl. HPLC chromatograms showing the reactions under chromatography condition E.

(a)

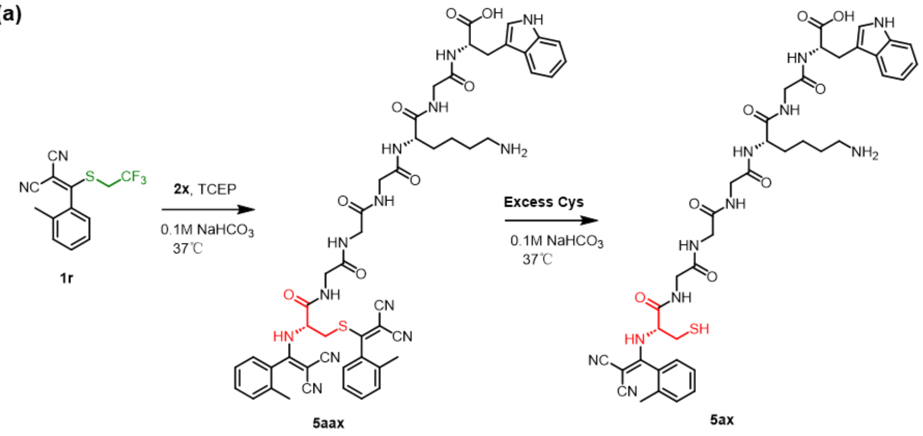

(b)

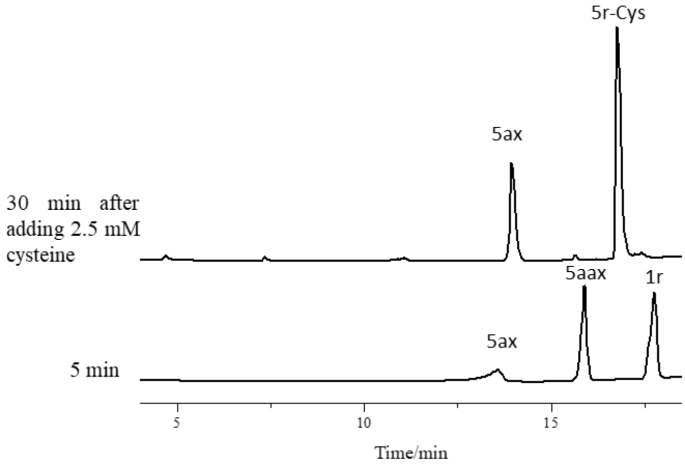

Figure S37. Reaction of 1r and 2x

Conversions were quantified by HPLC for reactions of 200 μM **1r** with 50 μM **2x** in the presence of 1 mM TCEP under 37 °C in 0.1 M $NaHCO_{3(aq)}$. HPLC chromatograms showing the reactions under chromatography condition A.

Supplementary Tables

Table S1. Conversions of 1a–1k reaction with 2x

Entry	R =	Condition	Conversion (2 h)	Conversion (6 h)	Conversion (12 h)	Conversion (24 h)
**1a**	2-methylphenyl	0.1 M $NaHCO_{3(aq)}$<br>37 °C	93% **5ax**<br>0% **6ax**	83% **5ax**<br>0% **6ax**	60% **5ax**<br>0% **6ax**	43% **5ax**<br>0% **6ax**
		0.1 M $NaHCO_{3(aq)}$<br>25 °C	87% **5ax**<br>0% **6ax**	81% **5ax**<br>0% **6ax**	73% **5ax**<br>0% **6ax**	68% **5ax**<br>0% **6ax**
		PBS<br>37 °C	45% **5ax**<br>0% **6ax**	47% **5ax**<br>0% **6ax**	42% **5ax**<br>0% **6ax**	29% **5ax**<br>0% **6ax**
**1b**	2-trifluoromethylphenyl	0.1 M $NaHCO_{3(aq)}$<br>37 °C	22% **5bx**<br>0% **6bx**	19% **5bx**<br>0% **6bx**	14% **5bx**<br>0% **6bx**	1% **5bx**<br>0% **6bx**
		0.1 M $NaHCO_{3(aq)}$<br>25 °C	15% **5bx**<br>0% **6bx**	18% **5bx**<br>0% **6bx**	14% **5bx**<br>0% **6bx**	12% **5bx**<br>0% **6bx**
		PBS<br>37 °C	5% **5bx**<br>0% **6bx**	6% **5bx**<br>0% **6bx**	7% **5bx**<br>0% **6bx**	6% **5bx**<br>0% **6bx**
**1c**	2-isopropyl	0.1 M $NaHCO_{3(aq)}$<br>37 °C	60% **5cx**<br>0% **6cx**	61% **5cx**<br>0% **6cx**	53% **5cx**<br>0% **6cx**	37% **5cx**<br>0% **6cx**
		0.1 M $NaHCO_{3(aq)}$<br>25 °C	45% **5cx**<br>0% **6cx**	60% **5cx**<br>0% **6cx**	61% **5cx**<br>0% **6cx**	56% **5cx**<br>0% **6cx**
		PBS<br>37 °C	26% **5cx**<br>0% **6cx**	30% **5cx**<br>0% **6cx**	23% **5cx**<br>0% **6cx**	9% **5cx**<br>0% **6cx**
**1d**	2-nitrophenyl	0.1 M $NaHCO_{3(aq)}$<br>37 °C	28% **5dx**<br>0% **6dx**	15% **5dx**<br>0% **6dx**	6% **5dx**<br>0% **6dx**	0% **5dx**<br>0% **6dx**
		0.1 M $NaHCO_{3(aq)}$<br>25 °C	25% **5dx**<br>0% **6dx**	24% **5dx**<br>0% **6dx**	16% **5dx**<br>0% **6dx**	1% **5dx**<br>0% **6dx**
		PBS<br>37 °C	64% **5dx**<br>0% **6dx**	50% **5dx**<br>0% **6dx**	33% **5dx**<br>0% **6dx**	14% **5dx**<br>0% **6dx**
**1e**	2-iodophenyl	0.1 M $NaHCO_{3(aq)}$<br>37 °C	55% **5ex**<br>0% **6ex**	18% **5ex**<br>0% **6ex**	0% **5ex**<br>0% **6ex**	0% **5ex**<br>0% **6ex**
		0.1 M $NaHCO_{3(aq)}$<br>25 °C	44% **5ex**<br>0% **6ex**	39% **5ex**<br>0% **6ex**	28% **5ex**<br>0% **6ex**	17% **5ex**<br>0% **6ex**
		PBS<br>37 °C	34% **5ex**<br>0% **6ex**	28% **5ex**<br>0% **6ex**	20% **5ex**<br>0% **6ex**	0% **5ex**<br>0% **6ex**
**1f**	2-bromophenyl	0.1 M $NaHCO_{3(aq)}$<br>37 °C	72% **5fx**<br>0% **6fx**	43% **5fx**<br>0% **6fx**	24% **5fx**<br>0% **6fx**	6% **5fx**<br>0% **6fx**
		0.1 M $NaHCO_{3(aq)}$<br>25 °C	58% **5fx**<br>0% **6fx**	51% **5fx**<br>0% **6fx**	43% **5fx**<br>0% **6fx**	26% **5fx**<br>0% **6fx**
		PBS<br>37 °C	51% **5fx**<br>0% **6fx**	28% **5fx**<br>0% **6fx**	26% **5fx**<br>0% **6fx**	8% **5fx**<br>0% **6fx**
**1g**	2-chlorophenyl	0.1 M $NaHCO_{3(aq)}$<br>37 °C	73% **5gx**<br>0% **6gx**	44% **5gx**<br>0% **6gx**	23% **5gx**<br>0% **6gx**	8% **5gx**<br>0% **6gx**
		0.1 M $NaHCO_{3(aq)}$<br>25 °C	55% **5gx**<br>0% **6gx**	53% **5gx**<br>0% **6gx**	40% **5gx**<br>0% **6gx**	24% **5gx**<br>0% **6gx**
		PBS<br>37 °C	51% **5gx**<br>9% **6gx**	30% **5gx**<br>17% **6gx**	17% **5gx**<br>24%**6gx**	4% **5gx**<br>26% **6gx**
**1h**	2-fluorophenyl	0.1 M $NaHCO_{3(aq)}$<br>37 °C	51% **5hx**<br>36% **6hx**	22% **5hx**<br>31% **6hx**	22% **5hx**<br>29% **6hx**	21% **5hx**<br>29% **6hx**
		0.1 M $NaHCO_{3(aq)}$<br>25 °C	77% **5hx**<br>14% **6hx**	57% **5hx**<br>23% **6hx**	44% **5hx**<br>29% **6hx**	30% **5hx**<br>28% **6hx**
		PBS<br>37 °C	0% **5hx**<br>90% **6hx**	0% **5hx**<br>90% **6hx**	0% **5hx**<br>92% **6hx**	0% **5hx**<br>90% **6hx**
**1i**	2-methoxyphenyl	0.1 M $NaHCO_{3(aq)}$<br>37 °C	58% **5ix**<br>34% **6ix**	16% **5ix**<br>51% **6ix**	0% **5ix**<br>63% **6ix**	0% **5ix**<br>58% **6ix**
		0.1 M $NaHCO_{3(aq)}$<br>25 °C	90% **5ix**<br>10% **6ix**	72% **5ix**<br>14% **6ix**	46% **5ix**<br>23% **6ix**	23% **5ix**<br>40% **6ix**
		PBS<br>37 °C	20% **5ix**<br>49% **6ix**	0% **5ix**<br>70% **6ix**	0% **5ix**<br>73% **6ix**	0% **5ix**<br>72% **6ix**
**1j**	*tert*-butyl	0.1 M $NaHCO_{3(aq)}$<br>37 °C	0% **5jx**<br>86% **6jx**	0% **5jx**<br>87% **6jx**	0% **5jx**<br>82% **6jx**	0% **5jx**<br>82% **6jx**
		0.1 M $NaHCO_{3(aq)}$<br>25 °C	0% **5jx**<br>82% **6jx**	0% **5jx**<br>77% **6jx**	0% **5jx**<br>74% **6jx**	0% **5jx**<br>74% **6jx**
		PBS<br>37 °C	0% **5jx**<br>95% **6jx**	0% **5jx**<br>93% **6jx**	0% **5jx**<br>90% **6jx**	0% **5jx**<br>90% **6jx**
**1k**	neopentyl	0.1 M $NaHCO_{3(aq)}$<br>37 °C	95% **5kx**<br>5% **6kx**	57% **5kx**<br>9% **6kx**	33% **5kx**<br>14% **6kx**	11% **5kx**<br>17% **6kx**
		0.1 M $NaHCO_{3(aq)}$<br>25 °C	96% **5kx**<br>4% **6kx**	86% **5kx**<br>5% **6kx**	70% **5kx**<br>5% **6kx**	53% **5kx**<br>15% **6kx**
		PBS<br>37 °C	63% **5kx**<br>7% **6kx**	34% **5kx**<br>14% **6kx**	16% **5kx**<br>19% **6kx**	6% **5kx**<br>27% **6kx**

Table S2. Comparison of rate constants

Reagent	Rate Constant (M^{-1} s^{-1}) TRIS (pH 8.5), 37 °C	Rate Constant (M^{-1} s^{-1}) PBS (pH 7.4), 25 °C	Reference*
o-TAMM **1a**	14	Est. 0.1[a]	This & Ref. 21
p-TAMM **1o**	310	Est. 3[a]	This & Ref. 21
o-Me-TAMM-SCH_2CF_3	n.d.	Est. 40[b]	This & Ref. 21
CBT	n.d.	1–10	Ref. 13
DPCP	n.d.	1–1000	Ref. 39
NCL	n.d.	0.1–10	Ref. 41/42

*Reference numbering refers to those listed in the manuscript.

Reference

(1) Bannwarth, C.; Ehlert, S.; Grimme, S. GFN2-xTB—An Accurate and Broadly Parametrized Self-Consistent Tight-Binding Quantum Chemical Method with Multipole Electrostatics and Density-Dependent Dispersion Contributions. *J. Chem. Theory Comput.* **2019,** *15* (3), 1652–1671.
(2) Paranthaman, S.; Hong, K.; Kim, J.; Kim, D. E.; Kim, T. K. Density functional theory assessment of molecular structures and energies of neutral and anionic Al(n) (n = 2-10) clusters. *J. Phys. Chem. A* **2013,** *117* (38), 9293–303.
(3) Neese, F. Software update: The ORCA program system-Version 5.0. *WIREs Comput. Mol. Sci.* **2022,** *12* (5), e1606.
(4) Lu, T.; Chen, Q. X. Shermo: A general code for calculating molecular thermochemistry properties. *Comput. Theor. Chem.* **2021,** *1200*, 113249.
(5) Zheng, X.; Li, Z.; Gao, W.; Meng, X.; Li, X.; Luk, L. Y. P.; Zhao, Y.; Tsai, Y.-H.; Wu, C. Condensation of 2-((alkylthio)(aryl)methylene)malononitrile with 1,2-aminothiol as a novel bioorthogonal reaction for site-specific protein modification and peptide cyclization. *J. Am. Chem. Soc.* **2020,** *142* (11), 5097–5103.
(6) Zha, M.; Lin, P.; Yao, H.; Zhao, Y.; Wu, C. A phage display-based strategy for the de novo creation of disulfide-constrained and isomer-free bicyclic peptide affinity reagents. *Chem. Commun.* **2018,** *54* (32), 4029–4032.
